# Non-equilibrium transport in polymer mixed ionic–electronic conductors at ultrahigh charge densities

**DOI:** 10.1038/s41563-024-01953-6

**Published:** 2024-07-26

**Authors:** Dionisius H. L. Tjhe, Xinglong Ren, Ian E. Jacobs, Gabriele D’Avino, Tarig B. E. Mustafa, Thomas G. Marsh, Lu Zhang, Yao Fu, Ahmed E. Mansour, Andreas Opitz, Yuxuan Huang, Wenjin Zhu, Ahmet Hamdi Unal, Sebastiaan Hoek, Vincent Lemaur, Claudio Quarti, Qiao He, Jin-Kyun Lee, Iain McCulloch, Martin Heeney, Norbert Koch, Clare P. Grey, David Beljonne, Simone Fratini, Henning Sirringhaus

**Affiliations:** 1 Cavendish Laboratory, University of Cambridge, Cambridge, UK; 2 Grenoble Alpes University, CNRS, Grenoble INP, Institut Néel, Grenoble, France; 3 Department of Chemistry, University of Cambridge, Cambridge, UK; 4 Institut für Physik and Center for the Science of Materials Berlin, Humboldt-Universität zu Berlin, Berlin, Germany; 5 Helmholtz-Zentrum Berlin für Materialien und Energie, Berlin, Germany; 6 Laboratory for Chemistry of Novel Materials, University of Mons, Mons, Belgium; 7 Department of Chemistry and Centre for Processable Electronics, Imperial College London, London, UK; 8 Department of Polymer Science and Engineering, Inha University, Incheon, South Korea; 9 Department of Chemistry, University of Oxford, Oxford, UK

**Keywords:** Thermoelectrics, Electronic properties and materials

## Abstract

Conducting polymers are mixed ionic–electronic conductors that are emerging candidates for neuromorphic computing, bioelectronics and thermoelectrics. However, fundamental aspects of their many-body correlated electron–ion transport physics remain poorly understood. Here we show that in p-type organic electrochemical transistors it is possible to remove all of the electrons from the valence band and even access deeper bands without degradation. By adding a second, field-effect gate electrode, additional electrons or holes can be injected at set doping states. Under conditions where the counterions are unable to equilibrate in response to field-induced changes in the electronic carrier density, we observe surprising, non-equilibrium transport signatures that provide unique insights into the interaction-driven formation of a frozen, soft Coulomb gap in the density of states. Our work identifies new strategies for substantially enhancing the transport properties of conducting polymers by exploiting non-equilibrium states in the coupled system of electronic charges and counterions.

## Abstract (web summary)

Mixed ionic–electronic transport in conducting polymers remains poorly understood. Here the authors observe non-equilibrium electronic transport when counterions are unable to equilibrate in response to gate-injected electronic carriers.

## Main

Conducting polymers are an example of a wider class of disordered metals that include liquid alkali metals, metallic glasses and granular metals^1^. Electrons in these materials are localized but correlated due to their Coulomb interactions. As a consequence, a soft Coulomb gap, that is, a suppression of the density of states (DOS) around the Fermi level *E*_F_, is expected to form and manifest itself in the charge-transport properties^2^. Coulomb-gap formation in conducting polymers has been inferred from simulations^3,4^ or from the temperature dependence of conductivity^5,6^, but is hard to study directly in experiments as it tends to be masked by structural heterogeneity^7^. Another consequence of Coulomb interactions, reported in some disordered metals at low temperatures^8,9^, are electron glass signatures in the conductivity, which reflect long-lived, metastable conduction states that do not equilibrate on the timescale of transport measurements. Whether similar ‘glassy’ transport signatures can be observed in conducting polymers has not yet been studied.

Polymers offer two key advantages for a better fundamental understanding of this correlated electron-transport regime at high carrier density: the ability to tune the carrier concentration over a wide range and the ease of integration into devices. In electrolyte-gated organic electrochemical transistors (OECTs), defined doping states can be dialled in by applying an ion gate voltage, *V*_IG_; charge transport at this doping level can then be studied using different techniques^10–13^. There have been recent reports of p-type^14^ and n-type OECTs^4^ in which the valence or conduction bands (derived from the highest (lowest) (un)occupied molecular orbital, HOMO (LUMO)) can be completely emptied (filled). However, in most materials such extreme band filling is limited by electrochemical degradation^15^. Here we show that in some p-type, donor–acceptor polymer-based OECTs, band filling can stably be driven even further to access deeper, HOMO−1-derived bands that cannot be realized in covalently bonded inorganic materials without inducing structural collapse. We also demonstrate that by adding a second, field-effect gate electrode to the OECT ion gate, non-equilibrium states can be induced in the coupled system of electrons and ions, in which the counterions are unable to rearrange in response to field-effect-induced changes in electron density. This approach enables us to study the correlated electron–ion transport physics of conducting polymers over a very wide range of doping levels.

## Extreme band filling in polymer OECTs

**Fig. 1:**
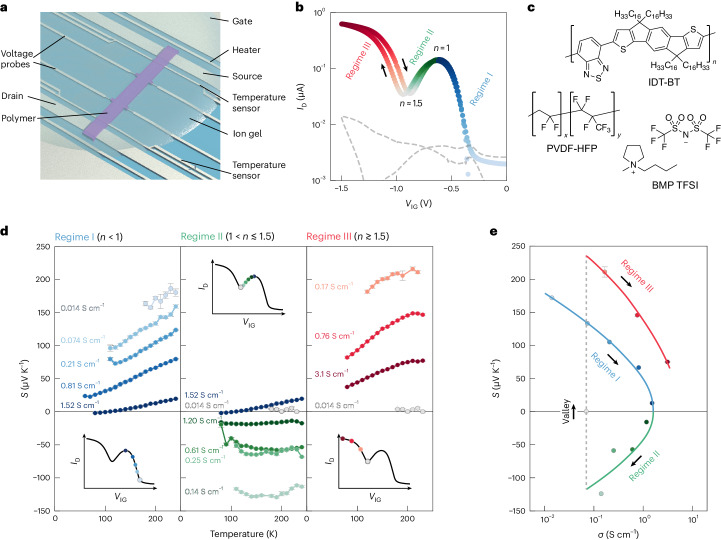
Observation of deep band-filling behaviour in IDT-BT OECTs. **a**, Device structure. **b**, Transfer curve for an IDT-BT OECT, taken at room temperature with a drain voltage *V*_D_ of −0.1 V. The dashed grey line indicates the gate leakage current and the arrow indicates the voltage sweep direction. *n* represents the number of dopant ion per polymer repeat unit. **c**, Chemical structures of IDT-BT, BMP TFSI and PVDF-HFP. *n*, *x*  and *y* represent the number of IDT-BT, PVDF, and HFP repeat units, respectively. **d**, Temperature dependence of the Seebeck coefficient *S* of IDT-BT at various doping levels. These doping levels are shown schematically on an OECT transfer curve in the inset. **e**, Seebeck–conductivity plot at 200 K for the data shown in **d**. The arrows denote the direction of increasing doping level for a given regime. In **d** and **e**, the data are presented as the mean Seebeck coefficient ± standard error of the mean, originating from fitting uncertainties of the on-chip thermometer calibration and the thermovoltage versus temperature difference plots. Lines are included as a guide to the eye.

Our OECTs (Fig. 1a) comprise large side gates to induce ion injection from a solid, non-aqueous ion gel based on 1-butyl-1-methylpyrrolidinium bis(trifluoromethylsulfonyl)imide (BMP TFSI)/poly(vinylidene fluoride-co-hexafluoropropylene) (PVDF-HFP) (Fig. 1c)^16^ on top of the polymer channel (Supplementary Note 1) and multi-functional electrodes for accurate measurements of four-point-probe conductivity *σ* and the Seebeck coefficient *S* (Supplementary Note 2). We focus on three representative widely studied polymers: indacenodithiophene-co-benzothiadiazole (IDT-BT), a donor–acceptor copolymer with high carrier mobilities in field-effect transistors (FETs) due to a low degree of energetic disorder^17^, despite exhibiting a high paracrystallinity (*g* ≈ 25%) and only local chain alignment;^18^ poly(2,5-bis(3-dodecylthiophen-2-yl)thieno(3,2-*b*)thiophene) (PBTTT), a polythiophene-based semicrystalline polymer that can be doped to conductivities of *σ* > 1,000 S cm^−1^;^19^ and poly[[2,5-bis(2-octadecyl)-2,3,5,6-tetrahydro-3,6-diketopyrrolo[3,4-*c*]pyrrole-1,4-diyl]-alt-(2-octylnonyl)-2,1,3-benzotriazole] (DPP-BTz), a semicrystalline copolymer^20^ with *σ* = 200–300 S cm^−1^. We focus on IDT-BT in the main text and discuss the other polymers in Supplementary Notes 3–6.

IDT-BT shows unusual OECT behaviour not reported previously: a peak in the drain current *I*_D_ around *V*_IG_ = −0.6 V is followed by a drop with increasingly negative *V*_IG_. However, rather than reaching the insulating state seen in other polymers^4,14,21^, we observe a second current rise when *V*_IG_ < −1 V (Fig. 1b). We refer to three different transport regimes (colour-coded in Fig. 1 and subsequent figures): Regime I at low doping levels up to the *I*_D_ peak (blue); Regime II between the peak and the valley state (green); and Regime III beyond the valley (red). Despite the high FET mobilities of IDT-BT, room-temperature conductivities at the peak are only 5–10 S cm^−1^, but in Regime III they can exceed 30 S cm^−1^.

We measured the temperature dependence of *σ* and *S* as a function of *V*_IG_. The conductivity *σ* has a non-metallic temperature dependence in all three regimes and reduces strongly with decreasing temperature (Supplementary Note 3.2). However, *S* varies distinctly across the regimes, and provides more direct insight into the electronic structure. In Regime I, *S* is positive; when approaching the peak, it reduces in magnitude and approaches a linear temperature dependence characteristic of metallic transport (Fig. 1d). This is consistent with theoretical predictions of a divergence in the dielectric constant, leading to an insulator–metal transition at relatively low doping levels (*n* ≈ 0.1)^22^. In Regime II, *S* switches from p-type to n-type, increases in magnitude with increasing ∣*V*_IG_∣ and becomes nearly independent of temperature between 100 and 250 K. When sweeping *V*_IG_ across the valley, *S* switches sign again from a large negative to a large positive value. In Regime III, *S* varies again linearly with temperature but is approximately threefold larger at a comparable p-type conductivity than in Regime I (Fig. 1e).

**Extended Data Fig. 1:**
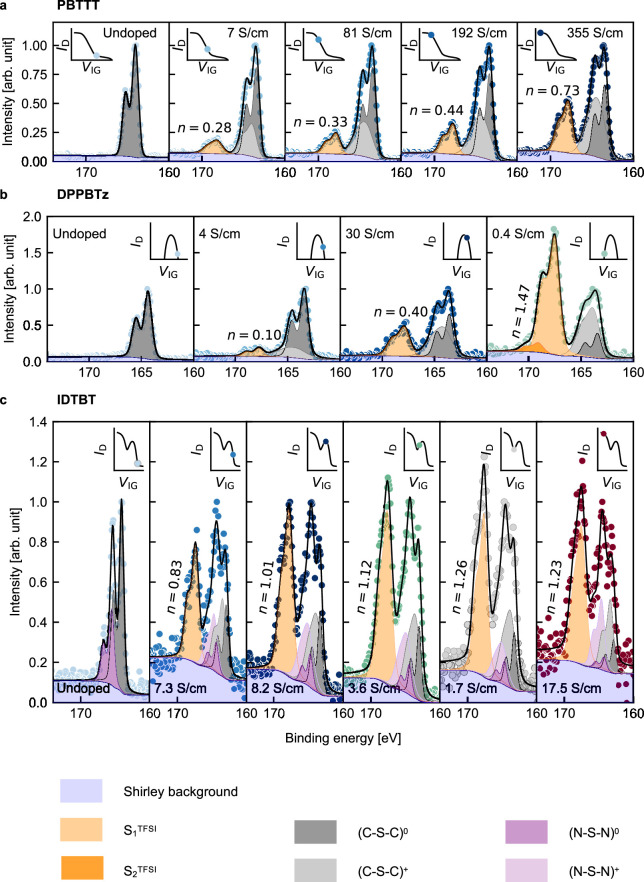
Carrier density analysis based on the comparison of the abundance of sulfur atoms in the polymer backbone and the dopant ion. Sulfur 2p peak in the X-ray photoemission spectra of (**a**) PBTTT, (**b**) DPP-BTz, and (**c**) IDT-BT at various doping levels. The dopant ion peak appears at higher binding energies, denoted as S_TFSI_. The number of dopant ions per polymer repeat unit *n*, determined from integration areas, is indicated on each doped spectrum. Close to the conductivity peak we estimate a 1:1 ratio of TFSI ions to polymer repeat units for all three materials, consistent with half-band filling. The doping level in the valley of IDT-BT is approximately 1.5 ions per repeat unit, indicative of some overlap between HOMO and HOMO-1 bands. Regime III should correspond to carrier densities of up to 3 ions per repeat unit, but direct measurements of these highest doped samples were challenging due to sample dedoping under ultrahigh vacuum. Fitting and analysis details are provided in Supplementary Note 3.4.

**Extended Data Fig. 2:**
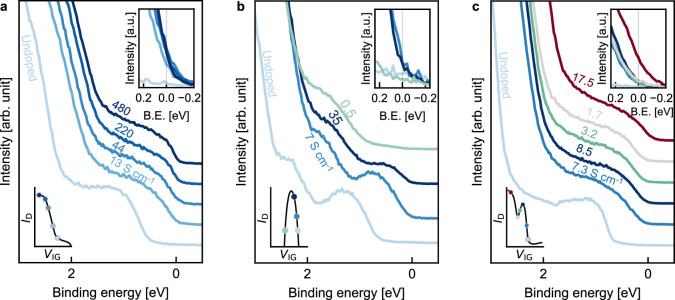
Doping induced changes in the frontier energy levels of conjugated polymers as inferred from ultraviolet photoemission spectroscopy (UPS). UPS spectra of (**a**) PBTTT, (**b**) DPP-BTz, and (**c**) IDT-BT at various doping levels. Top right insets show a detailed view of the Fermi level, highlighting the presence of a finite filled DOS indicative of metallic characteristics in the highly doped samples. In DPP-BTz the HOMO-derived band is completely emptied in the insulating state of Regime II. More detailed discussion is provided in Supplementary Note 3.3.

**Extended Data Fig. 3:**
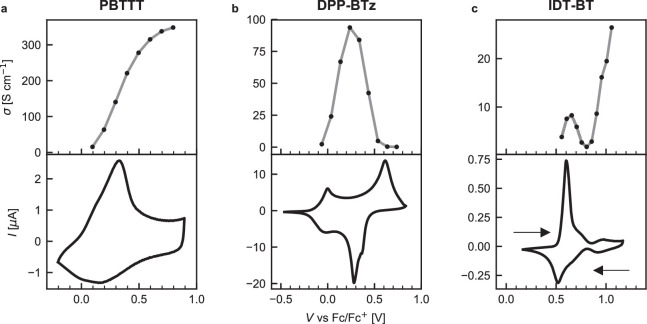
Multiple redox features in DPP-BTz and IDT-BT. Cyclic voltammogram (bottom panels, sweep rate 10 mV s^−1^ for PBTTT and DPP-BTz, 2 mV s^−1^ for IDT-BT) and the corresponding conductivity at various doping stages (top panels) for (**a**) PBTTT, (**b**) DPP-BTz, and (**c**) IDT-BT. In PBTTT, we see only one redox feature at the onset of its Regime I. In DPP-BTz, we see two redox features — one at the onset of Regime I, and another within Regime II. In IDT-BT, we see three redox features corresponding to the three transport regimes — two close together at 0.7 V vs Fc/Fc^+^ coinciding with the conductivity peak between Regimes I and II, and a third one at the onset of Regime III. The observation that the conductivity peak occurs between the first two redox waves — which each appear to be single-electron transfer events — is consistent with our picture of maximum conduction near half-band filling. Note that the voltage in cyclic voltammetry is applied to the polymer, such that the sign of the applied voltage is reversed relative to the OECT measurements.

**Extended Data Fig. 4:**
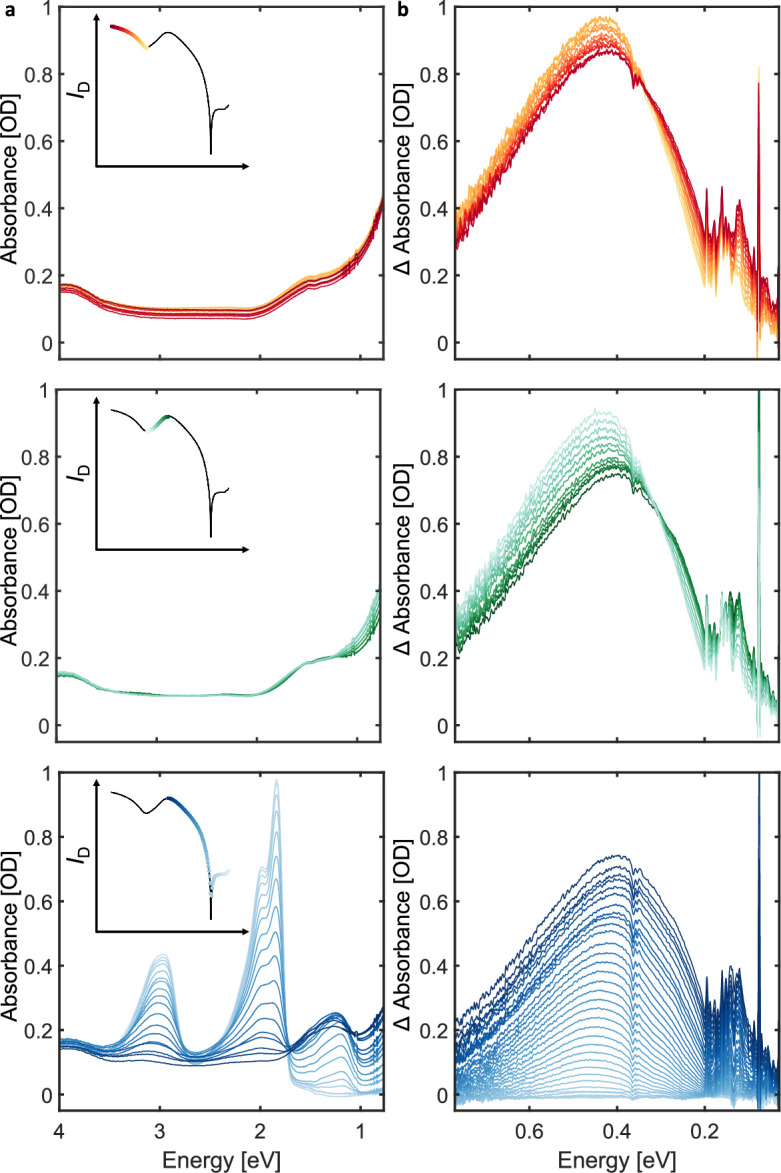
In-situ optical spectroscopy of ion gated IDT-BT. In-operando (**a**) UV-Vis-NIR and (**b**) FT-IR measurements of IDT-BT OECTs. UV-Vis-NIR spectra show absolute absorbance, FT-IR spectra show absorbance change from the off state (V_IG_ = 0 V). OD, optical density. In Regime I, the bleaching of the *π* − π^*^ band at 2 eV due to removal of charge from the HOMO band reaches completion exactly at the conductivity peak, consistent with the removal of one electron per repeat unit. The IR absorption similarly is maximized at the end of Regime I. In Regimes II and III, an isosbestic point appears in the IR spectrum near 0.3 eV, indicative of a one-to-one interconversion between polaronic species with fixed total concentration, that is the conversion of singly charged states into multiply charged states.

**Extended Data Fig. 5:**
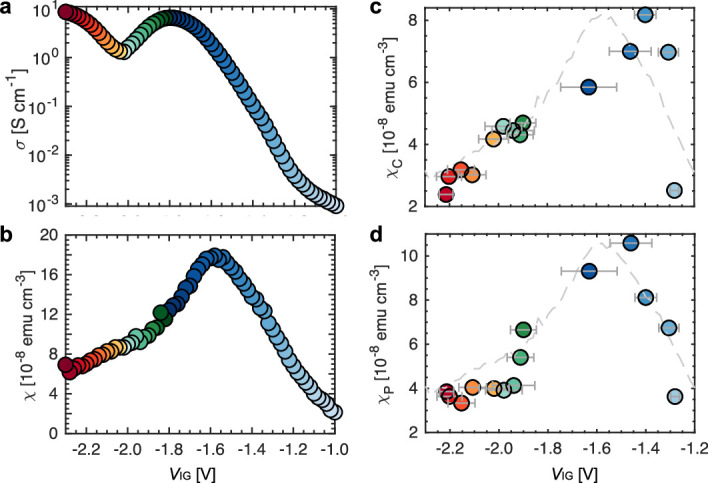
Electron spin resonance (ESR) spectroscopy of ion gated IDT-BT. (**a-b**) ESR measurements of an IDT-BT OECT at 290 K: (**a**) device transfer curve and (**b**) total magnetic susceptibility. The decrease in total susceptibility at *V*_IG_ < −1.6 V is attributed to the onset of spin pairing; this reduction slows down beyond the valley in the conductivity, which could signal the onset of Regime III in some parts of the heterogeneous microstructure. (**c-d**) Paramagnetic susceptibility components extracted from temperature-dependent measurements: (**c**) Curie component of susceptibility and (**d**) Pauli component of susceptibility. Dashed lines are a guide for the eye showing the scaled total magnetic susceptibility trend. Gate voltages in (**c,d**) have been corrected for threshold voltage shifts during temperature sweeps; the corrected gate voltages are presented as the best fit values ± mean absolute error, where the best fit values are obtained by minimising their residuals. The Curie component dominates at low doping levels, whereas the Pauli component is dominant around the conductivity peak. The Curie component persists up to the most negative *V*_IG_, which indicates that complete spin-pairing of these Curie spins is hindered, perhaps by strong on-site Coulombic repulsions or heterogeneity in the microstructure. The Pauli component drops considerably towards the conductivity valley, consistent with the interpretation of the Pauli susceptibility being a measure of the DOS at the Fermi level experienced by the metallic spins. See Supplementary Note 3.8 for an extended discussion of the physical interpretation and the gate voltage correction routine.

X-ray photoemission spectroscopy and gate current charging analysis show that the peak of conductivity corresponds to one dopant per polymer repeat unit (*n* = 1), that is, the HOMO-derived band is half-filled, whereas in Regime III the doping concentration exceeds *n* = 2 (Extended Data Fig. 1 and Supplementary Notes 3.4 and 3.5). Our observations are consistent with a simple band-filling interpretation, which assumes that in Regime III, holes are induced in deeper-lying states derived from HOMO−1: in Regimes I and III, *S* is determined by holes at the top of the HOMO- and HOMO−1-derived bands, respectively, whereas in Regime II, electrons remaining at the bottom of the HOMO-derived band determine the sign of *S*. At the transition between Regimes II and III, *S* is governed by the competition between electrons in the HOMO and holes in the HOMO−1-derived bands—causing *S* to be near zero in the valley but to take on a large value on either side. Complementary measurements presented in Extended Data Figs. 2–5 are fully consistent with this band-filling interpretation. We considered alternative mechanisms for the non-monotonic OECT and Seebeck behaviour, including the opening of a Coulomb gap around the Fermi level due to electron–electron repulsion^4^, changes in structural ordering upon doping and a previously reported mechanism that involves the coexistence of crystalline and amorphous domains;^23^ however, these did not provide a consistent explanation for our observations (Supplementary Notes 3 and 4).

**Extended Data Fig. 6:**
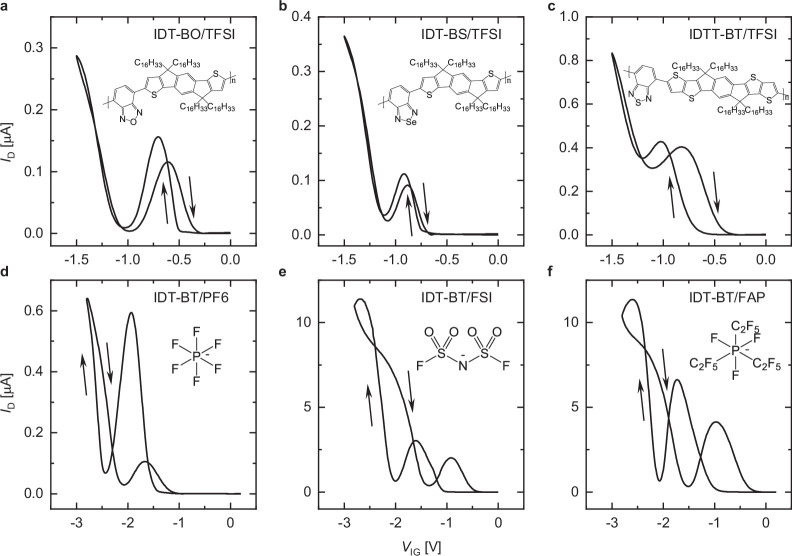
Other polymer/ion combinations allowing access to Regime III. (**a-c**) OECT transfer curves of other polymers gated with TFSI: (**a**) indacenodithiophene-co-benzooxadiazole (IDT-BO), (**b**) indacenodithiophene-co-benzoselenadiazole (IDT-BS), and (**c**) indacenodithieno[3,2-*b*]thiophene-co-benzothiadiazole (IDTT-BT). (**d-f**) OECT transfer curves of IDT-BT gated with different ions: (**d**) hexafluorophosphate (PF_6_), (**e**) bis(fluorosulfonyl)imide (FSI), and (**f**) tris(pentafluoroethyl) trifluorophosphate (FAP). Insets are the molecular structures of the corresponding polymers or ions. All transfer curves were taken at room temperature with *V*_D_ = −0.1 V. These results demonstrate that Regime III operation is not unique to IDT-BT gated with TFSI. The smallest of these anions, PF_6_, shows a decreased electrochemical stability, leading to a strong reduction in peak current on the reverse scan.

**Extended Data Fig. 7:**
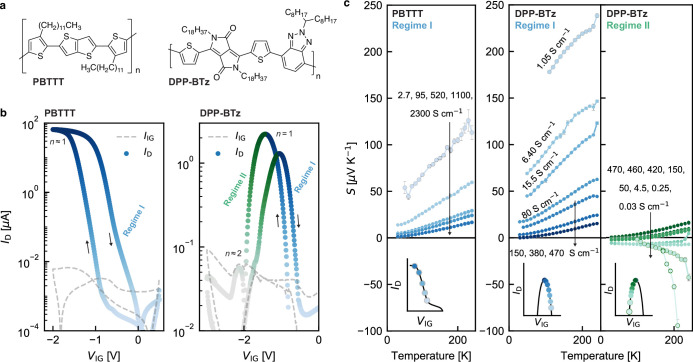
Band filling in PBTTT and DPP-BTz. (**a**) Molecular structures of PBTTT and DPP-BTz. (**b**) OECT transfer curves of typical PBTTT and DPP-BTz devices gated with BMP TFSI (room temperature, *V*_D_ = −0.1 V). In PBTTT a monotonic increase of conductivity with increasingly negative gate voltages is seen, suggesting that the device remains in Regime I throughout. Conversely in DPP-BTz a decrease of conductivity at high gate voltages is seen, suggesting a crossover to Regime II. (**c**) Temperature dependence of the Seebeck coefficient of PBTTT and DPP-BTz. Data are presented as the mean Seebeck coefficients ± standard error of the mean, originating from fitting uncertainties of the on-chip thermometer calibration and the thermovoltage versus temperature difference plots. The absence of n-type transition in PBTTT confirms that PBTTT remains in Regime I even at the highest doping levels (*n* ≈ 1) attainable in the present study. In DPP-BTz, a transition to n-type transport is observed beyond the conductivity peak, confirming the accessibility of Regime II in DPP-BTz.

Regime III operation was also observed in several other polymers (Extended Data Fig. 6a–c) and in IDT-BT OECTs gated with different ionic liquid anions (Extended Data Fig. 6d–f). We also observe consistent band-filling behaviour in PBTTT and DPP-BTz (Extended Data Fig. 7 and Supplementary Note 3.1), but with a smaller range of accessible doping levels: all three polymers exhibit a positive *S* in Regime I and approach a conductivity maximum near half-band filling (*n* = 1). In PBTTT, Regime II is not accessible. In DPP-BTz, we observe inversion to a negative *S* in Regime II, as in IDT-BT; however, a fully insulating state is reached, that is, Regime III is inaccessible.

**Extended Data Fig. 8:**
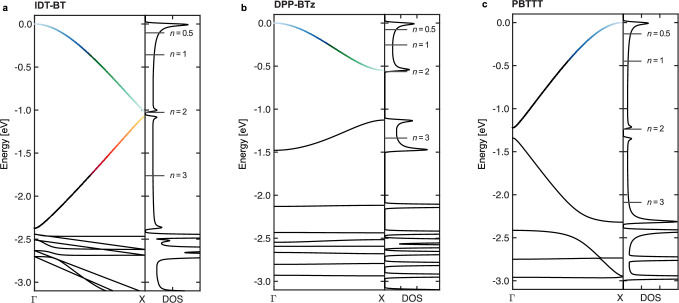
DFT DOS and band structure calculations. DFT band structure (left) and DOS (right) of (**a**) IDT-BT, (**b**) DPP-BTz, and (**c**) PBTTT along the polymer backbone direction Γ − X. Doping level *n* indicates the number of ions per repeat unit. In comparison to IDT-BT, DPP-BTz displays a relatively narrow HOMO (bandwidth = 0.55 eV), but a deeper HOMO-1 derived band edge which may contribute to the inaccessibility of Regime III. PBTTT shows a considerably broader HOMO (bandwidth = 1.22 eV) compared to DPP-BTz and IDT-BT, which likewise may contribute to the inaccessability of Regimes II and III (see further discussion in Supplementary Note 3). We stress that although the relative differences between the band structure and DOS derived from these calculations are instructive, they neglect important factors such as electron-ion interactions and microstructural disorder. Therefore, the DOS obtained here is not expected to be consistent to our experimental band filling data (Fig. 1 and Extended Data Fig. 7). GIWAXS data (Supplementary Note 4) suggest that structural changes upon doping, which are not captured in these DFT calculations, may be more relevant in explaining the inaccessability of Regime II in PBTTT.

Operando grazing-incidence wide-angle X-ray scattering (GIWAXS) (Supplementary Note 4) shows that microstructural changes upon ion incorporation play a major role in determining accessible doping ranges. In IDT-BT, doping generates only a minor reversible increase in crystallinity that is uncorrelated with conductivity. PBTTT, by contrast, forms a highly ordered co-crystal with one ion per monomer, the stability of which prevents the incorporation of further ions (Supplementary Note 4.3). DPP-BTz shows a partially irreversible reduction in crystallinity at high doping levels (Supplementary Note 4.2) that limits operation at very high doping levels. The absence of Regime III in DPP-BTz can also be rationalized via density functional theory (DFT) calculations performed on single, neutral polymer chains, which show that the second occupied band is expected to be energetically more accessible in IDT-BT than in DPP-BTz (Extended Data Fig. 8 and Supplementary Note 5). We warn, however, that these calculations neglect Coulomb interactions between charge carriers and with the counterions that are important in highly doped systems^24,25^. A more realistic electronic structure model, fully accounting for Coulomb interactions, will be provided below.

## Probing non-equilibrium states via double gating

**Fig. 2:**
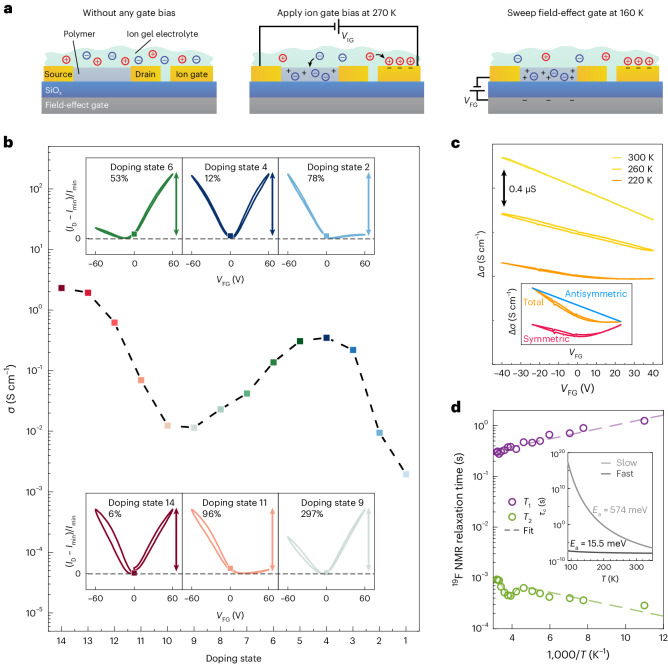
Non-equilibrium transport signatures in IDT-BT double-gated transistors. **a**, Schematic of the double-gated transistor experiment. **b**, Electrical conductivity at *V*_FG_ = 0 V of various doping states at 160 K. Insets show field-effect transfer curves for six representative doping states at 160 K. As the film conductivity changes by orders of magnitude as the doping level is increased, the drain current is normalized as (*I*_D_(*V*_FG_) − *I*_min_)/*I*_min_, where *I*_min_ is the minimum current in the field-effect transfer curve of the corresponding doping state. For each inset the maximum field-effect modulation is indicated as a percentage value. At the conductivity peak, the current decreases when changing *V*_IG_ but increases when changing *V*_FG_. **c**, Field-effect transfer curves for an ex-situ-doped IDT-BT device between 300 and 220 K, exhibiting non-linear behaviour at low temperatures. The transfer curves are vertically shifted for clarity. The magnitude of the arrow represents 0.4 μS. The inset shows a schematic of the decomposition of the 220 K transfer curve into a linear antisymmetric component and a symmetric component. **d**, ^19^F nuclear spin–lattice relaxation time (*T*_1_) and spin–spin relaxation time (*T*_2_) versus temperature (*T*) obtained from ^19^F NMR saturation recovery and spin-echo delay experiments of IDT-BT doped with TFSI. The inset shows the extracted correlation times (*τ*_c_) from a two-component fit described in Supplementary Note 7.

Whereas electrochemical doping enables *E*_F_ to be shifted deep into the HOMO/HOMO−1 band, the potential formation of a Coulomb gap at these ultrahigh carrier concentrations cannot be inferred unambiguously from OECT characteristics as the temperature scaling of conductivity varies with the doping level (Supplementary Note 3.2). To probe DOS changes more directly, we introduce a new double-gating technique that incorporates both a field-effect gate and an ion gate (Fig. 2a). First, we set the bulk doping level by applying *V*_IG_ and cooling the device with *V*_IG_ held on. Below the glass transition temperature of the electrolyte (~200 K), *V*_IG_ can be removed and the field-effect gate voltage (*V*_FG_) can be swept to record field-effect transfer curves without affecting the film doping level. This experiment probes the transport of additional electronic carriers injected into the DOS at a bulk doping state defined by *V*_IG_, without being charge compensated by extra counterions. By choosing a positive or negative *V*_FG_, we can inject either additional electrons or holes.

An important practical requirement for such double-gating experiments is minimizing the polymer thickness. In undoped FETs, only a region of ~1 nm thick near the polymer/SiO_2_ interface is affected by *V*_FG_; in our highly doped devices, the expected doping-induced increase in dielectric constant^22^ should further limit the field effect to the first polymer monolayer near the interface. Even for the thinnest practically achievable polymer films (~10 nm), *V*_FG_ only injects the equivalent of a bulk charge concentration of ~10^19^ cm^−3^, which is much smaller than the *V*_IG_-induced bulk doping levels (~10^21^ cm^−3^). The expected, *V*_FG_-induced current modulation is therefore at most a few per cent.

**Extended Data Fig. 9:**
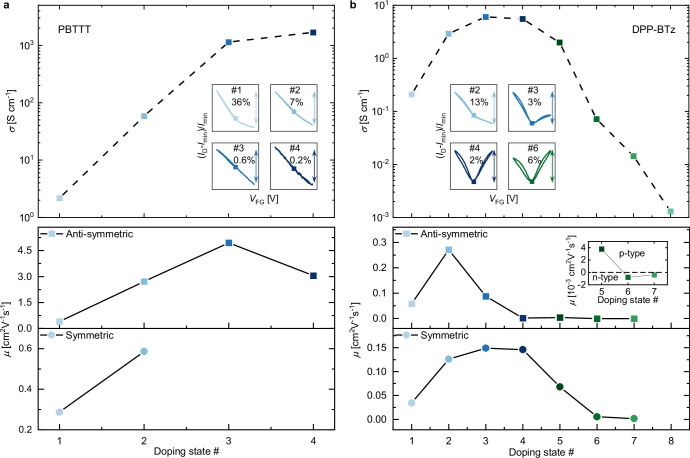
Non-equilibrium transport signatures in double-gated PBTTT and DPP-BTz measurements. Top panels show the electrical conductivities of (**a**) PBTTT and (**b**) DPP-BTz, at *V*_FG_ = 0 V for different doping states at 160 K, with insets showing field-effect transfer curves for several representative doping states and the percentage of maximum modulation in *I*_D_. Bottom panels show the symmetric and anti-symmetric carrier mobilities, as defined in Supplementary Note 6.1. For DPP-BTz, the inset in the anti-symmetric mobility plot is a zoomed-in view for doping states 5, 6, and 7. In DPP-BTz we observe an ambipolar behaviour similar to IDT-BT. In PBTTT, the non-equilibrium transport manifests itself as a deviation from linearity in the field-effect transfer curves, which is evident in doping states 1 and 2 at 160 K, but becomes observable and more pronounced in all doping states at lower temperatures (Supplementary Note 6.4). These results demonstrate that our observation of non-equilibrium transport is general and extends to higher mobility polymers with room temperature conductivities of > 100 − 1, 000 S cm^−1^.

Figure 2b shows six selected field-effect transfer curves (insets) measured at different doping states along the IDT-BT OECT transfer curve (main panel; full dataset in Supplementary Fig. 26). The experimental data disclose several surprising observations. First, the field-effect modulation is much larger than expected, varying from 12% near the peak to 297% near the valley. Second, we had expected the field-effect response to follow the OECT ion gate transfer characteristics—to be unipolar p-type in Regimes I and III and n-type in Regime II. At the peak (doping state 4), we had expected a current reduction for both positive and negative *V*_FG_. Instead, we observe a current increase in both directions. At most of the other doping states, a similar ‘graphene like’ ambipolar current increase is observed, although the current rise for negative *V*_FG_ is stronger than for positive *V*_FG_ in Regimes I and III and weaker in Regime II. Note that at the highest doping level (state 14), the field-effect transfer characteristics resemble those at the first peak (state 4), suggesting that we may be approaching half-filling of the HOMO−1 band. This behaviour was found to be general and was also observed in DPP-BTz and PBTTT (Extended Data Fig. 9 and Supplementary Note 6).

To analyse the non-linear transfer curves, we decompose them into a linear component that is antisymmetric with respect to *V*_FG_ = 0 (that is, an odd function: *I*_D_( − *V*_FG_) − *I*_D_(*V*_FG_ = 0) = −(*I*_D_(*V*_FG_) − *I*_D_(*V*_FG_ = 0))) and a non-linear component that is symmetric with respect to *V*_FG_ = 0 (that is, an even function: *I*_D_( − *V*_FG_) − *I*_D_(*V*_FG_ = 0) = *I*_D_(*V*_FG_) − *I*_D_(*V*_FG_ = 0)) (inset of Fig. 2c). The linear, antisymmetric component follows the behaviour expected from the ion gate transfer characteristics and the Seebeck coefficient, and is believed to be determined by the slope of the DOS at *E*_F_: in Regimes I and III, the DOS rises as *E*_F_ moves towards the centre of the HOMO/HOMO−1-derived band, resulting in a p-type linear component; and in Regime II, the DOS falls as *E*_F_ is pushed beyond the centre of the HOMO-derived band, resulting in an n-type linear component. At half-filling of the HOMO-derived band (doping state 4) the slope of the DOS at *E*_F_ vanishes, and the linear component disappears.

**Extended Data Fig. 10:**
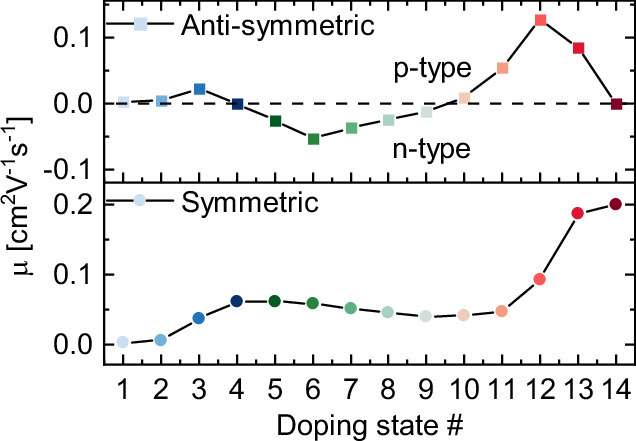
Field-effect mobilities extracted from the symmetric and anti-symmetric components of the field-effect transfer characteristics of IDT-BT. Measurements were performed at 160 K. The magnitude of the anti-symmetric field-effect mobilities are generally lower than the symmetric mobilities, for example 0.02 cm^2^ V^−1^ s^−1^ (anti-symmetric) vs. 0.04 cm^2^ V^−1^ s^−1^ (symmetric) for doping state 3, indicative of enhanced charge transport in the field-effect induced non-equilibrium states. Mobilities are extracted from the complete dataset shown in Fig. 2(b) and Supplementary Fig. 26.

The symmetric component, on the other hand, will be shown below to probe a Coulomb gap at *E*_F_ that is superimposed on the DOS. We can understand not only the ambipolar nature but also the unexpectedly strong field-effect response if we assume that the additional, *V*_FG_-induced carriers are able to access states at the edges of the Coulomb gap that have a higher DOS and/or higher degree of charge delocalization than states at the centre of the gap. This will allow the additional charge carriers, in essence, to move more quickly than the carriers induced by doping (Extended Data Fig. 10).

The observed ambipolar signatures are reminiscent of low-temperature (<4 K) field-effect gating experiments on other disordered metals, such as granular or oxide metals^9,26,27^. One explanation proposed for these is slow electron relaxation in the presence of a Coulomb gap caused by electron–electron interactions^28^. However, there are important differences—here, we observe these signatures at much higher temperatures and more stably, even when measuring very slowly or applying a field-effect bias stress for up to one day (Supplementary Note 6); we do not observe, for example, so-called double-dip signatures under similar measurement conditions^26^. This makes it unlikely that glassy electron relaxation, that is, the inability of the additional carriers to equilibrate with the doping-induced electronic carriers on the measurement timescale, is responsible in our system, as this would be expected to be fast at such high temperatures—that is, a soft Coulomb gap will remain tied to the shifting Fermi level when *V*_FG_ is changed^29,30^.

To better understand the origin of the non-linear symmetric component, we investigated its temperature dependence. Non-linear field-effect transfer characteristics were observed over a wide temperature range below 190 K (Supplementary Note 6.1). Measurements at higher temperatures are not possible on OECTs (as ions move in and out of the film in response to *V*_FG_), so films were doped ex situ via ion-exchange doping or by washing off the ionic liquid after electrochemical doping. The change in conductivity Δ*σ* = *σ* − *σ*_0_ (where *σ*_0_ = *σ*(*V*_FG_ = 0)) is plotted versus *V*_FG_ at different temperatures in Fig. 2c (raw data in Supplementary Fig. 28). Strikingly, at 300 K, the transfer curve of this mildly doped IDT-BT film in Regime I is unipolar p-type and perfectly linear. The non-linearity reproducibly appears only below ~240 K, which is close to the melting point of the ionic liquid (255 K)^31^ and the *β-*glass transition temperature of IDT-BT (249 K)^32^ associated with side-chain motion. This suggests that the appearance of the non-linear component is linked to the relaxation dynamics of the dopant counterions rather than the electrons.

To study the ion dynamics we measured the ^19^F nuclear spin–lattice and spin–spin relaxation times *T*_1_ and *T*_2_, respectively, of the TFSI ions in an IDT-BT film (Fig. 2d) using NMR. Nuclear spin relaxation is driven by fluctuating fields associated with molecular motions, enabling us to determine the correlation times of these motions. *T*_1_ decreases monotonically with increasing temperature, whereas *T*_2_ exhibits three different temperature regimes. Above 255 K and below 215 K, *T*_2_ increases with increasing temperature, indicating motional narrowing regimes corresponding to two different types of molecular motion that are fast enough to average effectively over local magnetic environments. The intermediate regime (215–255 K) reflects a slowing of the high-temperature motion, which causes motional narrowing to become ineffective. A fit to Redfield theory (Supplementary Note 7) yields correlation times for both motions. The faster, low-temperature-dominating motion with an activation energy (*E*_a_) of ~16 meV is probably associated with an intra-ionic motion, such as functional group rotations. The slower, high-temperature-dominating motion has a much higher activation energy (574 meV), which is typical for translational ion motion in polymers^33,34^, and slows by more than five orders of magnitude in the temperature range where the non-linearity appears.

This suggests that the unusual field-effect response is caused by the slowing of translational ion motion as the polymer approaches its glass transition. At room temperature, ionic motion is fast enough for ions to adjust to changes in carrier concentration and find their lowest energy equilibrium configuration as *V*_FG_ is swept. At 260 K, the timescale of ionic relaxation becomes comparable to the *V*_FG_ sweep rate, which manifests itself as hysteresis in the transfer curves (Fig. 2c). Below 240 K the ions can no longer respond to *V*_FG_-induced changes in carrier concentration and remain frozen in configurations they adopted before cooling. If instead a non-zero *V*_FG_ is applied to perturb this configuration during cooling, the system ‘memorizes’ this high-temperature equilibrium configuration, and the minimum of the ambipolar transfer characteristics at low temperature shifts to the *V*_FG_ value applied during cooling (Supplementary Fig. 30).

## Simulating non-equilibrium transport

**Fig. 3:**
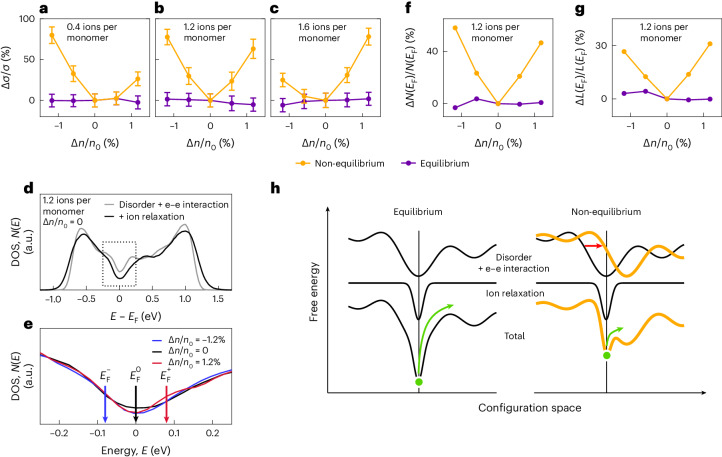
Theoretical modelling of non-equilibrium transport. **a**–**c**, Transfer curves calculated in and out of equilibrium for systems at electrochemical doping levels of 0.4 (**a**), 1.2 (**b**) and 1.6 ions per monomer (**c**). The effect of the field-effect gate bias is modelled as a relative change in the charge density, Δ*n*/*n*_0_, where *n*_0_ is the number of electrons in a full band. Data are presented as the mean of 400 realizations ± standard error of the mean. **d**, DOS in the absence of field-effect gating, with and without ionic relaxation at a doping level of 1.2 ions per monomer. e-e interaction, electron–electron interaction. **e**, DOS around *E*_F_ for positive and negative gate bias under out-of-equilibrium conditions. The arrows mark the Fermi level for different Δ*n*/*n*_0_. **f**,**g**, Relative variation of the DOS at *E*_F_ (**f**) and relative variation of the participation ratio *L*(*E*_F_) (**g**), measuring the degree of delocalization of the carriers. **h**, Schematic of the free energy landscape in equilibrium (left) and out of equilibrium (right). Equilibrium cases refer to mobile ion and/or zero-gate bias conditions, while non-equilibrium refers to the case of frozen ions with non-zero gate bias. The contributions of disorder and e-e interaction (top), ionic relaxation (middle) and their sum (bottom) are shown schematically. In the non-equilibrium condition the gate bias alters the electronic energy landscape (red arrow), but the ionic relaxation contribution remains unchanged. This leads to a smaller energy barrier (green arrow) to escape from the energy minimum.

Theoretical simulations were performed using a two-dimensional Hubbard-type model that we recently applied to study the interplay between Coulomb interactions and disorder^19^. The model has been generalized to enable the tuning of carrier concentrations with and without introducing charge-neutralizing counterions, thus mimicking electrochemical doping and the application of a field-effect bias, respectively. The simulations consider either equilibrium conditions, where ion positions are relaxed upon applying a gate bias, or non-equilibrium conditions, where ion positions are frozen (Supplementary Note 8). The field-effect transfer curves simulated under frozen-ion, non-equilibrium conditions are in good agreement with the experiments. For doping levels around half-filling (Fig. 3b, 1.2 ions per monomer) the conductivity, presented as its relative change Δ*σ*/*σ*, is enhanced symmetrically when adding or removing additional carriers, Δ*n*. When moving away from half-filling (Fig. 3a,c, for 0.4 and 1.6 ions per monomer, respectively), asymmetric curves are observed, agreeing with our experiments. This ambipolarity is absent in equilibrium simulations (Fig. 3a–c).

The ambipolarity is rooted in the formation of a soft Coulomb gap in the DOS at *E*_F_, which originates from the interactions between localized electrons in a disordered medium^2,19^. However, crucially, ionic relaxation deepens the Coulomb gap by enhancing disorder and charge localization (Fig. 3d)^35^. Under non-equilibrium conditions, *E*_F_ is shifted with respect to the centre of the gap upon field-effect gating (Δ*n* ≠ 0; Fig. 3e) because the dominant contribution to the pseudogap from the ionic reaction field is fixed, that is, frozen at its original, zero-bias position. This shift increases the number of states available for transport (Fig. 3f) as well as the delocalization of these states (Fig. 3g), both effects co-operating to yield an ambipolar conductivity enhancement. Under equilibrium conditions, on the other hand, *E*_F_ remains pinned at the DOS minimum (Supplementary Fig. 42), resulting in flat transfer curves.

To demonstrate the proposed mechanism Fig. 3h illustrates the free energy landscape in configuration space as determined by disorder and electron–electron interactions. The ion relaxation at zero gate bias deepens the potential wells and enhances state localization. The application of a gate bias with frozen ions alters the electronic contribution to the energy landscape but leaves the ionic contribution unchanged. This lowers the activation barriers (green arrows) for carriers to move, rationalizing the ambipolar conductivity increase.

## Spectroscopic evidence for enhanced delocalization

To validate our theoretical prediction of enhanced state delocalization at *E*_F_ upon field-effect gating (Fig. 3g), experimentally we used charge modulation spectroscopy (CMS), which measures the differential optical absorption (ΔAbs) as *V*_FG_ is modulated. This provides direct spectroscopic characterization of the additional carriers;^36^ a redshifted infrared polaron-induced absorption (0.1–0.5 eV) would indicate increased polaron delocalization^37^.

**Fig. 4:**
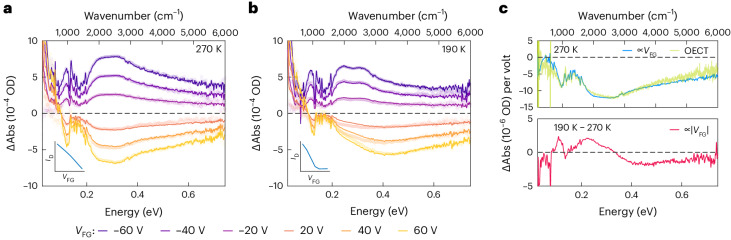
Spectroscopic evidence for enhanced delocalization of non-equilibrium states. **a**,**b**, CMS spectra of an IDT-BT:TFSI film doped to Regime I at 270 K (**a**) and 190 K (**b**). The bottom left insets show the transfer curves at each temperature. The symbols show the experimental data, and the solid lines are fits from the decomposed symmetric and antisymmetric component spectra as described in the text. OD, optical density. **c**, Antisymmetric (top) and symmetric (bottom) component spectra used to fit the data. The antisymmetric component (blue line, 270 K data shown) matches closely the OECT moving difference spectrum (green line) calculated from the data in Extended Data Fig. 4. The symmetric component (red line, difference between 190 K and 270 K data shown) is nearly independent of the doping level and reflects an increase in carrier delocalization upon field-effect gating below the ionic glass transition temperature.

At 270 K, (Fig. 4a) a negative *V*_FG_ leads to increased polaronic absorption, whereas a positive *V*_FG_ produces a corresponding bleaching signal without spectral changes (apart from an absorption tail below 0.1 eV from the CMS response of the silicon substrate). At 190 K (Fig. 4b) the CMS spectrum becomes more complex; the induced absorption and bleaching spectra at negative and positive *V*_FG_ values, respectively, no longer have the same shape. To interpret these spectra, we mirror the analysis of the electrical characteristics and decompose the measured absorption *A*(*ω*) into component spectra *c*(*ω*) that are symmetric and antisymmetric with respect to *V*_FG_, that is *A*(*ω*) = *c*_a_(*ω*)*V*_FG_ + *c*_s_(*ω*)∣*V*_FG_∣. A least-squares fit at each frequency provides a good match with the experimental data (Supplementary Note 9).

At 270 K, the antisymmetric component dominates (blue line in Fig. 4c) and matches closely the corresponding moving difference spectrum obtained from the OECT data in Extended Data Fig. 4 (green line in Fig. 4c). This indicates that above the transition temperature the polarons induced by ion or field-effect gating are indistinguishable spectroscopically and have the same degree of polaron delocalization. This is consistent with our previous work, showing that at such high doping levels the ions act as a uniform background charge density^19^.

Below the transition temperature (190 K data), whereas the antisymmetric component remains qualitatively unchanged (Supplementary Note 9), a pronounced symmetric component emerges. The response of the silicon appears in the symmetric component as well, but this can be removed by subtracting the 270 K symmetric component. The resulting subtracted symmetric component (Fig. 4c, red line) is remarkably consistent in spectral shape and intensity across a range of doping levels (Supplementary Note 9) and shows a broad negative, bleaching feature at high energy and a positive, induced absorption at low energy, that integrate to near zero absorption. This signal therefore does not reflect a change in carrier density but instead indicates that the polaron bands of both additional electron and hole carriers are shifted to lower energy. This shift provides clear validation that the field-induced, non-equilibrium carriers are indeed more delocalized than the ion-gate-induced equilibrium carriers, in good agreement with our theoretical model and electrical characteristics.

## Enhanced transport coefficients

**Fig. 5:**
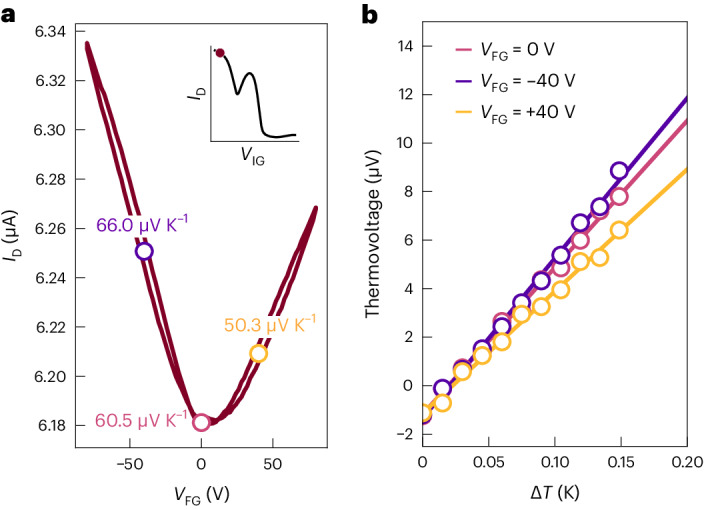
Enhancement of the Seebeck coefficient due to the frozen Coulomb gap. **a**,**b**, Field-effect transfer curve (*V*_D_ = −0.1 V) of a highly doped IDT-BT device measured at 190 K (in Regime III, see inset) (**a**), where changes in the magnitude of the Seebeck coefficient across the gap are indicated on the transfer curve, as inferred from thermovoltage measurements at various field-effect gate voltages (**b**).

The ability to shift *E*_F_ away from the centre of the Coulomb gap provides a route to enhancing the Seebeck coefficient. Within the Boltzmann transport framework, *S* is proportional to the logarithmic slope of the energy-dependent conductivity function, which is smallest at the bottom of the gap but rises as more delocalized states with a larger DOS become accessible at the edges of the gap. As a proof of principle we attempted to detect changes in the thermopower upon the field-effect gating of an IDT-BT double-gated device (Fig. 5) operating in a highly conducting Regime III state, similar to doping state 14. Indeed, when the soft gap is frozen at 190 K, a 10% larger Seebeck coefficient of 66.0 μV K^−1^ is recorded at *V*_FG_ = −40 V, compared with the ungated value of 60.5 μV K^−1^. This is remarkable, as field-effect gating only affects carriers near the dielectric interface. From a parallel conduction model, we estimate that a thermopower of up to an order of magnitude larger can be achieved if *E*_F_ can be similarly shifted in the bulk. For *V*_FG_ = +40 V, *S* is reduced compared with the ungated state, due to competition between the p-type bulk response and the expected n-type field-effect response (Supplementary Note 10). These observations validate our proposed model and demonstrate that thermoelectric transport coefficients *σ* and *S* can both be substantially enhanced in the non-equilibrium regime.

Our findings open up new strategies to considerably enhance the performance of conducting polymers for applications from thermoelectrics to bioelectronics. (1) In Regime III, markedly higher conductivities and Seebeck coefficients are achievable, possibly because polarons in the HOMO−1-derived band experience a higher dielectric constant/more efficient screening of Coulomb interactions^22^. The hitherto neglected HOMO−1-derived band should therefore be considered when designing high-performance polymers. (2) Although non-equilibrium transport phenomena in disordered metals have been confined to very low temperatures^8,9^, our new mechanism involves freezing a Coulomb gap in the electronic DOS by controlling the relaxation of dopant counterions that interact Coulombically with the electrons. This mechanism enables non-equilibrium phenomena much closer to room temperature. For practical applications, freezing of the gap above room temperature should become possible using polymers and ionic liquids/salts with higher glass transition/melting temperatures. Methods to induce highly delocalized non-equilibrium carriers, not just at interfaces but also in the bulk, are needed; this may be achievable through a two-step doping protocol, with ions introduced during the first step unable to relax during the second step. During the review process of this work we have become aware that potentially related non-equilibrium states in the system of polarons/bipolarons and counterions can be observed in poly(3,4-ethylenedioxythiophene)/poly(styrene sulfonate) (PEDOT/PSS) via X-ray photon correlation spectroscopy^38^. Our results suggest polymers could become powerful model systems for fundamental studies of non-equilibrium transport phenomena in disordered metals.

## Methods

### Sample preparation

Silicon/SiO_*x*_ substrates were used for the double-gated devices, and all the other devices were fabricated on Corning Eagle XG glass substrates. Electrodes were fabricated using standard photolithography, metal deposition and a lift-off process. Conjugated polymer films were spin coated using glass pipettes to minimize contamination^39^. All spin coating and annealing was performed in a nitrogen glovebox (<1 ppm H_2_O, O_2_) equipped with a molecular sieve solvent trap. For ex-situ-doped samples, the polymer films were doped using either ion-exchange doping or electrochemical doping in a nitrogen glovebox using a PalmSens4 potentiostat. For OECTs and double-gated devices, the polymer channels were patterned using photolithography, and the ion gel layers were spin coated to cover both the ion gate electrodes and the polymer channels. Further details (for example, spin-coating parameters for different polymers and doping procedures) can be found in Supplementary Note 1.1.

### Electrical, thermoelectric and electrochemical measurements

Room-temperature OECT measurements were carried out in a nitrogen glovebox. Low-temperature OECT and double-gated measurements were performed using a cryogenic probe station (a Desert Cryogenics probe station or a LakeShore Cryotronics CRX-4K closed-cycle cryogenic probe station). Seebeck measurements were performed in a LakeShore Cryotronics CRX-4K closed-cycle cryogenic probe station. For these measurements, a Keithley 2612B source measure unit and an Agilent 4155B semiconductor parameter analyser were used for the source voltage and to measure the current, and the thermovoltage signals were measured using a Keithley 2182A nanovoltmeter or a Keithley 6430 subfemtoamp source measure unit with a remote preamplifier. Cyclic voltammetry measurements were performed with a PalmSens4 potentiostat, a Pt counter electrode and an Ag quasi-reference electrode. Polymer films served as the working electrodes and ionic liquids as the electrolyte. To estimate the conductivity at different oxidation potentials, we used the chronoamperometry and linear sweep voltammetry functions of the potentiostat: a constant voltage was applied to the polymer film for 30 s, and then a current–voltage scan was quickly carried out between two electrodes in contact with the polymer film. Further details (for example, device geometries) can be found in Supplementary Note 1.2.

### Magnetic resonance spectroscopy

Continuous-wave electron spin resonance (ESR) measurements were carried out using a Bruker E500 spectrometer with an X-band microwave source and a Bruker ER 4122SHQE cavity. All measurements were carried out using an Oxford Instruments ESR900 helium cryostat. More details on our ESR set-up and analysis routines can be found in ref. ^40^. ESR studies at various doping levels were performed on OECTs, enabling in situ adjustment of the doping level.

NMR measurements were carried out using a Bruker 400 MHz dynamic nuclear polarization spectrometer (~9.4 T). A 3.2 mm LT MAS (low-temperature magic-angle spinning) probe was used, which could be tuned to ^1^H or ^19^F. Samples were spun at a MAS frequency of 11 kHz at all temperatures. The spin–lattice (*T*_1_) relaxation time values were obtained from saturation recovery experiments with echo detection (such a recoupling detection pulse can considerably attenuate any probe background signals). The spin–spin (*T*_2_) relaxation time values were obtained from spin-echo decay experiments. Spectral fitting and lifetime extractions were performed using the ssNAKE program^41^. Rotors were packed with the sample of interest (~5.35 mg of doped IDT-BT) together with the KBr reference in an argon glovebox. The sample temperature could be determined accurately from the well-established temperature dependence of the *T*_1_ of ^79^Br nuclei^42^. Accounting for possible thermal drift over the prolonged duration of signal acquisition, each nominal temperature value has been taken as an average between two measurements at the beginning and at the end of the signal acquisition period.

### Photoemission spectroscopy

X-ray photoemission spectroscopy was carried out using a JEOL JPS-9030 set-up with a monochromatic Al Kα source (photon energy, *hν* = 1,486.6 eV). The core-level binding energy was calibrated by setting the C 1*s* peak of adventitious carbon to 284.8 eV. Ultraviolet photoemission spectroscopy was performed using monochromatized light from a He discharge (*hν* = 21.22 eV; HIS 13, Focus) and a SPECS PHOIBOS 100 hemispherical analyser. A bias of −10 V was applied between the sample and the analyser for measurement of the secondary electrons to eliminate the influence of the analyser workfunction. All photoemission spectroscopy measurements were carried out under an ultrahigh vacuum of ~10^−9^ mbar.

Photoemission samples were ex situ electrochemically doped and then rinsed with acetonitrile in a nitrogen glovebox to remove any residual ionic liquid and then transferred to the spectrometer using vacuum suitcases in a nitrogen atmosphere to eliminate air exposure at any point after doping. Photoemission measurements were performed within 30 min of the samples being prepared, to minimize the possibility of sample dedoping and/or degradation.

### Optical spectroscopy

Optical spectroscopy measurements were performed in Oxford Instruments Optistat CF-V cryostats under a high vacuum of ~10^−6^ mbar. For the ultraviolet-visible-near-infrared (UV-Vis-NIR) measurements, the cryostat was outfitted with crystalline quartz windows, whereas for the Fourier transform infrared (FTIR) measurements, thallium bromoiodide (KRS-5) windows were used. UV-Vis-NIR measurements were performed using an Agilent Cary 6000i UV-Vis-NIR spectrophotometer fitted with a tungsten halogen Vis-NIR lamp and a deuterium arc UV-lamp. The NIR and infrared portion of the spectrum (<1.1 eV) was smoothed using a Savitzky–Golay filter with a filter window of 50 points. The UV-Vis-NIR spectra shown are single scans collected with an integration time of 0.025 s per point. FTIR measurements were performed using a Bruker VERTEX 70v FTIR spectrometer with a Bruker DigiTect mid-infrared DLATGS (deuterated L-alanine-doped triglycine sulfate) detector. Measurements on OECTs used an interferometer scan rate of 1.6 kHz and a resolution of 4 cm^−1^. Data were measured under a constant gate current and are presented as a simple moving average of nine scans. For the infrared field-effect-gated CMS measurements, an interferometer scan rate of 5 kHz and a resolution of 16 cm^−1^ were used for all measurements. A total of 128 scans were collected per modulation step, and 64 modulation cycles of (OFF ON ON OFF) were collected per gate voltage (8,192 scans total). The Keithley 2612B source measure unit was used to electrically gate the devices during the spectral measurements.

### Grazing-incidence wide-angle X-ray scattering

GIWAXS measurements were performed at the I07 Surface and Interface Diffraction beamline of Diamond Light Source using a photon energy of *hν* = 12.5 keV; images were collected using a PILATUS 2M detector. We used a smaller incidence angle of 0.15° for the operando OECT measurements to maximize the signal, whereas a slightly larger angle of 0.2° was used for film samples. Electrical measurements of the device were carried out using the Keithley 2612B source measure unit. The device was enclosed in a helium atmosphere to minimize sample degradation and scattering from the ambient gaseous species. Beam damage was characterized by changes in the source–drain current after each X-ray exposure. To minimize this degradation, we reduced the X-ray flux as much as practically possible while maintaining a sufficient signal-to-noise ratio.

### Modelling

Periodic DFT simulations were performed to compute the band structure of pristine polymers using the all-electron atomic orbital formalism implemented in the CRYSTAL17 code^43^. Calculations were performed at the PBE0/def2-SVP level of theory^44^ for a single, periodic one-dimensional polymer chain with a 6 × 1 × 1 sampling of the Brillouin zone. Further details of the DFT simulations can be found in Supplementary Note 5.

The electronic structure of doped polymers was modelled with a two-dimensional extended Hubbard-type model with long-range Coulomb interactions, solved in the Hartree–Fock approximation^19^. Calculations were performed on large supercells of an anisotropic square lattice. Model parameters are representative of the IDT-BT polymer (see Supplementary Note 8 for details). The conductivity was calculated within the framework of transient localization theory^19,45^. Results were obtained as an average over 400 realizations of the energetic disorder. Error bars in the plots correspond to the standard error of the mean.

## Online content

Any methods, additional references, Nature Portfolio reporting summaries, source data, extended data, supplementary information, acknowledgements, peer review information; details of author contributions and competing interests; and statements of data and code availability are available at 10.1038/s41563-024-01953-6.

## Supplementary information

Supplementary Information Supplementary Notes 1–10, Figs. 1–57 and Equations (1)–(31).

## Data Availability

The data underpinning this study are available from the University of Cambridge data repository at 10.17863/CAM.109880. Additional materials are available from the corresponding authors upon reasonable request.

## References

[1] Dugdale, J. S. *The Electrical Properties of Disordered Metals* (Cambridge Univ. Press, 1995).

[2] Efros, A. & Skhlovskii, B. Coulomb gap and low temperature conductivity of disordered systems. *J. Phys. C* **8**, L49–L51 (1975).

[3] Koopmans, M. & Koster, L. J. A. Carrier–carrier Coulomb interactions reduce power factor in organic thermoelectrics. *Appl. Phys. Lett.* **119**, 143301 (2021).

[4] Xu, K. et al. On the origin of Seebeck coefficient inversion in highly doped conducting polymers. *Adv. Funct. Mater.* **32**, 2112276 (2022).

[5] Yoon, C., Reghu, M., Moses, D. & Heeger, A. Characteristic temperature dependence of resistivity in PPy(PF_6_) near the metal–insulator transition. *Synth. Met.* **69**, 369–370 (1995).

[6] Wang, S., Ha, M., Manno, M., Frisbie, C. & Leighton, C. Hopping transport and the Hall effect near the insulator–metal transition in electrochemically gated poly(3-hexylthiophene) transistors. *Nat. Commun.* **3**, 1210 (2012). 23169051 10.1038/ncomms2213

[7] Kaiser, A. B. Systematic conductivity behavior in conducting polymers: effects of heterogeneous disorder. *Adv. Mater.* **13**, 927–941 (2001).

[8] Pollak, M. & Ovadyahu, Z. Non-ergodic dynamics of an electron glass. *J. Phys. I* **7**, 1595–1602 (1997).

[9] Pollak, M. Electrons in Anderson–Mott insulators. *Eur. Phys. J. Spec. Top.* **227**, 2221–2240 (2019).

[10] Lee, J., Panzer, M. J., He, Y., Lodge, T. P. & Frisbie, C. D. Ion gel gated polymer thin-film transistors. *J. Am. Chem. Soc.* **129**, 4532–4533 (2007). 17381097 10.1021/ja070875e

[11] Tanaka, H. et al. Thermoelectric properties of a semicrystalline polymer doped beyond the insulator-to-metal transition by electrolyte gating. *Sci. Adv.* **6**, 8065–8079 (2020). 10.1126/sciadv.aay8065 PMC7021494 32110735

[12] Ito, M. et al. Band mobility exceeding 10 cm^2^ V^−1^ s^−1^ assessed by field-effect and chemical double doping in semicrystalline polymeric semiconductors. *Appl. Phys. Lett.* **119**, 13302 (2021).

[13] Ito, H., Mada, H., Watanabe, K., Tanaka, H. & Takenobu, T. Charge transport and thermoelectric conversion in solution-processed semicrystalline polymer films under electrochemical doping. *Commun. Phys.* **4**, 8 (2021).

[14] Cho, K. G., Adrahtas, D. Z., Lee, K. H. & Frisbie, C. D. Sub-band filling and hole transport in polythiophene-based electrolyte-gated transistors: effect of side-chain length and density. *Adv. Funct. Mater.* **33**, 2303700 (2023).

[15] Panzer, M. J. & Frisbie, C. D. Polymer electrolyte-gated organic field-effect transistors: low-voltage, high-current switches for organic electronics and testbeds for probing electrical transport at high charge carrier density. *J. Am. Chem. Soc.* **129**, 6599–6607 (2007). 17472381 10.1021/ja0708767

[16] Lee, K. H. et al. ‘Cut and stick’ rubbery ion gels as high capacitance gate dielectrics. *Adv. Mater.* **24**, 4457–4462 (2012). 10.1002/adma.201200950 22760996

[17] Venkateshvaran, D. et al. Approaching disorder-free transport in high-mobility conjugated polymers. *Nature* **515**, 384–388 (2014). 25383522 10.1038/nature13854

[18] Cendra, C. et al. Unraveling the unconventional order of a high-mobility indacenodithiophene–benzothiadiazole copolymer. *ACS Macro Lett.* **10**, 1306–1314 (2021). 35549036 10.1021/acsmacrolett.1c00547

[19] Jacobs, I. E. et al. Structural and dynamic disorder, not ionic trapping, controls charge transport in highly doped conducting polymers. *J. Am. Chem. Soc.* **144**, 3005–3019 (2022). 35157800 10.1021/jacs.1c10651 PMC8874922

[20] Schott, S. et al. Charge-transport anisotropy in a uniaxially aligned diketopyrrolopyrrole-based copolymer. *Adv. Mater.* **27**, 7356–7364 (2015). 26479721 10.1002/adma.201502437 PMC4768648

[21] Paulsen, B. D. & Frisbie, C. D. Dependence of conductivity on charge density and electrochemical potential in polymer semiconductors gated with ionic liquids. *J. Phys. Chem. C* **116**, 3132–3141 (2012).

[22] Comin, M., Fratini, S., Blase, X. & D’Avino, G. Doping-induced dielectric catastrophe prompts free-carrier release in organic semiconductors. *Adv. Mater.* **34**, 2105376 (2022). 10.1002/adma.202105376 34647372

[23] Liang, Z. et al. n-type charge transport in heavily p-doped polymers. *Nat. Mater.* **20**, 518–524 (2021). 33398117 10.1038/s41563-020-00859-3

[24] Winkler, S. et al. Probing the energy levels in hole-doped molecular semiconductors. *Mater. Horizons* **2**, 427–433 (2015).

[25] Png, R.-Q. et al. Madelung and Hubbard interactions in polaron band model of doped organic semiconductors. *Nat. Commun.* **7**, 11948 (2016). 27582355 10.1038/ncomms11948 PMC5025745

[26] Vaknin, A., Ovadyahu, Z. & Pollak, M. Evidence for interactions in nonergodic electronic transport. *Phys. Rev. Lett.* **81**, 669–672 (1998).

[27] Amir, A., Oreg, Y. & Imry, Y. Electron glass dynamics. *Annu. Rev. Condens. Matter Phys.* **2**, 235–262 (2011).

[28] Yu, C. C. Time-dependent development of the Coulomb gap. *Phys. Rev. Lett.* **82**, 4074–4077 (1999).

[29] Tsigankov, N., Pazy, E., Laikhtman, D. & Efros, L. Long-time relaxation of interacting electrons in the regime of hopping conduction. *Phys. Rev. B* **68**, 184205 (2003).

[30] Ovadyahu, Z. Relaxation dynamics in quantum electron glasses. *Phys. Rev. Lett.* **99**, 226603 (2007). 18233309 10.1103/PhysRevLett.99.226603

[31] Properties and specification sheet for 1-butyl-1-methylpyrrolidinium bis(trifluoromethylsulfonyl)imide. *Sigma-Aldrich* https://www.sigmaaldrich.com/GB/en/product/aldrich/38894 (accessed November 2023).

[32] Xiao, M. et al. Linking glass-transition behavior to photophysical and charge transport properties of high-mobility conjugated polymers. *Adv. Funct. Mater.* **31**, 2007359 (2021).

[33] Bakar, R. et al. Decoding polymer architecture effect on ion clustering, chain dynamics, and ionic conductivity in polymer electrolytes. *ACS Appl. Energy Mater.* **6**, 4053–4064 (2023). 37064412 10.1021/acsaem.3c00310 PMC10091352

[34] Pace, G. et al. Tuning transport via interaction strength in cationic conjugated polyelectrolytes. *Macromolecules* **56**, 6078–6085 (2023).

[35] Ciuchi, S., Di Sante, D., Dobrosavljević, V. & Fratini, S. The origin of Mooij correlations in disordered metals. *npj Quantum Mater.* **3**, 44 (2018).

[36] Brown, P. J., Sirringhaus, H., Harrison, M., Shkunov, M. & Friend, R. H. Optical spectroscopy of field-induced charge in self-organized high mobility poly(3-hexylthiophene). *Phys. Rev. B* **63**, 125204 (2001).

[37] Qarai, M. B., Ghosh, R. & Spano, F. C. Understanding bipolarons in conjugated polymers using a multiparticle Holstein approach. *J. Phys. Chem. C* **125**, 24487–24497 (2021).

[38] Wu, R. et al. Bridging length scales in organic mixed ionic–electronic conductors through internal strain and mesoscale dynamics. *Nat. Mater.* **23**, 648–655 (2024). 38409601 10.1038/s41563-024-01813-3

[39] Simatos, D. et al. Effects of processing-induced contamination on organic electronic devices. *Small Methods* **7**, 2300476 (2023). 10.1002/smtd.202300476 37661594

[40] Schott, S. et al. Polaron spin dynamics in high-mobility polymeric semiconductors. *Nat. Phys.* **15**, 814–822 (2019).

[41] Van Meerten, S., Franssen, W. & Kentgens, A. ssNake: a cross-platform open-source NMR data processing and fitting application. *J. Magn. Reson.* **301**, 56–66 (2019). 30851666 10.1016/j.jmr.2019.02.006

[42] Thurber, K. & Tycko, R. Measurement of sample temperatures under magic-angle spinning from the chemical shift and spin-lattice relaxation rate of ^79^Br in KBr powder. *J. Magn. Reson.* **196**, 84–87 (2009). 18930418 10.1016/j.jmr.2008.09.019 PMC2632797

[43] Dovesi, R. et al. Quantum-mechanical condensed matter simulations with CRYSTAL. *Int. WIREs Comput. Mol. Sci.* **8**, e1360 (2018).

[44] Perdew, J., Ernzerhof, M. & Burke, K. Rationale for mixing exact exchange with density functional approximations. *J. Chem. Phys.* **105**, 9982–9985 (1996).

[45] Fratini, S., Nikolka, M., Salleo, A., Schweicher, G. & Sirringhaus, H. Charge transport in high-mobility conjugated polymers and molecular semiconductors. *Nat. Mater.* **19**, 491–502 (2020). 32296138 10.1038/s41563-020-0647-2

