# Supplementary material for: Non-equilibrium transport in polymer mixed ionic–electronic conductors at ultrahigh charge densities
Source: Nat Mater. 2024 Jul 26;23(12):1712–9. doi: 10.1038/s41563-024-01953-6 (PMC11599050; doi:10.1038/s41563-024-01953-6)
Supplement: Supplementary file 1 — Supplementary Notes 1–10, Figs. 1–57 and Equations (1)–(31). [file 41563_2024_1953_MOESM1_ESM.pdf]

nature materials

Supplementary information https://doi.org/10.1038/s41563-024-01953-6

Non-equilibrium transport in polymer mixed ionic–electronic conductors at ultrahigh charge densities

In the format provided by the authors and unedited

Supplementary Information

This document contains Supplementary Notes 1 to 10, Supplementary Figures 1 to 57, Supplementary Tables 1 to 8, and Supplementary Equations 1 to 31.

Supplementary Note 1	**Extended methods**	p. 2
Supplementary Note 2	**Design considerations of the microfabricated Seebeck devices**	7
Supplementary Note 3	**Band filling in IDT-BT, DPP-BTz, and PBTTT**	9
Supplementary Note 4	***In-operando* microstructural analysis**	19
Supplementary Note 5	**First-principles electronic structure calculations**	38
Supplementary Note 6	**Non-equilibrium transport in IDT-BT, DPP-BTz, and PBTTT**	41
Supplementary Note 7	**Nuclear Magnetic Resonance (NMR) spectroscopy**	57
Supplementary Note 8	**Theory of non-equilibrium transport phenomena**	59
Supplementary Note 9	**Infrared Charge Modulation Spectroscopy (CMS)**	65
Supplementary Note 10	**Double-gated Seebeck measurements**	70

Supplementary Note 1 Extended methods

1.1 Materials and device fabrication

1.1.1 Materials

IDT-BT (poly(indaceno(1,2-b:5,6-b')dithiophene-co-2,1,3-benzothiadiazole) was synthesized according to the previous report in Ref [1]. DPP-BTz (poly[[2,5-bis(2-octadecyl)-2,3,5,6-tetrahydro-3,6-diketopyrrolo[3,4-c]pyrrole-1,4-diyl]-alt-(2-octylnonyl)-2,1,3-benzotriazole]) was synthesized according to the previous report in Ref [2]. PBTTT (poly(2,5-bis(3-dodecylthiophene-2-yl)thieno(3,2-b)thiophene)) with molecular weight M_w 55,000 g/mol, M_n 38,000 g/mol and polydispersity of 1.46 (as measured by GPC in chlorobenzene) was synthesized according to the previous report in Ref [3]. 1-Butyl-1-methylpyrrolidinium bis(trifluoromethanesulfonyl) imide (BMP TFSI, 99.9%, <20 ppm water, <1 ppm halides) was purchased from Solvionic. Anhydrous acetonitrile (>99.9%, <10 ppm water) and 1,2-dichlorobenzene (>99.8%, <20 ppm water) were purchased from Romil Ltd. Poly(vinylidene fluoride-co-hexafluoropropylene) (PVDF-HFP) was purchased from Sigma Aldrich. Acetone used for ion gel solution was purchased from Fisher Scientific.

1.1.2 Substrates

Corning Eagle XG glass substrates were sourced from Corning Inc. SiO_x wafers (Item no. WSI04-1102001 with SiO2-0300-dry, < 0.005 Ohm-cm, 300 nm dry thermal oxide, (100) surface, single-side polished) were sourced from Active Business Company GmbH. SiO_x wafers (Article-No. WWD40525250B1314S501, $1-10$ Ohm-cm, 500 nm wet thermal oxide, (100) surface, single-side polished) were sourced from MicroChemicals GmbH. Undoped SiO_x wafers (Part number W001030, >10 kOhm-cm, 300 nm wet thermal oxide, (100) surface, double-side polished) were sourced from Inseto.

1.1.3 Sample fabrication environment

All conjugated polymer spincoating, annealing, and doping was performed in a nitrogen glovebox (MBraun LabMaster 130) equipped with a regenerable molecular sieve solvent trap. Solvent trap regenerations were performed at least weekly, and the glovebox was purged with fresh nitrogen (200 L min^{-1}) before and during sample preparation. Water and oxygen levels were less than 1 ppm. Polymer powders, ionic liquids, and dopants were stored and weighed in a separate glovebox (Belle Technologies) in which solvent work was not allowed. Water and oxygen levels in this box were less than 10 ppm.

1.1.4 IDT-BT films

Solutions of IDT-BT were prepared in 1,2-dichlorobenzene (DCB) at a concentration of 10 mg mL^{-1} and heated at 80°C overnight before use. IDT-BT films were spincoated from pre-heated 60°C solutions using pre-heated glass pipettes onto 60°C pre-heated substrates. Typical thickness films ($\sim$ 60 nm) of IDT-BT were spincoated at 1,500 rpm for 60 s. The thinner films ($\sim$ 10 nm) for double-gated measurements were diluted to 2.5 mg mL^{-1} and spincoated at 1,500 rpm. IDT-BT samples were dried at 100°C for 5 minutes after spincoating.

1.1.5 DPP-BTz films

Solutions of DPP-BTz were prepared in chlorobenzene (CB) at a concentration of 10 mg mL^{-1} and heated at 110°C before use. DPP-BTz films were spincoated at 2,000 rpm from pre-heated 110°C solutions using pre-heated glass pipettes onto 110°C pre-heated substrates. Thinner films for FET gated measurements were diluted to 2.5 mg mL^{-1} and spincoated at 2,000 rpm. Films were annealed at 110°C for 1 hour after spincoating then allowed to slowly cool to room temperature by switching off the hotplate. Aligned DPP-BTz films were prepared by blade coating with a substrate temperature of 40°C and a blade moving speed of 0.07 mm/s.

1.1.6 PBTTT films

Solutions of PBTTT were prepared in 1,2-dichlorobenzene (DCB) at a concentration of 10 mg mL^{-1} and heated at 80°C overnight before use. PBTTT films were spincoated from pre-heated 80°C solutions using pre-heated glass pipettes onto 80°C pre-heated substrates. Standard thickness films ($\sim$ 40 nm) of PBTTT were spincoated at 1,500 rpm for 60 s, while very thin films ($\sim$ 10 nm, for double-gated measurements) were diluted to 2.5 mg mL^{-1} and spincoated at 1,500 rpm. The films were subsequently annealed at 180°C for 20 minutes to induce the formation of the terrace phase microstructure of PBTTT.[3] The samples were allowed

to cool slowly to room temperature by switching off the hotplate. Aligned PBTTT samples were prepared according to a previous publication.[4]

1.1.7 Ion-exchange doping

Ion-exchange doping was performed as described previously.[5, 6] Briefly, films were sequentially doped using a solution of BMP TFSI (100 mM) and $FeCl_3$ (1 mM) in anhydrous acetonitrile. Doping level was controlled by varying the exposure time to the doping solution. To precisely control the exposure time, doping was performed on the spincoater by setting the first step of the recipe to 0 rpm with a delay equal to the desired doping time, and the second step to 8,000 rpm with maximum acceleration. The spincoater is started at the same moment the doping solution is pipetted onto the film. Shortly after the doping solution was spun off, the film was rinsed with 1 mL acetonitrile while still spinning to remove any residual BMP TFSI and $FeCl_3$.

For NMR samples, the doping method is slightly modified: ~ 0.5 mL of 10 mg/mL IDT-BT solution in dichlorobenzene was drop-casted on a 4×4 cm^2 Corning Eagle XG glass substrate. The dropcast film was ion-exchange doped with 90.9 mM $FeCl_3$ and 909.1 mM BMP TFSI in a nitrogen glovebox over an extended period of ten minutes to ensure effective bulk doping, followed by an hour of "re-exchange" in 100 mM BMP TFSI to minimize any residual $FeCl_3$.

1.1.8 *Ex-situ* electrochemical doping

Ex-situ electrochemical doping of polymer films was performed with a PalmSens4 potentiostat, a Pt counter electrode, and a Ag quasi-reference electrode. Polymer films served as working electrodes and ionic liquid BMP TFSI was used as the electrolyte. We use the chronoamperometry function of the potentiostat to electrochemically dope the films: a constant voltage was applied for 30 s, and then the sample was quickly transferred onto a spincoater to spin off most of the ionic liquid. While the sample was still spinning, the residual ionic liquid was washed away by anhydrous acetonitrile.

1.1.9 Organic electrochemical transistors (OECTs) and double-gated devices fabrication

The regular OECTs used in this work were side-gated and were equipped with multiple electrodes for conductivity and Seebeck measurements. They were fabricated on Corning Eagle XG glass substrates. For the double-gated devices that incorporate an additional field-effect gate on the bottom surface of the semiconducting polymer film we used silicon substrates with a 300 or 500 nm thermally grown silicon oxide as the gate-dielectric and the doped silicon as the bottom field-effect gate electrode. The substrates were first sonicated for 10 minutes in deionized water, acetone, and isopropanol and subsequently cleaned with oxygen plasma at 300 W for 10 minutes. A bilayer photolithography process was used to define the electrode configuration. For devices with platinum electrodes, 5/50 nm NiCr/Pt electrodes were sputter coated with an argon plasma at pressures of $\sim 10^{-4}$ mbar, in a chamber that had previously been pumped to a high vacuum of $\sim 10^{-6}$ mbar. For other devices with gold electrodes, 3/20 nm Cr/Au electrodes were thermally evaporated under a high vacuum of $\sim 10^{-6}$ mbar. More details on device geometry and fabrication process can be found in the following sections. After fabricating the metal electrodes by photolithography and patterning the polymer film to a well defined geometry using the process detailed in Ref. [7], an ion gel layer was spincoated at 5,000 rpm for 20 s to cover both the side-gate and the polymer channel. The ion gel solution was prepared by mixing PVDF-HFP, ionic liquid BMP TFSI, and acetone with a weight ratio of 1:4:20.[8]

1.2 Transport measurements

Transport measurements were primarily done on a multifunctional device in the shape of a Hall bar and equipped with electrodes for four-point-probe conductivity, Hall and Seebeck measurements. OECT measurements were performed either in a nitrogen glovebox or in a cryogenic probe station under high vacuum using instruments listed in Supplementary Table 1. All device architectures that have been used in this study are summarized in Supplementary Figure 1, for reasons that are discussed in Supplementary Note 2. Their use is detailed below:

1. Measurements performed on Device B:
   - Figure 1 (two-probe transfer and Seebeck) of main text.
   - Extended Data Figures 6 (two-probe transfer), 7 (two-probe transfer and Seebeck) and 9(a) (PBTTT two-probe transfer, conductivity, μ_{asym}, and μ_{sym}).
   - Supplementary Figures 4 (two-probe transfer and Seebeck), 5 (four-probe conductivity), 6 (four-probe conductivity), 7 (two-probe transfer), 8, 27 (PBTTT two-probe transfer), and 31 (PBTTT transfer curve).

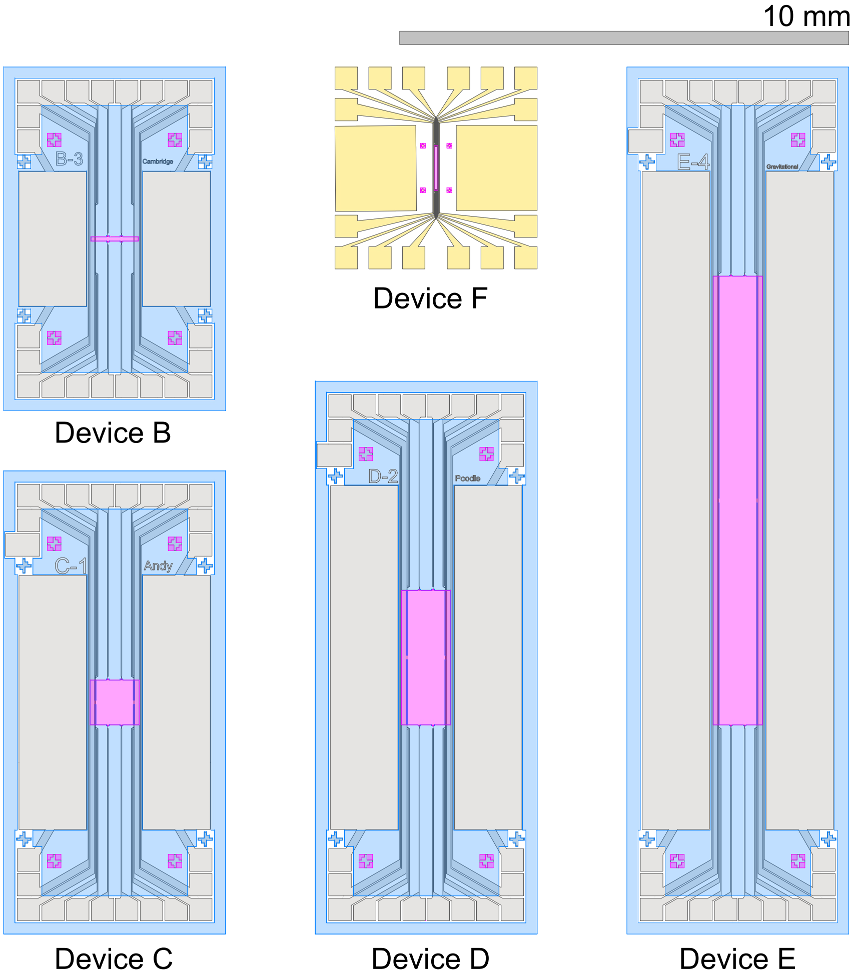

Supplementary Figure 1: **Variations of the on-chip microfabricated multifunctional transport devices.** Key dimensions of the devices are summarized in Supplementary Table 2.

Supplementary Table 1: Voltage measurement resolution of the used instruments.

Instrument	Input impedance	Resolution	Reference
Keithley 2182A Nanovoltmeter	> 10 GΩ	1 nV	[9]
Keithley 2612B SourceMeasure Unit	> 10 GΩ	100 nV	[10]
Keithley 6430 Sub-femtoamp SMU	$> 10^{16}$ Ω	1 μV	[11]
Agilent 4155B SPA	N.A.	2 μV	[12]

2. Measurements performed on Device C:
   - Supplementary Figures 38 (four-probe transfer curves), 39(b) (PBTTT μ_{asym}, μ_{sym}, $\Delta G/G_0$), and 56(f,g,h) (PBTTT two-probe transfer, double-gated Seebeck).
3. Measurements performed on Device D:
   - Supplementary Figures 36 (two-probe transfer curves), 37 (four-probe transfer curves), 39(a) (DPP-BTz μ_{asym}, μ_{sym}, $\Delta G/G_0$).
4. Measurements performed on Device E:
   - Figures 2(c) (two-probe transfer) and 5 (two-probe transfer and Seeebeck) of main text.
   - Supplementary Figures 28 (two-probe transfer), 30 (two-probe transfer), 32 (four-probe transfer), 33 (IDTBT μ_{asym}, μ_{sym}, $\Delta G/G_0$), 34 (two-probe transfer), and 56(c,d,e) (IDT-BT two-probe transfer, double-gated Seebeck).
5. Measurements performed on Device F:
   - Figure 2(b) (two-probe transfer and conductivity) of main text.
   - Extended Data Figures 9(b) (DPP-BTz two-probe transfer, conductivity, μ_{asym}, and μ_{sym}) and 10 (IDT-BT μ_{asym} and μ_{sym}).
   - Supplementary Figures 26 (IDT-BT two-probe transfer curve) and 31 (IDT-BT and DPP-BTz transfer curves).

Most of the transport measurements were performed on ion gel gated devices, allowing the transport physics to be efficiently characterized across a wide range of carrier densities on a single device, while also minimising potential sample-to-sample variations. These ion gel gated measurements were performed at cryogenic temperatures below 240 K to ensure that ions could not move between the ion gel and the polymer and change the doping level during the transport measurements, that are meant to probe a particular doping state. Our low-temperature measurements were performed in two cryogenic probe-stations, a Desert Cryogenics probe-station for measurements down to 80 K and a LakeShore Cryotronics CRX-4K closed-cycle cryogenic probe station for measurements down to 4.2 K. Only for the high temperature measurements between room temperature and 240 K we used *ex-situ* chemically or electrochemically doped films without an ion gel gate in order to fix the number of ions, and thus the carrier density, in the polymer. Our previous report has established the equivalence of the final products of these different doping methods.[5]

The electrical measurement units were chosen by considering the trade-off between the input impedances and the measurement resolutions. This was particularly important in the thermovoltage measurements:

- The highly conducting devices were typically associated with small Seebeck coefficients. The measured thermovoltages in this regime were as low as few μVs, necessitating the use of a high resolution nanovoltmeter, in this case a Keithley 2182A Nanovoltmeter. The use of a nanovoltmeter, however, was only practical for device resistances below 10 MΩ, and alternative measurement units were needed for more resistive devices.

- Measurements on lower conductivity samples were performed using a high input-impedance Keithley 6430 sub-femtoamp source-measure unit with its standard remote pre-amplifier. This unit was used for all thermovoltage measurements of the ion gel gated only IDT-BT, and the lower conductivity states of DPP-BTz and PBTTT — this would correspond to tens of MΩ in resistance terms.

For the ion gel gated devices, we also performed measurements on aligned polymer films prepared by mechanical rubbing (for PBTTT) and solution shear coating (DPP-BTz) to increase the device conductance. The alignment of the polymer increases the conductivity while maintaining the temperature dependence of the Seebeck coefficient unchanged,[4] allowing us to use the high-resolution nanovoltmeter for characterising states with relatively low conductivities.

The conductivity and transfer measurements were less demanding in this respect, since the current and voltage signals were relatively large in these measurements. For these measurements, we found the use of the general purpose Keithley 2612B source-measure unit or the Agilent 4155B Semiconductor Parameter Analyzer to be sufficient.

1.3 Device structures for spectroscopic and structural characterisations

Samples for photoemission spectroscopy experiments were prepared on Corning Eagle XG glass substrate. The samples were prepared on a simple linear four-probe conductivity device architecture (see Supplementary Figure 2), allowing for conductivity measurements prior to the photoemission experiments. 5/50 nm of NiCr/Pt metal electrodes were deposited via sputter coating, with its pattern defined by using a shadow mask. The polymer area is patterned via scratching, typically to an area of $\sim 4 \times 10$ mm^2. For both XPS and UPS, one of the metal electrodes of each sample was electrically grounded to prevent surface charging.

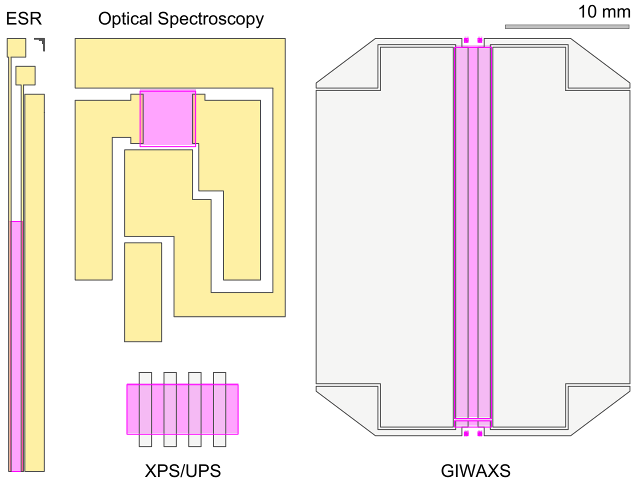

Supplementary Figure 2: **Device structures for spectroscopic and structural characterisations.**

Samples for UV-Vis-NIR measurements were prepared on Corning Eagle XG glass substrates, and on high resistivity undoped silicon wafers with a 300 nm thick wet SiO_2 layer on both sides for the FT-IR measurements. For the *in-situ* UV-Vis-NIR and FT-IR OECT measurements, samples were fabricated using a modified version of the device architecture shown in Supplementary Figure 2 with a smaller 1×1 mm active area. Pt electrode deposition, polymer spincoating, and ion gel spincoating were all conducted as described in Supplementary Note 1.1.9, with patterning done via scratching. For the infrared field-effect gated CMS measurements, samples were fabricated using the device architecture shown in Supplementary Figure 2. The substrate was positioned such that the lower contact (field-effect gate) was deposited over the edge of the substrate, shorting it to the silicon substrate.

GIWAXS measurements were performed *in operando* using an elongated OECT (L = 800 μm, W = 3 cm), as shown in Supplementary Figure 2. The channel needs to be significantly elongated along the X-ray direction to maximize the interaction between the film and the X-ray beam and thus the scattering signal of the polymer film under grazing incidence conditions. GIWAXS devices were prepared on a silicon wafer with 300 nm thermally grown oxide to prevent current-shorting/short-circuit through the doped silicon substrate, while reference samples were prepared on native oxide Si wafers. The pattern of both the metal electrodes and the polymer film were defined by photolithography.

Supplementary Note 2 Design considerations of the microfabricated Seebeck devices

In designing our transport devices, care has been taken to ensure the accuracy of the measured Seebeck and conductivity values, which will be described in this Supplementary Note. With this consideration, we have developed five different architectures as shown in Supplementary Figure 1, with their important dimensions summarized in Supplementary Table 2.

Conductivity and Seebeck measurements of ion gel gated only devices were performed on the Device B architecture. Device F was used for proof-of-concept double-gated transfer curve measurements, whereas more detailed measurements have been performed on Device C for PBTTT thin films, Device D for DPP-BTz films, and Device E for IDT-BT films.

A common feature of the materials of interest in this study is their small Seebeck coefficients, typically on the order of tens of μV K^{-1}, and at most ~ 300 μV K^{-1} for the more insulating polymers. Measurement of such small voltages are challenging, requiring thoughtful design of our thermoelectric devices. A long device channel is helpful to increase the temperature difference between the hot and cold sides, inducing higher thermovoltage to be measured. Here, the distance between the two thermometers are kept at 855 μm for all devices that have been used for Seebeck measurements. This however comes at a cost of increasing the device resistance, which at some point may become too large for our measurement units, e.g. the high-resolution nanovoltmeter. For such cases where both the electrical conductivity and Seebeck coefficient are relatively low, a wider channel is needed to increase the device conductance without compromising the channel length. All ion gel gated only measurements were done using on Device B. These relatively thick PBTTT, DPP-BTz, and IDT-BT films (~ 60 nm) were conductive enough to be measured on Device B's relatively narrow channel. Devices C, D, and E are all designed for increased device conductances to accommodate for thinner (~ 10 nm) polymer films in the double-gated measurements. IDT-BT is the most resistive of the three polymers, therefore we used the widest channel for IDT-BT.

Supplementary Table 2: Key dimensions of the devices shown in Supplementary Figure 1.

	Channel length L	Channel width W	Distance between hot and cold sides
Device B	900 μm	100 μm	855 μm
Device C	900 μm	1,000 μm	855 μm
Device D	900 μm	3,000 μm	855 μm
Device E	900 μm	10,000 μm	855 μm
Device F	50 μm	1,000 μm	60 μm

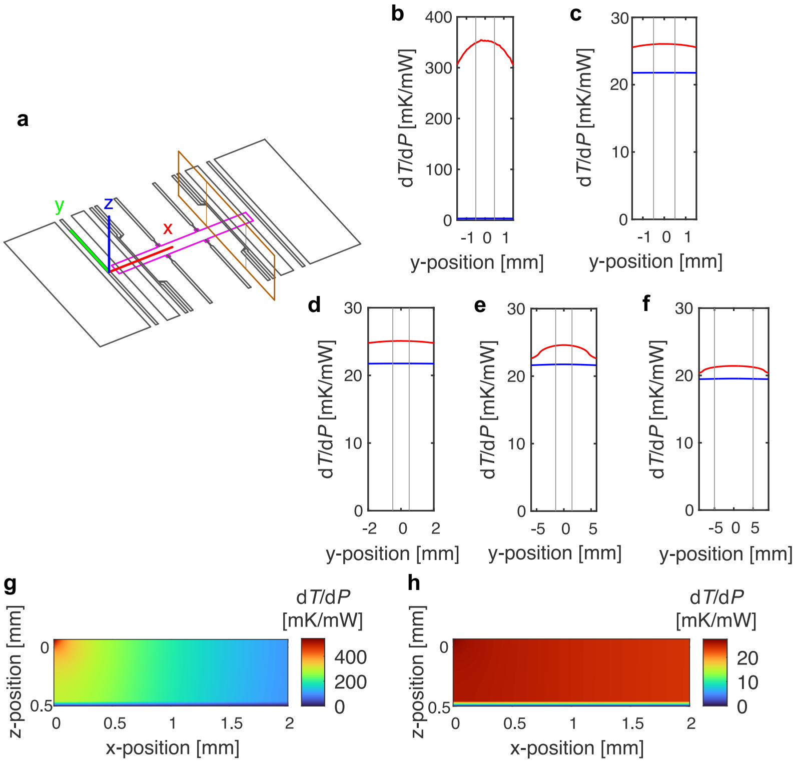

Supplementary Figure 3: **Simulated temperature profile in the microfabricated Seebeck devices.** (a) Schematic device, showing the reference coordinate system. Temperature profile along the y-direction on the xy-plane for (b) Device B on glass, as well as (c) Device B, (d) Device C, (e) Device D, and (f) Device E on silicon wafer. Notice that the temperature profile is uniform within the region over which the resistance thermometer is calibrated, as indicated by the grey vertical lines. Temperature profile on the xz-plane at $y = 0$ for (g) glass and (h) silicon substrates, demonstrating the effects of the much higher thermal conductivity of the latter.

In the following we focus on Device B as an example to illustrate our design considerations. To estimate the temperature gradient that would be evolved on this channel geometry, we performed finite-element simulation of the steady-state temperature distribution in response to the application of an arbitrary thermal power on the heater wire. For a 18 mm · 20 mm Eagle XG glass substrate (thermal conductivity: 1.09 W m^{-1} K^{-1} and heat capacity: 0.77 J g^{-1} K^{-1}) with a thickness of 525 μm and a moderate interfacial thermal conductivity of $2{\cdot}10^{-3}$ W m^{-1} K^{-1}, every 1 mW of applied heater power gives rise to temperature increases of 350 mK on the hot side and only around 5 mK on the cold side. Importantly, there is negligible temperature variation along the relevant length of the thermometer, i.e. along the y-direction (see coordinate system in Supplementary Figure 3(a)). Supplementary Figure 3(b) shows the temperature gradient along the y-direction, emphasizing that between the two voltage probes (grey vertical lines) used for the four-probe resistance calibration of the thermometer there is not a significant temperature variation for both the hot (red line) and cold (blue line) sides.

The double-gated measurements are performed on silicon substrates in order to make use of the SiO_2 dielectric for field-effect gating. The thermal conductivity of the silicon substrate (thermal conductivity: 148 W m^{-1} K^{-1} and heat capacity: 0.70 J g^{-1} K^{-1}) is significantly larger than its Eagle XG counterpart. As a result there is a significant temperature gradient out-of-plane and in-plane in a glass substrate, as illustrated in Supplementary Figure 3(g). This is not the case for a silicon substrate where its thermal conductivity is so high such that the bottleneck for thermal transport is by and large only the interfaces. In Supplementary Figure 3(h) the temperature of the silicon bulk is relatively uniform, and most of the temperature drop takes place at the interface, i.e. at $z \sim 0.5$ mm. Assuming the same architecture of Device B and that we are only changing the substrate from glass to silicon, its temperature gradient along the y-direction is shown on Supplementary Figure 3(c). Application of 1 mW of heater power leads to a significantly lower temperature increase on the hot side of only ~ 25 mK, resulting in only a small temperature difference on the substrate. It needs to be emphasized that the temperature gradient along the y-direction remains negligible within the boundaries of the resistance measurement length. Therefore, the architecture remains functional for Seebeck measurements on silicon substrate, although at a much lower temperature difference between the hot and cold sides.

Supplementary Note 3 Band filling in IDT-BT, DPP-BTz, and PBTTT

We have concentrated on IDT-BT in the main text due to the extensive degree of band filling that can be achieved in this polymer, spanning Regimes I, II, and III. This has allowed us to use IDT-BT as a model system to comprehensively demonstrate most features of the novel transport physics. However, to demonstrate the generality of our observations we have also investigated a range of other polymers, including DPP-BTz and PBTTT. DPP-BTz is a semicrystalline polymer with intermediate paracrystallinity of around $g \sim 12-13\%$. DPP-BTz is strictly speaking not a donor-acceptor polymer since both DPP and BTz are electron withdrawing. PBTTT is a polythiophene-based, semicrystalline polymer that can be doped to very high conductivities of $> 1{,}000$ S cm^{-1}, while retaining a high degree of microstructural order with low $\pi-\pi$ stacking paracrystallinity $g \sim 8\%$.[6] As a pure donor polymer PBTTT is expected to have a significantly wider intrachain bandwidth than IDT-BT and DPP-BTz. The different band filling characteristics observed in these three polymers are representative of the behaviour observed in a wider range of polymer. Depending on the polymer we either observed only Regime I (as in PBTTT), Regimes I and II (as in DPP-BTz; see Extended Data Figure 7 for PBTTT and DPP-BTz data) or Regimes I, II and III (as in IDT-BT). Apart from the MOEMP FAP gated PBTTT device in Supplementary Figure 4, the PBTTT and DPP-BTz samples discussed in this Supplementary Note were uniaxially aligned, to help with measurements of the Seebeck coefficients as discussed in Supplementary Note 2.

3.1 OECT transfer characteristics of PBTTT and DPP-BTz

PBTTT has a monotonic ion gel gated transfer curve, with the source-drain current constantly becoming larger with increasingly negative gate-voltages in Regime I as shown in Extended Data Figure 7(b). At very low doping levels, we saw a mostly insulating behaviour, with low values of electrical conductivity and relatively large Seebeck coefficients of up to around 100 μV K^{-1}. At conductivities of few hundreds of S cm^{-1} and more, an insulator-to-metal transition was seen with the samples showing sizeable conductivities at liquid-helium temperatures, and the Seebeck coefficients (Extended Data Figure 7(c)) becoming small and linearly varying with temperature, obeying the Mott formula for a metal. The transport characteristics of our metallic samples are fully consistent with previous reports on highly-doped PBTTT samples.[4, 13] We have explored the possibility of a crossover to Regime II in our BMP TFSI doped PBTTT, but we were unable to detect this. We detected only a marginal decrease in the source drain current at very high gate voltages beyond -3 V; however this seems to be caused by electrochemical degradation of the polymer and not a transition to Regime II, as corroborated by a concurrent increase in the gate leakage. This is consistent with the GIWAXS data discussed below that TFSI doped PBTTT adopts a stable structure with one dopant counterion per repeat unit and that it is very difficult to incorporate further TFSI anions into this structure.

Frisbie and coworkers recently reported a conductivity peak in their PBTTT electrochemical transistors gated with BMI FAP ion gels.[14] We have studied PBTTT OECTs with MOEMP FAP ion gels, where FAP ions are incorporated in the polymer film on p-doping and observed a similar decrease in the source drain current at high gate voltages, as shown in Supplementary Figure 4(a). As seen in IDT-BT (Figure 1 of main text) and DPP-BTz (Extended Data Figure 7), such conductivity peak could potentially be a transport signature indicating the crossover from Regime I to II. However, we caution that in these PBTTT devices we do not see a carrier polarity switch in the Seebeck coefficient to n-type on passing the conductivity peak, as seen in IDT-BT and DPP-BTz. Here, the Seebeck coefficient remains p-type and becomes smaller beyond the conductivity peak. The lack of n-type inversion in Frisbie's and our studies suggests that that the peak in the transfer curve observed in FAP-doped PBTTT is in fact not associated with Regime II operation, but may be associated instead with either an increase of microstructural disorder upon incorporation of the relatively large FAP ions or the hole-anion interactions playing a more important role in PBTTT than in IDT-BT. We attribute the difficulty of entering Regime II in PBTTT to the larger intrachain bandwidth of PBTTT, such that the electronic states that correspond to Regimes II and III are located far deeper in energy and remain inaccessible. This picture agrees with first principle calculations of PBTTT's electronic structure, shown in Extended Data Figure 8.

DPP-BTz exhibits a peak in its ion gel gated transfer curve, beyond which it enters Regime II and its Seebeck coefficient turns n-type. In contrast to IDT-BT, the transition from p-type to n-type does not occur exactly at the peak at room temperature, though at low temperature the n-type behaviour sets in close to the peak. At the highest doping levels in Regime II DPP-BTz exhibits a similar, near temperature independent Seebeck coefficient as IDT-BT. However, DPP-BTz does not enter Regime III. As we approach a fully insulating state at the end of Regime II the n-type Seebeck coefficient becomes larger and larger, but its temperature dependence becomes more and more difficult to measure accurately as the samples become very resistive. This strongly n-type response of DPP-BTz suggests that the Seebeck coefficient is governed

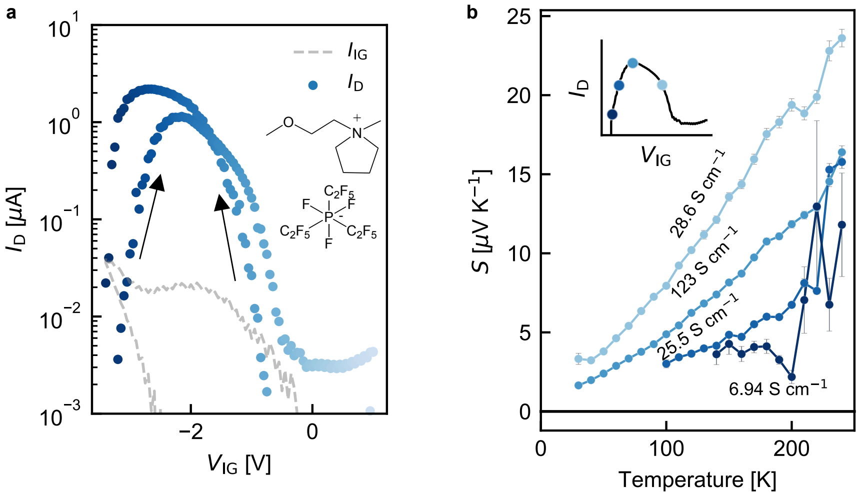

Supplementary Figure 4: **Absence of Regime II on passing the conductivity peak in FAP doped PBTTT.** (a) Transfer curve of MOEMP FAP gated PBTTT, showing a peak in conductivity (inset is the molecular structure of MOEMP FAP). (b) Seebeck coefficient showing the absence of transition to n-type transport in Regime II. Data are presented as the mean Seebeck coefficients ± standard error of the mean, originating from fitting uncertainties of the on-chip thermometer calibration and the thermovoltage versus temperature difference plots.

by the electrons remaining at the bottom of the HOMO band, with minimal contributions from holes on the top of HOMO-1. This is consistent with our interpretation that the bandgap between HOMO and HOMO-1 is larger in DPP-BTz compared to IDT-BT and explains the absence of Regime III in DPP-BTz.

3.2 Temperature dependence of conductivity of PBTTT, DPP-BTz and IDT-BT

To obtain further insight into the transport physics we studied the temperature dependence of the electrical conductivity $\sigma(T)$. In Supplementary Figure 5, we present the absolute and normalized $\sigma(T)/\sigma(200\text{ K})$ conductivity values of PBTTT, DPP-BTz, and IDT-BT as a function of doping level. The measurements were performed using the linear four-probe conductivity technique, thus eliminating the effect of contact resistance on both the magnitude and the temperature dependence of the conductivity. It is only for the more conducting PBTTT samples that we saw a clear conductivity signature of the presence of metallic states, with up to 60% of the 200 K conductivity remaining at liquid helium temperatures. Even for these samples, we saw mostly thermally activated transport, with metallic transport signatures, i.e. a decrease in conductivity with increasing temperature, being observed only at the highest temperatures (> 200 K) and in the most highly doped samples. While the temperature dependence of DPP-BTz's electrical conductivity was found to be stronger than PBTTT, a finite conductivity of around 0.1 S cm^{-1} remained at 5 K on the conductivity peak. IDT-BT, on the other hand, showed significantly stronger thermal activation compared to the two other polymers and became practically insulating with $\sigma < 10^{-3}$ S cm^{-1} at $T < 50$ K even for the highest conductivity states. From variable temperature conductivity alone, it is therefore not obvious that delocalized metallic states are present in any of the polymers, although other transport and spectroscopic signatures have established their presence unambiguously. The apparent contradiction between the thermally activated temperature dependencies of electrical conductivity indicating hopping and signatures of metallic transport, such as the linear temperature dependence of the Seebeck coeffiicent or the presence of a Pauli paramagnetic susceptibility, is often attributed to the presence of morphological heterogeneity of the polymer and is accounted for in Kaiser's heterogeneous transport model [15]). Kaiser's model proposes that while delocalized carrier wavefunctions exist in the crystal domains, thermal activation is needed for transport through the disordered grain boundary regions. The Seebeck coefficient in such heterogeneous microstructure is governed by the region over which most of the temperature gradient is evolved i.e. the ordered domains, while the electrical conductivity is reflective of the most resistive component in the transport pathway, i.e. the grain boundaries.

It is instructive to analyse the measured temperature-dependent conductivity in terms of a variable-range hopping framework, in which electrical conductivity scales with temperature as $\sigma = \sigma_0 \exp\left(-T_0/T\right)^{1/(d+1)}$. Here σ_0 is a pre-exponential factor, T_0 is the hopping temperature, and d is the dimensionality of hopping.

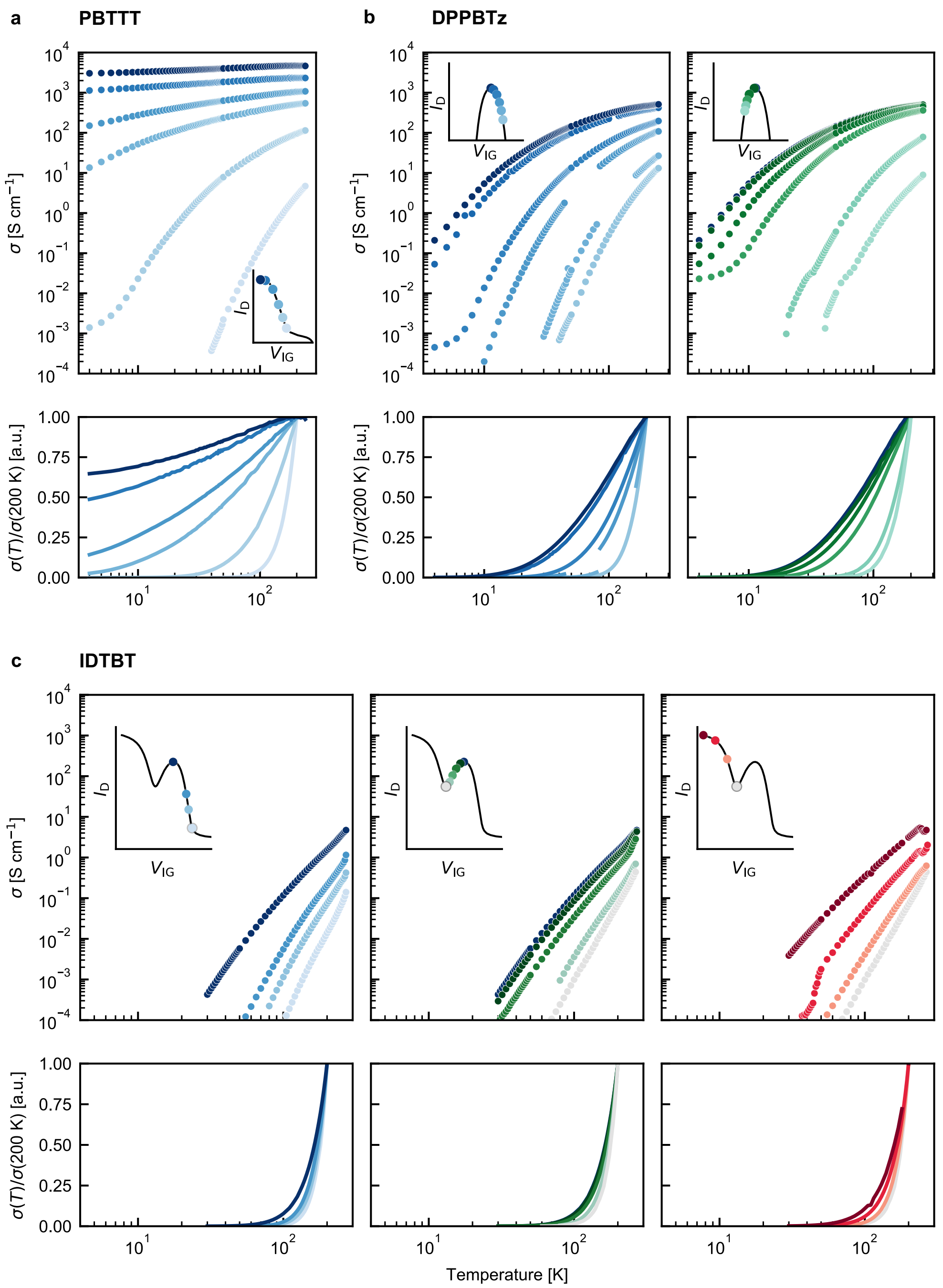

Supplementary Figure 5: **Thermally activated electrical conduction in doped polymers.** Top panels show the temperature dependence of conductivity in (a) PBTTT, (b) DPP-BTz, and (c) IDT-BT. Bottom panels show the conductivity at low temperatures normalized to the 200 K value, $\sigma(T)/\sigma(200\text{ K})$.

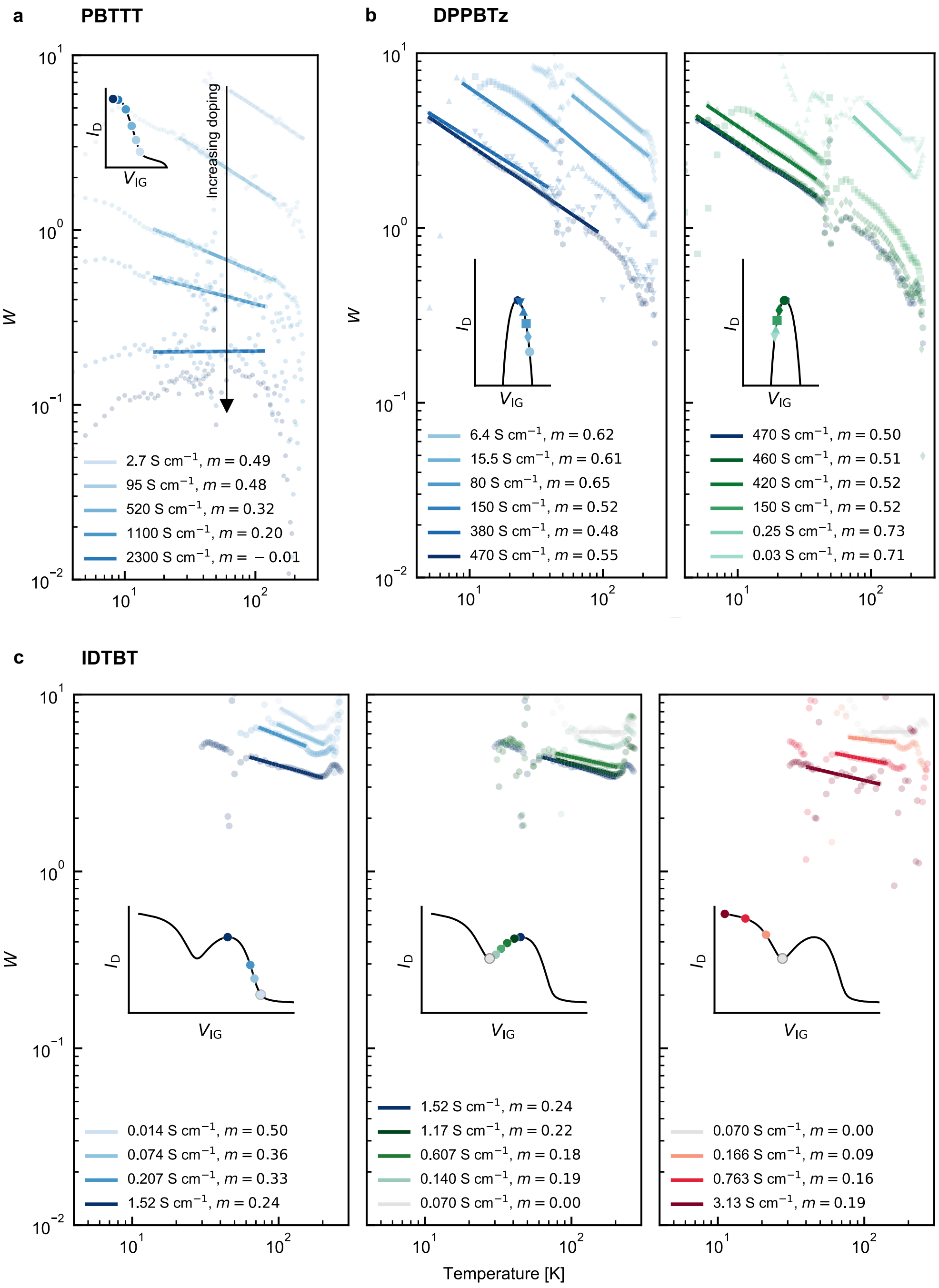

Supplementary Figure 6: **Variable range hopping fits of the conductivity data.** The data points are plotted as Zabrodskii plots, i.e. plots of $W = \mathrm{d}\ln\sigma/\mathrm{d}\ln T$ against T on a double logarithmic scale, where the fitting exponent m is then given by the slopes of the curves. Scatter symbols denote experimental data points, while solid lines correspond to the fits. Shown in the panels are fitting results of (a) PBTTT, (b) DPP-BTz, and (c) IDT-BT.

We are mostly interested in the exponent $m = 1/(d+1)$ which contains information on both hopping dimensionality d but can also indicate the importance of electron-electron interaction and formation of Coulomb gap when $m = 1/2$. The most unbiased way of assigning m is through Zabrodskii analysis, where the reduced activation energy $W = \mathrm{d}\ln\sigma/\mathrm{d}\ln T$ is plotted as a function of temperature on a double logarithmic scale, such that m is the slope of the datapoints in this representation. Zabrodskii plots for all polymers of interest are shown in Supplementary Figure 6. We note that m for PBTTT (< 100 S cm^{-1}) and IDT-BT (~ 0.01 S cm^{-1}) at the lowest doping levels, and DPP-BTz at the conductivity peak, is indeed close to 0.5. This could indicate Efros-Skhlovskii variable-range hopping, where strong carrier interactions drive the formation of a Coulomb gap, which is consistent with our interpretation of the non-equilibrium transport data discussed below. However, m varies significantly with doping level: In PBTTT and IDT-BT, m decreases as the sample is doped to higher levels. $m \sim 1/3$ for 520 S cm^{-1} PBTTT and 0.21 S cm^{-1} IDT-BT in Regime 1, which would be the exponent expected for a two-dimensional variable-range hopping process. At even higher doping densities, PBTTT becomes metallic with a positive slope of W and thus weakly negative m. In IDT-BT, m also becomes smaller at these higher doping levels, reaching $m \sim 1/4$ on the conductivity peak. Rather interestingly, m is considerably lower for Regimes 2 and 3 of IDT-BT being almost around 0.2 or lower, decreases towards the valley in both regimes, and is lowest at the valley. DPP-BTz, rather differently shows values of m that are higher than 0.5, with the value increasing away from the conductivity peak towards the most insulating states in Regimes 1 and 2. We do not attempt a detailed interpretation of this complex behaviour of the m values here, but merely state that the temperature-dependent conductivity in all three polymers bears the signatures typically observed in many disordered metals.

3.3 Band filling in ultraviolet photoemission spectroscopy

Consistent band filling signatures were also observed in the changes of the polymers' frontier molecular orbitals upon doping, experimentally probed through ultraviolet photoemission spectroscopy (UPS).

The UPS spectrum of undoped PBTTT (Extended Data Figure 2(a)) shows a broad HOMO feature. Upon doping, we observed bleaching of the HOMO feature intensity, consistent with removal of electrons from HOMO in p-doping, and the Fermi level moving into the band edge indicative of metallic states (see inset of Extended Data Figure 2(a)). Our use of monochromatic He source minimizes the likelihood of the finite signal at Fermi level coming from polychromaticity of the photon source.

In DPP-BTz (Extended Data Figure 2(b)), an approximately Gaussian-shaped HOMO is observed for the undoped sample. As the doping level is increased towards the conductivity peak, we see a finite density-of-states at the Fermi level along with bleaching of the HOMO peak. It is in DPP-BTz that we see the clearest evidence for band filling: at very high doping levels in Regime II where the material returns to its insulating state, the HOMO has completely disappeared, and the Fermi level lies within a gap, as in an undoped semiconductor, consistent with complete filling of the HOMO band.

In contrast, even in the undoped state of IDT-BT its HOMO shows less feature than DPP-BTz, likely due to multiple overlapping bands as suggested by the closely spaced redox events in cyclic voltammetry (Extended Data Figure 3 and Supplementary Note 3.6). We cannot resolve an insulating state at the transition between Regimes II and III in UPS which is consistent with the transfer characteristics. We see finite density-of-states at the Fermi level for all doped IDT-BT samples, with noticeably stronger intensity for the Regime III sample.

Consistently with the DFT calculated band structures (Extended Data Figure 8), the experimental HOMO of PBTTT is significantly wider than that of IDT-BT and DPP-BTz. This serves as yet another independent, photoemission-based evidence support for our interpretation of larger HOMO bandwidth resulting in the absence of Regimes II and III in PBTTT.

3.4 Estimation of carrier concentration from X-ray photoemission spectroscopy

We considered two techniques for estimating carrier densities in our highly doped polymers: X-ray photoemission spectroscopy and gate charging current analysis. In highly ion-exchange doped PBTTT films, we have previously demonstrated X-ray photoemission spectroscopy (XPS) to be a reliable and practical method,[6] which provides similar doping concentrations to quantitative nuclear magnetic resonance (QNMR). XPS cannot be performed *in-situ* on the OECTs, but we used *ex-situ* electrochemically doped polymer films. We were able to monitor continuously the doping level throughout the electrochemical doping process by regularly taking cyclic voltammograms and/or I-V characteristics. This feedback allowed us to fine tune the doping state in Regimes I, II, and III.

Quantitative XPS analysis of these TFSI doped polymers takes advantage of the fact that the dopant anion and all of the polymers contain a common element, sulfur. This allows us to minimize uncertainties associated with the use of relative sensitivity factors in quantitative analyses involving different elements. The doping levels, here quantified as the number of dopant ion per monomer n, can then be estimated by taking the ratio of the integrated area of the chemically shifted sulfur peak of the TFSI$^-$ anion molecules

to the sulfur peak of the polymers. Despite the surface sensitive nature of XPS experiments, our previous depth profiling experiments on ion-exchange doped samples [5] have established the uniformity of doping level across the film thickness.

The sulfur 2p X-ray photoemission spectra of PBTTT, DPP-BTz, and IDT-BT at various doping levels are presented in Extended Data Figure 1. For the undoped PBTTT and DPP-BTz, a single S2p doublet, comprising of the two spin-orbit components $S2p_{1/2}$ and $S2p_{3/2}$, is sufficient to fit the sulfur spectra. This doublet arises from the neutral $(C\text{-}S\text{-}C)^0$ unit in PBTTT's and DPP-BTz's thiophene as well as PBTTT's thienothiophene rings. In PBTTT, strictly there should also be chemical inequivalency between the sulfur atoms of the thiophene and the thienothiophene units — we presume that these are not distinguishable within the limits of the instrument resolution. The constraints of these fittings are that the binding energies of the spin-orbit components are split by 1.18 eV, and that the ratio of the two integrated intensities is 0.5. These parameters were fit for the undoped polymer and then fixed for the whole doping range. For undoped IDT-BT, two pairs of doublet are needed to fit the sulfur spectrum. We attribute the two doublets to the different chemical environments of the sulfur atoms in the IDT's $(C\text{-}S\text{-}C)^0$ and the BT's $(N\text{-}S\text{-}N)^0$ units, the latter showing higher binding energy as it is more electronegative. We assume that the same 1.16 eV spin-orbit splitting applies between the doublet components of the IDT's $(C\text{-}S\text{-}C)^0$ and the BT's $(N\text{-}S\text{-}N)^0$ units.

For the doped PBTTT and DPP-BTz, we firstly note that another sulfur doublet is seen at significantly higher binding energies (~ 168 eV) than the polymer sulfur doublet. We attribute this new doublet to the $TFSI^-$ anion that gets incorporated into the film upon doping, represented by S_1^{TFSI} in Extended Data Figure 1. The significant chemical shift between the polymer and the ion doublets reflect the very different electron densities of the two. In addition to this, we notice that the polymer peak, in contrast to the undoped case, can no longer be satisfactorily fit with a single S2p doublet. We conclude, in agreement with Winkler's C60 [16] and Jacob's PBTTT [6] interpretations, that the second polymer S2p doublet has got to be associated with sulfur atoms whose binding energy are increased in the vicinity of hole polarons. In our previous analysis of $HFSI^-$ doped PBTTT we found that two doublets were needed to satisfactorily fit the $HFSI^-$ sulfur peak, which we attributed to one of the SO_2 groups being closer to the polymer backbone than the other.[6] Here, we only see the need for two doublets in the very highly $TFSI^-$ doped DPP-BTz (right most panel of Extended Data Figure 1(b)) which we annotate as S_2^{TFSI}. While we attempted to fit all the polymers XPS spectra using a single peak for TFSI anions, in this specific case a good fit required two peaks, similar to our previous publication.[6]

In doped IDT-BT, we see shifts towards higher binding energies on the polymer sulfur peaks of both the donor and the acceptor units. We interpret this as an evidence of the absence of systematic preferential doping on either the IDT or the BT units.

The results of these carrier density analyses are indicated next to the $TFSI^-$ peak in Extended Data Figure 1. For the highest electrochemical doping levels attainable in PBTTT, corresponding to conductivities of more than 350 S cm^{-1} in an unaligned sample, we saw carrier densities of around ~ 0.7 ions per monomer (Extended Data Figure 1(a)). This is in good agreement with our previous XPS experiments on ion-exchange doped PBTTT samples.[6] For the less conducting samples in our present study, we noted a carrier density of approximately 0.3 to 0.4 ions per monomer at the point PBTTT becomes metallic.

We note that a similar upper limit of carrier densities of around 0.6 ions per monomer has also been inferred through gate charging current analysis in Frisbie's BMI FAP PBTTT report.[14] The relatively small carrier densities attainable in this FAP system, which is not greater than that observed in our TFSI experiments, along with the lack of n-type transport in the region of decreasing conductivity provide ample support that this peak in conductivity is not associated with Regime II transport, as laid out in Supplementary Note 3.1.

Our previous reported carrier density estimates of ion-exchange doped DPP-BTz and IDT-BT films are 0.57 and 0.80 respectively.[6] The typical conductivity of similarly doped unaligned DPP-BTz van der Pauw device is around 80 S cm^{-1}, and it is 10 S cm^{-1} for unaligned IDT-BT. While these carrier densities are high, identification of their exact doping states was beyond the scope of the previous study, and is not trivial via ion-exchange doping. In the present study, we opted to work on electrochemically doped samples since they in general allow better control over the doping states; this is made possible by sampling cyclic voltammograms and/or I-V characteristics as mentioned above.

Indeed in IDT-BT we have been able to precisely adjust the doping level. We found that the carrier density on IDT-BT's conductivity peak of ~ 10 S cm^{-1} is consistently 1 ion per monomer in multiple repeats of the experiment, agreeing with half filling of the HOMO. However, at higher carrier densities, e.g. for the valley, we see less consistency between the carrier densities inferred from repeated experiments, which we attribute to the sample instabilities under the demanding experimental conditions. For instance, in this particular dataset, the carrier density on the second rise of 1.23 ions per monomer ($\sigma = 17.5$ S cm^{-1}) is slightly lower than that of the valley's 1.26 ions per monomer ($\sigma = 1.23$ S cm^{-1}). We have identified several experimental challenges in these XPS measurements at the very high doping levels in IDT-BT. First, we have seen a tendency for these samples to dedope under ultrahigh vacuum. As such, within the practical

timescale of an experiment ($\sim$ 1 hour of signal acquisition), there is likely to be a reduction in the carrier density. Moreover, we have indications of dedoping associated with X-ray irradiation. Beam damage was particularly evident in our preliminary experiments with a non-monochromatic AlKα source (Thermo Fisher Escalab 250Xi), which was manifest as a decrease in the TFSI S2p doublet intensity with increasing X-ray exposure. We have since alleviated some of these problems by using a monochromatic AlKα source (JEOL JPS-9030) and thus significantly lower fluxes. However, the possibility of some remanent degradation cannot be fully ruled out. In Supplementary Note 3.5 we discuss how a combination of XPS and gate charging current analysis could provide a better estimate of IDT-BT's carrier density at the valley.

In DPP-BTz, in addition to sample dedoping under ultrahigh vacuum, we encountered an extra technical challenge in the adjustment of the doping level. Here, although the doping state could be well adjusted while the ionic liquid remains on the polymer surface, we see significant dedoping when the sample is rinsed with acetonitrile. For instance, a sample that is doped well into the insulating state of Regime II typically becomes more conducting after rinsing, and similarly a sample around the peak conductivity would become significantly less conducting. As a consequence, it is considerably more difficult to maintain the accuracy of the doping level in DPP-BTz after the acetonitrile rinsing step. In a sample that had been intended for the peak conductivity we see a carrier density of only 0.40 ions per monomer, corresponding to a conductivity of 30 S cm^{-1}. To produce a sample close to the peak we intentionally overdoped samples into Regime II to compensate for the loss in the carrier density, but even in these the final carrier density appeared slightly lower than that at the actual peak. For the insulating state in Regime II we doped the sample as much as possible by applying the highest practical doping voltage (1.8 to 2 V). The complete bleaching of the HOMO band in the UPS spectra of this sample (Extended Data Figure 2(b)), as well as their very low conductivity of 0.4 S cm^{-1}, provides a confirmation of the highly doped nature of this sample. At this doping state we recorded a very high carrier density of $\sim$ 1.5 ions per monomer (although the dedoping issues still exist).

To conclude, in IDT-BT and DPP-BTz we have confirmed the possibility of reaching doping levels of higher than 1 ion per monomer, consistent with doping beyond Regime I. We have been able to establish with confidence that the doping level at the conductivity peak of IDT-BT (between Regimes I and II) is 1 ion per monomer. In IDT-BT, estimating the carrier densities in Regimes II and III accurately has proven challenging due to doping instabilities under the demanding experimental conditions of the photoemission setup. We have recorded carrier densities of $\sim$ 1.2 on the valley which should be treated as a lower bound value, considering the experimental challenges. In DPP-BTz, significant sample dedoping following the ionic liquid rinsing step rendered it challenging to adjust the doping level to a high degree of accuracy. In our most doped sample, however, we saw a doping level of 1.5 ions per monomer, which is significantly larger than the threshold for accessing transport in Regime II. Importantly, in any of our PBTTT samples we have never observed carrier densities of greater than $\sim$ 0.7 ion per monomer, providing further support to our argument that the carrier densities attainable in PBTTT are not high enough to reach Regime II and beyond.

3.5 Estimation of carrier concentration from gate charging current analysis

We applied a complementary analysis using the gate-charging current to provide a better estimation of IDT-BT's carrier density in the very highly doped regimes. Such analysis is a commonly used technique for carrier density estimation in the OECT literature, e.g. in Frisbie's recent work discussed above.[14] Integrating the gate current in a transfer curve measurement, we obtain an estimate of the charge density p:

$$p = \frac{1}{reLWt} \int I_{\mathrm{G}} \mathrm{d}V_{\mathrm{IG}}, \tag{1}$$

where r is the constant sweep rate of the ion gate voltage V_{IG}, e is the magnitude of an elementary charge, and L, W, and t are the length, width, and thickness of the polymer channel. Taking into account the mass of the monomer M and the density of the polymer ρ, we arrive at the charge per monomer pM/ρ.

This routine method is, however, often complicated by several experimental difficulties. First, the densities of many polymer semiconductors, including IDT-BT and DPP-BTz, are not accurately known. Second, there will inevitably be device-to-device variations in the dimensions of the polymer, e.g. the film thickness. Therefore, it is typically rather challenging to accurately estimate carrier densities based on gate-current analysis solely. We illustrate this problem by showing in the top panels of Supplementary Figure 7 the gate current I_{G} of two devices, Device 1 and Device 2, that were fabricated on the same substrate. It is evident that the absolute values of the gate-current, and thus the derived carrier densities, of the two devices are significantly different, with Device 1's current being almost twice as large as that of Device 2. In reality, this difference is likely to represent differences in the polymer dimensions, and is not an actual reflection of carrier density difference.

We propose a way to circumvent this practical problem by taking a ratiometric approach. In this approach we first suppose that the carrier density at the conductivity peak n_{peak} is 1 ion per monomer as consistently observed from XPS. We then measure how much more charges is introduced in the charging process from

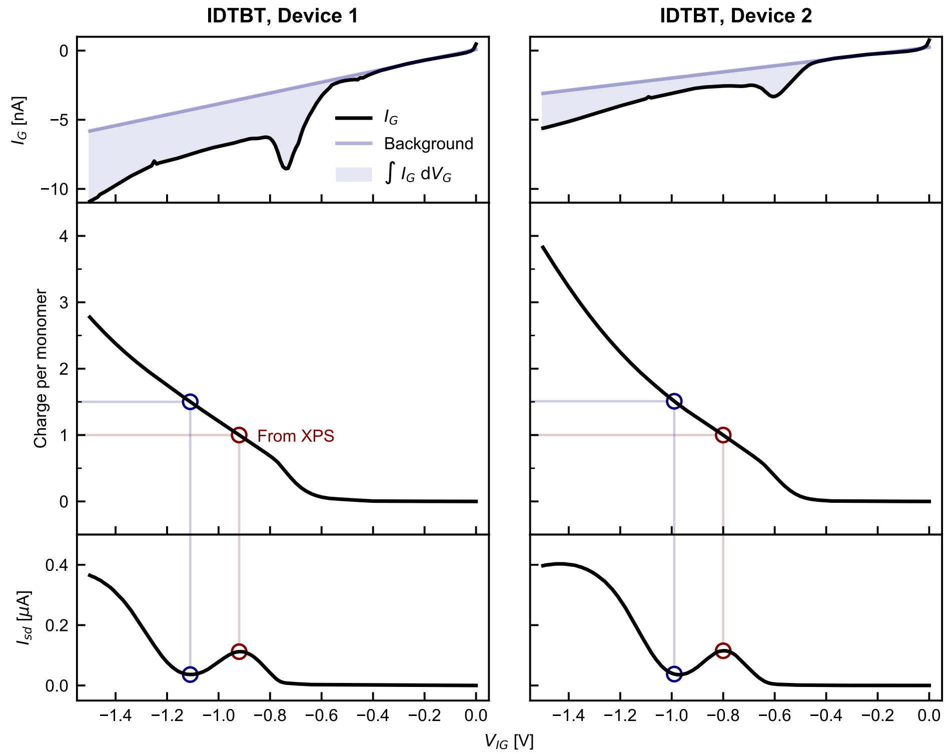

Supplementary Figure 7: **Complementary estimation of carrier density based on charging gate-current.**

the peak to the valley, based on the gate charging current. Within this approach, the carrier density on the valley n_{valley} is given by:

$$n_{\mathrm{valley}} = \int_{V_{\mathrm{IG}}=0}^{V_{\mathrm{IG,valley}}} I_{\mathrm{G}} \mathrm{d}V_{\mathrm{IG}} = \left(\frac{\int_{V_{\mathrm{IG}}=0}^{V_{\mathrm{IG,valley}}} I_{\mathrm{G}} \mathrm{d}V_{\mathrm{IG}}}{\int_{V_{\mathrm{IG}}=0}^{V_{\mathrm{IG,peak}}} I_{\mathrm{G}} \mathrm{d}V_{\mathrm{IG}}} \right) \cdot n_{\mathrm{peak}}, \tag{2}$$

as illustrated in Supplementary Figure 7. In performing the gate current integration we have taken off the linear background from the linear response at relatively low V_{IG}. Despite the significant differences in the absolute values of gate current I_{G} of Devices 1 and 2, we see that the resulting ratio $\int_{V_{\mathrm{IG}}=0}^{V_{\mathrm{IG,valley}}} I_{\mathrm{G}} \mathrm{d}V_{\mathrm{IG}} / \int_{V_{\mathrm{IG}}=0}^{V_{\mathrm{IG,peak}}} I_{\mathrm{G}} \mathrm{d}V_{\mathrm{IG}}$ is consistently ~ 1.5 for both. This suggests that the carrier density at the valley is most likely closer to ~ 1.5, slightly higher than the XPS-inferred value of 1.26.

We note that for this method to be useful, we need at least two well-defined doping states in the OECT transfer curve. A doping state is well-defined within this context if it was positioned between two regimes, e.g. the peak delinates Regimes I and II and the valley separates Regimes II and III. As a result, for the polymer systems studied here such a comparison can only be made in IDT-BT, where we could use the peak as the first well-defined state, and the valley as the second. We have not been able to provide an equivalent analysis for the carrier density in IDT-BT's Regime III due to the lack of a well-defined state across the second rise. Likewise, in DPP-BTz the presence of a peak in between of Regimes I and II is not enough for this method of carrier density analysis to be employed.

3.6 Cyclic voltammetry

Cyclic voltammetry (CV) measurements provide further support for the band filling interpretation. As shown in Extended Data Figure 3, the trends of conductivity versus oxidation potential for different polymers can be well correlated with the shapes of the CV curves.

For PBTTT, we only observed one reversible redox feature, and the conductivity increases with oxidation potential until irreversible degradation occurs. In DPP-BTz, we observed two reversible redox features, with the maximum conductivity occurring between the two waves and very low conductivity observed at potentials above the second oxidation process.

In IDT-BT, the shape of the cyclic voltammogram is the most complicated. When measuring at a scan rate of 10 mV s^{-1} we first observed two overlapping redox features. These can however be distinguished at

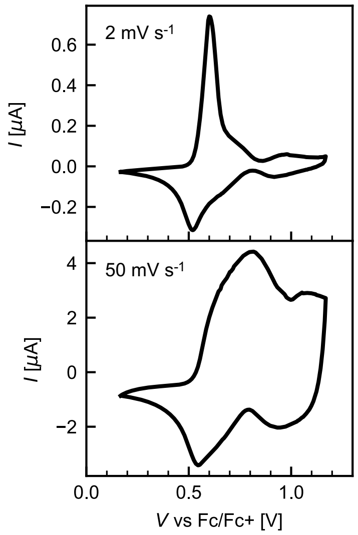

Supplementary Figure 8: **Scan rate dependence of the cyclic voltammogram.** (top panel) a well resolved IDT-BT cyclic voltammogram taken at 2 mV s^{-1}, and (bottom panel) a broad feature in cyclic voltammogram taken at 50 mV s^{-1}.

very slow sweep rate. As shown in Extended Data Figure 3 (reprinted in the top panel of Supplementary Figure 8 for convenience) at a scan rate of 2 mV s^{-1}, close to 0.7 V vs Fc/Fc^+ we observed a stronger peak at lower potential and a second, shoulder-like feature at higher potential; in contrast, at 50 mV s^{-1}, we only observe a very broad feature. The conductivity peak occurs in between these two redox waves, which is consistent with our picture of maximum conduction near half-band filling. At a higher potential we observe a third feature in IDT-BT, which corresponds to the onset of Regime III in the transfer curve. We attribute these three redox features to the three transport regimes in IDT-BT.

3.7 *Operando* UV-Vis and Infrared Spectroscopy on IDT-BT OECTs

Operando spectroscopic measurements of OECTs are also consistent with band filling. Extended Data Figure 4(a) shows optical absorption spectra throughout the ultraviolet-visible-near infrared (UV-Vis-NIR) of a device gated through each regime. In Regime I (blue lines), the π-π^* band at 2 eV bleaches with increasing doping due to the removal of charge from the HOMO band. This bleaching reaches completion exactly at the conductivity peak (see I-V curve inset), consistent with the removal of one electron per repeat unit. Beyond this doping level, no significant spectral changes are visible above 1 eV. FTIR measurements (Extended Data Figure 4(b)) show a corresponding growth in the P1 polaron band ($\sim$ 0.4 eV) [17] up to the conductivity peak. Beyond the peak we observe a clear isosbestic point near 0.3 eV, indicating that gating beyond the peak does not increase the total polaronic absorption but rather changes the nature of these states. In each regime we also observe systematic shifts in the position of the polaron band peak. In Regime I, the peak position red shifts with increasing $|V_{\mathrm{IG}}|$, indicating that polarons are becoming more delocalized, then blue shifts in Regime II indicating increasing localization. Finally in Regime III, the peak position red shifts again, suggesting that polarons again become more delocalized. This interpretation in terms of polaron delocalization is also consistent with the observed spectral changes of the so-called A peak around 0.15 eV. A strong A peak is a signature of a delocalized polaron [18] and we observe that the A peak becomes less intense in Regime II and regains intensity in Regime III. These trends follow those of the device conductivity, consistent with our previous work showing that at these high doping levels carrier delocalization is strongly correlated with conductivity.

3.8 Electron spin resonance (ESR) spectroscopy

3.8.1 Paramagnetic susceptibility analysis

We determined the susceptibility χ from the double-integrated area of the ESR peak. χ initially increases with increasing doping in Regime I, as paramagnetic charge carriers with $S = 1/2$ are introduced (Extended Data Figure 5(b)). It reaches a peak before the peak in conductivity, which indicates the onset of spin pairing as bi- and multi-polaron states are being formed.[19] We had expected a second rise of χ, as we enter Regime

III, instead we only observe that the reduction in χ slows down as we cross the point at which the valley in the conductivity occurs. This could potentially be explained by an overlap in the distribution of HOMO and HOMO-1 levels, i.e., the HOMO states not fully having been emptied everywhere yet, when the first holes are introduced into the HOMO-1 states.

From variable temperature measurements (290 K to 30 K) we separated the magnetic susceptibility into two contributions of comparable magnitude:[19, 20] a temperature dependent Curie contribution (χ_C) from localized spins, potentially residing in disordered regions of the film, and a temperature independent Pauli contribution (χ_P) from metallic spins (Extended Data Figure 5(c, d)) forming in the more ordered domains. χ_C is the dominant contribution at low doping levels and peaks before the conductivity reaches its maximum. It decreases monotonically from there onwards, although we detect a clear χ_C contribution up to the highest gate voltages applied. This indicates that full spin pairing of these Curie spins is hindered even at the highest doping levels, which could be a consequence of strong on-site Coulomb repulsions. Alternatively, the incomplete spin pairing could result from heterogeneity in the microstructure, i.e. the amorphous regions of the polymers may sterically hinder further insertion of dopant ions. χ_P on the other hand is the dominant contribution near the conductivity peak and drops significantly as we approach the valley. This is consistent with the interpretation of χ_P as a measure of the DOS at the Fermi level experienced by metallic spins. However, we note that the peak in conductivity is not reached exactly when the metallic spins experience the maximum DOS at E_F, but only at slightly higher doping level. This could again be a manifestation of the heterogeneity of the microstructure: the maximum conductivity is only reached when also the more disordered regions of the film that connect the metallic domains become sufficiently highly doped to support efficient charge transport.

3.8.2 Ion gate voltage correction

In order to account for threshold voltage drifts after variable temperature measurements, an ion gate voltage correction routine was applied. This involved taking first a full gate voltage scan at 290 K and measuring the four different parameters, T_1, T_2, magnetic susceptibility and conductivity. We then conducted temperature sweeps at different applied gate voltages. To correct for any variations in threshold voltage we took the 290 K parameters from each temperature sweep and plotted them not at the actual applied gate voltage, but attempted to find the set of parameters from the initial gate voltage scan that most closely matched the 290 K parameter values from the temperature sweep. The corresponding gate voltage was used for the correction. This involved calculating the absolute magnitude d_n of the differences in the values of each of the four parameters ($n = 1 - 4$) and minimising the cost function $y = \sum_n d_n$; the corrected gate voltages are presented as the best fit values (i.e. least residuals) $\pm$ the mean absolute error ($\sum_n d_n/n$). Equal weighting was applied to all parameters even though we acknowledge this may not be the case. For example, any slight degradation of the polymer could have significantly different effects on the aforementioned parameters as conductivity is a bulk measurement contrasting the ESR parameters which represent a local probe to the system. We also acknowledge that if the temperature sweeps are initiated before an equilibrium is achieved in the doping state, this will inevitably systematically underestimate the correct gate voltage. In order to mitigate this, the gate current was monitored during the voltage hold needed to dope the film and the measurement was only started after a 2 hour bias, for which we observe a current change of less than 1% for each experiment.

Supplementary Note 4 *In-operando* microstructural analysis

Grazing incidence wide-angle X-ray scattering (GIWAXS) was used to study the microstructural evolution as a function of the applied ion gate voltage. The microstructure was probed *in operando* as a function of doping level to detect any potential reduction in order at high ion loadings, which is an alternative hypothesis for the drop in the sample conductivity in Regime II of IDT-BT and DPP-BTz (though not the rise in Regime III).

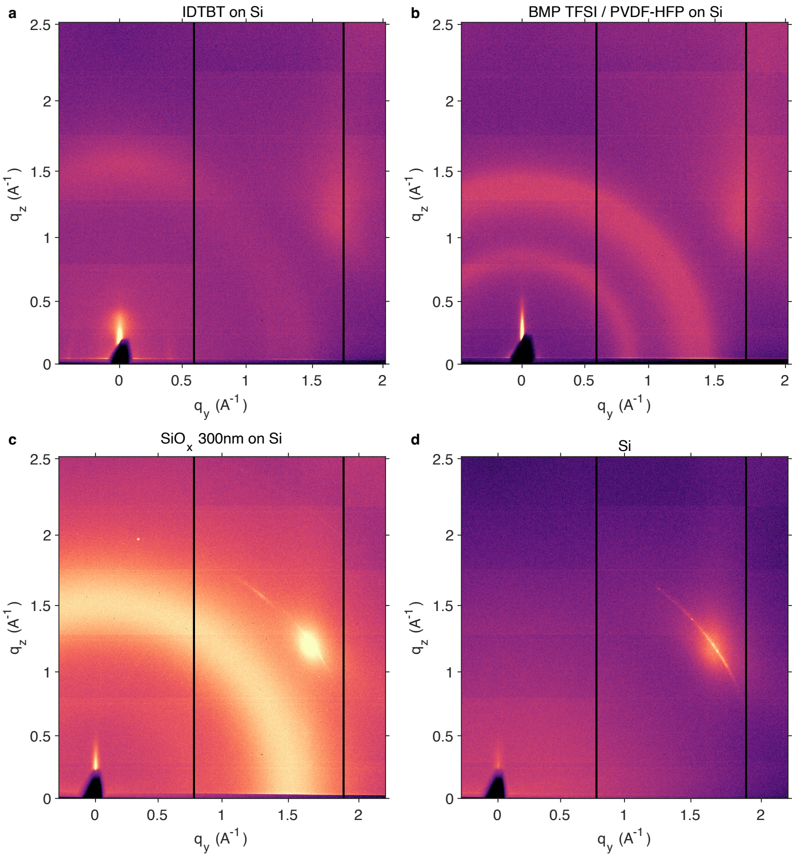

Supplementary Figure 9: **GIWAXS reference samples.** (a) Undoped IDT-BT thin film on bare Si. (b) BMP TFSI / PVDF-HFP ion gel on bare Si. (c) SiO_x/Si wafer (300 nm oxide layer). (d) Bare Si wafer (native oxide). All diffraction patterns were collected under identical exposure conditions and incidence angle, and are plotted with the same logarithmic color scale.

Supplementary Figure 9 shows GIWAXS patterns of IDT-BT and the BMP TFSI / PVDF-HFP ion gel on native oxide Si substrates, as well as GIWAXS patterns of an uncoated 300 nm SiO_x substrate, as used in the *operando* measurements, and the native oxide Si substrate for reference measurements. All films shown in Supplementary Figure 9 were spincoated under identical conditions to the devices, and are plotted with the same color scale, therefore the relative intensities correspond to the expected signal from each component in device measurements. Clearly, the background signal of the ion gel (Supplementary Figure 9(b)) and the SiO_x oxide layer (Supplementary Figure 9(c)) show scattering intensity similar or somewhat larger than that of the polymer itself. Therefore, in the following we primarily focus on the changes in scattering intensity during gating, rather than attempting to reconstruct the full 2D scattering pattern of the polymer by subtracting off background contributions.

In addition, cumulative beam damage to the device during repeated measurements limits the signal to noise ratio of our *in-operando* measurements. For each polymer, we measured multiple devices under different exposure conditions in an attempt to optimize the measurement. Beam damage was assessed by conducting hysteresis scans and monitoring for discontinuities in the drain and gate currents. In general, a drop in drain current on the order of a percent per measurement was deemed an acceptable compromise, although at very high doping levels we observed that beam damage was inevitably larger than at lower doping levels. These limitations of course limit the detail with which we can analyze the microstructure of our materials, however we stress that the aim of these measurements is to support our understanding of band filling behavior in these systems, rather than to perform a detailed structure-property analysis.

4.1 *In-operando* GIWAXS measurements of IDT-BT

Supplementary Figure 10 shows raw data for an IDT-BT ion gel gated device measured *in operando*. The device was gated at −2.3 V with a gate current compliance of 600 nA; measurements were taken at a relatively small 0.15° incidence angle to maximize the interaction volume with the film due to the relatively weak scattering from IDT-BT. GIWAXS measurements were taken every 300 s during gating, with each measurement corresponding to two 3 s exposures at 9.9% of full beam intensity. These exposure conditions correspond to a balance between minimizing beam damage, while maintaining enough scattering intensity to allow for a useful microstructral analysis.

By eye, there is almost no visible change in the raw GIWAXS data shown in Supplementary Figure 10. This implies that only relatively small changes in crystallinity occur during gating, consistent with our interpretation of the non-monotonic drain current in ion gated IDT-BT originating from band filling. To visualize the changes in scattering more clearly, we calculated difference images from the $t = 0$ s data, as shown in Supplementary Figure 11. These images are plotted as percentage change in pixel intensity on a linear scale (i.e. $(I(t) - I(0))/I(0))$). From these difference images, we can see a clear enhancement of the out-of-plane stacking peak at 0.3 Å^{-1}, previously assigned as the (200) lamellar stacking peak.[21, 22] In addition, a weak isotropic signal increase of $\sim 5\%$ around 1.6 Å^{-1}, and a corresponding signal decrease at 0.8 Å^{-1} are visible, although these appear to increase monotonically through the measurement and likely result from beam damage to the ion gel.

Supplementary Figure 12 shows linecuts taken from the diffraction images shown in Supplementary Figure 10. The in-plane linecuts (Supplementary Figure 12(a)) are taken just above the scattering horizon ($0.03 < q_y < 0.06$) Å^{-1}, while the out of plane linecuts (Supplementary Figure 12(b)) are shifted slightly off-center ($0.05 < q_z < 0.1$) Å^{-1} to better separate the weak scattering signal in IDT-BT from the background specular reflection. Again, little is visible aside from the enhancement of the (200) peak ($q_z \sim 0.25$ Å^{-1}) upon gating. To better visualize the effects of gating, we again plot difference data (Supplementary Figure 12(c, d)). Again, in-plane we see almost no changes, apart from the presumed ion gel beam damage discussed above, visible as a reduction in scattering intensity at $q_y < 1$ Å^{-1} and enhancement in intensity at $q_y \sim 1.6$ Å^{-1} of $\sim 5\%$. Out-of-plane, we again see the strongly enhanced (200) peak as discussed above, with no other significant changes visible.

To more quantitatively evaluate changes in the polymer microstructure upon gating, we fit the in-plane and out-of-plane linecuts, as shown in Supplementary Figure 13. Supplementary Figure 13(a) shows the out-of-plane fit. The (200) and (010) peaks are fit by Lorentzian functions, assuming paracrystalline-dominant peak broadening typical for disordered conjugated polymers.[23] To account for the ion gel and SiO_x background, we use linecuts from the reference samples shown in Supplementary Figure 9 which are allowed to vary by arbitrary scaling factors. We allow a negative contribution from the bare Si linecut as both the ion gel and SiO_x backgrounds contain contributions from the underlying Si wafer. These background scaling factors were constrained for all measurements to the values obtained from fitting using the $t = 0$ linecut (shown in Supplementary Figure 13(a)). The scaling factors obtained from this fit were close to those expected based on exposure times (3 seconds for *in-operando* measurements, 1 second for reference samples). In-plane, only the (001) peak is of interest, therefore we fit the data using a simple Lorentzian function with linear background over ($0.3 < q_y < 0.5$) Å^{-1}, which was allowed to vary for each fit.

The results of the peak fits are shown in Supplementary Figure 13(d-f); *in-situ* electrical measurements are shown in Supplementary Figure 13(c) for reference. Supplementary Figure 13(d) shows the fit parameters of the lamellar stacking (200) peak ($q_z \sim 0.3$ Å^{-1}) vs. measurement time. In addition to the $\sim$200% increase in signal intensity (bottom subpanel) visible by eye in the difference data above, we also see a slight expansion of the stacking distance from 20.8 to 22.2 Å (top subpanel), consistent with the incorporation of dopant ions into the lamellar regions. Peak width (middle subpanel) is approximately unchanged, increasing slightly at later times perhaps due to slight beam damage during cumulative measurements. Similar systematic changes with gating are also visible in the (001) backbone repeat peak (Supplementary Figure 13(f)). We observe a decrease in the (001) stacking distance upon gating. This contraction is expected: polaron formation results in a change in the backbone electronic structure from a benzylic structure, where the double bonds are largely normal to the backbone repeat direction, to a quinodal type structure, where the double bonds lie along the backbone repeat direction. This increase in bond order along the backbone should lead to a contraction in stacking distance, along with further planarization of the backbone due to the formation of double bonds between each IDT and BT units. This planarization is visible as a decrease in the peak width (i.e. an increase in coherence length) of the (001) peak (Supplementary Figure 13(f), middle panel) although the low intensity of this feature leads to relatively large error bars and limits further analysis. As in the (200) peak, we observe a decrease in peak intensity over the course of the measurement due to beam damage. Lastly, Supplementary Figure 13(e) shows the corresponding fit results for the π-stacking peak (010), also obtained

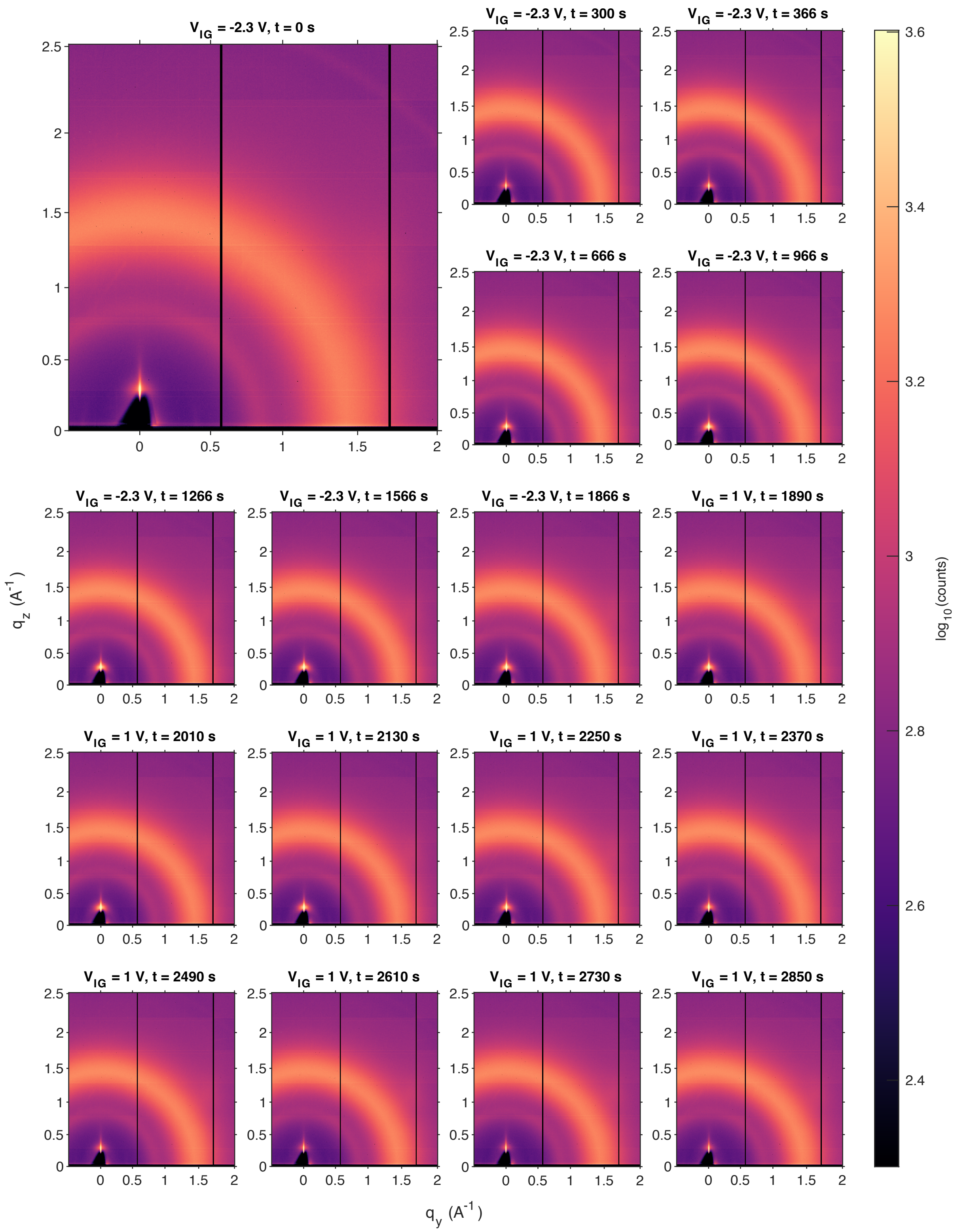

Supplementary Figure 10: **IDT-BT in-situ ion gated GIWAXS raw data**. Data is plotted in log scale. From $t = 0$ to $t = 1{,}866$ s the device is being gated on at -2.3 V; from $t = 1{,}890$ s onwards the device is being gated off at +1 V. $V_D = -0.1$ V during the entire experiment.

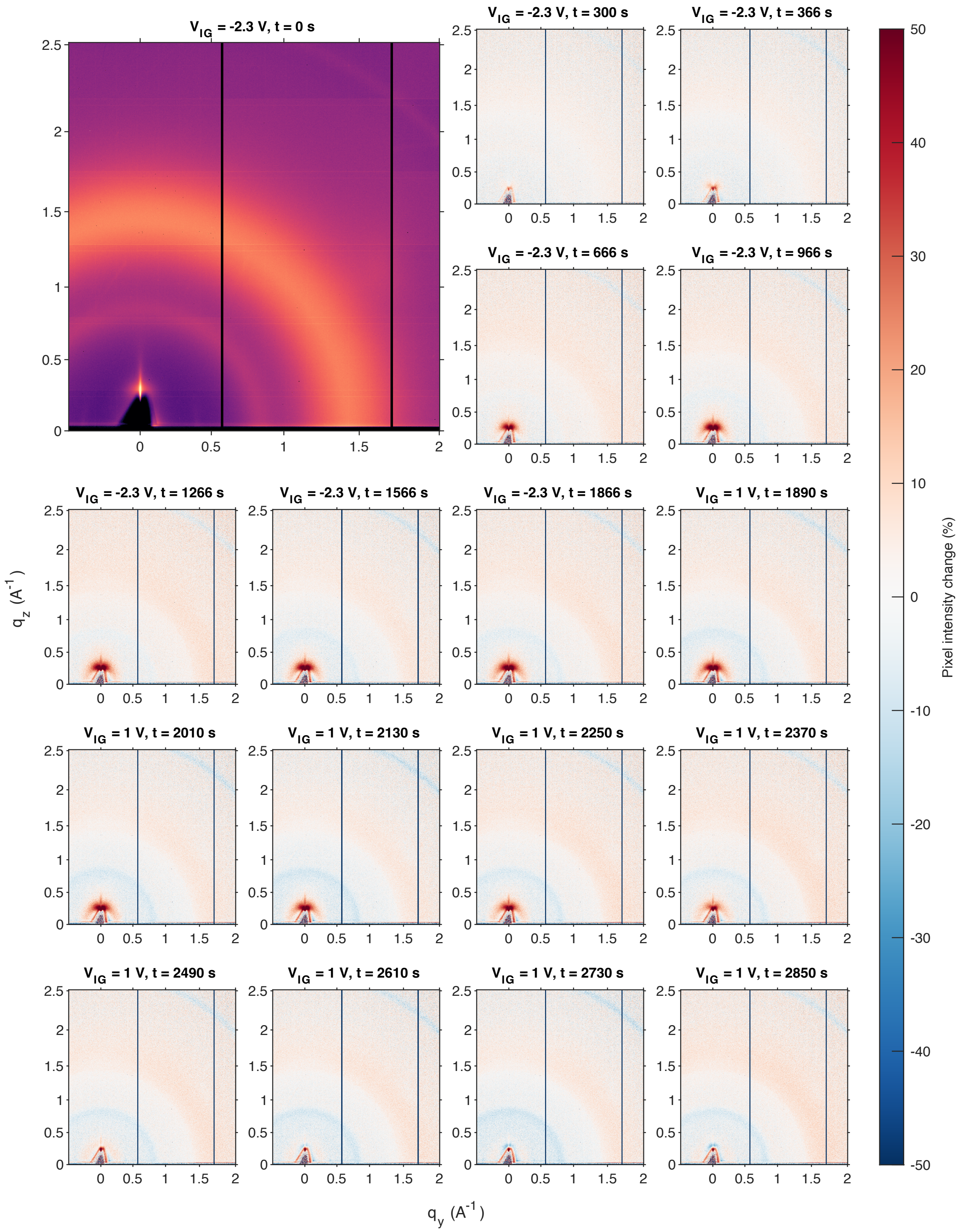

Supplementary Figure 11: **IDT-BT in-situ ion gated GIWAXS difference images**. Raw data is shown for $t = 0$ s, upper left; other images show the difference from $t = 0$ s as percentage change in pixel intensity on a linear scale (i.e. $(I(t) - I(0))/I(0)$). Red areas indicate increased intensity; blue areas indicate decreased intensity.

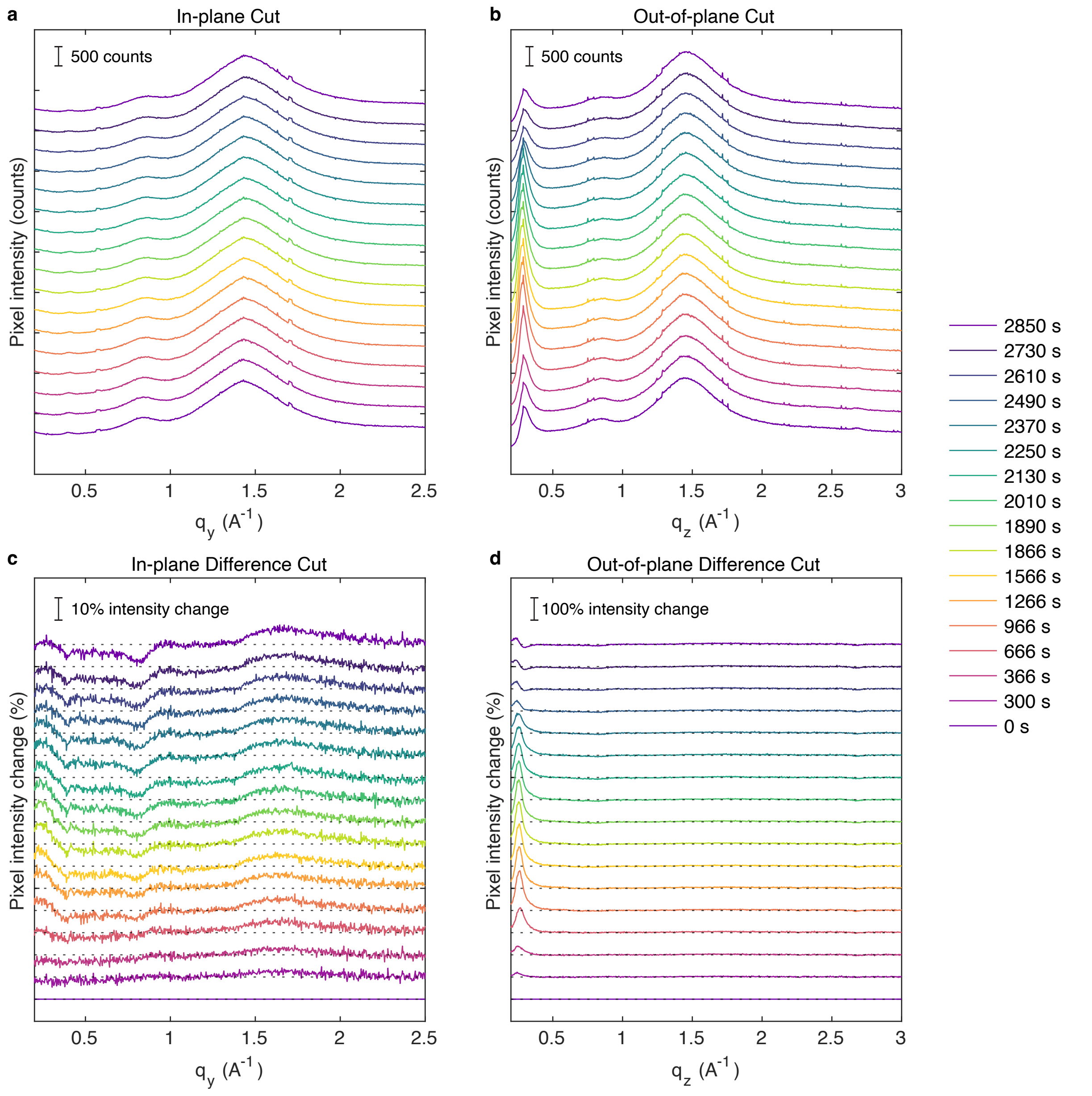

Supplementary Figure 12: **IDT-BT in-situ ion gated GIWAXS linecuts**. (a) In-plane linecut ($0.03 < q_y < 0.06$) Å^{-1}. (b) Out-of-plane linecut ($0.05 < q_z < 0.1$) Å^{-1}. (c) In-plane difference linecut from $t = 0$ s. (d) Out-of-plane difference linecut from $t = 0$ s. Dashed lines in panels (c, d) indicate 0 intensity change baseline for each linecut.

from the out-of-plane fits. We observe no obvious changes to stacking distance, peak width, or peak intensity.

Notably, the trends in both intensity and stacking distance for both (200) and (001) are well-correlated, but do not show the same non-monotonic behavior seen in the electrical measurements (Supplementary Figure 13(c)). Instead, both stacking distance and intensity increase steadily up to the peak (half-band filling), then remain essentially constant, following the same trend upon discharge. These observations are consistent with a reversible structural change upon doping and dedoping. Most importantly, these results clearly indicate that the non-monotonic behavior seen upon ion gating do not result from systematic changes in film disorder as doping level is varied.

4.2 *In-operando* GIWAXS measurements of DPP-BTz

Supplementary Figure 14 shows raw data for a DPP-BTz device gated at −2.2 V with a gate current compliance of 400 nA; measurements were taken at a relatively small 0.15° incidence angle to maximize the interaction volume with the film. GIWAXS measurements were taken every 300 s during gating, with each measurement corresponding to two 5 s exposures at 0.052% of full beam intensity. For the dedoping scan, the device was gated at 1 V with a gate compliance of 300 nA. An accidental change in measurement conditions

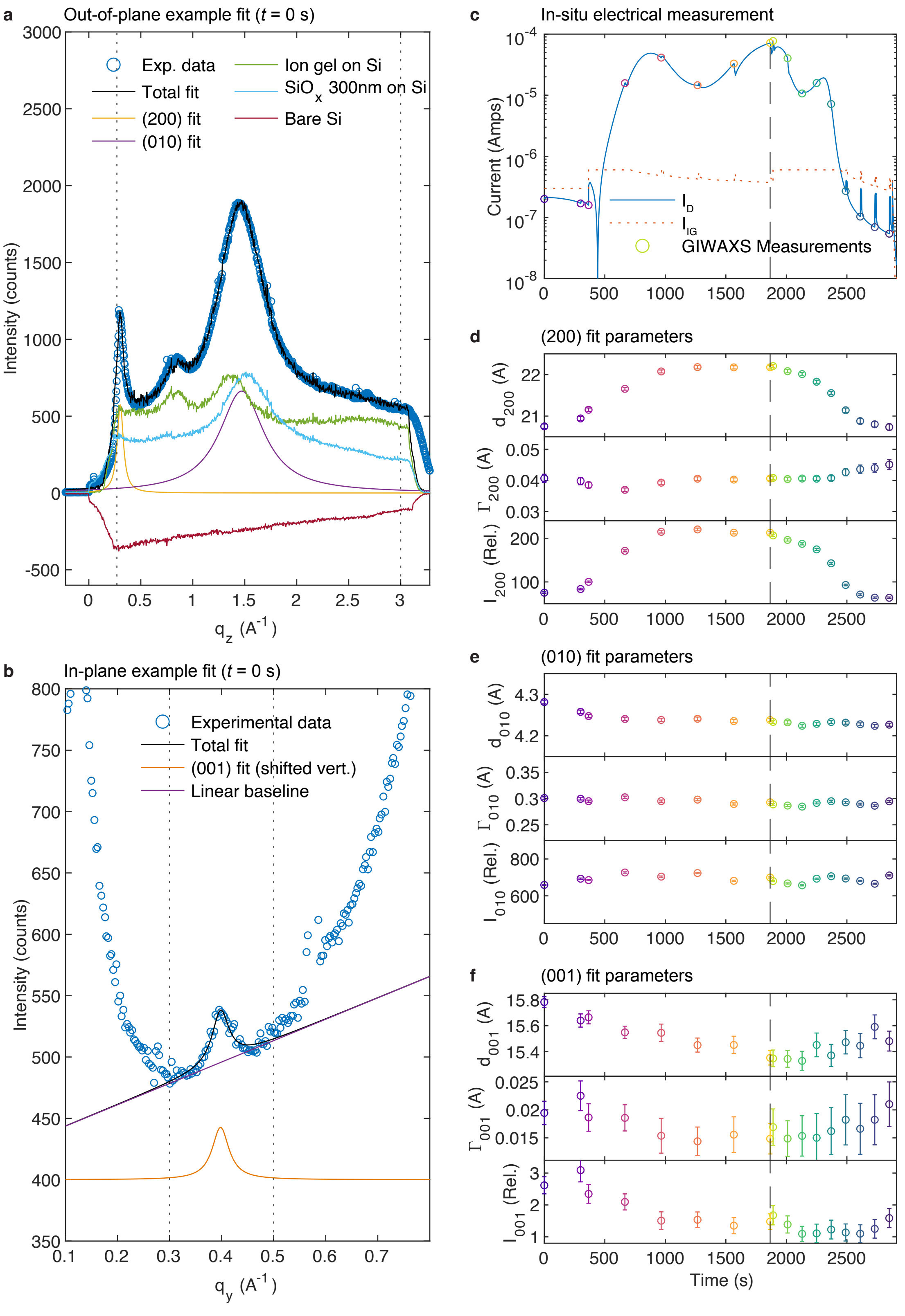

Supplementary Figure 13: **IDT-BT in-situ ion gated GIWAXS peak fitting**. (a) Example out-of-plane linecut fit ($t = 0$ s shown). (b) Example in-plane linecut fit for (001) peak ($t = 0$ s shown). Vertical dashed lines indicate range of data used for fitting. (c) *In-situ* electrical measurement; circles indicate GIWAXS measurements, color code corresponds to those in Supplementary Figure 12. Dashed vertical line in (c-f) indicates the time when the gate voltage was switched from -2.3 V to $+1$ V. (d) (100) stacking distance (top) peak width (middle) and peak amplitude (bottom) from out-of-plane linecut fits. (e) (010) stacking distance (top) peak width (middle) and peak amplitude (bottom) from out-of-plane linecut fits. (f) (001) stacking distance (top) peak width (middle) and peak amplitude (bottom) from in-plane linecut fits. Data in (d-f) are presented as best fit values with error bars representing the fit uncertainty as the standard error of the mean. Error bars for stacking distances reflect the random uncertainty of the fit only; there is an additional systematic error on the order of $w/2D \sim 3\%$ (where w is the channel width and D is the sample-detector distance).

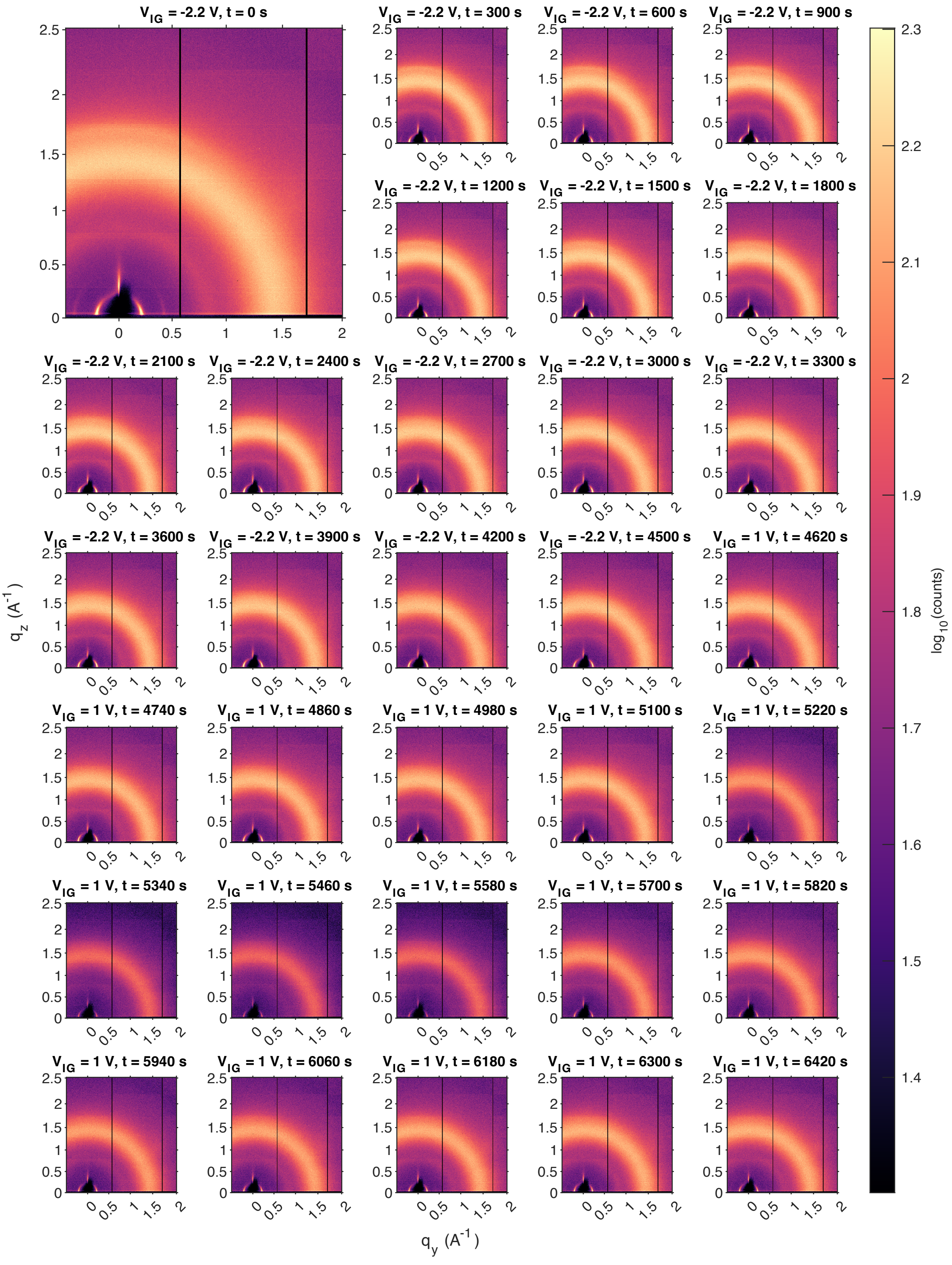

Supplementary Figure 14: **DPP-BTz in-situ ion gated GIWAXS raw data**. Data is plotted in log scale. From $t = 0$ to $t = 4{,}500$ s the device is being gated on at -2.2 V; from $t = 4{,}620$ s onwards the device is being gated off at +1 V. $V_{\mathrm{D}} = -0.1$ V during the entire experiment. A fluctuation in sample or beam position occurred between $t = 5{,}220$ s and $t = 5{,}820$ s leading to a decrease in signal intensity; at later times the signal intensity mostly recovered.

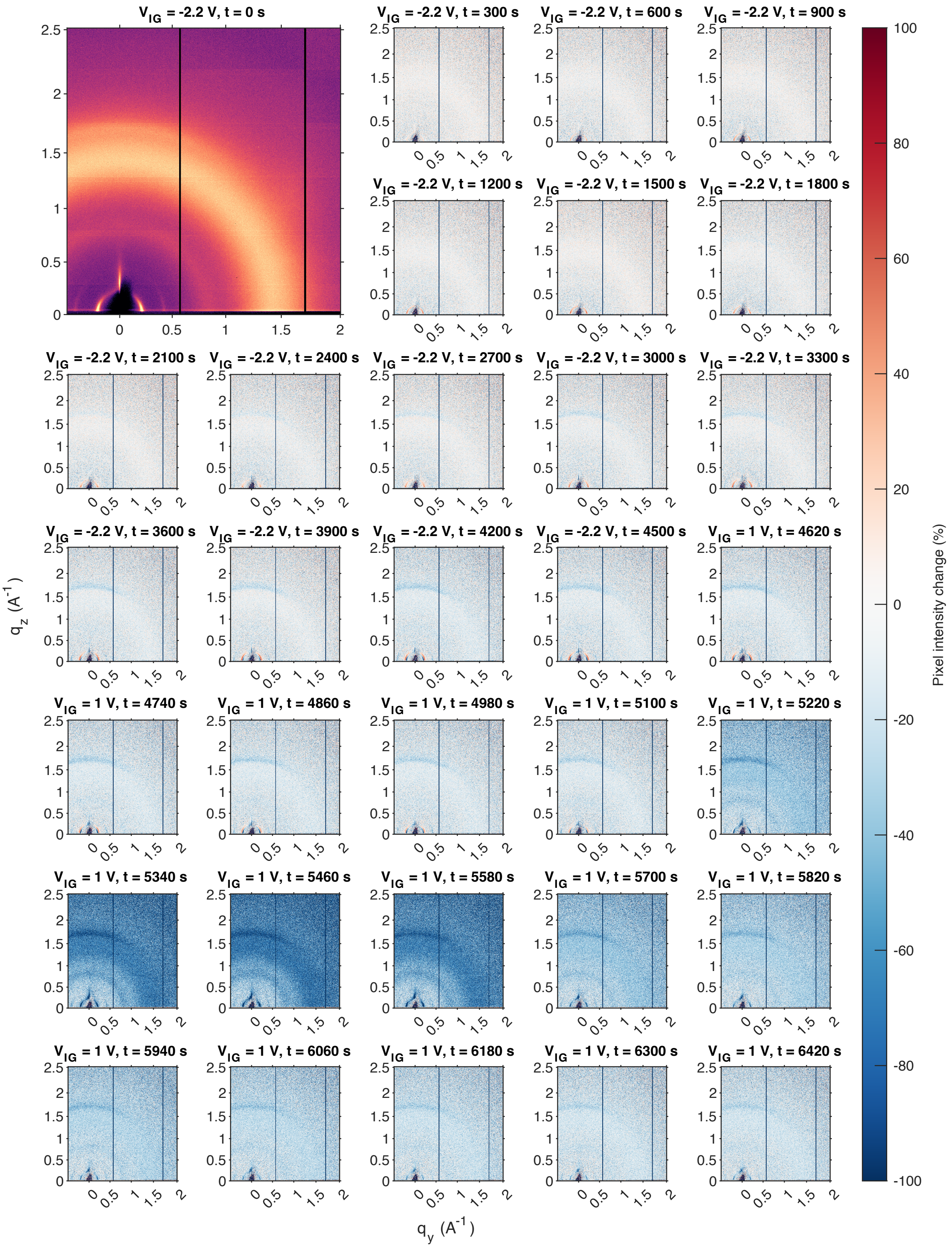

Supplementary Figure 15: **DPP-BTz in-situ ion gated GIWAXS difference images**. Raw data is shown for $t = 0$ s, upper left; other images show the difference from $t = 0$ s as percentage change in pixel intensity on a linear scale (i.e. $(I(t) - I(0))/I(0)$). Red areas indicate increased intensity; blue areas indicate decreased intensity. A fluctuation in sample or beam position occurred between $t =$ 5,220 s and $t =$ 5,820 s leading to a decrease in signal intensity; at later times the signal intensity mostly recovered.

led to a decrease in signal intensity near the end of the experiment. In particular, data at 5,340, 5,460, and 5,580 s showed around 40% lower intensity than preceding measurements, while all following measurements were reduced in intensity by about 10%. As described below, this reduced the fit quality somewhat, but still allows us to perform a semi-quantitative analysis.

Supplementary Figure 15 shows difference images from $t = 0$ s. We observe changes in the in-plane (100) peak at $q_y = 0.2$ Å^{-1}, corresponding to the lamellar stacking direction of the polymer, as well as a shift in the out-of-π-stacking peak at $q_z = 1.7$ Å^{-1}. The loss in signal intensity during the dedoping scan (5,220 s onwards) is also clearly visible.

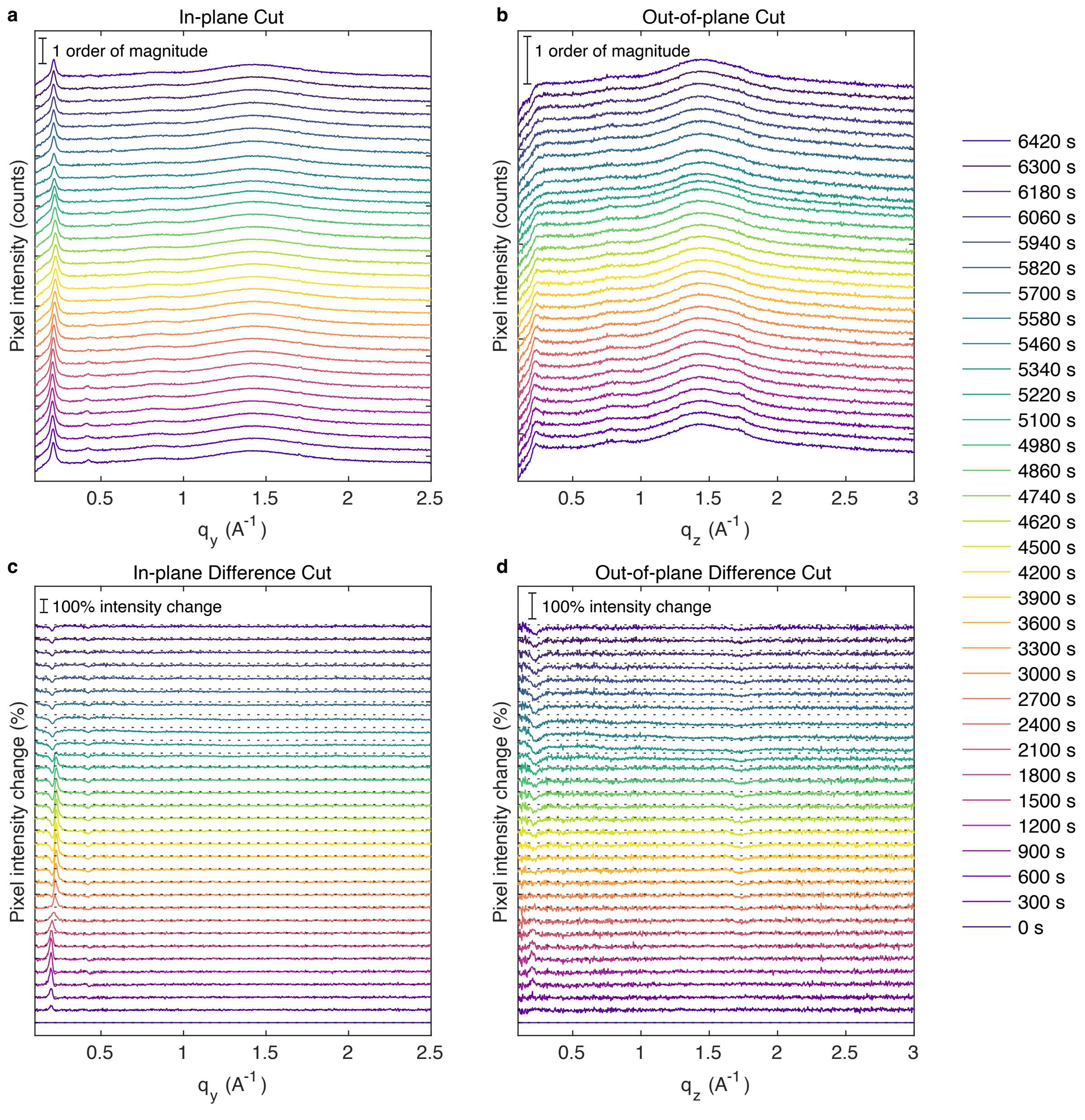

Supplementary Figure 16: **DPP-BTz in-situ ion gated GIWAXS linecuts**. (a) In-plane linecut ($-0.06 < q_y < -0.03$) Å^{-1} plotted on a logarithmic intensity scale. (b) Out-of-plane linecut ($0.03 < q_z < 0.06$) Å^{-1} plotted on a logarithmic intensity scale. (c) In-plane difference linecut from $t = 0$ s. (d) Out-of-plane difference linecut from $t = 0$ s. Dashed lines in (c, d) indicate 0 intensity change baseline for each linecut.

Supplementary Figure 16 shows linecuts taken from the diffraction images shown in Supplementary Figure 14. The in-plane linecuts (Supplementary Figure 16(a)) are taken just above the scattering horizon ($0.03 < q_y < 0.06$) Å^{-1}, while the out of plane linecuts (Supplementary Figure 16(b)) are shifted slightly off-center ($-0.06 < q_z < -0.03$) Å^{-1} to better reject the background specular reflection; both plots are shown in log scale. Again, to better visualize the effects of gating we also plot the difference from $t = 0$ s data (Supplementary Figure 12(c, d)). A clear increase in the in-plane (100) lamellar stacking peak ($q_y = 0.2$

Å^{-1}) upon gating is visible.

Mirroring our analysis of IDT-BT, we fit the *operando* in-plane and out-of-plane linecuts to obtain a more quantitative picture of the microstructural evolution of DPP-BTz upon ion gating. Supplementary Figure 17(a, b) shows example fits. We fit the out-of-plane linecut to the sum of two Lorentzian functions, corresponding to the π-stacking (010) peak ($q_z \sim 1.7$ Å^{-1}) and a disordered halo feature previously assigned to disordered alkyl side chains [2] ($q_z \sim 1.4$ Å^{-1}). The in-plane linecut (Supplementary Figure 17(b)) is fit to three Lorentzians, corresponding to the lamellar stacking (100) and (200) peaks and the aforementioned disordered alkyl halo. In both in-plane and out-of-plane fits, we use the same background components used in the IDT-BT analysis in Supplementary Note 4.1. Due to the fluctuation in signal intensity described above, in the in-plane fits the relative ratio of background components were fixed by the $t = 0$ s fit, but the total background intensity was allowed to vary for each fit. In the out-of-plane fits, we found it necessary to fully re-fit the background in each measurement to obtain good fits in the dedoping scan (above 5,220 s) in order to achieve convergence on the π-stacking peak. Due to these experimental artifacts, the extracted intensities above $t =$ 5,220 s should be treated as only semi-quantitative.

Supplementary Figure 17(c) shows the *in-situ* I-V measurement. We gated the device until the drain current reached approximately two orders of magnitude below the peak; upon dedoping, the peak drain current is slightly over half the value obtained in the forward sweep, similar to the measurements obtained in our lab (see Extended Data Figure 7). Therefore, under the quite low dose conditions used for this measurement (0.52% of the available beam power) beam damage does not appear to be a concern.

The (100) lamellar stacking peak (Supplementary Figure 17(d)) shows unusual behavior, to our knowledge not previously observed in an ion gated polymer. Initial gating to the peak leads to a slight expansion ($\sim$1 Å) of the lamella (top subpanel), consistent with insertion of ions into the side-chain region of the crystal structure. However, upon gating past the peak, we observe a reversal in this behaviour: the stacking distance contracts, eventually reaching a value ($\sim$1.5 Å) lower than the undoped polymer as we reach complete HOMO band filling. The peak width (middle subpanel) and intensity (bottom subpanel) show an increase in the intensity of the peak, along with a slight reduction in width, both correlating with an increase in lamellar ordering. However, the highest degree of ordering occurs at around 2,000 s, somewhat after the maximum in drain current and lamellar stacking distance (1,200 s). Beyond this point, we observe a decrease in intensity and an increase in peak width, both consistent with an increase in disorder, up to full band filling. Upon dedoping, the stacking distance follows the same trend observed during doping, although the maximum and fully dedoped stacking distances are both reduced by about 1 Å. Peak width follows a similar trend to doping, eventually reaching a value similar to $t = 0$ at complete dedoping. Peak intensity also appears to be increasing again at the beginning of the dedoping scan, however beyond 5,220 s the data is not reliable. The changes in stacking distance observed here are quite small in comparison to those previously seen in systems with more ordered lamellar stacking such as PBTTT, where an expansion of >6 Å upon doping occurs.[5]

Supplementary Figure 17(e) shows the fit to the π-stacking peak obtained from the out-of-plane fits. Up to the conductivity peak, we observe an essentially unchanged π-stacking distance (top subpanel), beyond the peak it increases steadily by about 0.2 Å up to full band filling. Upon dedoping, π-stacking distances recover to the undoped value, however peak intensity (bottom subpanel) decreases irrecoverably upon gating past the peak. Peak width, which is inversely proportional to paracrystallinity, does not appear to vary significantly over the measurement. However, due to the strong background signal and progressive weakening in peak intensity near full band filling and in the reverse scan, achieving convergence at all time steps was not possible.

These observations, together with the fits to the (100) peak above, suggest that upon gating past the peak, some ions may be pulled from the sidechain region into some of the π-stacking domains, leading to a reduction in (100) stacking distance and an increase in (010) stacking distance. However, the full reversibility of the (010) stacking distance but irreversible loss of intensity implies a heterogeneous doping mechanism, where some fraction of π-stacked crystallites are irreversibly lost during gating, but those that survive do not appear to suffer an appreciable increase in disorder. This reduction in π-stacking aggregates may be responsible for the reduction in peak drain current observed on the reverse scan, i.e., the hysteresis in the I-V characteristics.

Although the increase in π-stacking distance beyond the peak might suggest that the reduction in drain current is partly caused by dopant-induced structural disorder, it is similarly plausible that the changes in the electronic state of the π-system past the peak could lead to the observed structural changes. Upon doping, polaron delocalization is well-understood to lead to planarization of polymer backbones as well as a reduction in π-stacking distances.[24] In Regime I, before half band filling, polaron delocalization therefore drives an increase in crystalline order, while ion incorporation typically leads to a decrease in crystalline order. The observation of negligible changes in π-stacking, and an increase in lamellar stacking, suggests that up to the peak, attractive interactions from inter-chain polaron delocalization are sufficient to prevent incorporation of ions into π-stacks. However, beyond the peak, further doping leads to a *decrease* in polaron delocalization;

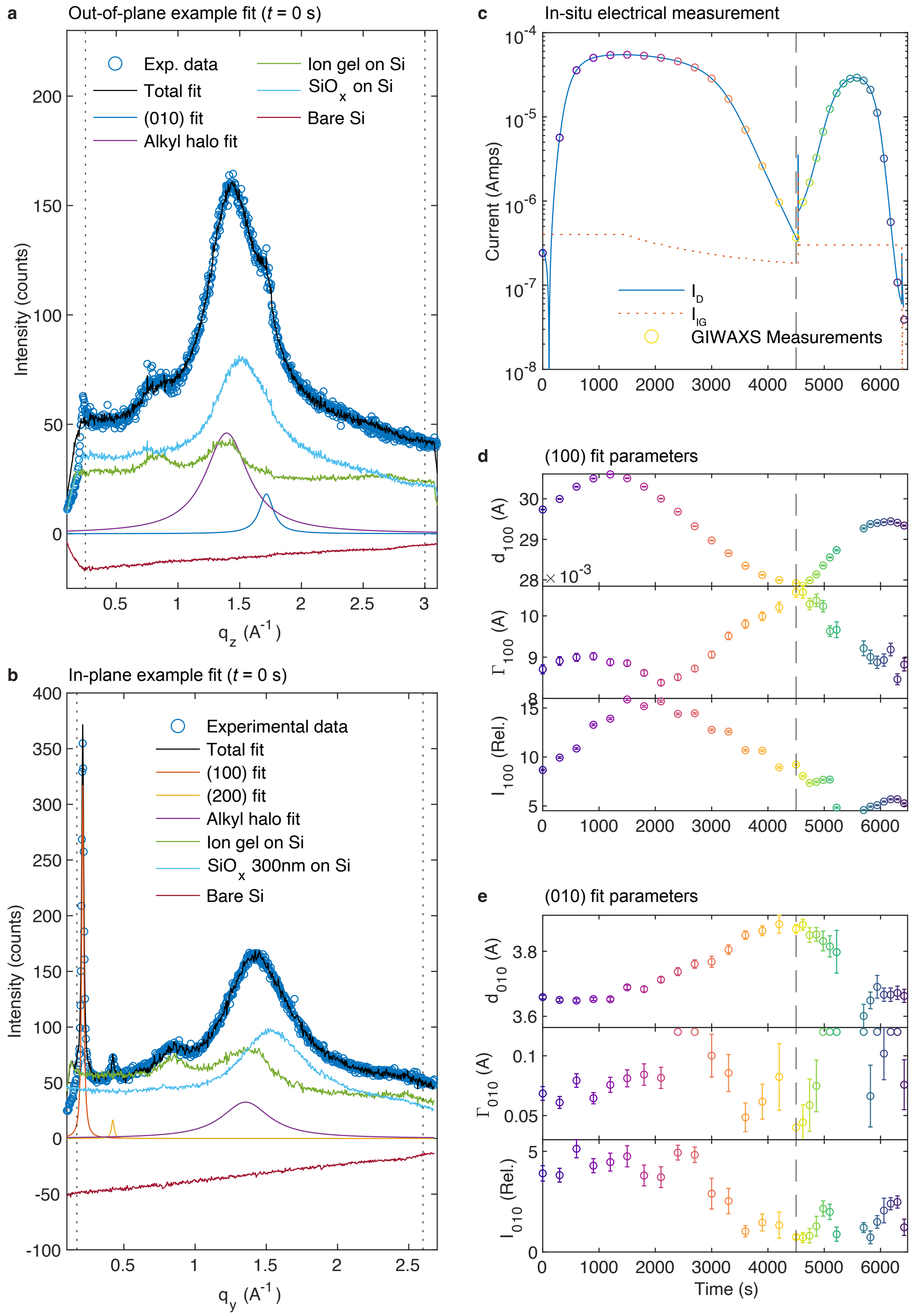

Supplementary Figure 17: **DPP-BTz in-situ ion gated GIWAXS peak fitting**. (a) Example out-of-plane linecut fit ($t = 0$ s shown). (b) Example in-plane linecut fit ($t = 0$ s shown). Vertical dashed lines indicate range of data used for fitting. (c) *In-situ* electrical measurement; circles indicate GIWAXS measurements, color code corresponds to those in Supplementary Figure 16. Dashed vertical line in (c-f) indicates the time when the gate voltage was switched from -2.2 V to $+1$ V. (d) (100) stacking distance (top) peak width (middle) and peak amplitude (bottom) from out-of-plane linecut fits. (e) (010) stacking distance (top) peak width (middle) and peak amplitude (bottom) from out-of-plane linecut fits. Data in (d-f) are presented as best fit values with error bars representing the fit uncertainty as the standard error of the mean.

further doping therefore weakens the attractive interactions preventing ion incorporation into the π-stacks. We expect that incorporation of ions into π-stacks is an autocatalytic process, similar to that previously observed for ion incorporation into crystalline lamellar regions.[5, 25] Once ions begin to intercalate into a π-stack, the increase in disorder leads to faster incorporation into this region, leading to an irreversible loss of only some π-aggregates. Upon dedoping, the process reverses, but only for π-aggregates which had little to no ion incorporation; aggregates which sustained significant ion intercalation presumably remain amorphous upon dedoping. In these heavily intercalated crystallites some ions remain in the π-stacking region during dedoping, leading to a reduction in average lamellar stacking distance on the dedoping scan. We therefore observe both reversible and irreversible structural changes that are connected with the observed band filling behavior, but appear to be *driven by changes in the electronic structure of the polymer*, rather than the opposite.

4.3 *In-operando* GIWAXS measurements of PBTTT

Supplementary Figure 18 shows raw data for a PBTTT device gated at -2.0 V with a gate current compliance of 300 nA; measurements were taken at a relatively small incidence angle (approx. 0.15°) to maximize the interaction volume with the film. GIWAXS measurements were taken every 300 s during gating, with each measurement corresponding to two 3 s exposures at 0.052% of full beam intensity. For the dedoping scan (Supplementary Figure 19), the device was gated at 1.5 V with a gate compliance of 300 nA. Supplementary Figure 20 and Supplementary Figure 21 show difference images from $t = 0$ s for the doping and dedoping scans, respectively. We observe a strong intensity enhancement and stacking distance contraction in the out-of -plane lamellar stacking direction, as well as a contraction in the out-of-plane π-stacking at $q_z = 1.7$ Å^{-1}, consistent with our previous report.[5] The same changes are clearly visible in the in-plane and out-of-plane linecuts (Supplementary Figure 22).

Pseudocolor plots of linecut intensity vs. time (Supplementary Figure 23) provide a clearer view of the time-evolution of the microstructural evolution. The in-plane data in Supplementary Figure 23(a, c) shows a clear shift in the π-stacking peak from about $q_y = 1.7$ Å^{-1} to $q_y = 1.8$ Å^{-1} between $t = 0$ and $t = 2{,}400$ s, with relatively little change in intensity or peak width (more detailed analysis from fitting is given in Supplementary Figure 24). However, the out-of-plane lamellar stacking peaks in Supplementary Figure 23(b, d) show clear evidence of a phase transition, as previously argued.[5] In particular, the (200) and (300) lamellar stacking peaks at $q_z = 0.7$ Å^{-1} and $q_z = 1.0$ Å^{-1}, respectively, first shift to lower q and broaden significantly, before being replaced by a new series of much narrower lamellar stacking peaks at $t = 2{,}400$ s. The clear discontinuity visible at $t = 2{,}400$ s indicates that as ions intercalate into the lamella, there is first a slight expansion and increase in disorder, followed by a rapid recrystallization into a new, more ordered phase.

Following the same analysis of IDT-BT and DPP-BTz above, we fit the *in-operando* in-plane and out-of-plane linecuts to obtain a more quantitative picture of the microstructural evolution upon ion gating. Supplementary Figure 24(a-c) shows example fits. We fit the out-of-plane linecut to the sum of five Lorentzian functions spaced by integer multiples, corresponding to the (h00) lamellar stacking peaks of PBTTT. The in-plane linecut (Supplementary Figure 17(b)) is fit to two Lorentzians, corresponding to the π-stacking (010) and backbone repeat (003) peaks.[3] In both the in-plane and out-of-plane fits, we use the same background components used in the IDT-BT analysis in Supplementary Note 4.1. Background contributions were fit for $t = 0$ spectra and held constant for all other time points. Measurements between $t = 5{,}400$ and 6,000 s were excluded from fitting due to a fluctuation in sample alignment.

Supplementary Figure 24(d) shows the *in-situ* I-V measurement. PBTTT:TFSI shows very high electrical conductivity (exceeding 1,000 S cm^{-1}[5]); at this conductivity, the very wide, short channel device necessitated for *in-operando* GIWAXS measurements provides to an underestimate of the true film conductivity. To obtain a more accurate measurement of film conductivity, we performed *in-operando* I-V measurements on both the main device channel as well as a separate, smaller channel (800 by 800 μm^2) placed just outside the main channel (see device structure in Supplementary Figure 1). The drain current of this smaller channel is reported in Supplementary Figure 24(d). We gated the device until the drain current had nearly plateaued, corresponding to a conductivity of approximately 500 S cm^{-1}. These values are similar to the measurements obtained in our lab (see Extended Data Figure 7), therefore, under the quite low dose conditions used for this measurement (0.52% of the available beam power) beam damage again does not appear to be a concern.

Supplementary Figure 24(e-i) show lamellar stacking (h00) fit parameters obtained from the out-of-plane linecut fits. Peak positions for higher order peaks were contrained to multiples of the (100) stacking distance, therefore Supplementary Figures 24(f-i) only show peak width and intensity. Consistent with previous reports of PBTTT:TFSI [5, 6, 13] we observe a significant expansion of the lamellar stacking distance from just under 20 Å to about 25 Å, indicative of intercalation of TFSI ions into the sidechain region. As discussed above, the incorporation of ions first leads to a slight expansion of the stacking distance by only about 1 Å, up to drain

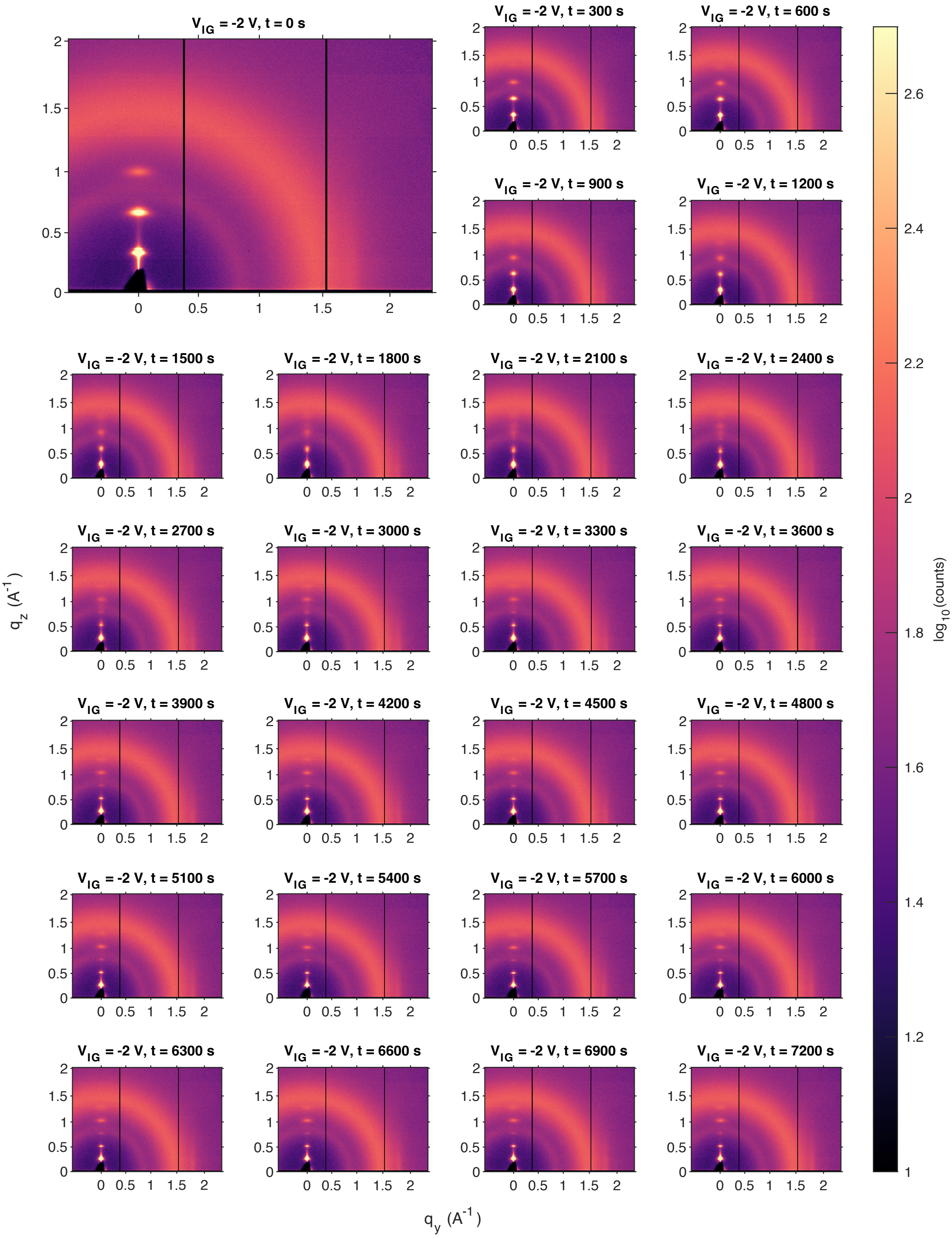

Supplementary Figure 18: **PBTTT in-situ ion gated GIWAXS raw data, doping scan**. Data is plotted in log scale. From $t = 0$ to $t = 7{,}200$ s the device is being gated on at -2 V; from $t = 7{,}320$ s onwards the device is being gated off at $+1.5$ V (see Supplementary Figure 19). $V_\mathrm{D} = -0.1$ V during the entire experiment.

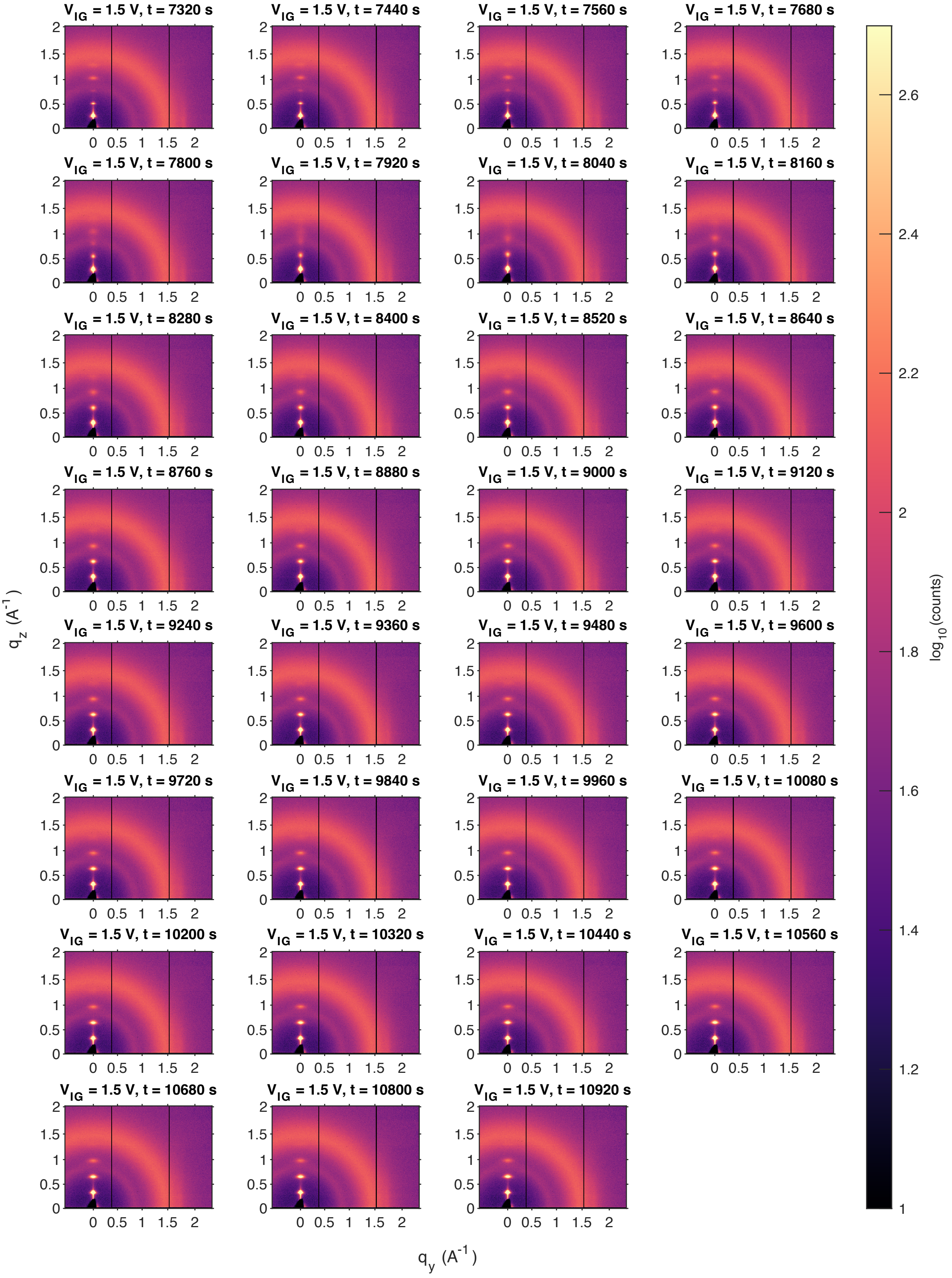

Supplementary Figure 19: **PBTTT in-situ ion gated GIWAXS raw data, dedoping scan**. Data is plotted in log scale. From $t = 0$ to $t = 7{,}200$ s the device was being gated on at -2 V (see Supplementary Figure 18); from $t = 7{,}320$ s onwards the device is being gated off at $+1.5$ V. $V_D = -0.1$ V during the entire experiment.

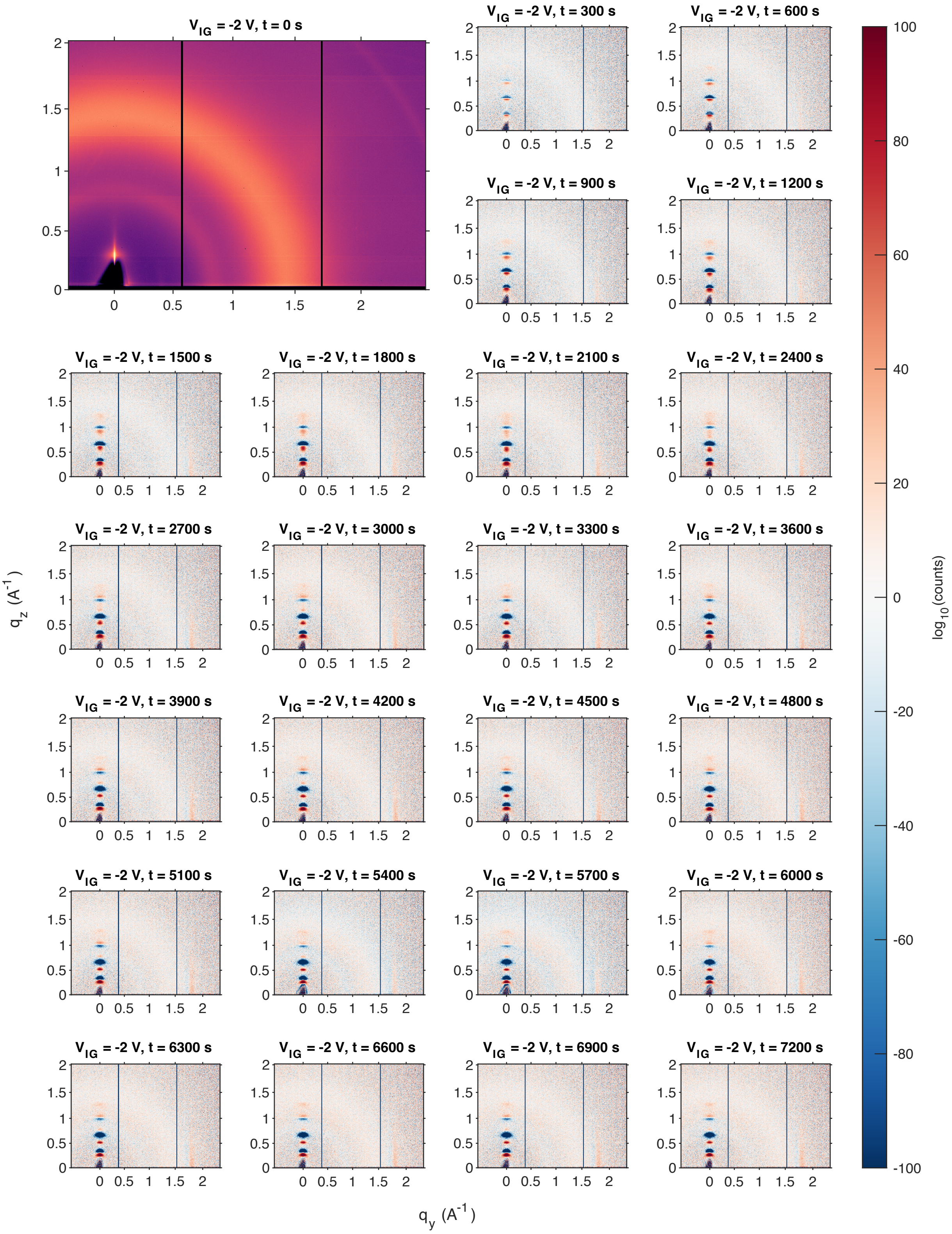

Supplementary Figure 20: **PBTTT in-situ ion gated GIWAXS difference images, doping scan**. Raw data is shown for $t = 0$ s, upper left; other images show the difference from $t = 0$ s as percentage change in pixel intensity on a linear scale (i.e. $(I(t) - I(0))/I(0)$). Red areas indicate increased intensity; blue areas indicate decreased intensity.

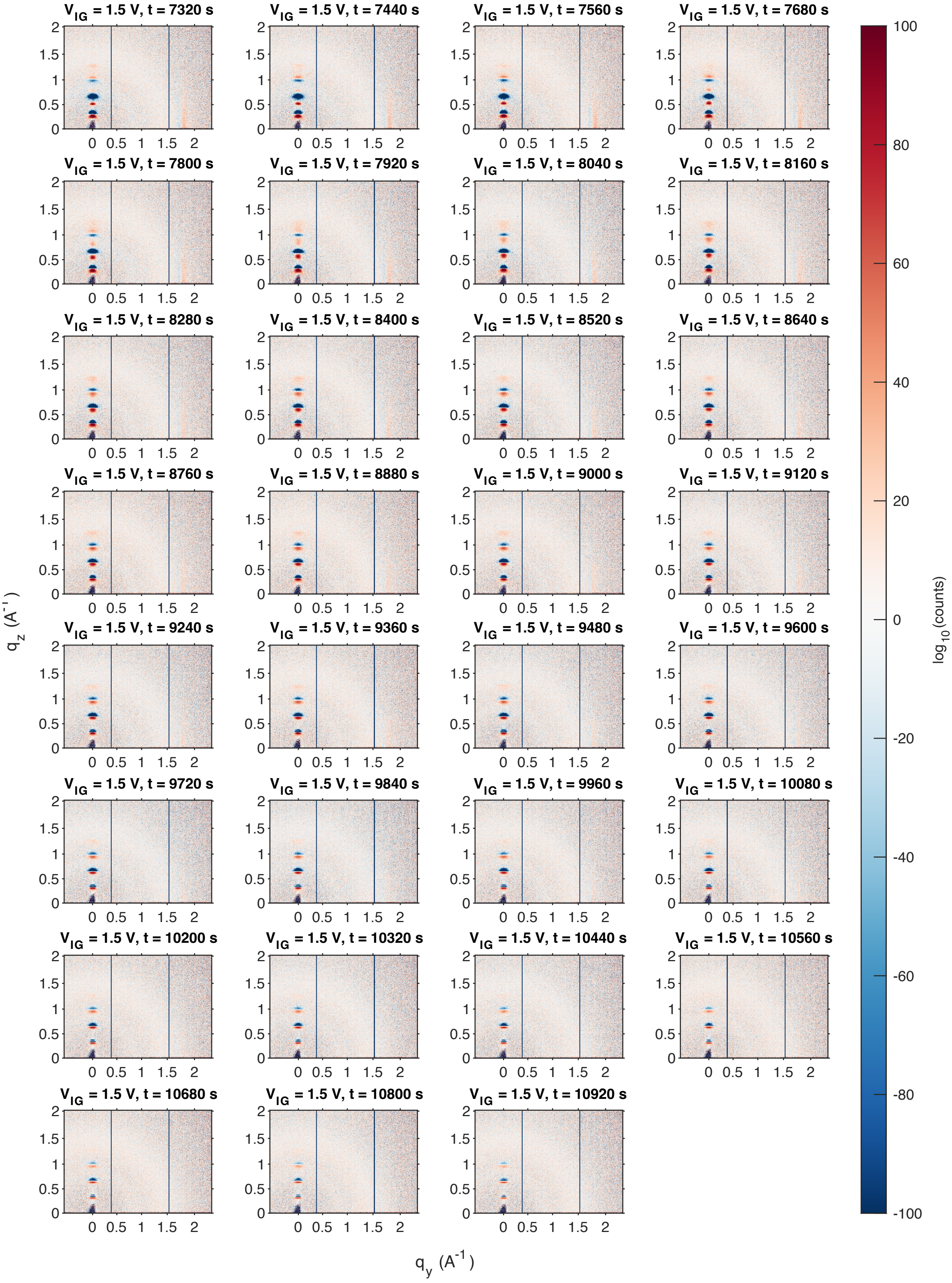

Supplementary Figure 21: **PBTTT in-situ ion gated GIWAXS difference images, dedoping scan**. Raw data is shown for $t = 0$ s, upper left; other images show the difference from $t = 0$ s as percentage change in pixel intensity on a linear scale (i.e. $(I(t) - I(0))/I(0)$). Red areas indicate increased intensity; blue areas indicate decreased intensity.

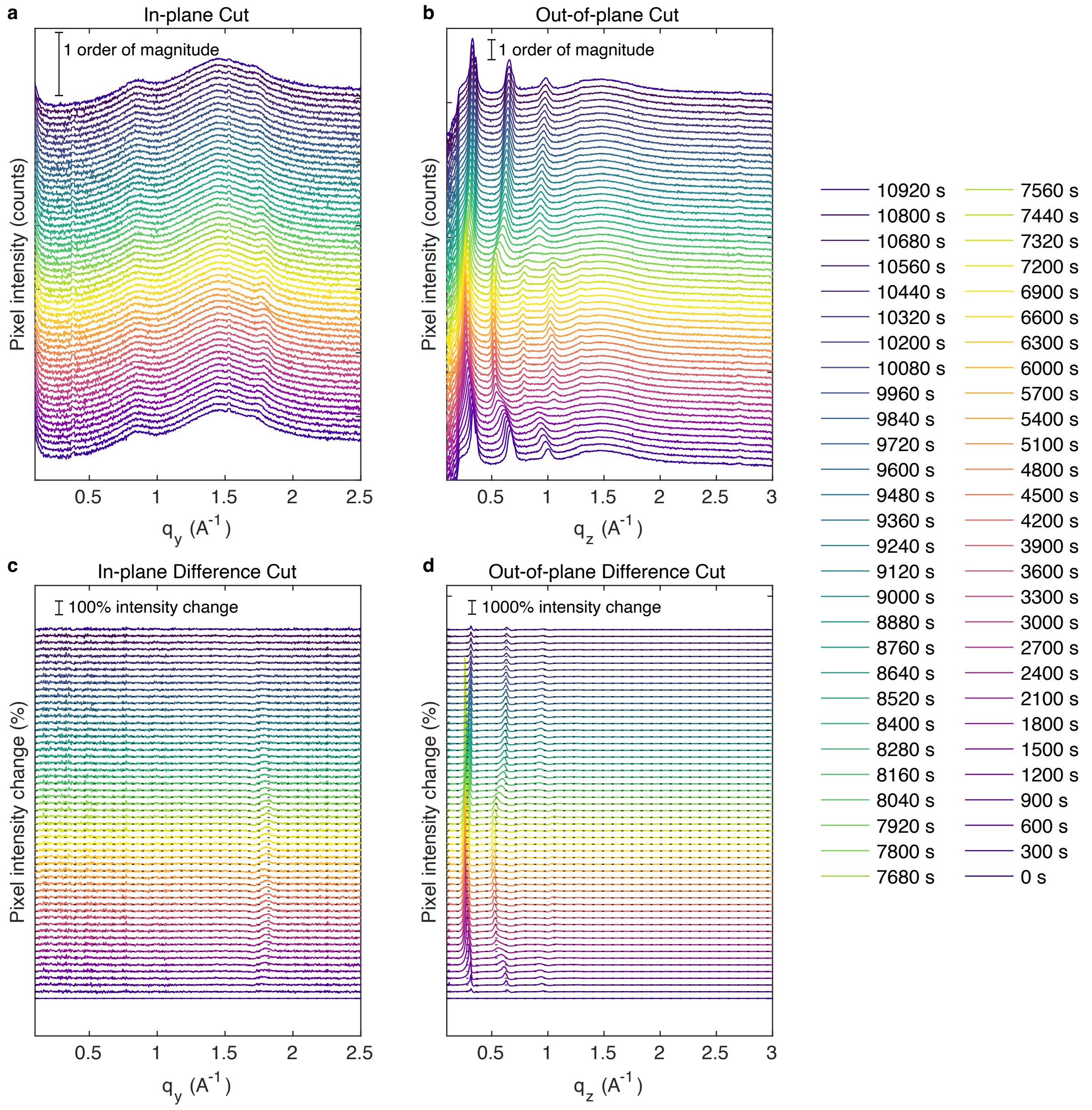

Supplementary Figure 22: **PBTTT in-situ ion gated GIWAXS linecuts**. (a) In-plane linecut ($0.03 > q_y > 0.06$) Å^{-1} plotted on a logarithmic intensity scale. (b) Out-of-plane linecut ($0.01 < q_z < 0.04$) Å^{-1} plotted on a logarithmic intensity scale. (c) In-plane difference linecut from $t = 0$ s. (d) Out-of-plane difference linecut from $t = 0$ s. Dashed lines in (c, d) indicate 0 intensity change baseline for each linecut.

currents of about 50 μA (corresponding to conductivity around 100 S cm^{-1}). In this initial phase, we observe a slight enhancement in (100) intensity, but also an increase in (100) peak width, suggestive of an increase in the crystalline fraction, but also an increase in the disorder of these crystallites. From about $t =$ 1,500 s the peak width reaches a maximum and begins to narrow, while simultaneously the stacking distance rapidly increases by 5 Å. The higher order peaks similarly show maxima in the peak width, although these occur at later times for successively higher order peaks; for (100) the maximum occurs near $t =$ 1,500 s, while for (200) it is near $t =$ 2,000 s and for (300) it is near $t =$ 2,500 s. Order-dependent broadening is a well-known signature of paracrystalline disorder,[23] which is characterized by a loss of coherence over longer distances (i.e. cumulative disorder). This time period between $t =$ 1,500 and 2,500 s also corresponds to the period where the lamellar stacking distance is rapidly changing, which we previously argued reflect the nucleation of a new, ion-intercalated crystalline phase.[5] These intercalated crystallites grow and consume the relatively disordered, partially intercalated crystallites or amorphous regions, their increasing size leads to longer coherence lengths and a corresponding decrease in successively higher order peak widths. Beyond $t =$ 2,500 s the stacking distance remains essentially constant, while all lamellar stacking peaks show a slow decrease in peak width, indicative of a slow increase in order, presumably as ionic vacancies within the lattice

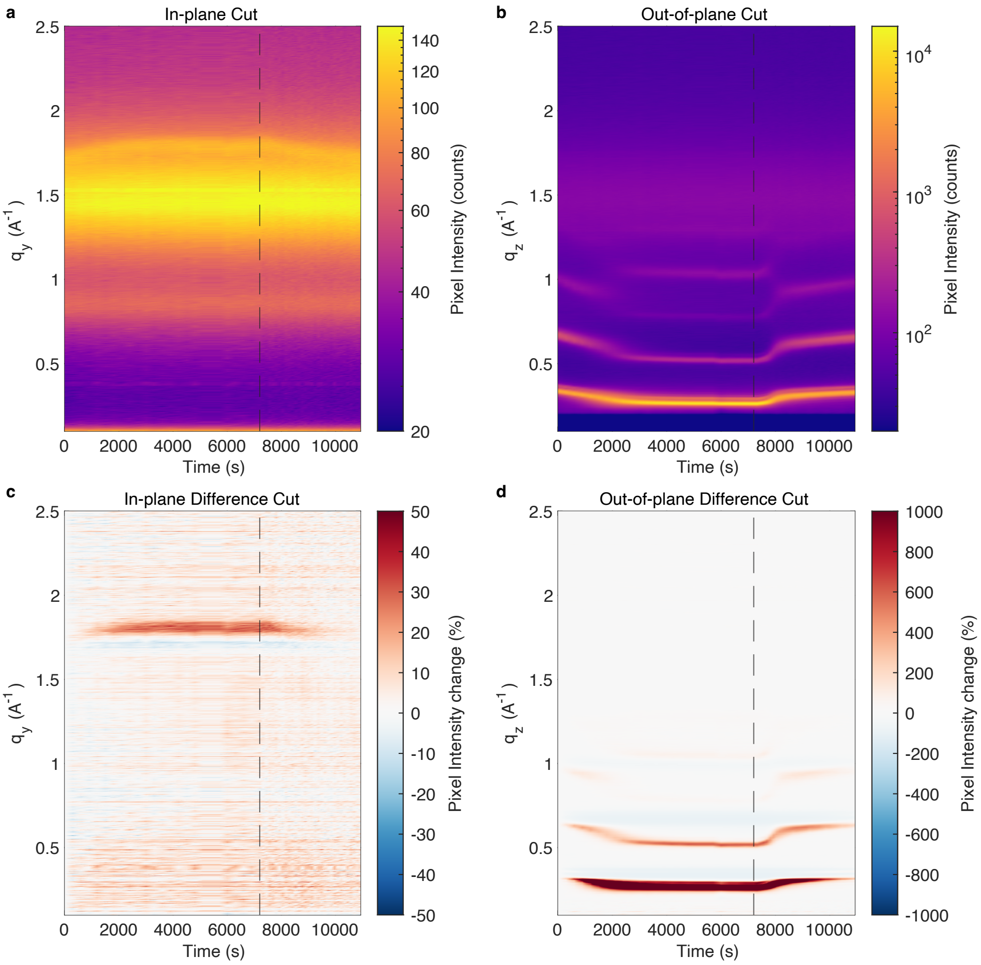

Supplementary Figure 23: **PBTTT in-situ ion gated GIWAXS time-dependent intensity plots**. (a) Pseudocolor plot of in-plane linecut intensity vs. time. (b) Pseudocolor plot of out-of-plane linecut intensity vs. time. (c) In-plane difference linecut from $t = 0$ s. (d) Out-of-plane difference linecut from $t = 0$ s. Dashed lines indicate the time when the gate voltage was switched from -2.0 V to $+1.5$ V.

are filled. The dedoping scan shows essentially identical behavior; the higher order stacking peaks broaden slightly earlier, as ions begin to de-intercalate and reduce the coherence length. Together, these observations indicate that the ion-intercalated phase formed at high doping levels is highly stable and well-ordered, and that crystalline order increases further as ionic vacancies in the intercalated co-crystal are filled.

The behavior of the π-stacking peak (Supplementary Figure 24(j)) is more straightforward. We observe a steady decrease in stacking distance and peak width up to about t = 2,500 s, consistent with previous reports,[5] due to polaron delocalization over adjacent chains in crystalline domains.[24] This increase in order, presumably a result of the void space in the sidechain region of PBTTT [26, 6] is responsible for the high conductivity in PBTTT:TFSI. However, the high order of this intercalated phase likely also explains the inability to reach Regime II in PBTTT:TFSI. We previously showed that the maximum achievable doping level in PBTTT:TFSI appears to be 1 ion per monomer, i.e. half-band filling (Supplementary Note 3 and Extended Data Figure 7). As the lamellar stacking data in Supplementary Figure 24(e-i) suggests, this fully intercalated phase should be maxmimally ordered; after nucleation of the intercalated phase, peak widths monotonically decrease with increasing doping. To reach Regime II in PBTTT:TFSI, we therefore would need to introduce additional ions into this highly ordered crystalline phase. If a still more heavily doped ion-intercalated crystalline phase is not possible, incorporation of ions beyond 1 ion per monomer will come at an energetic cost proportional to the stability of the 1:1 intercalated phase. Therefore, the high degree of order in the 1:1 PBTTT:TFSI phase would be predicted to generate a shift in the $PBTTT^{+} \longleftrightarrow PBTTT^{2+}$ redox potential, likely past the stable electrochemical window of the polymer. We note that no higher energy redox wave is observable by CV in Extended Data Figure 3. In this sense, the fact that the most disordered polymer studied here, IDT-BT, can achieve the most extreme band filling, while the most ordered polymer, PBTTT, achieves the least, is likely not coincidental.

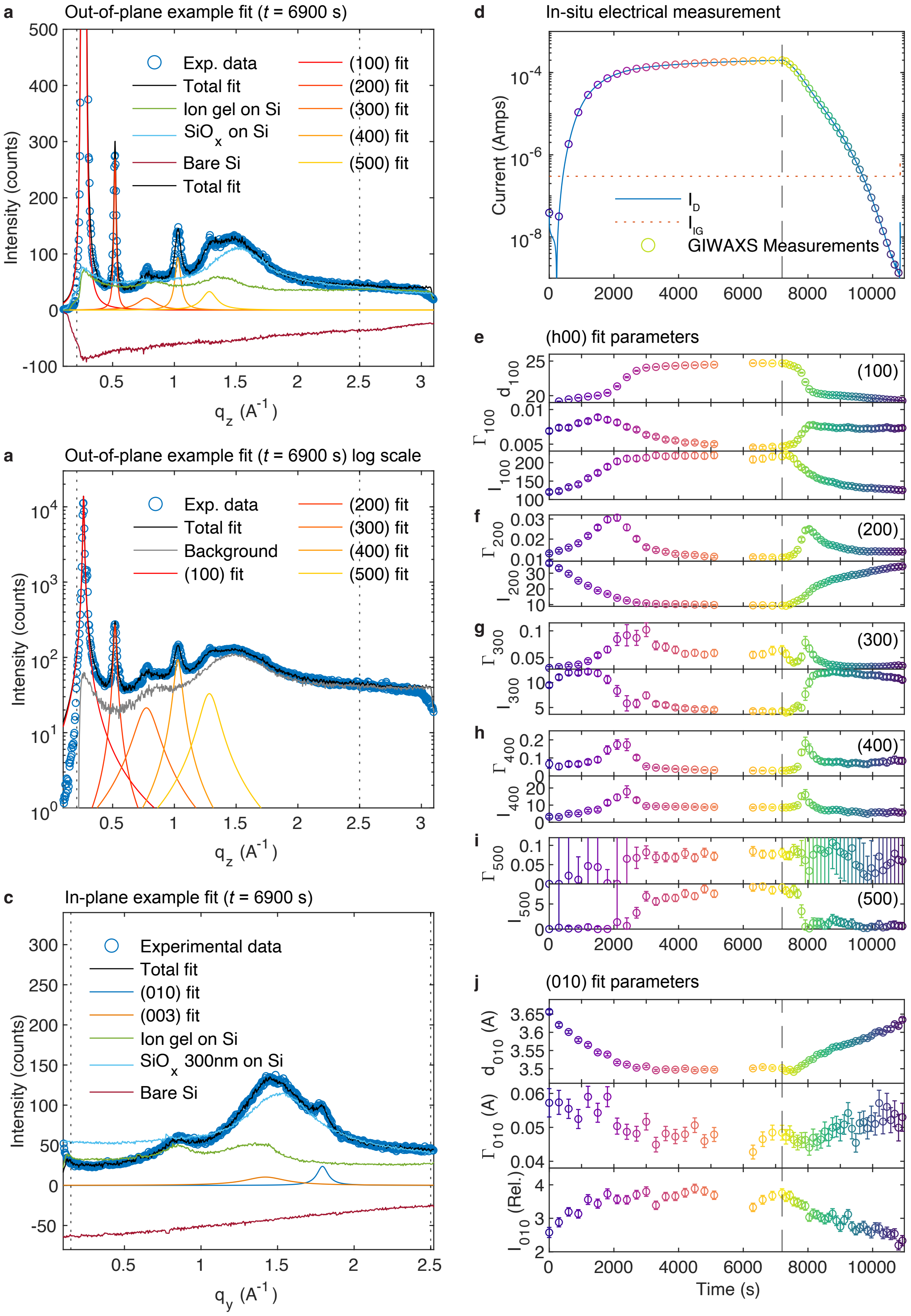

Supplementary Figure 24: **PBTTT in-situ ion gated GIWAXS peak fitting**. (a-b) Example out-of-plane linecut fit ($t = 6{,}900$ s shown) plotted on (a) linear scale and (b) log scale. (c) Example out-of-plane linecut fit ($t = 6{,}900$ s shown). Vertical dashed lines indicate range of data used for fitting. (d) *In-situ* electrical measurement; circles indicate GIWAXS measurements, color code corresponds to those in Supplementary Figure 22. Note that *in-situ* conductivity in PBTTT devices were measured via a separate channel to ensure accurate conductivity measurement; see Supplementary Figure 1. Dashed vertical line in (d-f) indicates the time when the gate voltage was switched from -2.0 V to $+1.5$ V. (e-i) Lamellar (h00) stacking parameters from out-of-plane linecut fits: (e) (100) stacking distance (top), peak width (middle), and peak amplitude (bottom); (f) (200); (g) (300); (h) (400); and (i) (500) peak widths (top) and intensities (bottom), respectively. (j) (010) stacking distance (top) peak width (middle) and peak amplitude (bottom) from in-plane linecut fits. Data in (e-j) are presented as best fit values with error bars representing the fit uncertainty as the standard error of the mean. Error bars for stacking distances reflect the random uncertainty of the fit only; there is an additional systematic error on the order of $w/2D \sim 3\%$ (where w is the channel width and D is the sample-detector distance).

Supplementary Note 5 First-principles electronic structure calculations

The band structure of pristine polymers has been obtained from periodic Density Functional Theory (DFT) simulations, using the all-electron, atomic centred basis formalism implementation available in CRYSTAL17.[27] This specific implementation makes the calculation of exact exchange much less computationally demanding compared to plane-wave based simulations, hence paving the way for the employment of accurate hybrid exchange-correlation functionals. Here, we resorted to the PBE0 functional for the description of the exchange-correlation potential,[28] coupled with the def2-SVP basis set. Calculations have been performed for a single polymer chain (1D-periodic model), considering a single chemical repeat unit (see Figure 1 in the main text) in the cell. A self-consistent calculation was performed considering a $6 \times 1 \times 1$ automatic sampling of the Brillouin zone, in the Monkhorst-Pack scheme,[29] where only the reciprocal-space direction associated to the polymer chain axis is considered. We set SCF convergence parameter to 10^{-8} Ha and very tight integral tolerance factors (keyword `TOLINTEG 10 10 10 10 20`).

The DFT band structures and densities of states (DOS) for the valence states of pristine IDT-BT, PBTTT and DPP-BTz are shown in the left-hand and middle panels of Supplementary Figure 25 respectively. The bands of these three systems reveal a rather complex structure, characterized by the simultaneous presence of dispersive and flat bands. In order to gain insight on the microscopic features determining such a convoluted band structure, we compare the DFT simulations to a multi-band 1D tight binding (TB) model. This model includes several orbitals for the different fragments (i.e. rigid conjugated units separated by soft torsions) of the repeat units, where the fragments represent the sites of the polymer chain. Specifically, we considered 5 orbitals for IDT-BT (HOMO, HOMO-1, HOMO-2, HOMO-3 of IDT; HOMO of BT), 6 orbitals for PBTTT (HOMO, HOMO-1 of T-Me; HOMO, HOMO-1 of TT), and 11 orbitals for DPP-BTz (HOMO, HOMO-1, HOMO-2, HOMO-3 of DPP; HOMO, HOMO-1, HOMO-2 of BTz; HOMO, HOMO-1 of T). Site energies and transfer integrals have been computed at the PBE0/def2-SVP level, consistent with DFT band structures. Fragment calculations, including those employed to compute the Hubbard U (see below) have been performed with the ORCA code.[30]

The TB bands and DOS are superimposed to their DFT counterparts in Supplementary Figure 25, showing a good agreement with reference calculations, especially in the high-energy region that is relevant to p-type doping. The right-hand panels of Supplementary Figure 25 report the partial densities of states (PDOS), i.e. the partitioning of the DOS into the contribution of single orbitals, shedding light on the nature of the bands. For IDT-BT, we recognize two topmost valence bands separated by a 0.1 eV energy gap around -1 eV. We emphasize that in spite of this small gap, the two bands have a neatly distinct character, the highest-energy band (1 eV bandwidth) being mostly composed by the IDT HOMO and the second one by the IDT HOMO-1. Both bands have a minor contribution from the BT HOMO, mediating the interaction between non-neighboring IDT units *via* a superexchange mechanism. This result supports the interpretation of experimental data in Figure 1 of the main text, ascribing Regimes I and II (i.e. up to charge densities of approximately 2 electrons per repeat unit) to the progressive depletion of a first band by electrochemical doping, and Regime III to the same process on the second band which lies very close in energy to the first one. Moreover, the insight gained from the TB PDOS permits to safely adopt an effective single-band model for IDT-BT in Regimes I and II, which we adopt in the following to describe electron-electron and electron-ion interactions at different charge densities.

In contrast, the band structure of PBTTT can instead be interpreted as a 4.5 eV-wide band formed of similar proportions of the T-Me and TT HOMOs, which is cut in the middle by dispersionless bands. The high-energy valence states of DPP-BTz are instead characterized by two well-separated bands of 0.6 and 0.4 eV bandwidth, respectively. These states feature comparable proportion of states from the DPP, BTz, and T HOMOs.

The on-site electronic Hubbard repulsion has been computed at the DFT (PBE0/def2-TZVP) level as $U = E^{2+} + E^{0} - 2E^{+}$,[31] where E^0, E^+, and E^{2+} are the total ground-state energies of the neutral fragment, cation, and di-cation, respectively. The calculated U terms are reported in Supplementary Table 3. We have considered as a fragment both the entire chemical repeat unit and its subunits, e.g. for IDT-BT we have also considered the IDT and the BT separately. We note that for this system, on which we will mostly focus in the following, taking the whole IDT-BT repeat unit or just the IDT fragment leads to similar U values, due to the localization of the highest occupied molecular orbitals (HOMO) on IDT. Calculations have been performed in vacuum and in a polarizable continuum model (PCM) with dielectric constant $\varepsilon_r = 4$, typical of organic materials.

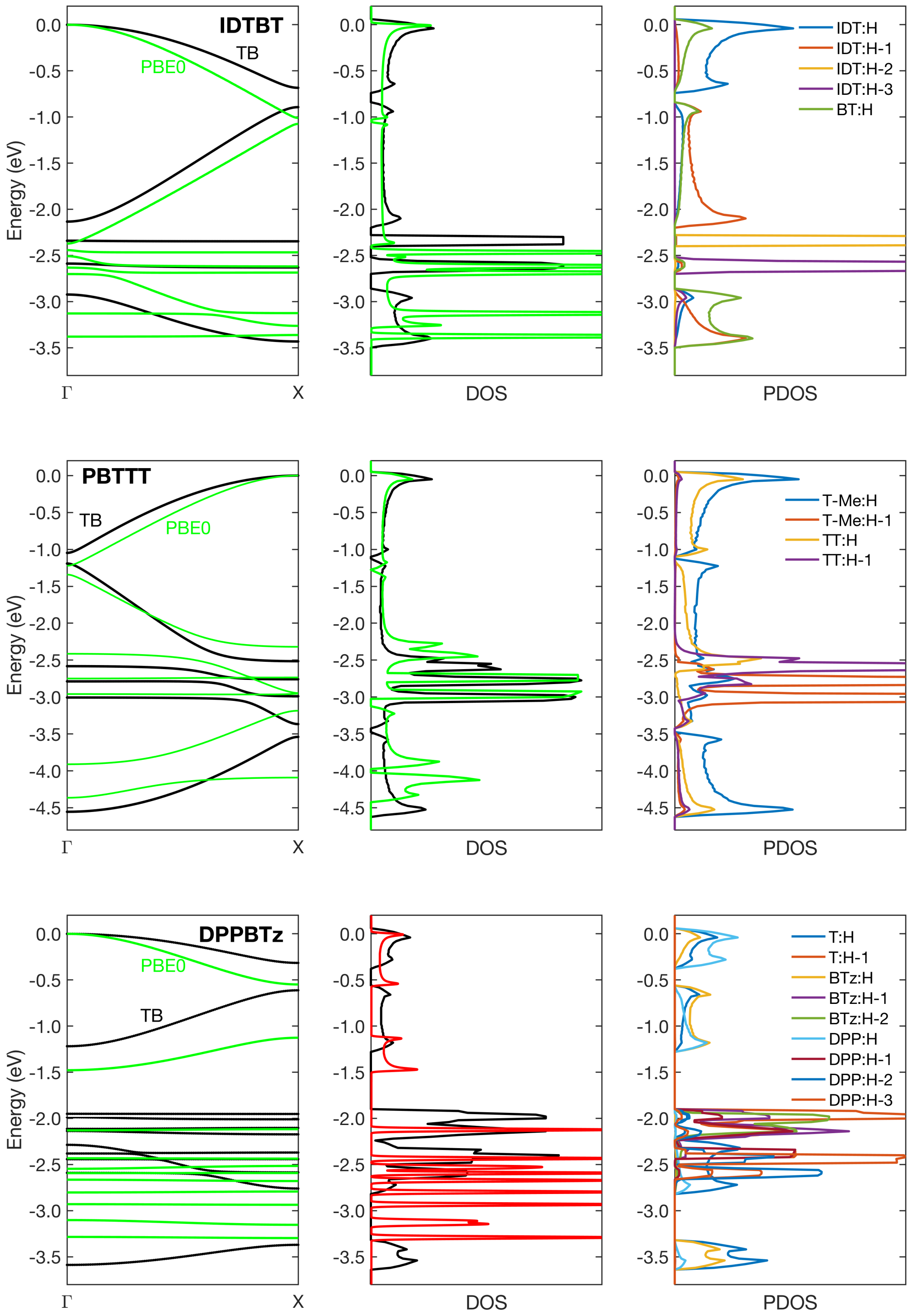

Supplementary Figure 25: **Bandstructure of IDT-BT, PBTTT, DPP-BTz**. DFT (PBE0, green lines) and tight binding (TB, black lines) band structure (left-hand panels) and DOS (central panels) for 1D chain of IDT-BT (top), PBTTT (middle) and DPP-BTz (bottom). The left-hand panels shows the partial DOS from TB calculations, showing the contribution of the different fragment orbitals to the DOS (HOMO is abbreviated as H). Note that IDT-BT and DPP-BTz present two thiophenes per repeat unit, whose contributions to PDOS have been added. The top of the valence band is set to zero energy.

Supplementary Table 3: Hubbard U calculated for the repeat units and sub-units of IDT-BT, PBTTT and DPP-BTz at the PBE0/def2-TZVP level, in vacuum ($\varepsilon_r = 1$) and in PCM ($\varepsilon_r = 4$).

System	Fragment	U [eV]	
		$\varepsilon_r = 1$	$\varepsilon_r = 4$
IDT-BT	IDT-BT	3.54	1.56
	IDT	4.18	1.87
	BT	6.21	3.00
PBTTT	PBTTT	3.94	1.69
	T-Me	6.99	3.55
	TT	5.85	2.68
DPP-BTz	DPP-BTz	3.46	1.48
	DPP	5.62	2.58
	BTz	6.21	3.00
	T	7.65	3.92

Supplementary Note 6 Non-equilibrium transport in IDT-BT, DPP-BTz, and PBTTT

6.1 Non-equilibrium transport in IDT-BT

We first cover further details of the non-equilibrium transport measurements on IDT-BT. In Figure 2 of the main article, we show the field-effect transfer curves for 6 representative doping states. In Supplementary Figure 26, we include a full set of transfer curves for all 14 doping states that were measured at 160 K. One can observe the full evolution of the apparent ambipolarity with increasing doping level; the characteristics evolve from predominantly p-type (Regime I), to ambipolar (near peak) and predominantly n-type (Regime II), and further to ambipolar again (near valley), and back to predominantly p-type (Regime III).

As mentioned in the main article, room temperature operation of double-gated devices is not possible. When operating our double-gated devices at room temperature the field-effect gate is still capacitively coupled to the ion gel, preventing V_{FG} and V_{IG} from being controlled independently. An example is shown in Supplementary Figure 27. From the ion gate transfer curve in Supplementary Figure 27(a), we can see that the device turns on at around +0.5 V, and the current is already quite high (2 μA) at 0 V and further increases to 5 μA at -0.5 V. The field-effect transfer curves, however, behave quite differently from what one would expect. For example, the maximum current for $V_{\mathrm{IG}} = 0$ V and -0.5 V are very similar, the hysteresis is huge, and the 0 V curve has a very large slope. Using the capacitance of 300 nm SiO_2 and assuming charge density modulation only occurs at the interface, the apparent mobility extracted from the $V_{\mathrm{IG}} = 0$ V transfer curve is estimated to be over 200 cm^2 V^{-1} s^{-1}, which is not realistic. These observations suggest that sweeping V_{FG} can still affect the bulk doping level of the polymer (i.e. by driving ions in and out of the polymer) when the ions are mobile. Although only data for PBTTT is shown in Supplementary Figure 27, these arguments also apply more generally to DPP-BTz and IDT-BT. Consequently, we only sweep V_{FG} at low temperatures when the ions are fully frozen.

Therefore, we opted to use an *ex-situ* doped IDT-BT device to study the field-effect transfer characteristics near room temperature. The full temperature dependence of the field-effect transfer curve of such a device in Regime I is shown in Supplementary Figure 28. A crossover from linear to non-linear occurs at somewhere between 240 K and 250 K, below which the transfer curve is always non-linear. Slightly above the crossover temperature (e.g., 260 K), the transfer curve exhibits large hysteresis, indicating that the timescale of the physics behind the non-linear effect at this temperature is comparable to the timescale of the transfer curve measurement. Similar phenomena have also been observed when the counter-ion is switched from TFSI to FSI as shown in Supplementary Figure 29; we note that the FSI results were acquired in Regime II, so the field-effect gate transfer curves are n-type.

For these measurements, no field-effect gate voltage is applied during cooling, so the center of the symmetric component is at 0 V. We show in Supplementary Figure 30 that if we apply a non-zero V_{FG} at temperatures above the crossover temperature and keep it on during cooling (i.e., apply field cooling conditions), we can "freeze" the new equilibrium configuration created at this new voltage, and hence move the center of the symmetric component to the applied voltage. Furthermore, the center of the symmetric component can be moved back to 0 V by applying a subsequent zero field cooling. These observations are completely reproducible. It is the disappearance of the symmetric component at high temperature, as well as the observation that the centre of the symmetric component could be shifted by field-cooling, that strongly suggest that this component is caused by a non-equilibrium effect that only occurs below some crossover temperature. As discussed in the main text this is reminiscent of observations in amorphous/polycrystalline InO [32] and granular Al.[33]

It is helpful to quantify the relative contribution of the symmetric component to the total field-effect response. This could be quantified by extracting apparent mobilities associated with the anti-symmetric and symmetric components of each transfer curve, that we term anti-symmetric mobility μ_{asym} and symmetric mobility μ_{sym}, respectively. Here we provide some considerations of this phenomenological mobility estimation. The anti-symmetric component of each transfer curve is by definition completely linear, allowing a relatively straightforward anti-symmetric mobility extraction from such near-ideal transfer characteristics. On the contrary, the symmetric component shows significant non-linearity; in fact, this component is expected to have a Lorentzian lineshape.[34] We have adhered to the recommendations of Podzorov et al.[35] for the estimation of mobilities from non-ideal transfer characteristics, i.e. the mobility estimates are based on electrically equivalent ideal field-effect transistors with linear transfer charactreristics and zero threshold voltages.

The evolution of anti-symmetric and symmetric mobilities with doping level for IDT-BT is summarized in Extended Data Figure 10. At the start of Regime I, the linear component initially dominates transport, resulting in a mostly unipolar p-type transfer characteristic. As the doping level is increased towards the conductivity peak, the transfer curve becomes increasingly ambipolar. At the conductivity peak (state #4)

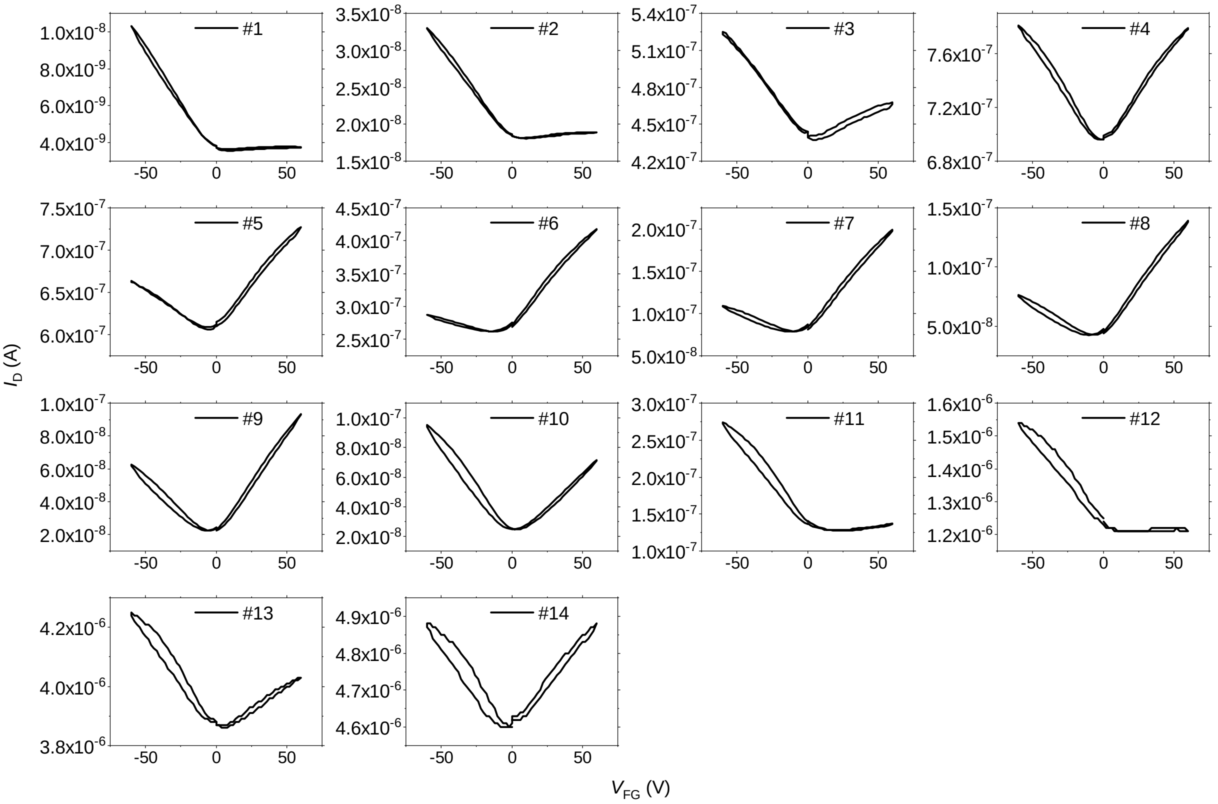

Supplementary Figure 26: **Detailed doping-level-dependent evolution of IDT-BT's field-effect transfer curves.** This figure shows the complete measurement dataset of Figure 2(b); doping level increases with increasing doping state number, the details of which can be seen in Figure 2(b). Measurements were taken at 160 K with $V_{\mathrm{D}} = -0.1$ V.

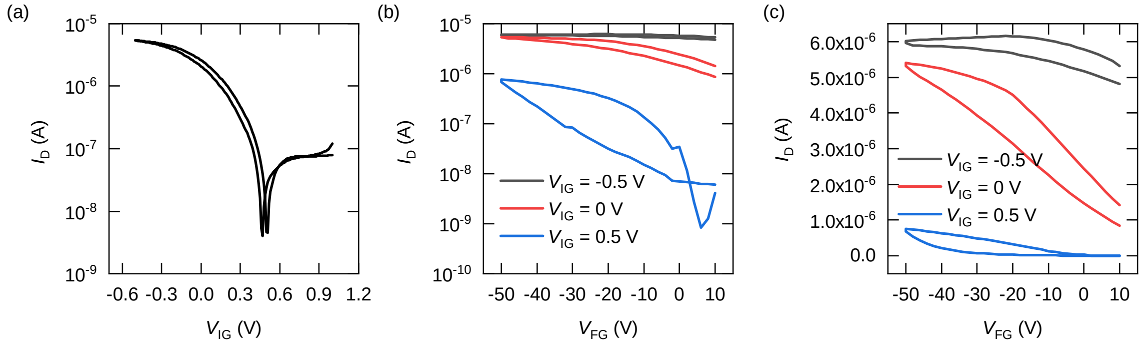

Supplementary Figure 27: **Room temperature operation of a double-gated PBTTT device.** (a) Ion gate transfer curve without applying any field-effect gate voltage. (b) Semi-log scale and (c) linear scale field-effect gate transfer curves at different ion gate voltages. All of the transfer curves were taken at V_{D} = -0.1 V.

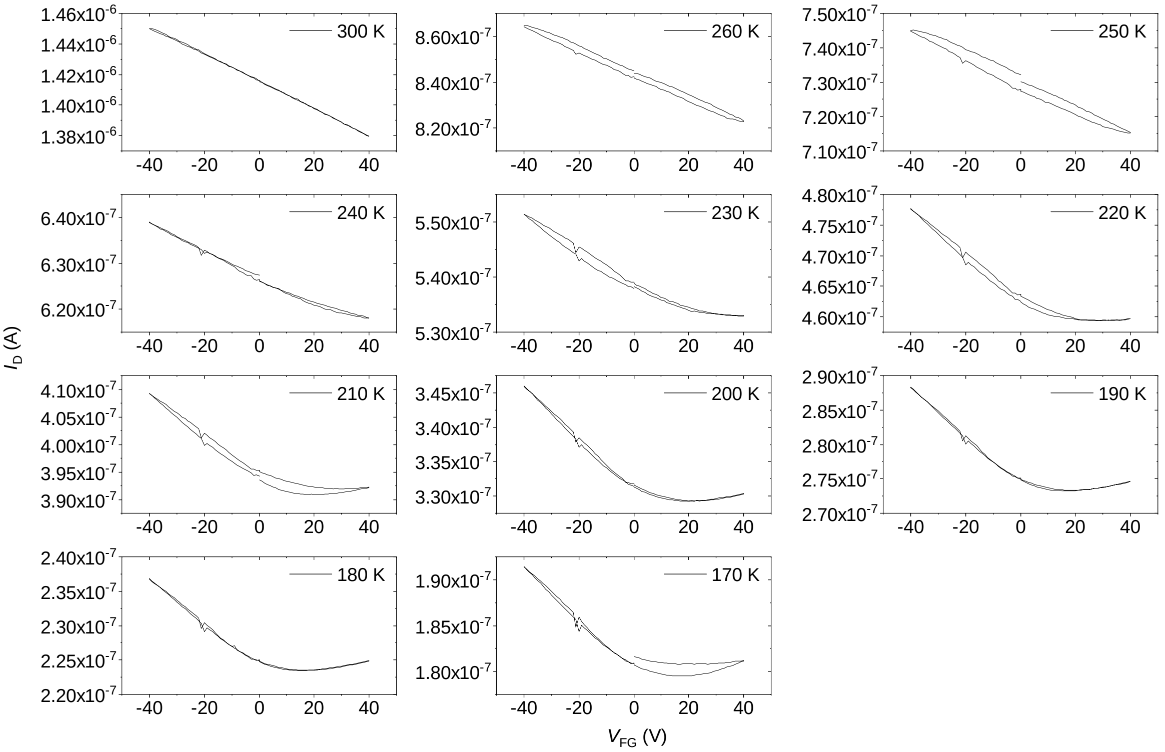

Supplementary Figure 28: **Temperature dependence of the field-effect transfer curves of an *ex-situ* TFSI doped IDT-BT above and below the crossover temperature of non-equilibrium transport.** This figure shows the complete measurement dataset of Figure 2(c). Measurements were taken between 300 K and 170 K with V_D = -0.1 V.

we see a very ambipolar transfer characteristic with balanced p- and n-type transport. Here, transport is dominated by the symmetric component, as signified by a local maximum in the symmetric mobility and an almost-zero anti-symmetric mobility.

It is important to emphasize that μ_{asym} should not be interpreted as an average carrier mobility. For example, at the conductivity peak the average carrier mobility (state #4) is $\sim$ 0.005 cm^2 V^{-1} s^{-1} as estimated from the bulk conductivity (assuming 1 charge per monomer at the peak), while μ_{asym} is almost 0. We suggest that μ_{asym} can be interpreted as the mobility of extra carriers added into states near E_F. At the peak adding extra carriers to into the density of states does not lead to a change in the current, i.e. the mobility of these added carriers is zero.

Entering Regime II just beyond the conductivity peak, transport remains ambipolar in state #5, although higher currents are now achieved on the n-type side of the transfer characteristic. Correspondingly, in Extended Data Figure 10 we assign a negative anti-symmetric mobility to this state to signify that the transport is now predominantly n-type. The anti-symmetric mobility peaks in Regime II when the transfer curve is mostly unipolar n-type, e.g. state #6, which is roughly at the halfway between the peak and the valley. Past the halfway point we see both the anti-symmetric and symmetric mobilities decreasing, although at a higher rate for the former, such that the ambipolarity becomes again more and more pronounced. At the valley (state #9), complete ambipolarity is seen again. Although the symmetric mobility lies at a local minimum at the valley, the anti-symmetric mobility and bulk conductivity are reduced much more significantly. To illustrate the point, we emphasize that μ_{sym} at the valley is only 35% lower than that at the peak, while the bulk conductivity is 30 times lower. Consequently, across all transport regimes, the contribution from the symmetric component is most pronounced at the valley.

In Regime III we see significantly higher mobility values, which we have attributed to the higher dielectric constant and more efficient screening of Coulomb interactions in the HOMO-1 band. The magnitudes of both components increase away from the valley, with the anti-symmetric mobility reaching the global maximum in this experiment at state #12, exceeding 0.1 cm^2 V^{-1} s^{-1}, a very respectable value for a disordered, doped polymer. At the highest doping levels in states #13 and #14, the decrease in the anti-symmetric mobility is accompanied by a steep increase in the non-equilibrium counterpart. This is marked by the field-effect

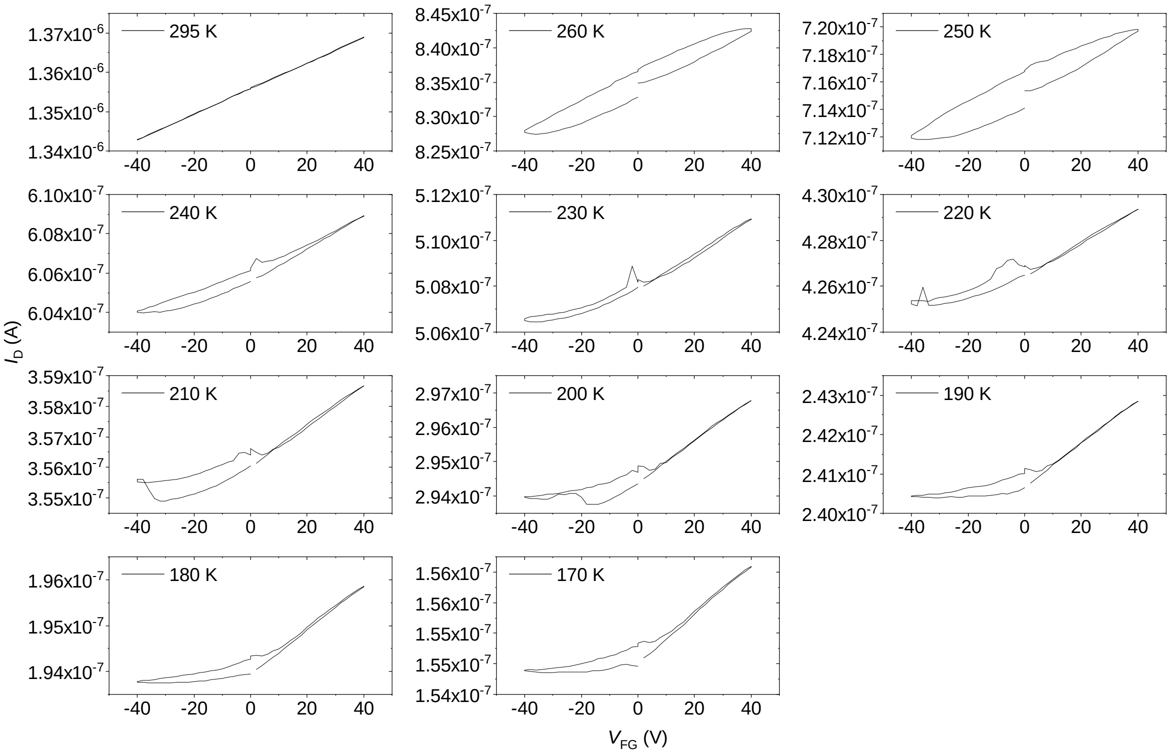

Supplementary Figure 29: **Temperature dependence of the field-effect transfer curves of an *ex-situ* FSI doped IDT-BT above and below the crossover temperature of non-equilibrium transport.** Measurements were taken between 295 K and 170 K with $V_{\mathrm{D}} = 0.1$ V. This device was doped to Regime II, so the field-effect transfer curves are n-type.

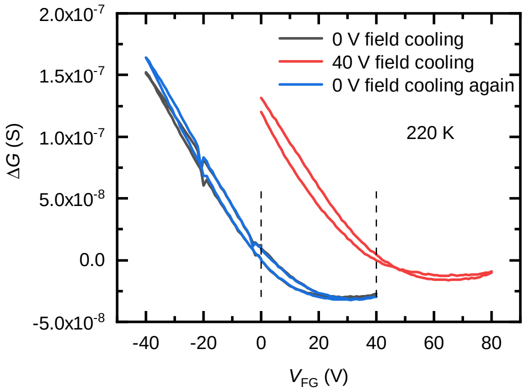

Supplementary Figure 30: **Tuning the position of the non-equilibrium 'dip' by field cooling.** Transfer curves were taken at 220 K. Between each transfer curve, the device was heated to 260 K, and then cooled back to 220 K with the corresponding value of V_{FG}. Here, the change of conductance is expressed as $\Delta G = G – G_{\mathrm{f}}$, where G_{f} is the conductance at the gate voltage for field cooling (0 and 40 V, respectively). This is the same device shown in Figure 2(c).

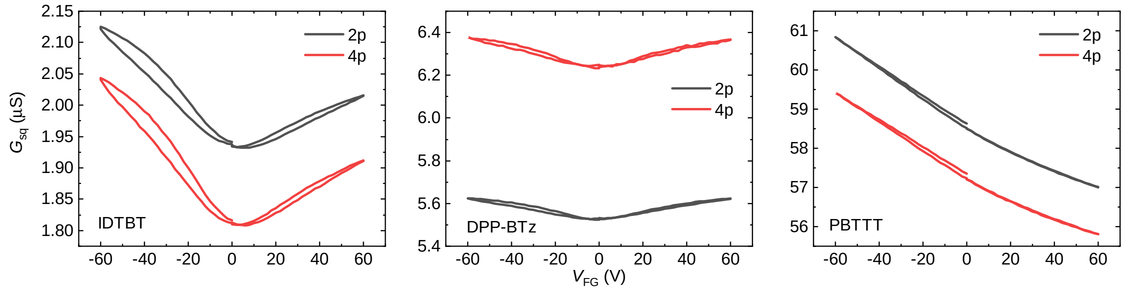

Supplementary Figure 31: **Comparison of two-probe and four-probe field-effect transfer curves of the same device: IDT-BT (left), DPP-BTz (middle), and PBTTT (right).** Notice that there is no substantial difference between two- (2p) and four-probe (4p) sheet conductance values (G_{sq}). Measurements were taken at 160 K with V_D = -0.1 V.

gate transfer curves of states #13 and #14 becoming ambipolar again, reminiscent of the trends seen at the conductivity peak (state #4), inferring that at the highest doping values we are approaching the centre of the HOMO-1 band.

We summarize the recurrent trends in the anti-symmetric and symmetric mobilities of IDT-BT. Across all transport regimes, the anti-symmetric mobility values are highest in the middle of each regime (cf. states #3, #6, and #12). On the other extreme, anti-symmetric mobilities are near-zero on states marking carrier polarity inversions (states #4, #10, and likely #14) in addition to the more trivial case of states with very low doping levels (states #1 and #2). Across Regimes I, II, and III, the symmetric mobility exhibits a similar dependence on doping-level as the conductivity, i.e., it is locally maximized on the conductivity peak, and likewise locally minimized in the valley. However, it varies less than the conductivity across the different doping states.

We note that the transfer curves in Figure 2 of the main text, Extended Data Figure 10, as well as Supplementary Figures 26, 28, and 29 have all been obtained through two-probe measurements. As shown in Supplementary Figure 31, our comparison of two- and four-probe double-gated measurements show no significant difference in the non-linearity of the transfer curves. The use of two-probe measurements is therefore sufficient in Figure 2, Extended Data Figure 10, Supplementary Figures 26, 28, and 29, where we are primarily interested in the evolution of the non-linearity in the transfer curves with doping level or temperature.

We now turn our focus to how doping level affects the temperature dependence of IDT-BT's field-effect gate transfer curve. Selected transfer curves at illustrative temperatures are shown in Supplementary Figure 32. While we do not observe any significant difference between the evolution of the non-linearity in our two- and four-probe measurements, contact resistance effects will inevitably compromise the accuracy of the extracted mobility values. Accordingly, the transfer curves in Supplementary Figure 32 and the extracted parameters (Supplementary Figure 33) have been obtained via four-probe measurements to minimize the effect of contact resistance. We also would like to note that the non-equilibrium feature in IDT-BT is not affected by the gate voltage scan rate (Supplementary Figure 34), which indicates that the timescale related to the non-equilibrium feature in IDT-BT is much larger than the timescale for transfer curve measurements at temperatures below 190 K.

For IDT-BT, for all doping states there is a significant non-linearity of the transfer curves at any temperature below 190 K. We quantify the extent of this non-linearity by taking the quantity $\Delta G/G_0$, as also used by Pollak et al. in their InO film studies.[32] Here, the change of conductance ΔG is defined as $\Delta G = G{-}G_0$ and G_0 is the conductance at $V_{FG} = 0$. At any doping level, $\Delta G/G_0$ grows exponentially with decreasing temperature as shown in the bottom panels of Supplementary Figure 33. There is a tendency for the ratio to be lowest on states of higher conductivities, e.g. the peak and the second rise, where G_0 from the bulk of the sample dominates. Likewise, $\Delta G/G_0$ is maximized for states of lower conductivities, with the highest being recorded around the valley.

From the transfer curves we also extracted the anti-symmetric mobility μ_{asym} and symmetric mobility μ_{sym} as shown in the top and middle panels of Supplementary Figure 33. These panels are shown on logarithmic scales to visualize better the small mobility values at low temperatures, with the anti-symmetric mobility plotted on symlog scale to also account for the negative mobilities in Regime II. We see that both μ_{asym} and μ_{sym} are thermally activated. The symmetric mobility is however less thermally activated compared to the bulk counterpart associated with G_0, hence the increasing $\Delta G/G_0$ as temperature goes down. It is at the very low temperatures ($\sim$ 50 K) that we see the most significant non-linearity of the field-effect gate transfer curves, with ΔG easily reaching 10% of the bulk conductance G_0.

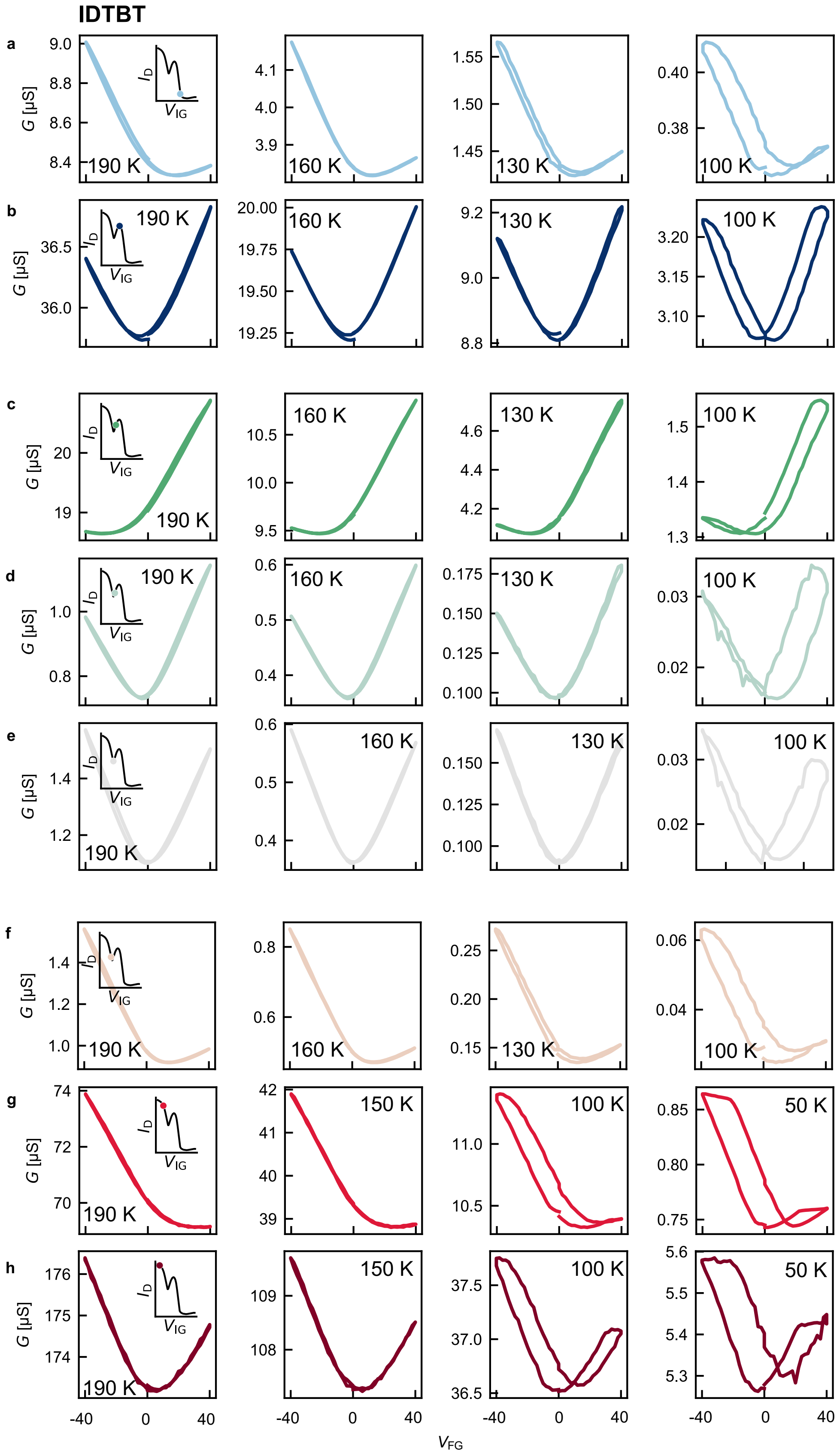

Supplementary Figure 32: **Temperature dependence of the field-effect gate transfer curve of IDT-BT at various doping levels.**

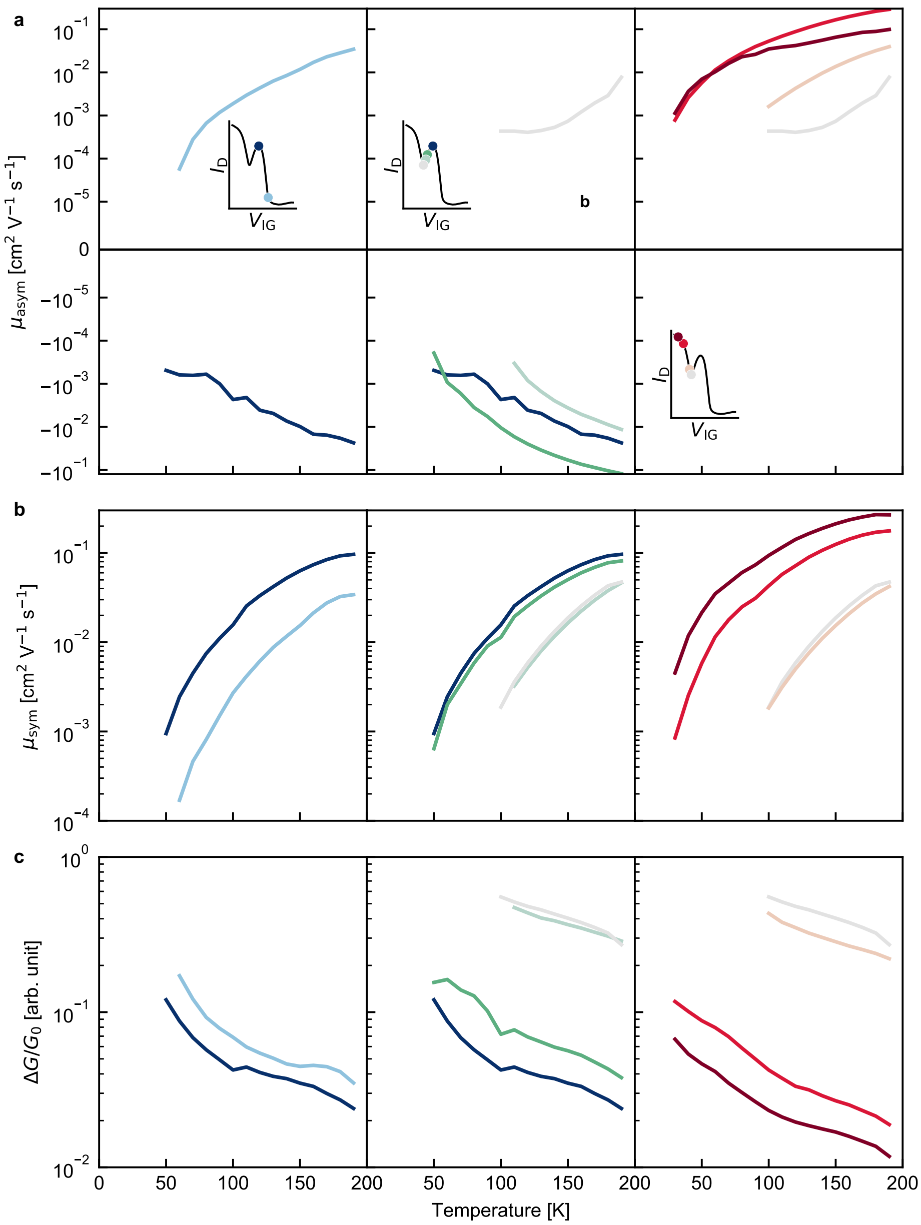

Supplementary Figure 33: **Parameters describing the temperature dependence of IDT-BT field-effect gate transfer curve.** (a) anti-symmetric mobility μ_{asym}, (b) symmetric mobility μ_{sym}, and (c) $\Delta G/G_0$ ratio as a function of temperature. The parameters are extracted from the transfer curves shown in Supplementary Figure 32, as defined in Figure 2(c) of the main article. *The top panels are plotted on a symlog scale.*

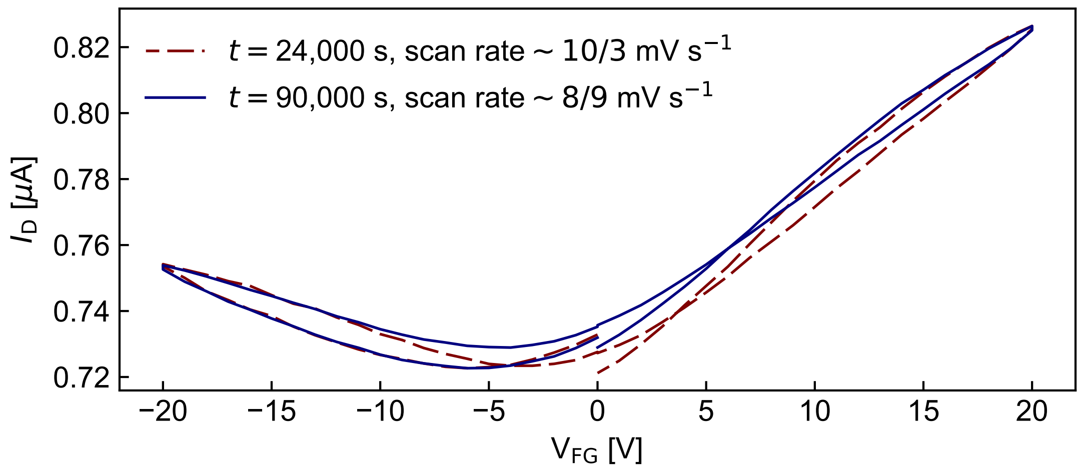

Supplementary Figure 34: **Scan rate dependence of IDT-BT's field-effect gate transfer curve.**

At 100 K and lower, hysteresis can be observed in the field-effect gate transfer curves, which is clockwise for negative V_{FG} and counter-clockwise for positive V_{FG} (drain current for the reverse scan is higher for both sides). The main reason for the emergence of hysteresis at low temperature is the insulating nature of IDT-BT: with decreasing temperature, the polymer resistance becomes higher and higher while the magnitude of hysteresis does not change much, such that hysteresis becomes obvious in the measured drain current. For example, the difference in drain current between forward and reverse scans is on the order of 1 nA, which is hardly visible compared with the gate-modulated drain current at 190 K (tens or hundreds of nA modulation depending on doping levels), but becomes clearly visible when the drain current modulation is only several nA at much lower temperatures.

6.2 Double-gated IDT-BT devices with a different ion gel: BMI PF_6

We discuss devices with BMI PF_6 ion gels, where the anion is both smaller and more symmetrical than TFSI. Supplementary Figure 35 shows field-effect transfer curves measured on two IDT-BT double-gated devices, one using (a) a BMP TFSI ion gel and the other using (b) a BMI PF_6 ion gel. We observe essentially identical behavior at 180K for both devices. In Regime I, we observe p-type dominant ambipolar behaviour, becoming more ambipolar at the conductivity peak between Regime I and II. Note that the curves shown nominally for the peak are not doped precisely to the peak: the TFSI device was measured slightly before the peak (Regime I) while the PF_6 device was measured slightly after (Regime II). In Regime II both devices show n-type dominant ambipolar behavior, again becoming more ambipolar near the valley. Finally, in Regime III, we again observe p-type behaviour in both samples. The temperature dependence of the transfer curves in Regime I, shown in the lower right subpanel of (a) and (b), show similar reduction in conductivity for both ions. PF_6 is both smaller and more symmetrical than TFSI, and does not have a permanent dipole moment. Therefore, the observation of similar non-equilibrium behaviour in both ions suggests that the origin of the non-equilibrium effects are not due to dipolar interactions, such as a freeze-out of ionic rotational motions.

6.3 Non-equilibrium transport in DPP-BTz

We now discuss how non-equilibrium transport manifests itself in DPP-BTz. For these double gated transport measurements we use unaligned films, since we have not yet optimized the alignment process for these ultrathin ($\sim$ 10 nm) polymer films.

In DPP-BTz, we see non-equilibrium transport signatures very similar to that of IDT-BT. This is most clearly seen in the 160 K field-effect gate transfer curves of DPP-BTz (Extended Data Figure 9), showing similar nonlinear and ambipolar behaviour. As with IDT-BT, we see signficantly larger field-effect gate modulation than the modulation expected from the expected density of the field-effect modulated carriers. On the peak of the conductivity (i.e. around state #4) we observe an ambipolar increase in the source drain current with the application of both positive and negative field-effect gate voltages. The field-effect response of DPP-BTz's field-effect gate is completely consistent with that of IDT-BT. Importantly, this demonstrates clearly that non-equilibrium transport is not limited to a relatively low conductivity regime, but can also be observed in polymers with high conductivities exceeding 100 S cm^{-1}.

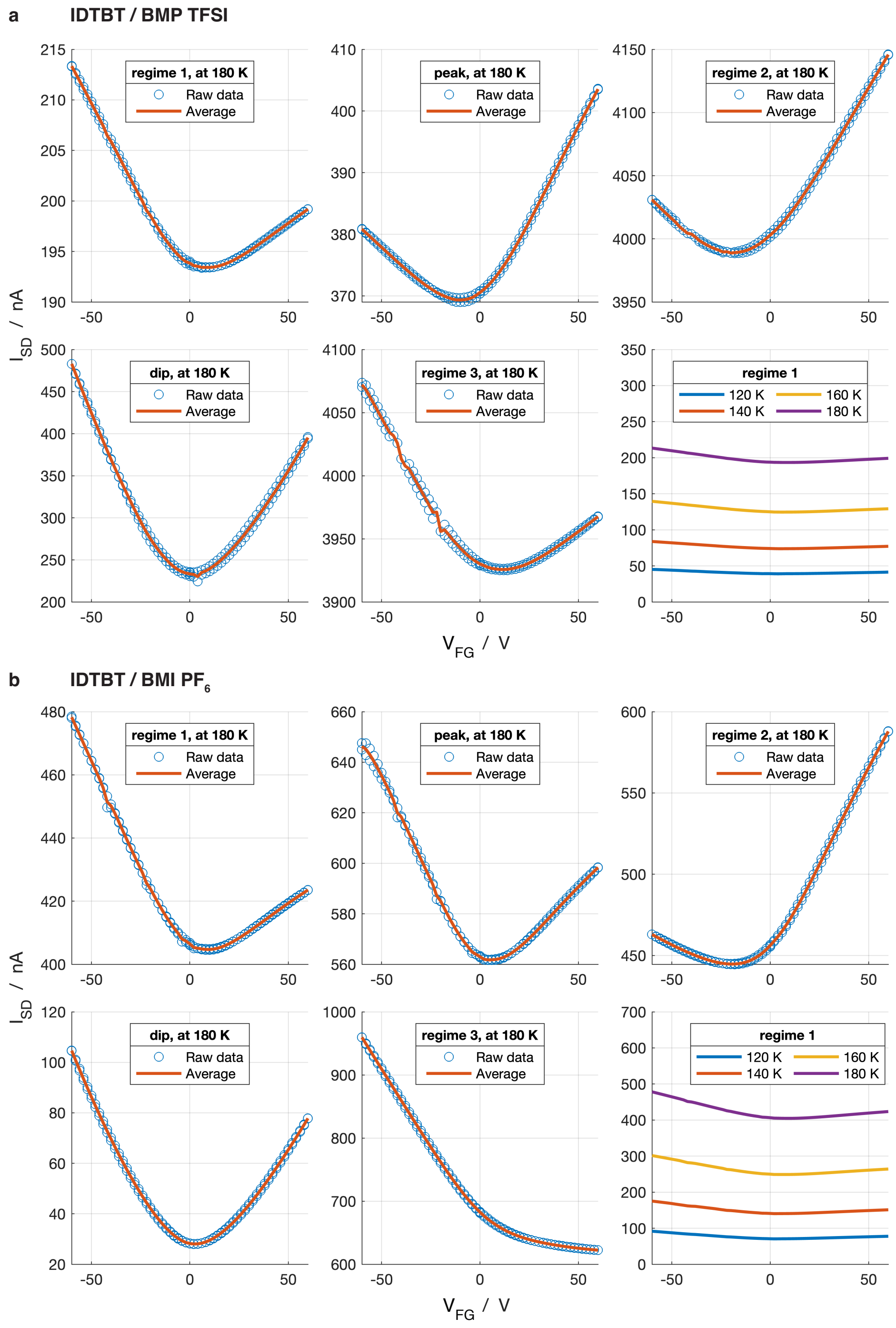

Supplementary Figure 35: **IDT-BT field-effect gated transfer curves with various ion gels.** (a) IDT-BT / BMP TFSI field-effect gate transfer curves at various doping states (indicated in each panel). Lower right panel shows temperature-dependence of the Regime 1 field-effect transfer curve. (b) Corresponding data in an IDT-BT / BMI PF_6 device.

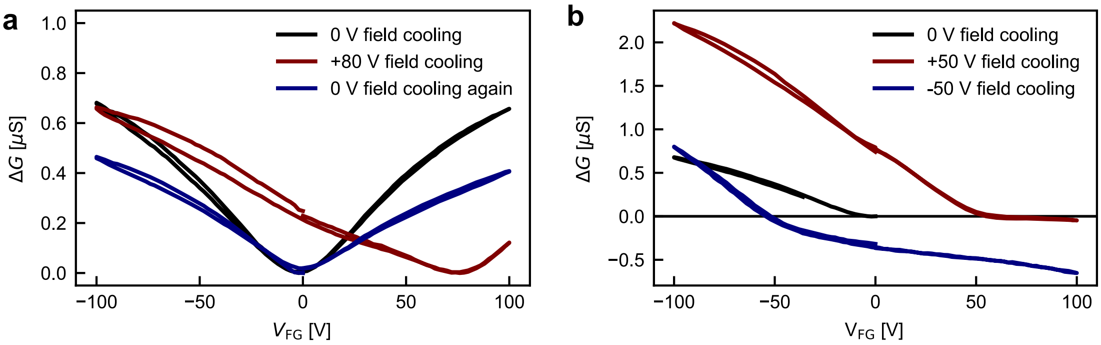

Supplementary Figure 36: **Field cooling of DPP-BTz.** Transfer curves were taken at 150 K, for a device whose doping level has been set to (a) the peak of the OECT transfer curve and (b) slightly before the peak. Between the different fields, the device was heated to 190 K, and stressed at 0 V for more than 12 hours, before cooling back down with the corresponding value of V_{FG}.

Field cooling experiments on DPP-BTz provide further evidence for the non-equilibrium behaviour. As shown in Supplementary Figure 36, application of a non-zero field-effect gate voltage on cooling below the crossover temperature results in shifts of the center of the symmetric component of the transfer curve to the corresponding field-effect gate voltage, just as observed in IDT-BT (Supplementary Figure 30). The lower ΔG in the second 0 V field cooling sequence in Supplementary Figure 36(a) is likely to be caused by a slight degradation of the polymer film, as the measurements were taken a few days apart.

We note that the crossover in DPP-BTz seems to take place at a lower temperature than IDT-BT. For instance, in the DPP-BTz field cooling experiments (Supplementary Figure 36), the centre of the dip was controlled by stressing the field-effect gate at relatively low temperatures of 190 K and lower. In IDT-BT, in contrast, the field was applied at much higher temperatures, e.g. 260 K. This suggests that in DPP-BTz the ions remain able to respond to changes in carrier concentration down to lower temperatures when compared to IDT-BT.

The changes of DPP-BTz's field-effect gate transfer curves with doping level are summarized in Extended Data Figure 9(b). We see clear parallels between the trends of IDT-BT and DPP-BTz — the anti-symmetric mobility of DPP-BTz is initially small and positive (state #1), is maximized at ~ 0.27 cm^2 V^{-1} s^{-1} in the middle of Regime I (state #2), and as the doping level is increased further it turns negative signifying the p- to n-type transport transition around the peak (state #4). The doping level dependence of DPP-BTz's symmetric mobility, similarly to that of IDT-BT, closely resembles the trend of its conductivity. We recorded a maximum symmetric mobility of 0.15 cm^2 V^{-1} s^{-1} in the vicinity of its conductivity peak (states #3 and #4).

The main difference between IDT-BT and DPP-BTz with regards to the doping level dependence is evident mainly in Regime II, where DPP-BTz shows vanishingly small mobilities. For states #5, #6, and #7, where transport is predominantly n-type, we see very low anti-symmetric mobilities ranging from 4×10^{-3} to -1×10^{-3} cm^{-2} V^{-1} s^{-1}, which are about two orders of magnitude smaller than their Regime I counterparts.

Temperature evolution of DPP-BTz's field-effect transfer curve is summarized in Supplementary Figure 37. Unlike the case of IDT-BT, we have used two-probe measurements in these low-temperature experiments. As previously shown in Supplementary Figure 31, the two-probe and four-probe transfer curves look very similar, therefore we expect the two-probe results to be representative of the most important details.

The anti-symmetric mobility, symmetric mobility, and $\Delta G/G_0$ ratio are summarized in Supplementary Figure 39(a). We see a few trends that are analogous to IDT-BT. First, both the anti-symmetric and symmetric mobility are thermally activated. Similarly to IDT-BT, $\Delta G/G_0$ grows exponentially larger with lowering temperature, reaching highest values of upwards of $\sim$ 0.1 at 30 K. The lowest $\Delta G/G_0$ at any temperature is recorded at the conductivity peak, considering the large bulk conductance G_0 at this state; likewise, the ratio is the highest for a relatively low conductivity state (cf. the most highly doped state in Regime II). We do not notice any significant qualitative difference between the temperature dependencies between the μ_{asym}, μ_{sym}, and $\Delta G/G_0$ of IDT-BT and DPP-BTz.

6.4 Non-equilibrium transport in PBTTT

In PBTTT we see a slightly different manifestation of the non-equilibrium behaviour. As shown in Extended Data Figure 9(a), for the highest conductivity states #3 (σ = 1,150 S cm^{-1}) and #4 (1,700 S cm^{-1}), we observe a very small field-effect gate field-effect modulation of less than 1% at 160 K. We do, however, detect

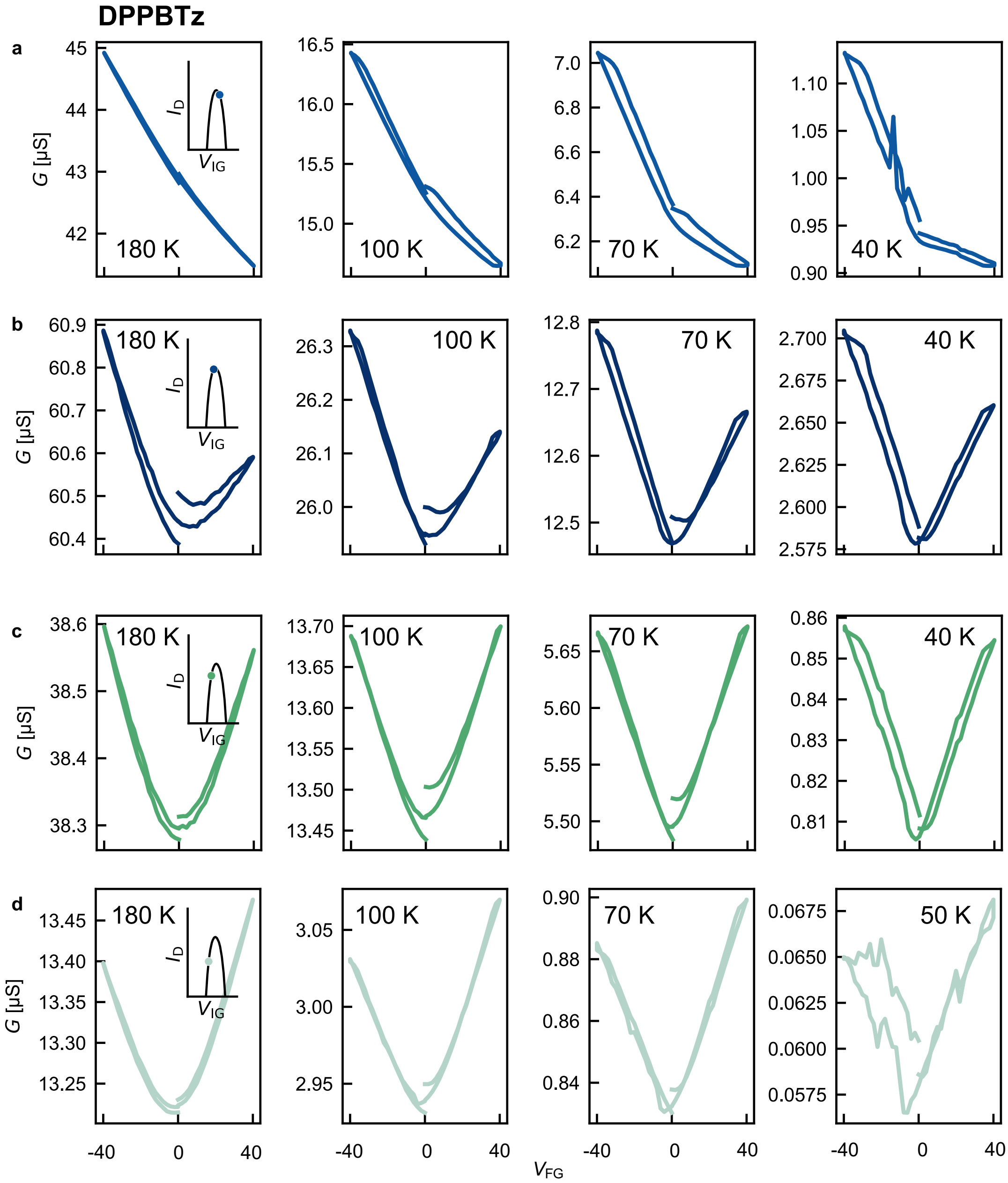

Supplementary Figure 37: **Temperature dependence of the field-effect gate transfer curve of DPP-BTz at various doping levels.**

a more substantial field-effect modulation for the lower conductivity states #1 (2 S cm^{-1}) and #2 (60 S cm^{-1}); for these states, we also see the transfer curve being slightly non-linear, suggesting the presence of non-equilibrium states analogous to IDT-BT and DPP-BTz.

The temperature dependence of the four-probe measurements of PBTTT's field-effect transfer curves and the associated extracted parameters are summarized in Supplementary Figures 38 and 39(b) respectively. As with IDT-BT and DPP-BTz the ratio of $\Delta G/G_0$ also increases at lower temperatures for PBTTT, as shown in the bottom panel of Supplementary Figure 39(b). For low conductivity states (e.g. states #1 and #2) the non-linearity — that is already present at 160 K — becomes stronger as temperature is decreased. Importantly, we observe the transfer curves of the highest doping states #3 and #4 also becoming non-linear at very low temperatures, more clearly seen at 50 K and below, as evident in Supplementary Figure 38. We observe a negative correlation between $\Delta G/G_0$ and conductivity, where higher doped samples show a more linear field-effect transfer curve at any temperature.

At first the trends seen in PBTTT might appear significantly different from DPP-BTz and IDT-BT, the transfer curves of which are already very much non-linear at 160 K. However, we recall that the gradual emergence of the non-linearity below a crossover temperature is in principle analogous to IDT-BT's behaviour as discussed in Supplementary Figure 28. PBTTT and IDT-BT, therefore, fundamentally only differ in their crossover temperatures, with the latter at much higher temperatures of ~ 260 K compared to the former's 50 K and below. To illustrate the point, in Supplementary Figure 40 we compare the $\Delta G/G_0$ ratios of IDT-BT's conductivity peak (similar to state #4) to the most doped state of PBTTT (similar to state #2 and #3). While the carrier densities of these IDT-BT and PBTTT states are the most comparable (1 and ~ 0.7 ions per monomer respectively, as discussed in Supplementary Note 3.4), we notice significantly higher $\Delta G/G_0$ ratios in IDT-BT. For instance, the highest ratio recorded in IDT-BT is 10^{-1} at around 50 K, while for PBTTT it is just about reaching 2×10^{-3} even at below 10 K.

Observation of the non-linearity in PBTTT's field-effect transfer curves provides strong evidence for the generality of non-equilibrium transport physics and the formation of a Coulomb gap in a very wide range of polymer systems. This is especially significant when considering that PBTTT differs from DPP-BTz and IDT-BT in many respects. We have seen, for instance, that PBTTT shows markedly different band filling physics due to its microstructure, electronic structure, and conductivity (Supplementary Notes 3 and 4). Here, we will argue that the main characteristic that sets PBTTT apart, with regards to the non-equilibrium transport physics, is its ability to host highly delocalized charge transport.

A Coulomb gap is a consequence of the *localization* of carriers in real-space as a consequence of some Coulombic, electrostatic interaction. Traditionally, this localization is associated with interactions between charges (*charge-charge interactions*), such as the ideas invoked in the initial conception of a Coulomb gap in the pioneering works of Efros and Skhlovskii.[36] Our theoretical calculations in Supplementary Note 8 show that an equally important Coulombic interaction contributing to the formation of the Coulomb gap in doped polymer semiconductors is the *charge-ion interaction*. To understand how important such *charge-charge* and *charge-ion interactions* are in specific systems, we invoke here the conceptual framework of the Mott-Hubbard model, in which *localization* (or 'correlation') is considered to arise from a competition between the carriers kinetic energy W as measured by the bandwidth and the *carrier-carrier interaction energy* U. In this model, W promotes delocalization, whereas U causes carriers to localize in a Mott-insulating state when $U >> W$. In the context of our doped conjugated polymers, we argue that fundamentally *delocalization* as represented by W plays a very similar role not just in relation to the interaction between charges, but also in relation to the interaction between charges and ions. Localization should be less pronounced in doped polymers when W is significantly larger than the *charge-ion interaction* energy.

We have established through various means that the ability to host delocalized transport, as represented by the bandwidth W, in PBTTT is much larger than in IDT-BT and DPP-BTz. Fundamentally, this can be thought as having a structural origin, where a more ordered structure allows extensive electronic coupling both along the backbone and the π-stacking direction.[37] Indeed, we have previously concluded that structural and dynamic disorder limit the achievable electrical conductivity in polymers.[6] PBTTT's low paracrystalline disorder affords much higher conductivity to be achieved compared to DPP-BTz and IDT-BT of comparable carrier densities.[6] From the experiments in the present study, while we see signatures of microscopic carrier delocalization in all three polymer systems in our Seebeck, ESR, and UPS measurements, we note that PBTTT is the only system showing any signature of macroscopic carrier delocalization on the length scale of a device. This is well evidenced by its much weaker temperature dependence of conductivity (Supplementary Note 3.2) and the observation of weak localization in our previous magnetoresistance experiments.[38] On the other hand, delocalization at extended length scales is typically prevented by stronger structural disorder in DPP-BTz and IDT-BT. In conclusion, our extensive studies, both previous and present, have consistently indicated a very delocalized transport in PBTTT, and therefore the formation of Coulomb gap in this highly crystalline system is the most unexpected between the three investigated polymers.

Our observation of the lower $\Delta G/G_0$ magnitudes in PBTTT is consistent with the more delocalized nature of its carriers. As carrier interaction is less effective in localizing the carriers, we expect the formation of

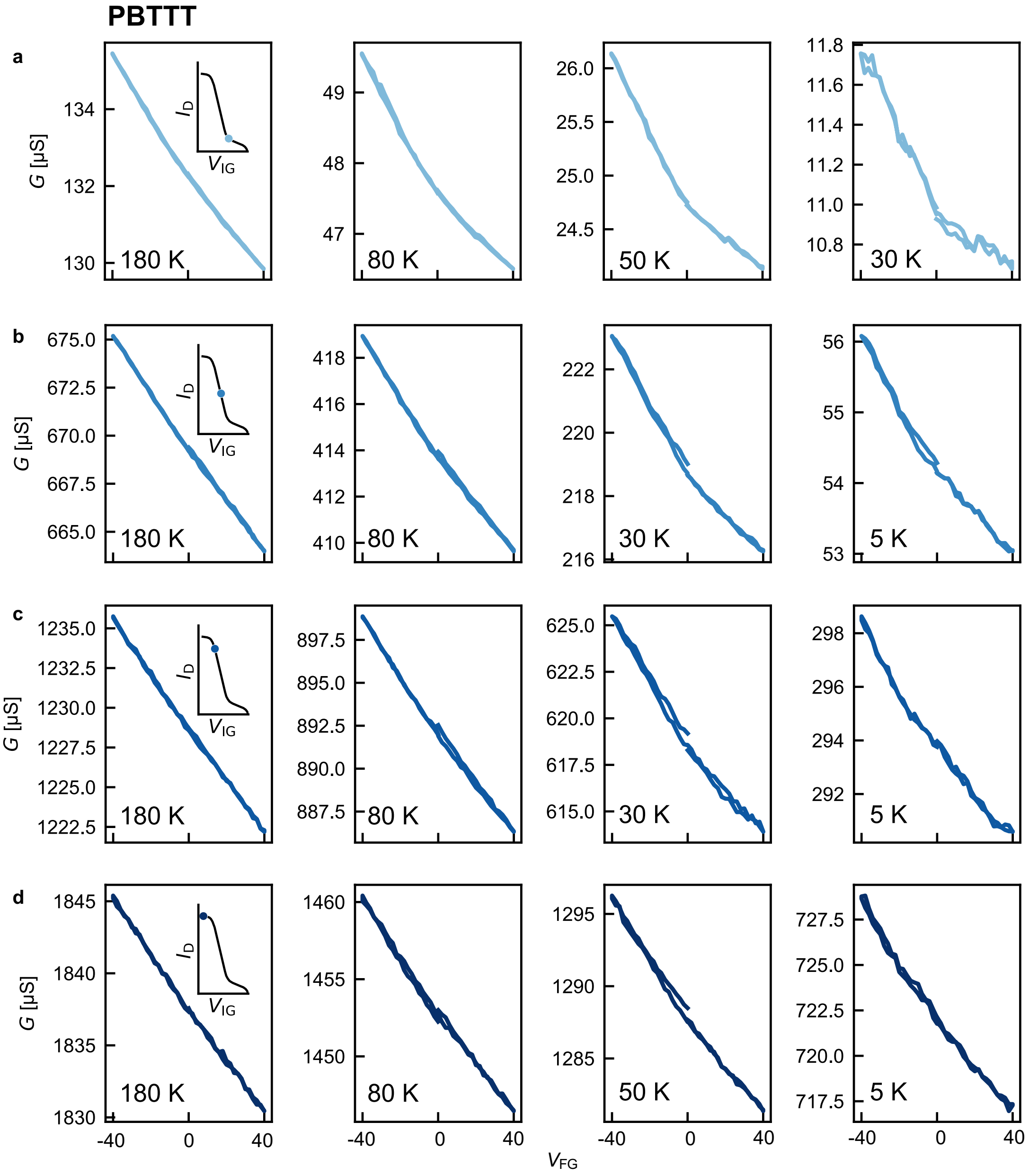

Supplementary Figure 38: **Temperature dependence of the field-effect gate transfer curve of PBTTT at various doping levels.**

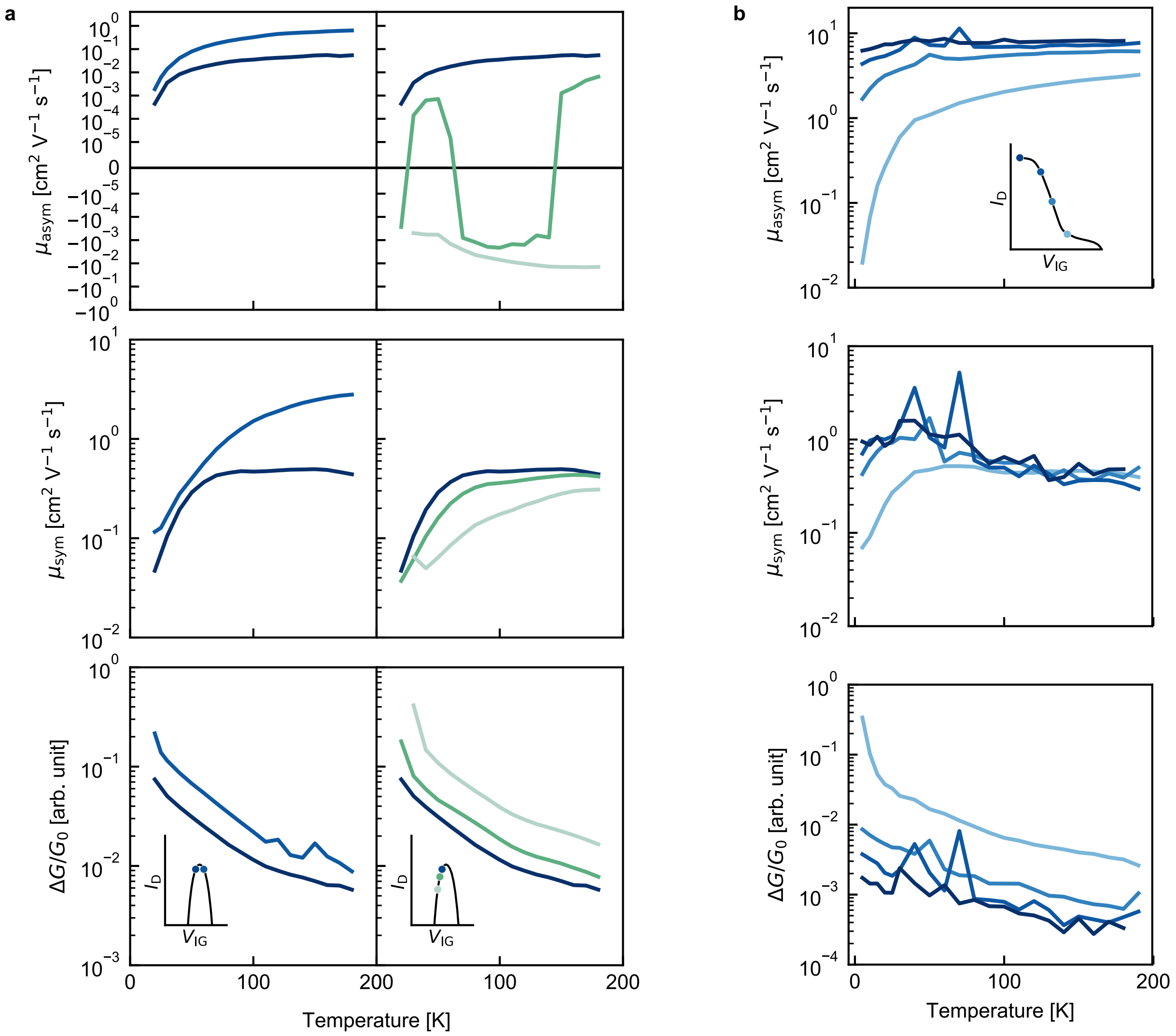

Supplementary Figure 39: **Parameters describing the temperature dependence of DPP-BTz and PBTTT field-effect gate transfer curves.** (Top panel) anti-symmetric mobility μ_{asym}, (middle panel) symmetric mobility μ_{sym}, and (bottom panel) $\Delta G/G_0$ ratio as a function of temperature for (a) DPP-BTz and (b) PBTTT. The parameters are extracted from the transfer curves shown in Supplementary Figures 37 and 38, as defined in Figure 2(c) of the main article.

a softer pseudogap. In such a pseudogap, the density-of-states at its Fermi level is reduced, but remains significantly greater than zero, whereas in the case of a harder pseudogap the pronounced interaction effects further reduce the density-of-states towards zero. The smaller observed $\Delta G/G_0$ is likely to be a reflection of a softer pseudogap, that is to say that there is a smaller difference between the density-of-states (and consequently conductance G) exactly at the Fermi level (when $V_{\mathrm{FG}} = 0$) and away from the bottom of the gap. This is compared to the harder pseudogap associated with higher $\Delta G/G_0$ in DPP-BTz and IDT-BT. This consideration suggests that measurements of the non-linear field-effect response could provide a simple method to compare the relative importance of Coulombic interactions and Coulomb gap formation on the charge transport properties of different conjugated polymers.

In the discussion so far we have focussed on the role of W, the other driving force for (de-)localization is of course the strength of the carrier-ion interaction energy, which may not be the same for the three polymers. It might be argued, that this energy is not likely to differ significantly, because the distances between the ions and polymer chains are to first order determined by the ion size, which is the same for the three systems. Furthermore, our X-ray data (Supplementary Notes 4.1, 4.2, 4.3, and Ref [6]) show that ions penetrate the interlamellar spaces of all polymer films, such that their distances to the mobile charges on the polymer backbones should be comparable. However, it is expected that in a more disordered system like IDT-BT some ions can become incorporated in configurations close to the backbone or intercalated into the π-stacks leading to stronger charge-ion interactions and driving the system towards localization than in a system like PBTTT, where the interlamellar sites into which ions become incorporated are well defined.

Similar arguments about delocalization can also be made to rationalize the observed doping level dependence of PBTTT's $\Delta G/G_0$. As the doping level is increased, the Coulomb potential wells of the ions spatially overlap, such that carriers become more delocalized on the smoothened potential landscape. An estimate for the point at which the transport is fully delocalized is provided by the Mott criterion,[39] $N_{\mathrm{d}}^{-1/3} R_{\mathrm{dop}} \sim 0.2$, where N_{d} is the doping level and R_{dop} the ion-charge distance. Importantly, as N_{d} increases, the system moves closer towards the threshold set by the Mott criterion and the potential well overlaps more. At low-to-moderate doping levels carriers are more likely to be localized, while carriers become increasingly delocalized as the dopant level is increased. As a consequence, it is expected that the same level of $\Delta G/G_0$ is reached only at a lower temperature in a more highly doped state, as seen in the bottom panel of Supplementary Figure 39(b). For the highly delocalized states of the highest doping levels (e.g. states #3 and #4), even at liquid helium temperatures we see smaller $\Delta G/G_0$ than the 180 K values of a lowly doped sample (e.g. state #1).

The apparent differences in the temperature at which the non-linear characteristics can be detected first, which is lowest in PBTTT and highest in IDT-BT, are likely to reflect both the structural dynamics of ion motion within the polymer host, as probed by the NMR experiments in Figure 2, and the width of the Coulomb gap in the DOS. The lattice dynamics of the polymer, in particular its glass transition temperature, and the molecular structure of the ion are likely to determine the temperature at which the ions become unable to rearrange in response to changes in carrier concentrations. On the other hand, a narrower, softer gap is likely to manifest itself at lower temperatures than a broader and harder gap feature. At the present stage we do not have a detailed understanding of the interplay between these two factors in the different polymer systems: we have only undertaken a detailed NMR characterisation of the ion dynamics in IDT-BT. For a full understanding detailed NMR studies on different polymer systems and dopant counterions are needed to fully understand the factors that govern at which temperature non-equilibrium effects can be observed in different systems. This goes beyond the scope of the present work.

Finally, we discuss the noteworthy features of the temperature dependence of PBTTT's mobilities. While the anti-symmetric mobility μ_{asym} of PBTTT displays a similar thermal activation, its value is still remarkably high even at liquid helium temperatures, i.e. 6 $\mathrm{cm}^2\ \mathrm{V}^{-1}\mathrm{s}^{-1}$ compared to a room temperature value of 8 $\mathrm{cm}^2\ \mathrm{V}^{-1}\mathrm{s}^{-1}$. This mirrors the temperature dependence of PBTTT's electrical conductivity, with the < 10 K values of both remaining greater than 50% than its 180 K value.

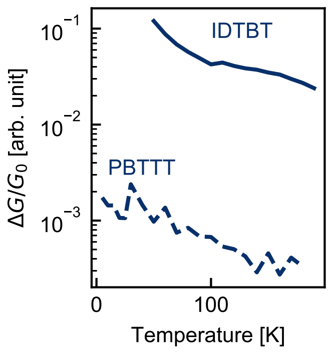

Supplementary Figure 40: **Temperature dependence of the $\Delta G/G_0$ ratio of PBTTT and IDT-BT with comparable doping levels.**

Supplementary Note 7 Nuclear Magnetic Resonance (NMR) spectroscopy

NMR spectroscopy probes nuclear spins through the Zeeman interaction under an applied magnetic field. In order to study the ion dynamics we perform NMR measurements on ^{19}F nuclei as they are spin 1/2 and exclusive to TFSI ions in the system of doped IDT-BT. By measuring the spin-lattice/longitudinal relaxation time (T_1), and the spin-spin/transverse relaxation/decoherence time (T_2) and their temperature dependence, the dynamic processes that lead to spin relaxation can be quantified. Details on the extraction of the relaxation times can be found in the Methods section at the end of the main text.

Figure 2(d) and Supplementary Figure 41 show that T_1 increases with decreasing temperature for all temperatures measured. This sets the upper limit of the correlation rates (that drive relaxation) to the Larmor frequency of ^{19}F (∼377 MHz) at these temperatures. Interestingly, T_2 shows three distinct regimes in its temperature dependence.

At high temperature (255-320 K), T_2 increases with increasing temperature. This is interpreted as motional narrowing and the relaxation times can be described within the Redfield theory:[40]

$$\frac{1}{T_1} = \gamma(B_x^2 + B_y^2)\frac{\tau_C}{1+\omega_L^2\tau_C^2}, \tag{3}$$

and:

$$\frac{1}{T_2} = \frac{1}{2T_1} + \gamma^2 B_z^2 \tau_C, \tag{4}$$

where γ is the nuclear gyromagnetic ratio, $B_{x,y,z}$ represent the Cartesian components of the local field strengths with the z-axis defined by the direction of the applied magnetic field B_0, $\omega_L = \gamma B_0$ is the Larmor frequency of the nucleus, and τ_C is the correlation time of the magnetic field fluctuations (reciprocal of correlation rate). Essentially, T_1 spin-lattice relaxation is driven by stochastic local field fluctuations experienced by the TFSI molecules as they move within the polymer matrix, that are most effective in inducing spin flips when the correlation rate matches the Larmor frequency. In this motional narrowing regime T_2 increases monotonically with increasing motion rate because the motion is fast enough to average effectively over individual magnetic field environments.

As the motion slows at low temperatures, local fields start to act as static fields and induce spin precession around locally varying magnetic field axis. In this inhomogeneous broadening regime, in which the lineshape is no longer Lorentzian, Supplementary Equation 4 fails to accurately describe the relaxation behavior and the expression needs to be adapted:[41, 42]

$$T_2' = \tau_C + \frac{1}{\gamma B_z} + \frac{1}{\gamma^2 B_z^2 \tau_C}, \tag{5}$$

where,

$$\frac{1}{T_2} = \frac{1}{2T_1} + \frac{1}{T_2'}. \tag{6}$$

In the inhomogeneous broadening regime T_2' is determined by the time spent at each local field site, plus the average time taken for the spin to lose its initial phase through precession (first two terms of Supplementary Equation 5). The third term acts to recover the behavior described in Supplementary Equation 4 at the fast motion limit. In the slow motion limit, T_2 will therefore increase with decreasing motion rate. This is observed experimentally in the intermediate temperature regime between 215-255 K, where a decrease in temperature results in an increasing T_2.

The experimental relaxation behavior in Figure 2(d) and Supplementary Figure 41, at temperatures above 215 K, can be described by Supplementary Equations 3 and 6. However, below this temperature, T_2 again decreases with decreasing temperature suggesting that we enter a second, low-temperature motional narrowing regime, where once again the T_2 behavior is captured by Supplementary Equation 4. This is interpreted as reflecting different types of molecular motion that govern spin relaxation of the ^{19}F nuclei at high and low temperatures.

We hypothesize that the low temperature motional narrowing is governed by fast intra-molecular motions, such as bond rotations/librations of the TFSI ion, while at high temperatures the relevant motion is the slower translational diffusion of the ions within the polymer matrix. These two relaxation pathways are combined as:

$$\frac{1}{T_{1,2}} = \frac{1}{T_{1,2}^S} + \frac{1}{T_{1,2}^F}, \tag{7}$$

where $T_{1,2}^S$ are the relaxation times for the slower motion that is the dominant relaxation pathway at high temperatures and $T_{1,2}^F$ are the relaxation times for the faster motion that dominates low temperatures. In order to fit the experimental data to the model, fluctuating fields are taken to be isotropic $(B_x = B_y = B_z)$ and a simple Arrhenius relationship is assumed, relating temperature to correlation times:

$$\tau_C = \tau_0 \exp \frac{E_a}{k_B T}, \tag{8}$$

where τ_0 is the correlation time at infinite temperature, k_B is the Boltzmann's constant, and E_a is the activation energy.

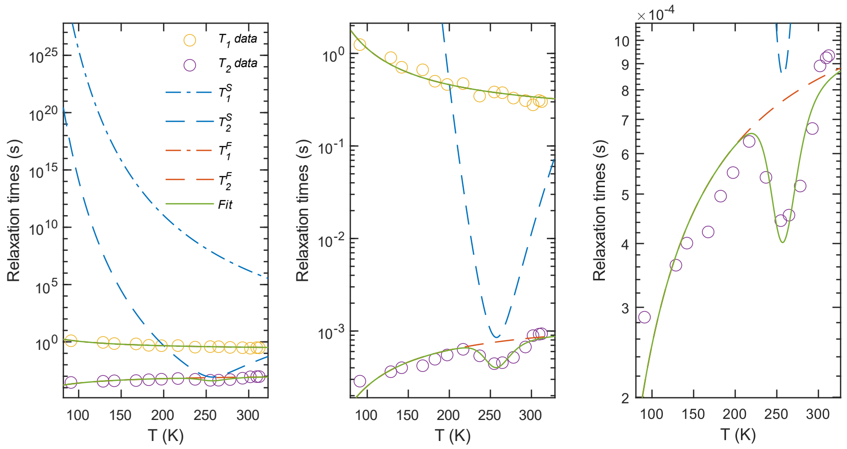

Supplementary Figure 41: [19]**F Nuclear Magnetic Resonance relaxation times fit.** The results of the NMR fitting using the model described above, with τ_0, E_a, and B as fitting parameters for each relaxation pathway in the model. Fitting results are given in Supplementary Table 4. Correlation times extracted are plotted against temperature in Figure 2.

Supplementary Table 4: NMR relaxation times fit results.

	τ_0	E_a	B
Slow motion	1.72×10^{-15} s	0.574 eV	8.8×10^{-5} T
Fast motion	6.57×10^{-9} s	15.5×10^{-3} eV	7.85×10^{-3} T

Supplementary Note 8 Theory of non-equilibrium transport phenomena

8.1 Model Hamiltonian

The electronic structure of doped polymers is described with a model Hamiltonian, defined on a two-dimensional (2D) lattice describing π-stacked polymer chains. The proposed model extends the one proposed in our recent work,[6] and includes disorder, electron-electron interactions, and the mutual interaction between electrons and the interaction with the ions introduced upon electrochemical doping. The system Hamiltonian can be written as sum of three terms:

$$H = H_{\mathrm{el}} + H_{\mathrm{ion}} + H_{\mathrm{el-ion}}. \tag{9}$$

The electronic system is described by an extended Hubbard Hamiltonian with long-range Coulomb interactions:

$$H_{\mathrm{el}} = \sum_i \epsilon_i \, \hat{n}_i + \sum_{i,j,\sigma} t_{ij} \, c^\dagger_{i\sigma} c_{j\sigma} + U \sum_i \hat{n}_{i\alpha} \hat{n}_{i,\beta} + \frac{1}{2} \sum_{i,j} V_{ij} \, \hat{n}_i \hat{n}_j, \tag{10}$$

where $c^\dagger_{i\sigma}$ ($c_{i\sigma}$) creates (annihilates) an electron with spin σ at site i, $\hat{n}_{i\sigma} = c^\dagger_{i\sigma} c_{i\sigma}$ and $\hat{n}_i = \hat{n}_{i\alpha} + \hat{n}_{i\beta}$; ϵ_i are the site energies (including the effect of dopant ions, see below), t_{ij} are the charge transfer integrals between nearest-neighbor sites, U is the on-site Hubbard's repulsion, and $V_{ij} = v(\mathbf{r}_i - \mathbf{r}_j)$, where $v(\mathbf{r}) = (\varepsilon_r |\mathbf{r}|)^{-1}$ and ε_r is the dielectric constant.

We aim to solve the Hamiltonian in Supplementary Equation 10 for 2D cells of hundreds of sites (i.e. a large supercell of the primitive cell) with periodic boundary conditions, accounting for energetic disorder affecting site energies and charge transfer integrals. We hence opt for a Hartree-Fock (spin-restricted) treatment of electron-electron interactions, leading to the effective single-particle Hamiltonian:

$$\begin{aligned} H^{\mathrm{HF}}_{\mathrm{el}} &= \sum_i \epsilon_i \, \hat{n}_i + \sum_{i,j} t_{ij} \, c^\dagger_i c_j \\ &+ \sum_i \left[\sum_k P_{kk} (U + \widetilde{V}_{ik}) \right] \hat{n}_i - \frac{1}{2} \sum_{i,j} P_{ij} \, \widetilde{V}_{ij} \, c^\dagger_i c_j \end{aligned} \tag{11}$$

that self-consistently depends on the density operator $\hat{P}$.[43] In the presence of long-range Coulomb interactions with periodic replica, the inter-site term reads:

$$\widetilde{V}_{ik} = \sum_l v(\mathbf{r}_k - \mathbf{r}_i - \mathbf{T}_l), \tag{12}$$

where the sum extends over the translation vectors $\mathbf{T}_l$ of the supercell, excluding the term $\mathbf{T}_l = \mathbf{r}_k - \mathbf{r}_i = 0$. The two-dimensional (2D) lattice sums in Supplementary Equation 12 have been computed with the Ewald method.[44]

Charge-neutralizing counterions that are introduced in the polymer film by electrochemical doping typically insert into the alkyl region, close to the plane of π-stacked conjugated chains.[6] We model the electrostatic potential of the ions in terms of classical point charges, resulting in an ionic contribution to site energies:

$$H_{\mathrm{ion}} = \sum_i \epsilon^{(\mathrm{ion})}_i \, \hat{n}_i, \tag{13}$$

where:

$$\epsilon^{(\mathrm{ion})}_i = \sum_{n,l} v(\mathbf{r}_i - \mathbf{R}_n - \mathbf{T}_l). \tag{14}$$

$\epsilon^{(\mathrm{ion})}_i$ can be calculated for a given set of ionic positions $\mathbf{R}_n$, entering the electronic Hamiltonian as an external inhomogeneous field, contributing to the energetic disorder on site potentials. As shown in a previous work, the effect of the electrostatic potential of dopant ions is a minor one,[6] so that in the present study this is implicitly incorporated in the diagonal disorder.

For the scope of the present work, it is actually more important to consider the response of the ions to the field of the electrons on polymer chains. This can be described assuming small ionic displacements $\mathbf{X}_m$ around the equilibrium positions in the absence of electron-ion interaction, $\mathbf{R}_m$. The electron-ion Hamiltonian reads:

$$H_{\mathrm{el-ion}} = \frac{K}{2} \sum_m |\mathbf{X}_m|^2 - e \sum_{im} \mathbf{g}_{im} \cdot \mathbf{X}_m \, \hat{n}_i, \tag{15}$$

where e is the elementary charge, K is the force constant, describing the restoring potential in the harmonic approximation, and:

$$\mathbf{g}_{im} = \sum_l \nabla v(\mathbf{r}_i - \mathbf{R}_m - \mathbf{T}_l) \tag{16}$$

is the linear coupling constant between ionic displacements and electrons.

The equilibrium ionic displacements can be computed upon differentiating the ground state energy with respect to displacements:

$$\mathbf{X}_m^{(\mathrm{eq})} = \frac{e}{K} \sum_j \mathbf{g}_{jm} \langle \hat{n}_j \rangle \tag{17}$$

and then plugged into Supplementary Equation 15 to obtain the electron-ion Hamiltonian at equilibrium ionic displacements:

$$\begin{aligned} H_{\mathrm{el-ion}}^{(\mathrm{eq})} &= -\frac{e^2}{K} \sum_i \left(\sum_{jm} \mathbf{g}_{im} \cdot \mathbf{g}_{jm} \langle \hat{n}_j \rangle \right) \hat{n}_i \\ &+ \frac{e^2}{2K} \sum_m \left| \sum_j \mathbf{g}_{jm} \langle \hat{n}_j \rangle \right|^2 . \end{aligned} \tag{18}$$

The response of the ions to π-electrons on the polymer chains finally results in a self-consistent term in the system Hamiltonian, which adds up to the self-consistent one resulting from the mean-field treatment of electron-electron interaction. The second term in Supplementary Equation 18 is a constant that can be disregarded.

The self-consistent term arising from electron-ion interaction is nonlocal in nature, i.e. the potential generated at a given site depends on the charge density on all other sites. It has been shown that similar long-range non-local terms can be mapped into an effective local interaction.[45, 46, 47] For the sake of simplicity, and to avoid the proliferation of parameters, a local approximation is adopted also here, leading to:

$$H_{\mathrm{el-ion}}^{(\mathrm{eq})} = -2\lambda \sum_i \langle \hat{n}_i \rangle \hat{n}_i,$$

where λ is the relaxation energy that is the single parameter measuring the strength of the coupling between electrons and ions. Within this framework, the ionic degrees of freedom are mapped onto a set of site coordinates linearly coupled to the site charge operator (Holstein model).

8.2 Model parameters and computational details

Calculations have been performed on large supercells of a 2D square lattice. The model parameters are chosen as to describe IDT-BT, and follow from the *ab initio* results reported in Supplementary Note 5. Accordingly, a single-band model with one-electron per site is adopted to describe this system in Regimes I and II. The full list of parameters employed in the calculations is provided in Supplementary Table 5. The electronic model accounts for diagonal and off-diagonal Gaussian disorder and strongly anisotropic hoppings. IDT-BT is known to be a disordered polymer with a strong resilience towards torsional disorder.[48] We hence adopt intra-chain hoppings with mean values set by the DFT bandwidth (Supplementary Figure 25) and relatively-low fluctuations (20%) associated with torsional disorder. Fluctuations of the same magnitude and with zero mean are assumed for inter-chain hoppings. The value for the Hubbard U is set to an intermediate value between those calculated for the entire IDT-BT repeat unit and the IDT fragment mostly participating to the valence band (see Supplementary Table 3). The relaxation energy is an empirical parameter that is set to $\lambda = 0.2$ eV, which is the smallest value that produces appreciable changes in the DOS and, consequently, that determines the pinning of the Coulomb gap when E_F is tuned with the field-effect gate bias. Other plausible values of λ (e.g. half or double its value) do not change qualitatively the results. The other parameters are set to typical values for conjugated polymers.

Calculations employed supercells with translational vectors $\mathbf{T}_1 = a(n_{xy}\hat{\mathbf{x}} - \hat{\mathbf{y}})$ and $\mathbf{T}_2 = a(\hat{\mathbf{x}} + n_{xy}\hat{\mathbf{y}})$, where $\hat{\mathbf{x}}$ and $\hat{\mathbf{y}}$ are the Cartesian unit vectors. Such a square-tilted supercell combines the proper handling of the isotropic long-range Coulomb interactions, with an optimal sampling of the Brillouin zone of the disorder-free system. Results presented herein have been obtained for supercells of 170 sites, corresponding to $n_{xy} = 13$.

All the results reported in this work have been obtained as averages over 400 disorder realizations. For each realization, the eigenstates of the mean-field Hamiltonian are expressed as linear combinations of site orbitals:

$$|n\rangle = \sum_k c_{kn}\, \phi_k, \tag{19}$$

Supplementary Table 5: Model parameter values employed in the calculations.

Parameter	Value	Note
a	5 Å	square lattice spacing
ϵ	0 ± 0.20 eV	site energies (mean $\pm$ stdev)
$t_{\|\|}$	0.25 ± 0.05 eV	intra-chain hopping (mean $\pm$ stdev)
$t_{\perp}$	0 ± 0.05 eV	inter-chain hopping (mean $\pm$ stdev)
U	1.8 eV	Hubbard repulsion
ε_r	4	dielectric constant
λ	0.2 eV	ionic relaxation energy

and have energy E_n. The delocalization of charge carriers is quantified by the participation ratio:

$$L_n = \frac{1}{\sum_i |c_{in}|^4}, \tag{20}$$

which measures the number of sites over which wavefunctions extend. The conductivity is calculated within the framework of the transient localization theory in the relaxation time approximation (RTA).[49, 50] The dc conductivity reads:

$$\sigma_\alpha = \frac{\pi}{\hbar v} \sum_{mn}{}' (1 - f_m)(1 - f_n) \, \frac{e^{-\beta\epsilon_m} - e^{-\beta\epsilon_n}}{E_n - E_m} \, |\langle n|\hat{J}_\alpha|m\rangle|^2 \, \Lambda(E_n - E_m; 0, \gamma), \tag{21}$$

where $\epsilon_m = E_m - E_F$, $f_n = f(E_n)$ being f the Fermi function, $\hat{J}$ is the current operator and $\Lambda(E; 0, \gamma)$ is a zero-centered Lorentzian function with half-width at half-maximum γ, which corresponds to the typical energy scale of vibrational motion ($\gamma = 10$ meV is used throughout this work); $\hbar$ is the reduced Planck constant, v the system volume, $\beta = 1/(k_B T)$, k_B the Boltzmann constant, and T the absolute temperature set to 300 K. The subscript α labels Cartesian components of which we report the average $\sigma = (\sigma_x + \sigma_y)/2$.

Hartree-Fock calculations have been performed at some fixed number of electrons (canonical ensemble), the charge density being controlled by electrochemical doping and by the field-effect gate bias. Electrochemical p-type doping is modeled as the removal of electrons (addition of holes) to the full band of the pristine system. This charge is counterbalanced by the introduction of an equal amount of negatively charged ions, ensuring charge neutrality. The level of electrochemical doping is hence measured in ions per monomer as in the experiments, ranging from 0 (pristine system) to 2 ions/monomer (fully depleted band) in our single-band model. The effect of the field-effect gate bias is described by introducing or removing electrons, leading to a charged system; this resembles the condition of the field-effect channel locally around the polymer/dielectric interface. The charge density modulation achieved by field-effect gate bias is measured in terms of the relative change in the charge density $\Delta n/n_0$, where Δn is the variation in the number of electrons due to the bias and n_0 is the number of electrons in the full band (pristine system). We note that the charge density modulation introduced with the field-effect gate bias are typically two orders of magnitude smaller than those achieved by electrochemical doping; this nevertheless result in large variations in the conductivity, in agreement with our experiments.

In this work we report sets of results obtained according to different treatments of the ionic relaxation mimicking the different protocols used in the experiments, as described next. Calculations not accounting for ionic relaxation are obtained by setting $\lambda = 0$. In the ion-relaxation equilibrium scheme, the self-consistent term in Supplementary Equation 19 is included and updated during the iterative solution of the Hartree-Fock Hamiltonian. In this scheme, the solution of the electronic problem is obtained in the self-consistent field given by electron-electron interaction and ion relaxation at any given charge density determined by the gate bias and electrochemical doping. Finally, tuning the field-effect gate bias in the non-equilibrium scheme corresponds to a self consistent problem for the electrons only, while the reaction field of the dopant ions is kept frozen to the one previously computed in the zero-bias equilibrium calculation.

8.3 Supplementary calculation results

Supplementary Figure 42 shows a set of DOSs computed for systems at various electron densities, ranging from weakly doped (0.05 ions/monomer doping), to intermediate doping (0.4, 0.8, 1.2 and 1.6 ions/monomer doping), to almost completely filled bands (1.95 ions/monomer doping). At zero field-effect gate bias ($\Delta n/n_0 = 0$,

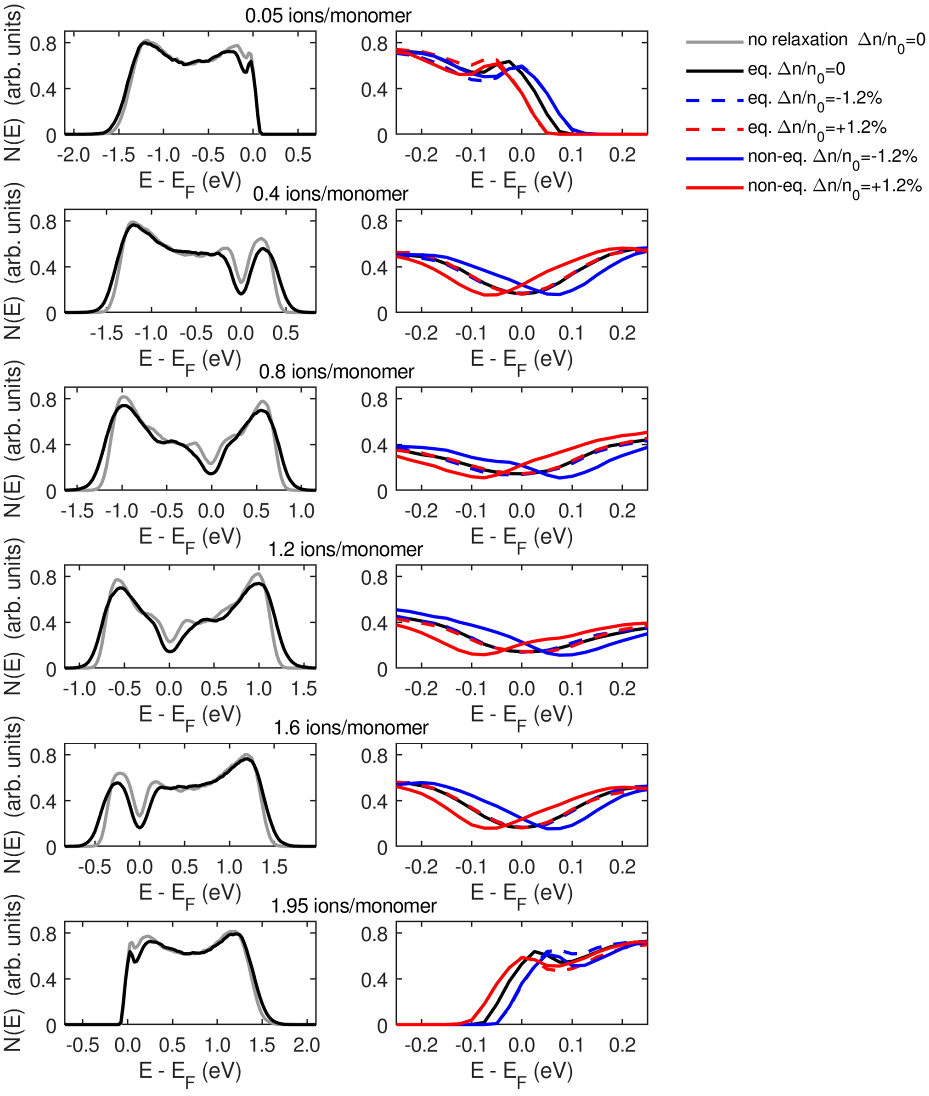

Supplementary Figure 42: **Density of states (DOS, $N(E)$, in arbitrary units) calculated at various electrochemical doping levels.** Left-hand panels show the DOS in the absence of field-effect gate bias ($\Delta n/n_0 = 0$), with ($\lambda = 0.2$ eV) and without ($\lambda = 0$) ionic relaxation. Right-hand panels show a zoomed-in view of the DOS around E_F, including results at finite gate biases (red/blue lines) obtained in the equilibrium (dashed line) and non-equilibrium (solid line) schemes. Note that in the present figure, all the DOSs are plotted against $E - E_F$, i.e. zero energy corresponds to the Fermi energy specific to each system. This differs from Figure 3(e) in the main manuscript, where the DOSs minima are aligned to a common energy.

left-hand column panels), all studied cases exhibit a suppression of the DOS at E_F due to electron-electron interactions (gray line), that is amplified by ionic relaxation (black line). This effect is more important at intermediate band fillings, resulting in the opening of a broader pseudogap.

At finite field-effect gate bias (Supplementary Figure 42, right-hand column panels, blue and red lines), equilibrium and non-equilibrium calculations (dashed and solid lines, respectively) lead to nearly indistinguishable values of the DOS at E_F for the 0.05 and 1.95 ions/monomer doped systems. At intermediate fillings, instead, the two treatments of ionic relaxation yield qualitatively different results. In the equilibrium scheme the DOSs calculated for different values of $\Delta n/n_0$, when reported with respect to the Fermi level specific to each system, are all superimposed on each other. This is due to the fact that both electron-electron interactions and ionic relaxation cooperate in creating the pseudogap at E_F, i.e. the DOS minimum remains tied to E_F as Δn is varied. Conversely, in the non-equilibrium scheme the DOS minima are offset from E_F at finite $\Delta n/n_0$. This results from the contribution of ionic relaxation being frozen to the zero-bias solution ($\Delta n/n_0 = 0$).

Supplementary Figure 43 shows an analogous behavior for the energy-dependent participation ratio $L(E)$, which measures the degree of delocalization of the electronic wavefunctions as a function of their energy. At

zero field-effect gate bias ($\Delta n/n_0 = 0$, left-hand panels) the participation ratio of states at the Fermi level is suppressed, reflecting the more localized nature of states in the Coulomb gap.[6] Such a localization is further enhanced by ionic relaxation (see black vs. gray curve, see legend). In the presence of a finite field-effect gate bias, $L(E)$ depends on whether equilibrium or non-equilibrium scheme is adopted for ionic relaxation, especially at intermediate band filling (0.4-1.6 ions/monomer), similar to what occurs to the DOS (see Supplementary Figure 42). In these cases, higher L values are attained at E_F upon gating in the non-equilibrium scheme, indicative of more delocalized (more mobile) charge carriers.

The conductivity dependence on field-effect gate bias is shown in Supplementary Figure 44 (left-hand panels), calculated at various levels of electrochemical doping, together with the DOS ($N(E_F)$, central panels) and participation ratio ($L(E_F)$, right-hand panels), both evaluated at E_F. All quantities are reported for ion reaction treated in the equilibrium (purple) and non-equilibrium (orange) schemes. At ultra-low and ultra-high filling (0.05 and 1.95 ions/monomer) the conductivity dependence presents a monotonic dependence of the bias-induced electron density, $\Delta n/n_0 = 0$. The conductivity increases as the gate bias shifts the Fermi level away from band edges, resulting in a higher DOS and more delocalized states. Indeed, the conductivity correlates well with both the DOS $N(E_F)$ and the participation ratio $L(E_F)$. Moreover, the results obtained from the equilibrium and non-equilibrium schemes are similar.

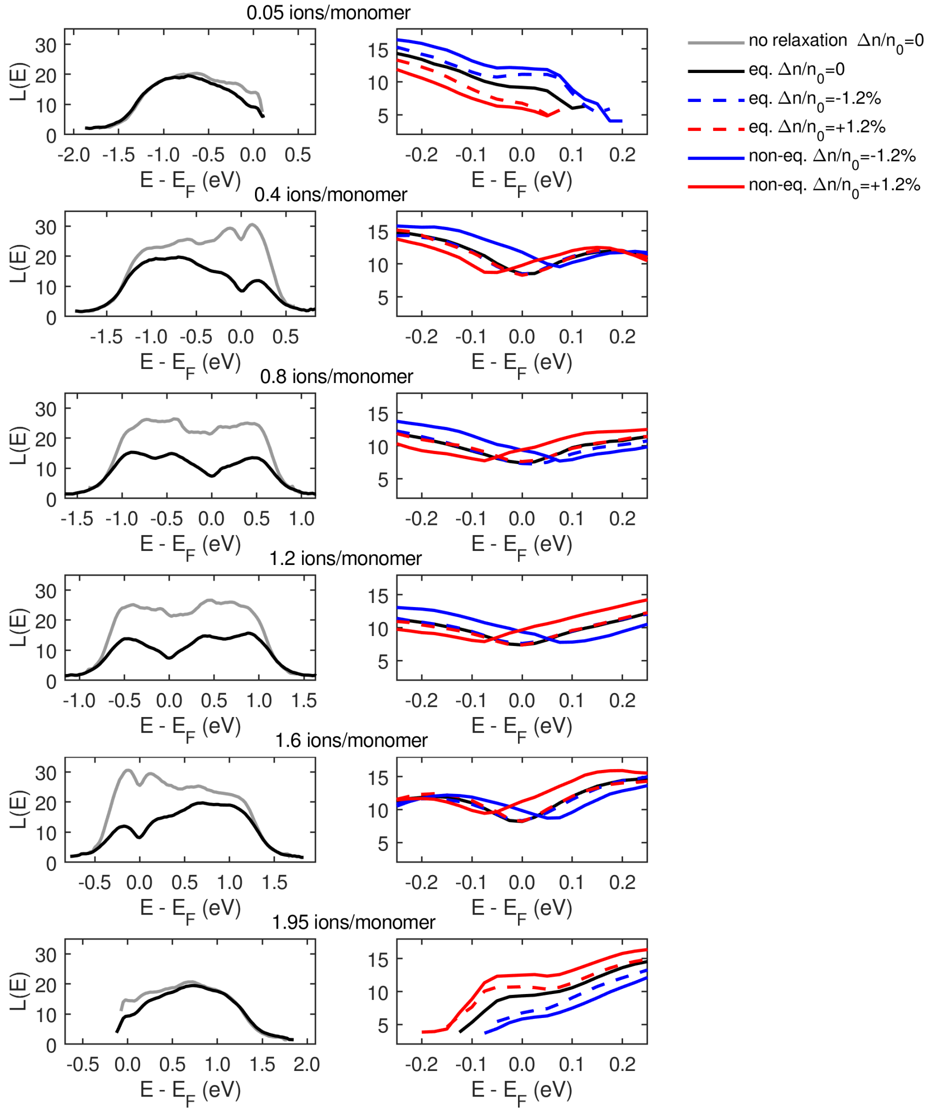

Supplementary Figure 43: **Energy-dependent participation ratio $L(E)$ calculated at various electrochemical doping levels.** Left-hand panels show $L(E)$ in the absence of field-effect gate bias ($\Delta n/n_0 = 0$), with ($\lambda = 0.2$ eV) and without ($\lambda = 0$) ionic relaxation. Right-hand panels show a zoomed-in view of $L(E)$ around E_F, including results at finite gate biases (red/blue lines) obtained in the equilibrium (dashed line) and non-equilibrium (solid line) schemes.

At intermediate doping levels (0.4-1.6 ions/monomer) the conductivity shows a pronounced ambipolar behavior against field-effect gate bias only in the non-equilibrium ion relaxation scheme. Conversely, under equilibrium conditions the conductivity does not exhibit a clear trend with gate bias in this regime. The strong non-equilibrium ambipolar behavior is seen to originate from both the increased number of conducting states (larger $N(E_F)$) and their increased delocalization (larger $L(E_F)$). In line with our experimental observations (see Figure 2(b) in the main manuscript and Supplementary Figure 26), the ambipolar behavior is symmetric with respect to the gate bias around half filling (0.8 and 1.2 ions/monomer) and it acquires some asymmetry at 0.4 and 1.6 ions/monomer. Our calculations ascribe this asymmetry to the more localized nature of carriers towards the band edges: $L(E_F)$ evolution with the gate bias is slightly anti-symmetric at 0.4 and 1.6 ions/monomer, while the trend of $N(E_F)$ remains symmetric.

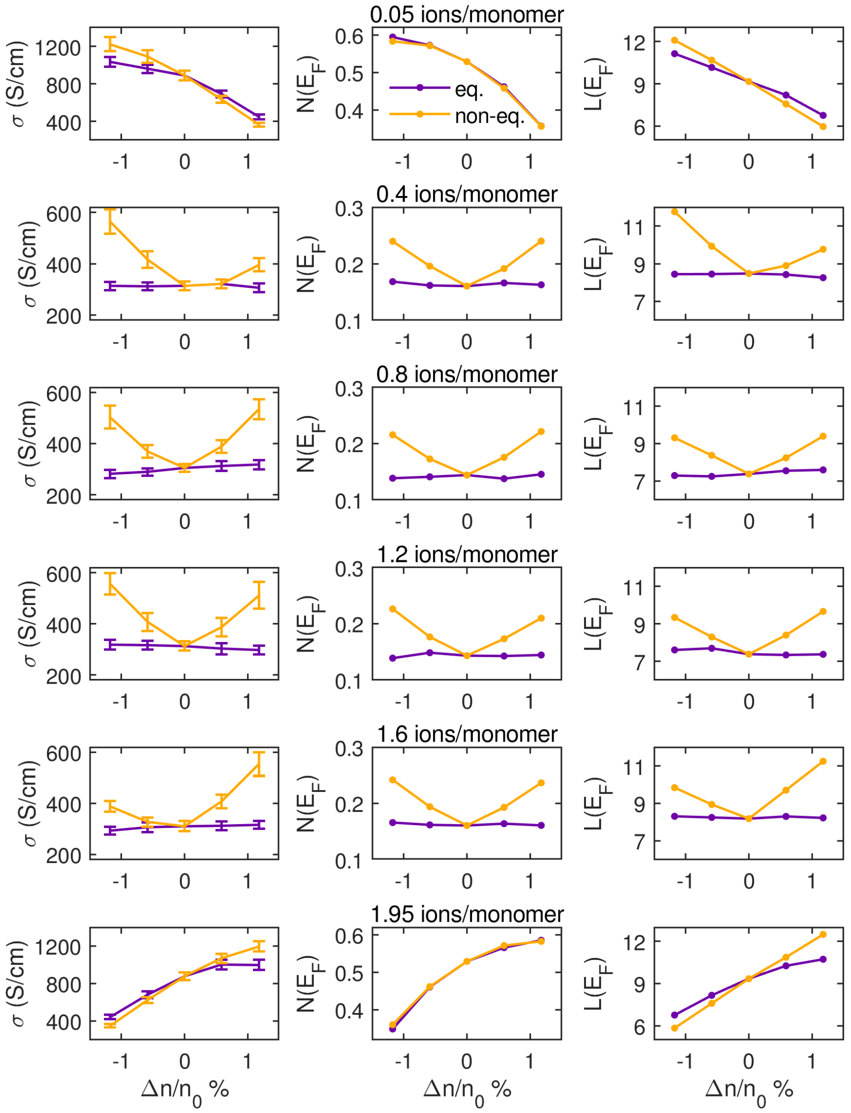

Supplementary Figure 44: **Conductivity (left), DOS (middle) and participation ratio (right) as a function of the field-effect gate bias for various levels of electrochemical doping.** Purple and orange lines refer to equilibrium and non-equilibrium calculations. DOS and participation ratio are both evaluated at the Fermi level. Data are presented as the mean of 400 realizations ± standard error of the mean.

Supplementary Note 9 Infrared Charge Modulation Spectroscopy (CMS)

Infrared charge modulation spectroscopy (CMS) is a method for measuring the absorption spectra of polaron states in field-effect transistors that has been applied routinely in the literature of organic semiconductors as a probe of charge carrier delocalization.[51, 37, 52] To the best of our knowledge the technique has not previously been employed in the study of highly doped organic semiconductors. These measurements are performed as an *operando* I-V and spectroscopic measurement using a Fourier-transform infrared (FTIR) spectrometer: we modulate the carrier density in the material using a field-effect gate and measure the corresponding change in infrared absorption. Due to the low density of charges induced by field effect, the signal intensity in a typical CMS experiment is extremely small (Δ Abs $< 10^{-3}$ OD), which necessitates careful experimental conditions.

9.1 Device considerations

Our CMS devices are bottom-gate, bottom-contact architecture, consisting of a 4 by 4 mm^2 channel formed by Cr/Au electrodes on a 300 nm SiO_x / Si wafer (device structure shown in Supplementary Figure 2). To ensure high IR transmission through the Si substrate, we use high resistivity (> 10 kΩ cm) intrinsic float-zone wafers. The high resistance of these substrates results in a large RC time constant that limits the switching speed of our devices, typically requiring approximately 20 seconds to achieve a stable gate current at low temperatures (150 - 190 K). This charging time in turn defines the maximum rate at which the carrier density can be modulated. As we will discuss below, operating near this maximum frequency is critical to minimizing noise. In practice, we typically collected 128 FTIR scans per modulation step, requiring 25-30 seconds.

As in all field-effect measurements we report, minimizing gate leakage is critical to observing the non-linear transfer characteristics shown in Supplementary Figure 28. At low temperatures, typical drain currents in our measurements are on the order of 100 nA. The gate-modulated changes in gate current are typically on the order of 1% of the drain current, approx. 1 nA, implying that gate leakage should ideally be no more than a few hundred pA. This condition was verified throughout each measurement.

Ensuring a stable doping level is also critical due to the long measurement times ($>$ 12 hours per temperature) and the dependence of the polaron band shape on doping level. Empirically, we observe that ion-exchange doped films typically display somewhat better stability than electrochemically doped films, motivating their use in these devices. We note that the dopant counter-ion TFSI is identical for electrochemically doped samples and ion-exchange doped samples.[5] Stability is further enhanced by coating the film with a 25 nm CYTOP-S layer, which is sufficiently thin to avoid any interference effects. Last, we find that stability is maximized by pumping the optical cryostat housing the sample to nearly the base pressure ($\sim 10^{-6}$ mBar) before starting the helium flow, and collecting data at warmer temperatures first. These steps minimize condensation of water or other impurities on the sample surface, which appear to mediate dedoping. These steps result in a drain current that is stable to within a few percent over the course of the measurement, indicating that both doping level and temperature remain sufficiently stable over the full experimental timeframe.

9.2 Spectroscopic measurement considerations

The broad lineshape of charge-induced absorption bands in polymers implies that our spectroscopic measurements require a very flat and stable baseline. We take several steps to ensure this. In an FTIR measurement, the intensity seen by the detector is extremely low during nearly the entire scan, with the exception of a handful of datapoints very close to the zero path length difference (ZPD) crossing of the interferometer. To obtain a spectrum, we Fourier transform around the ZPD. At ZPD, the interferometer passes the full intensity of the light source to the detector, while at other positions only a small fraction of frequencies are passed. If the detector response is non-linear, this non-linearity will most strongly affect datapoints near the ZPD, where the light intensity is highest. After Fourier transforming the data, these nonlinearities lead to baseline distortions. High-sensitivity IR detectors, such as mercury cadmium telluride (MCT) show intrinsically non-linear response [53] and therefore are not suitable for measurements of broad polaron bands typical in doped polymers. In our CMS measurements we therefore use a deuterated L-alanine doped triglycene sulphate (DLaTGS) detector, which is considerably less sensitive but provides a highly linear response.

FTIR spectrometers are typically constructed as single-beam instruments, meaning that there is no reference beam capable of detecting fluctuations in the light source or detector responsivity. As a result, drift in the temperature of the source or detector can lead to variation of the baseline over time. In a CMS experiment, these effects are cancelled by measuring the difference between gate-on and gate-off absorption. However, baseline drift on the timescale of the modulation cycle will still appear as an apparent signal. Although this drift is quite small on absolute terms, it represents the dominant source of noise in our CMS measurements.

To minimize this error, we therefore need to maximize the modulation rate in our experiments. As discussed above, the modulation rate is limited by the RC time constant of the gate to ∼30 s. Additionally, we find that using faster interferometer scan rates and collecting data at lower resolution both improve baseline stability, presumably because both of these parameters result in more scans per second, and passing through ZPD more often leads to a more stable detector temperature. Together, our optimized experimental conditions (5 kHz scan rate, 16 cm^{-1} resolution, 128 scans per modulation cycle) lead to baseline errors on the order of 10^{-5}, approximately 50 times smaller than the measured CMS signal intensity.

9.3 Data analysis

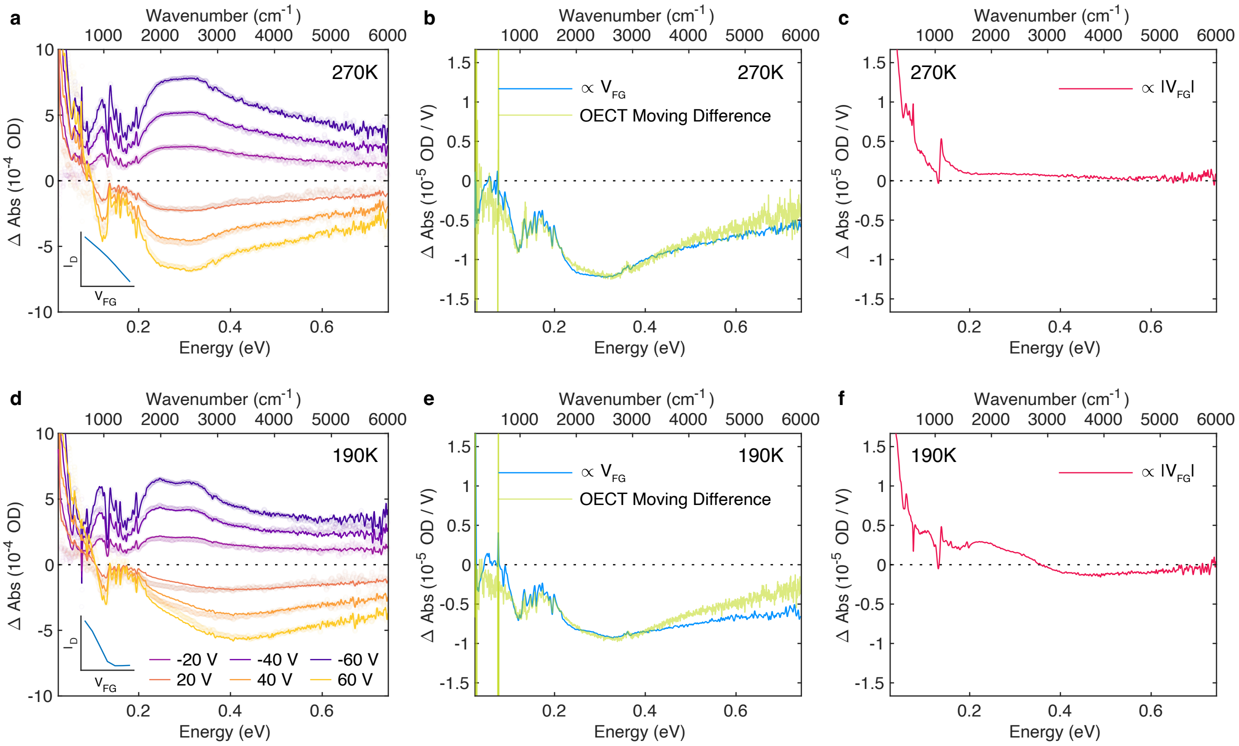

Supplementary Figure 45: **Charge modulation spectroscopy of IDT-BT in Regime I.** (a, d) Raw data (circles) and fit using symmetric and anti-symmetric components (solid lines). I-V curve is shown in the inset. Data in (a) was measured at 270 K (above the ionic glass transition); data in (d) was measured at 190 K (below the ionic glass transition. (b, e) Anti-symmetric component spectrum ($\propto V_{FG}$) obtained from (b) 270 K data and (e) 190 K data, with scaled OECT moving difference spectrum for comparison. (c, f) Symmetric component spectrum ($\propto |V_{FG}|$) obtained from (c) 270 K data and (f) 190 K data. The device was doped using a BMP TFSI / PMA solution (100/1 mM, ACN) for 60 s. Room temperature device conductivity was 2.0 S cm^{-1}.

For each sample, we collect CMS spectra at 6 gate voltages (-60 to 60 V in 20 V increments) at two temperatures —first at 270 K, well above the ionic glass transition, then at 190 or 150 K, below the ionic glass transition. As discussed in the main text, at high temperatures above the ionic glass transition the spectral response is proportional to V_{FG} (Supplementary Figures 45(a), 46(a), and 47(a)). This response is what we might expect: we are modulating the carrier density by only a small amount relative to the doping density, therefore gating only moves E_F very slightly within the DOS. In this small window accessible by field-effect gating, the polaron localization lengths would be expected to be nearly constant, leading to a CMS signal that maintains a constant shape but scales with gate voltage (i.e., with changes in carrier density). We note that this argument mirrors that made above in the discussion of Supplementary Figure 28, where the constant carrier mobility within this window leads to a drain current that is linear with gate voltage.

Below the ionic glass transition (Supplementary Figures 45(d), 46(d), and 47(d)) the spectral response is no longer linear with gate voltage. In particular, in the sample doped to near the conductivity peak (Supplementary Figure 46(d)), at lower energies around 0.2 eV the absorption scales much more closely to $|V_{FG}|$. We again note that the changes in CMS signal vs. temperature closely mirror that of the drain current—above the glass transition, both appear to be linear (i.e., proportional to V_{FG}) while below the glass transition, they become non-linear, taking on a dependence of approximately $|V_{FG}|$ when doped to near half-band filling.

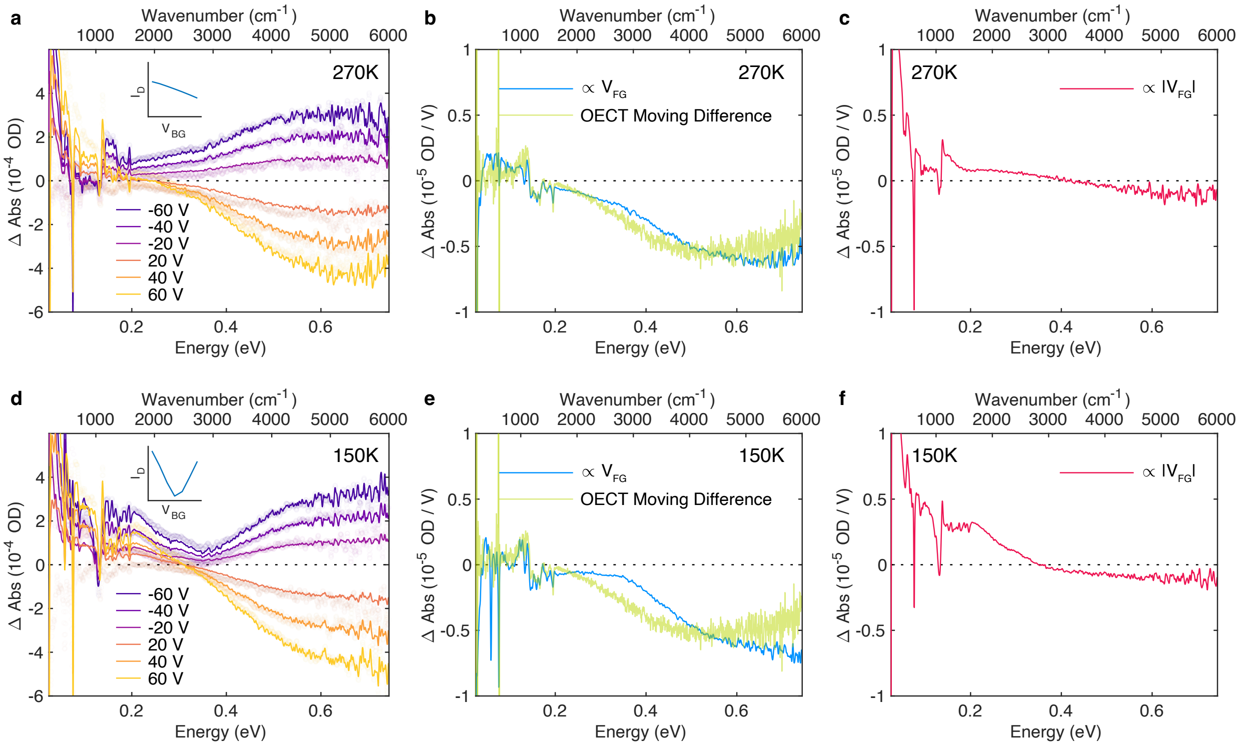

Supplementary Figure 46: **Charge modulation spectroscopy of IDT-BT at the conductivity peak between Regime I and II.** (a, d) Raw data (circles) and fit using symmetric and anti-symmetric components (solid lines). I-V curve is shown in the inset. Data in (a) was measured at 270 K (above the ionic glass transition); data in (d) was measured at 150 K (below the ionic glass transition). (b, e) Anti-symmetric component spectrum ($\propto V_{\mathrm{FG}}$) obtained from (b) 270 K data and (e) 150 K data, with scaled OECT moving difference spectrum for comparison. (c, f) Symmetric component spectrum ($\propto |V_{\mathrm{FG}}|$) obtained from (c) 270 K data and (f) 150 K data. The device was doped using a BMP TFSI / $FeCl_3$ solution (100/1 mM, ACN) for 30 s. Room temperature device conductivity was 9.9 S cm^{-1}.

Following this observation, we decompose the spectra into anti-symmetric and symmetric components, i.e. $A(E) = c_a(E)V_{\mathrm{FG}} + c_s(E)|V_{\mathrm{FG}}|$, as discussed in the main text. This decomposition is performed by performing an independent least-squares fit to the equation above at each energy E, yielding two component spectra $c_a(E)$ and $c_s(E)$, representing respectively the absorption components anti-symmetric and symmetric with respect to V_{FG}. Supplementary Figures 45-47 show these component spectra for each dataset; in each Figure, panels (b) and (e) show the anti-symmetric component at high and low temperature, respectively, while panels (c) and (f) show the symmetric component at high and low temperature, respectively. These components can then be used to reconstruct the experimental CMS spectra at each gate voltage. These reconstructions are shown as the solid lines in Supplementary Figures 45-47 panels (a) and (d), while experimental data are shown as open circles. Agreement is quite good, indicating that our analysis accurately captures the gate-induced changes in absorption. We note, however, that our theoretical model predicts an increase in carrier delocalization with increasing $|V_{\mathrm{FG}}|$, which would imply that higher order terms (i.e. $|V_{\mathrm{FG}}|^n$) should be nonzero. We have limited our analysis here to only first-order contributions due to the risk of overfitting.

As illustrated above in Supplementary Figure 28, and the inset in panel (a) of Supplementary Figures 45-47, at high temperature the field-effect gate transfer curve is linear, reflecting the fact that the carrier mobility is constant over the small range of carrier densities accessible by field-effect gating. As a consequence, we might expect that the localization length, or equivalently the shape of the CMS spectra, should also remain constant for all V_{FG}. This is indeed what we observe—the anti-symmetric component spectra (panel (b), Supplementary Figures 45-47) dominate the overall CMS spectra, while the symmetric component only shows a tail at low energy due to the Si substrate (discussed in more detail below). These anti-symmetric component spectra appear negative because they show the absorbance per volt, and for p-type materials a negative gate voltage results in a positive absorbance.

For comparison, we calculated moving difference spectra from our *operando* ion gated FTIR spectra shown in Extended Data Figure 4.[1] These spectra, shown for each regime in Supplementary Figure 48, represent

[1]Mathematically, these moving difference spectra are a convolution of the ion gating spectra with a kernel [1 0 ... 0 -1] along the scan number dimension, where the total number of elements equals the window size (9 for spectra shown in Figure 48).

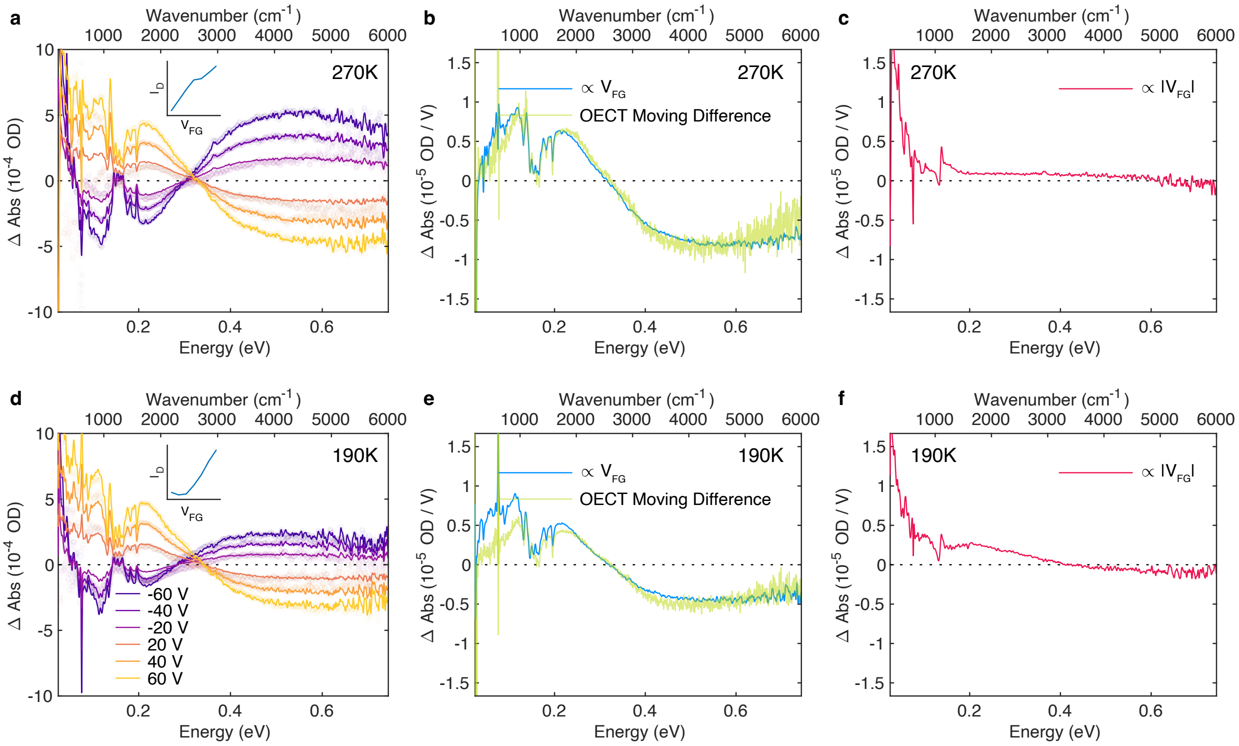

Supplementary Figure 47: **Charge modulation spectroscopy of IDT-BT in Regime II.** (a, d) Raw data (circles) and fit using symmetric and anti-symmetric components (solid lines). I-V curve is shown in the inset. Data in (a) was measured at 270 K (above the ionic glass transition); data in (d) was measured at 190 K (below the ionic glass transition. (b, e) Anti-symmetric component spectrum ($\propto V_{\mathrm{FG}}$) obtained from (b) 270 K data and (e) 190 K data, with scaled OECT moving difference spectrum for comparison. (c, f) Symmetric component spectrum ($\propto |V_{\mathrm{FG}}|$) obtained from (c) 270 K data and (f) 190 K data. The device was doped using a BMP TFSI / $FeCl_3$ solution (100/1 mM, ACN) for 60 s. Room temperature device conductivity was 6.0 S cm^{-1}.

the differential change in absorption over a small change in carrier density, as in CMS spectra, however in this case the carrier density is varied by ion gating (i.e. a change in doping level) rather than field effect.

As shown in the blue line in panel (b) of Supplementary Figures 45-47, these ion gated moving difference spectra closely match the anti-symmetric component spectra when suitably scaled, including a negative sign as indicated above. This similarity indicates that at these high doping levels, the localization length (and therefore presumably the charge carrier mobility) of charges induced by field effect and by doping are identical. This is consistent with our previous work (ref. [6]) in which we found that at high doping level, conductivity is independent of ion size for most polymers because carriers are delocalized over a distance that is large relative to the ion-ion spacing. In this regime, the carriers are unable to feel the local variation in potential due to ions, and therefore see the ionic charge as a nearly uniform background charge density, as in the depletion region of an FET. This point is rather important, as it indicates that the enhancement in thermoelectric properties achievable by field effect should be equally possible by doping, potentially enabling a bulk version of the interfacial enhancement discussed below in Supplementary Note 10.

The symmetric component spectra contain the evidence for enhanced delocalization upon gating below the ionic glass transition, however they also contain two notable artifacts which do not affect our interpretation, but we will discuss first for completeness. In the high temperature symmetric component ($\propto |V_{\mathrm{FG}}|$, panel (c) in Supplementary Figures 45-47, all three samples plotted together for comparison in Supplementary Figure 49(a)) we observe a sharp, dispersive-like feature at 0.13 eV that is nearly identical in all three samples, and can easily be identified as the Stark shift of the Si-O vibrational mode within the SiO_x dielectric layer. This signature also appears in the low temperature data, and thus cancels nicely in the difference spectrum of the low - high temperature symmetric component (Figure 4(c) main text). More significantly, we also observe a strongly increasing absorption at low frequency, which again is nearly identical for all three samples (Supplementary Figure 49(a)). We assign this feature to the absorption of gating-induced charges in the undoped Si substrate—in essence the CMS spectrum of the Si gate. This feature appears in the symmetric component because the carrier density in our Si substrates ($\lesssim 10^{12}$ cm^{-3} estimated from resistivity) is smaller than the carrier density induced by field-effect ($3-4\times10^{12}$ cm^{-2} at 60 V). As a result, both positive and

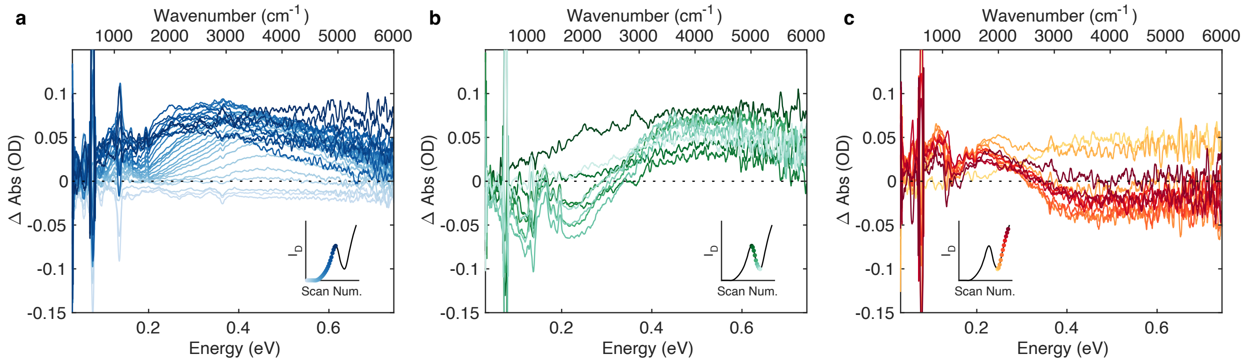

Supplementary Figure 48: **IDT-BT ion gel gated moving difference spectra.** (a-c) Moving difference spectra for (a) Regime I, (b) II, and (c) III calculated from data shown in Extented Data Figure 4, using a sliding window size of 9 scans; only every 4th scan is plotted for clarity. Color code is shown on IV-curve, inset.

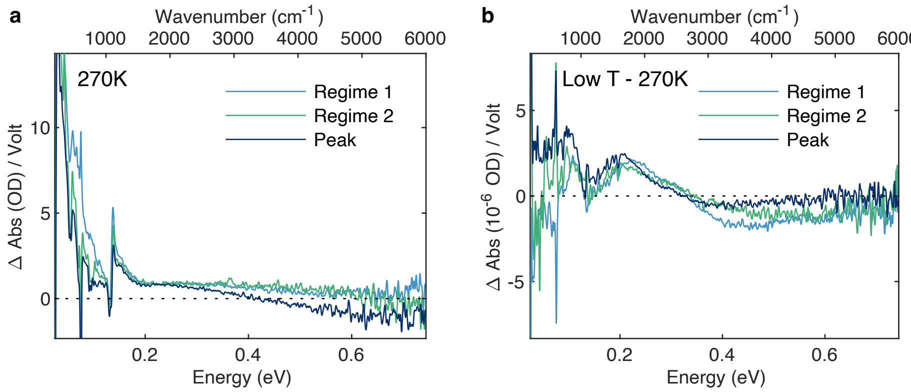

Supplementary Figure 49: **Comparison of IDT-BT CMS symmetric component spectra for all datasets.** (a) 270 K symmetric component spectra. (b) Low temperature symmetric component (190 or 150 K) minus 270 K symmetric component.

negative gating results in an increase in the Si substrate conductivity, resulting in a Drude tail that increases in magnitude with $|V_{\mathrm{FG}}|$. This artifact primarily affects data below 0.1 eV, which is largely below even the low energy polaron A peak,[17] and therefore does not pose a major concern for our measurements. At low temperature (panel (f) in Supplementary Figures 45-47), this tail appears quite similar as at high temperature because the conductivity of Si varies quite weakly in this range.[54, 55]

We can obtain a clear signature of the change in the symmetric component spectrum due to the ionic glass transition by taking the difference between the low temperature and high temperature symmetric component spectra. This is plotted in Figure 4(c) of the main text for the Regime I sample, and in Supplementary Figure 49(b) for all three samples together. We observe a remarkable similarity in these spectra, which all show an enhanced absoprtion at low energy and a bleach at high energy, indicative of a spectral shift in the polaron band to lower energy. Notably, the shape of these spectra are remarkably similar to those observed in the ion gel gated moving difference spectra in Regime III (Supplementary Figure 48(c)) and to the negative of those in Regime II (Supplementary Figure 48(b)). These similarities stem from the fact that above 1 ion per monomer, further doping must necessarily convert singly charged states into multiply charged spectra, leading to the appearance of isosbestic points in the IR spectra (Extended Data Figure 4). In these regimes, therefore, the concentration of absorbing species remains constant, but the localization of these species changes—carriers become increasingly localized upon doping in Regime II, while in Regime III they become increasingly delocalized. This is identical to what we see in the symmetric component spectra, which by nature can only reflect changes in localization, rather than carrier density. Below the ionic glass transition temperature, addition or removal of charges both show the same red-shifted spectra, indicating an increase in carrier delocalization. This enhanced delocalization with gating occurs even though the change in carrier density possible by field effect is quite small, indicating the existence of a narrow dip in the DOS that becomes frozen at low temperature. In this sense, our CMS data provide direct spectroscopic evidence for a frozen soft gap in the DOS at E_F.

Supplementary Note 10 Double-gated Seebeck measurements

10.1 Theoretical framework

We first describe our hypothesis on how the gap formation evident from the low-temperature transfer-curves would affect the Seebeck coefficient. Within Fritzsche's framework of thermoelectric transport coefficients,[56, 57] solving the Boltzmann transport equation gives the expression for electrical conductivity σ:

$$\sigma = \int \sigma_E \left(-\frac{\partial f}{\partial E}\right) \mathrm{d}E, \tag{22}$$

where $\partial f/\partial E$ is the energy-derivative of the Fermi-Dirac distribution and σ_E is the energy-dependent transport function. Within the relaxation time approximation σ_E is depends on the relaxation time τ_s and the carrier velocity v, and is proportional to the density-of-states $g(E)$. By extension of the same framework, the Seebeck coefficient S can be written as:

$$S = \frac{k_B}{e} \frac{\int \sigma_E \left(-\frac{\partial f}{\partial E}\right) \left(\frac{E-E_F}{kT}\right) \mathrm{d}E}{\int \sigma_E \left(-\frac{\partial f}{\partial E}\right) \mathrm{d}E}. \tag{23}$$

For a metallic system where transport happens at states near the Fermi level E_F, Supplementary Equation 23 can be simplified by taking the first non-vanishing term of the Taylor series expansion around $E = E_F$, yielding:

$$S = -\frac{\pi^2}{3} \frac{k_B}{E} k_B T \left[\frac{\mathrm{d} \ln \sigma}{\mathrm{d} \ln E}\right]_{E=E_F}. \tag{24}$$

In this metallic case, it follows from the logarithmic derivative term that both the sign and the magnitude of the Seebeck coefficient depend on the asymmetry of the current contributions below and above E_F. While the relaxation time approximation is unlikely to be the most appropriate model of the transport physics of polymeric conductors, we note that the $\sigma_E \propto g(E)$ proportionality is expected to remain valid. The sign and the magnitude of the Seebeck coefficient, to a first approximation, can therefore be interpreted as being mostly dictated by the slope of the density-of-states.

We now discuss how a frozen Coulomb gap is expected to influence the Seebeck coefficient. To illustrate this we have constructed a simplistic density-of-states model in Supplementary Figure 50 to understand qualitatively the changes of the Seebeck coefficient in the vicinity of a frozen Coulomb gap. We discuss two cases of density-of-states obtained by superimposing a background DOS with a linear slope (panels (a) and (e)) and a gap feature (panels (b) and (f)). In Supplementary Figure 50(a-d), (left panels, dark green line), the background DOS decreases with increasing energy, while in (e-h) (right panels, maroon line) the background DOS is increasing with increasing energy. The case depicted in the left panels correspond to the top of the valence band or HOMO, where $g_0(E)$ increases as we go to more negative energies (i.e. deeper into the band). This corresponds to transport in Regime I, where the doping level is below half-band filling, or equivalently in Regime III for the HOMO-1 counterpart. The panels on the right shows the case corresponding to Regime II, where the $g_0(E)$ decreases towards more negative energies beyond half-band filling.

On the second row, in Supplementary Figures 50(b) and (f) we show the Coulomb gap that will be superimposed on $g_0(E)$ at the Fermi level. This gap is modelled as a Lorentzian:

$$C(E) = \frac{A}{\pi} \left(\frac{\Gamma/2}{(E - E_F)^2 + (\Gamma/2)^2}\right). \tag{25}$$

Here, A is a scaling factor that dictates the depth of the Lorentzian gap, Γ is the full width at half maximum (FWHM, here chosen to be 10 meV), and E_F the Fermi level. The gaps in (b) and (f) are modelled to be completely identical. Supplementary Figures 50(c) and (g) show the total density-of-states including the contribution from the Coulomb gap, $g(E) = C(E) + g_0(E)$.

We recall from Supplementary Equation 24 that the Seebeck coefficient is largely dictated by the slope of the density-of-states. In our case, it is the slope of the total density-of-states, i.e. $\frac{\mathrm{d}}{\mathrm{d}E}(g(E)) = \frac{\mathrm{d}}{\mathrm{d}E}(C(E) + g_0(E)) = \frac{\mathrm{d}}{\mathrm{d}E}(C(E)) + \frac{\mathrm{d}}{\mathrm{d}E}(g_0(E))$ that is of relevance. We show the derivatives for both cases on the third row panels, in Supplementary Figures 50(c) and (g). We note the following:

- If the Fermi level remains tied exactly to the centre of the Coulomb gap, which is expected to be the case in experiments performed under equilibrium, i.e., without field-effect gating, $\frac{\mathrm{d}}{\mathrm{d}E}(C(E))$ is zero: the Coulomb gap has no effect. As shown in the detail plot around the Fermi level in Supplementary Figure 51(b), the slope at $E - E_F = 0$ comes entirely from $g_0(E)$, because the center of $C(E)$, the slope is zero. In our simplistic model, under equilibrium conditions the Seebeck cofficient would therefore be determined by the slope of $g_0(E)$, which is positive in Regime I and III and negative in Regime II.

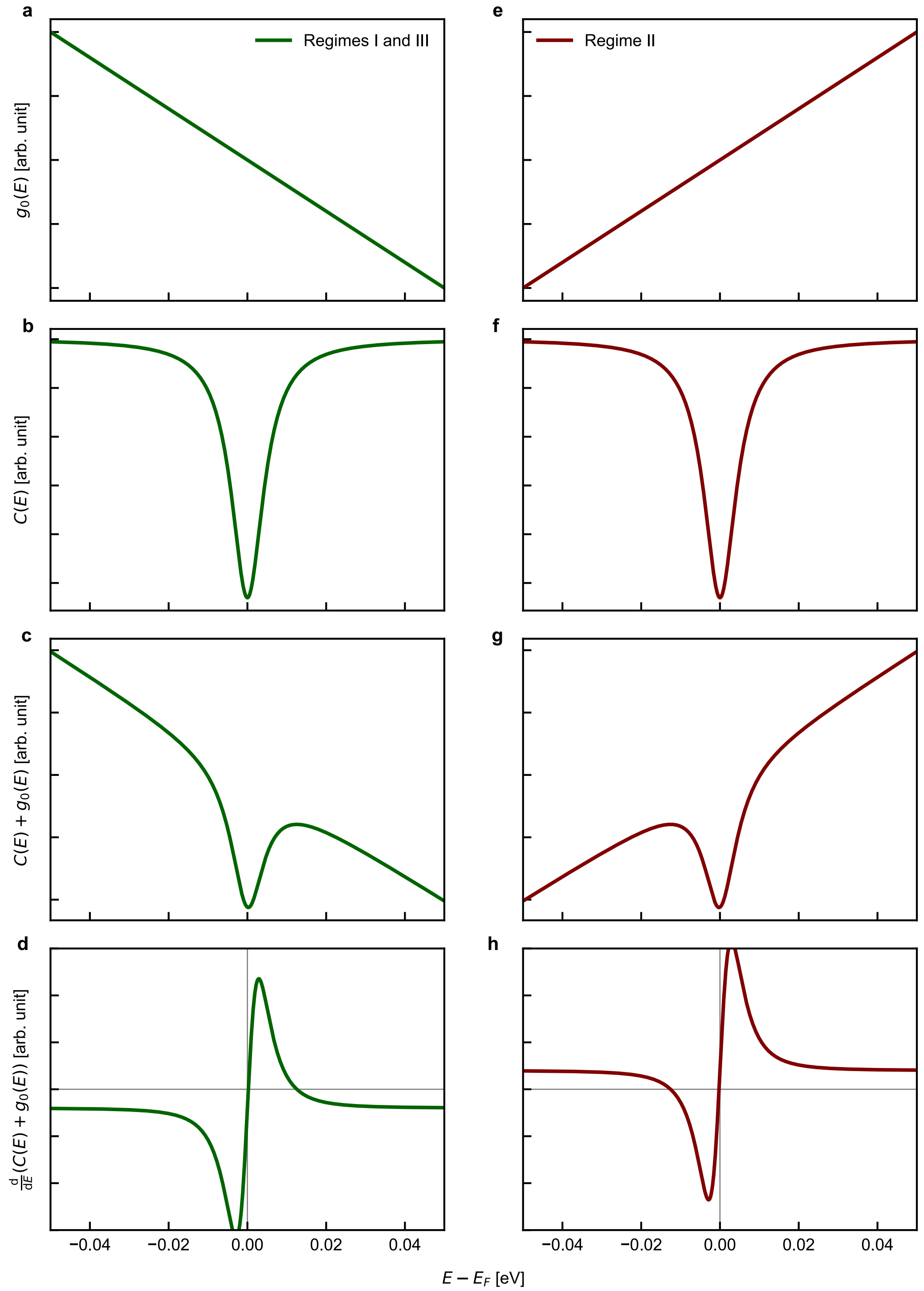

Supplementary Figure 50: **Density-of-states toy models demonstrating the effect of a Coulomb gap on thermoelectric properties.** (a, e) Background DOS $g_0(E)$, (b, f) Coulomb gap feature $C(E)$, (c, g) total DOS $g(E) = C(E) + g_0(E)$, (d, h) derivative of the total DOS $\frac{\mathrm{d}}{\mathrm{d}E}(g(E))$, which is proportional to the Seebeck coefficient.

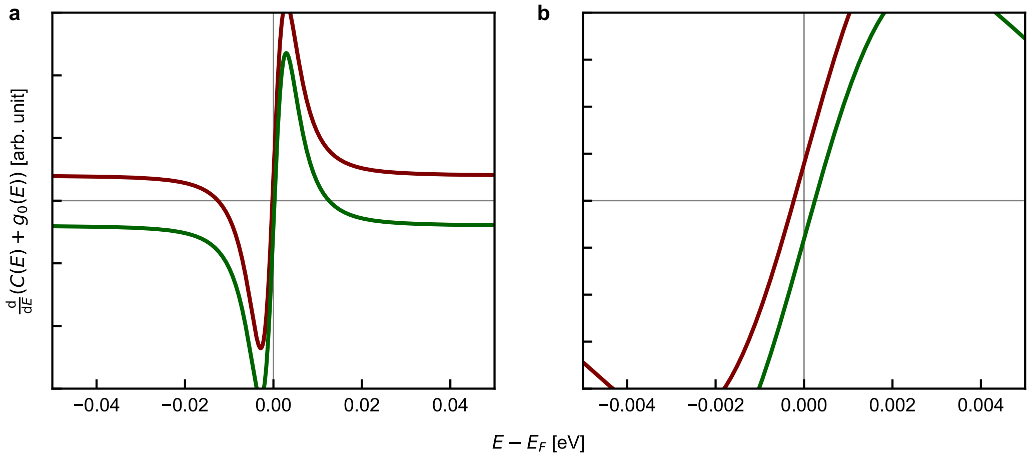

Supplementary Figure 51: **Detailed view of the slope of the total density-of-states $g(E)$ around the Fermi level.** Colour code is consistent with Supplementary Figure 50, where dark green curve corresponds to Regime I and III (left panels of Supplementary Figure 50) and maroon curve to Regime II (right panels of Supplementary Figure 50).

- If, however, the Fermi level is moved away from the centre of the gap into the rapidly changing part of the Coulomb gap contribution to the density of states, (i.e. the Coulomb gap remains frozen upon changes in carrier concentration) the Seebeck coefficient can be enhanced or reduced significantly by the contribution from $\frac{\mathrm{d}}{\mathrm{d}E}(C(E))$.

  In Regimes I and III (green line), the slope can be made more negative, and consequently the Seebeck coefficient more positive, by shifting the Fermi level to deeper energies (Supplementary Figure 51(b)). Similarly for Regime II (maroon), the slope can be made more positive, and the thermopower more negative, by shifting the Fermi level to shallower (more positive) energies.

  Conversely, shifting the Fermi level to less negative energies in Regimes I and III initially leads to $\frac{\mathrm{d}}{\mathrm{d}E}(g(E))$ becoming less negative, reducing the thermopower. Pushing the Fermi level even further, the slope eventually changes sign and the thermoelectric response switches to n-type, as long as the slope of $g_0(E)$ is not too large. An equivalent statement holds true for Regime II. If it is possible to push the Fermi level even further out of the Coulomb gap, the Seebeck coefficient again becomes dominated by the slope of $g_0(E)$, reverting to the sign it has at center of the gap.

We stress that this toy model is not meant to be a realistic representation of the density of states of a real polymer system. It is merely meant to illustrate qualitatively the behaviour of non-equilibrium states as measured in our double-gated Seebeck experiment.

10.2 Experimental challenges

Double-gated Seebeck measurements pose considerable experimental challenges as they compound the challenges of measuring the small Seebeck coefficient in highly doped films and detecting the field-effect modulation of the Seebeck coefficient:

- The Seebeck coefficient of highly doped polymer films is typically less than 100 μV K^{-1}, as seen in Figure 1 of the main article, and Extended Data Figure 7. Measuring such a small voltage generated in a highly conducting film typically requires the use of a high-resolution nanovoltmeter, which allows reliable signal detection even at a level of hundreds of nV. From the perspective of the device-under-test, the device conductance should be as high as possible, to make sure that the signal-to-noise ratio is maximized.

- In field-effect gated Seebeck measurements of undoped polymers, where the thermopower is measured as a function of field-effect modulated carrier density, the main measurement challenge is the large device resistance and the fact that a large gate voltage is needed to modulate the Seebeck coefficient.

  The problem with device resistance is solved by using a high input-impedance measurement unit - we use a Keithley 6430 Sub-femtoamp Source Meter Unit with input impedance of $> 10^{15}$ Ω (Supplementary Table 1). The large gate voltage poses a more difficult measurement problem, because any leakage

current through the dielectric needs to be minimized as not to interfere with the signal of interest. As will be detailed in Supplementary Note 10.2.1, the presence of a leakage current through the polymer channel could potentially be picked up as an additional potential drop that could create an artifact in the measured thermovoltage. We refer to this error as *thermovoltage error due to gate leakage.* Previous experiments have highlighted the importance of ensuring very low gate currents of less than 100 pA. We argue in Supplementary Figure 53 that in addition to monitoring the gate leakage current, a ground-polarity check also helps to verify the absence of any potential measurement artefacts.

  In a typical undoped FET we expect sizeable changes in the thermopower with field-effect modulation — from Heikes formula we expect this to be 198 μV K^{-1} for every decade of carrier density modulation. This relatively large change in signal is rather tolerant to the thermovoltage error due to gate leakage. As an illustration, an error of 1 μV does not pose a significant problem when the change in the signal that we are looking for is on the order of 100 μV, but it will become an issue for a signal on the order of 10 μV signal. A large signal also allows us to tolerate the lower resolution of the high input-impedance measurement unit compared to a nanovoltmeter, which is 1 μV for the case of Keithley K6430.

- For both measurements described above, a large potential on the order of tens of V is applied to the on-chip heater, to induce Joule heating and a temperature difference ΔT across the channel. If there is a leakage pathway between the heater and the polymer channel, we can also have *thermovoltage error due to heater leakage*. This will be discussed in Supplementary Note 10.2.2.

The double gated Seebeck measurements effectively combines the more challenging aspects of both measurements. The Seebeck coefficients in this doping regime are inherently small due to the high carrier densities, yet we are looking for small changes on top of these already small signals by applying large gate voltages. The problem is further complicated as we have opted to use field-effect gated device architecture on Si/SiO_2 substrates in order to be able to leave the top polymer surface accessible to the ion gel. As detailed in Supplementary Figure 3, the high bulk thermal conductivity of silicon compared to the interfacial thermal conductance results in small temperature differences ΔT between the hot and cold sides of the device. As the thermovoltage is proportional to ΔT, and the Seebeck coefficient S is inherently small, the net thermovoltage signal that is measured will inevitably be small. This places stringent constraints on voltage artifacts due to leakage currents (e.g. gate or heater leakage), which under some circumstances can be comparable in magnitude to the thermal voltage signal.

10.2.1 Thermovoltage error due to gate leakage

We now present a more formal analysis of how the gate and heater leakage could influence the measured signal in our double-gated Seebeck experiments. We concentrate first on the issue of gate leakage. Supplementary Figure 52 shows a schematic of the components of our double-gated Seebeck device that are relevant to this analysis. On the schematic, we have superimposed an equivalent circuit representation that we will use to account for the potential measurement artifacts. The polymer channel between the hot and cold temperature sensors is modelled as a resistance $R_{\text{tot}} = R_{\text{S1}} + R_{\text{S2}}$. As noted in the schematic, there is a temperature gradient along the channel, such that the left side of the device is hotter than the right side. A voltmeter measures the potential of the cold side relative to the grounded hot side ($\frac{\perp}{=}$). At the polymer/dielectric interface, the carrier density is field-effect modulated by a potential V_{FG} applied to the silicon field-effect gate. We take into account the dielectric imperfections by assuming that a large but finite resistance R_{L} through the dielectric layer at some point in the device, such that the application of V_{FG} will be inevitably accompanied by the passage of some leakage current I_{L}.

We note that this equivalent circuit representation has been deliberately simplified to only contain the terms that should be most relevant in the context of the experiment. For instance, we have deliberately excluded the time-dependence of the capacitance of the dielectric, since these are dc experiments with time scales chosen such that the dielectric is always fully charged long before the start of the signal acquisition. We have also omitted the resistance of the silicon field-effect gate, which is always small compared to the relevant values of R_{L}.

Consider first the ideal case, where the dielectric is perfectly insulating and $R_{\text{L}} = \infty$. In this case, there is no current flowing through the dielectric $I_{\text{L}} = 0$ in response to the application of the field-effect gate potential. The dielectric behaves as a capacitor to store the energy associated with V_{FG}. When a voltmeter is connected between the hot and cold side sensors of the device we then pick up purely a thermoelectric signal, with no leakage-associated artifacts. Importantly, when changing the measurement polarity, i.e. from cold-ground to hot-ground or vice versa, we should only observe a sign change of the voltage, while the magnitude remains constant.

For the case where R_{L} is large but finite, a leakage current will flow through the dielectric to the polymer channel and finally into the ground. The leakage current I_{L} can be calculated by noticing that the total

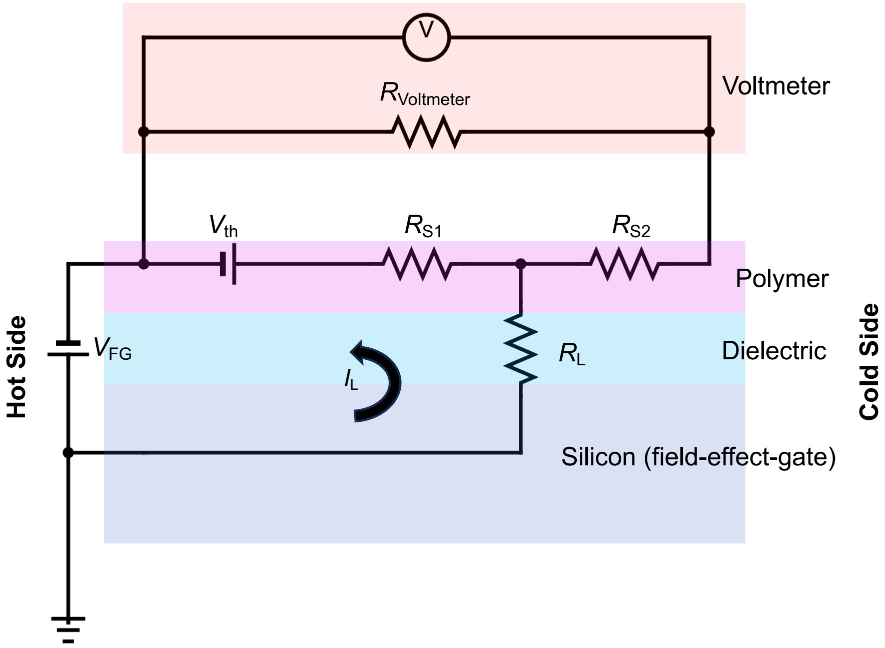

Supplementary Figure 52: **Equivalent circuit model to account for field-effect leakage in a double-gated Seebeck measurement.** The polymer channel is modeled by a resistance of $R_{\text{tot}} = R_{\text{S1}} + R_{\text{S2}}$ and a p-type Seebeck response given by V_{th}. The application of a field-effect gate voltage V_{FG} on a dielectric with finite resistance leads to the flow of leakage current through a pathway with resistance R_{L}. The device is connected to a voltmeter with input impedance $R_{\text{voltmeter}}$ to facilitate the measurement of thermovoltage.

resistance of this leakage pathway comprises of the resistance of the leakage pathway through the dielectric R_{L}, as well as resistance of the two parallel pathways through the polymer:

$$I_{\text{L}} = \frac{V_{\text{FG}}}{R_{\text{L}} + [R_{\text{S1}}^{-1} + (R_{\text{S2}} + R_{\text{Voltmeter}})^{-1}]^{-1}}. \tag{26}$$

Because the Seebeck coefficient corresponds to the open-circuit voltage generated by a temperature gradient, $R_{\text{Voltmeter}}$ must always be large relative to $R_{\text{tot}} = R_{\text{S1}} + R_{\text{S2}}$. Therefore, the above expression simplifies to:

$$I_{\text{L}} = \frac{V_{\text{FG}}}{R_{\text{L}} + R_{\text{S1}}}. \tag{27}$$

The magnitude of R_{S1} depends on the sample conductivity and the location of the leak in the dielectric layer, and therefore can range in value between 0 and R_{tot}.

Having quantified the magnitude of the leakage current, we now discuss how this could be picked up as a voltage artifact. Since the polymer channel that is resistive, by Ohm's law the passage of a leakage current I_{L} through the relevant portion of the polymer channel (with resistance R_{S1}) corresponds to a potential difference of V_{S1}:

$$V_{\text{S1}} = I_{\text{L}} \times R_{\text{S1}} = \left(\frac{V_{\text{FG}}}{R_{\text{L}} + R_{\text{S1}}}\right) R_{\text{S1}} = \left(\frac{R_{\text{S1}}}{R_{\text{L}} + R_{\text{S1}}}\right) V_{\text{FG}}. \tag{28}$$

V_{S1} will add or subtract with the thermal voltage, leading to a measurement artifact that we refer to as *thermovoltage error due to gate leakage.* From Supplementary Equation 28 we note:

- V_{S1} is expected to vary monotonically with V_{FG}. Consequently, both the polarity and magnitude of V_{S1} will be proportional to V_{FG}.
- V_{S1} should ideally be less than a few hundreds of nanovolts, or at most 1 μV, as the thermal voltage signal is only about an order of magnitude larger. We first note that since $R_{\text{L}} >> R_{\text{S1}}$ for doped polymers in general, we can approximate Supplementary Equation 28 to $V_{\text{S1}} \sim (R_{\text{S1}}/R_{\text{L}})V_{\text{FG}}$. Assuming that the dielectric performs equally well on average (R_{L} constant), V_{S1} scales linearly with the resistance of the polymer channel R_{S1}.

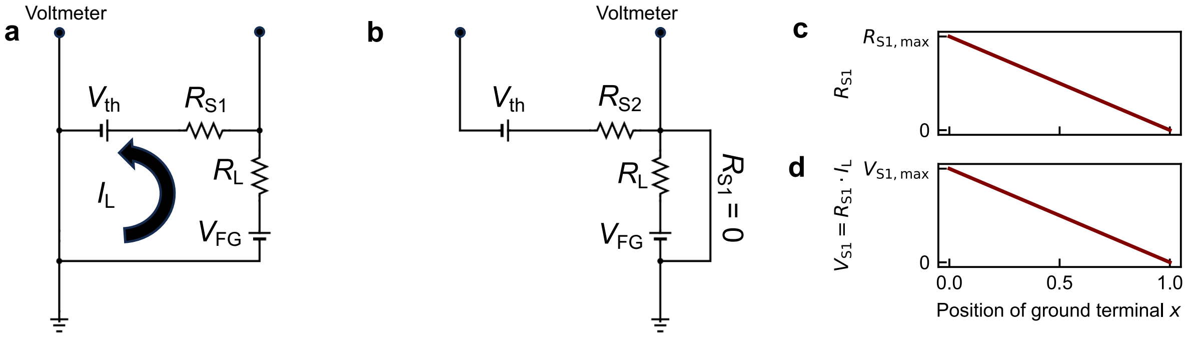

Supplementary Figure 53: **Dependence of the *thermovoltage error due to gate leakage* V_{S1} on the position of the leakage pathway.** Equivalent circuit model for the cases where the defect is located exactly on the (a) hot side and (b) cold side of the device. Generalized variations of (a) the resistance of the polymer channel that is passed by the leakage current R_{S1} and (b) the corresponding thermovoltage error V_{S1} as a function of the position of the ground terminal x.

Typically, we have a leakage of 50 pA at $V_{FG} = 50$ V, therefore $R_L \sim 10^{12}$ Ω. The device resistance of our most conducting PBTTT films is on the order of 10 kΩ, such that $V_{S1} \sim 10$ nV. Gate leakage and V_{S1} is therefore not expected to be a significant problem in our PBTTT measurements, since the estimated V_{S1} is significantly lower than the signals that are being measured.

On the other hand, the device resistance of our most conducting IDT-BT devices in Regime III is still at least on the order of 1 MΩ. This means that $V_{S1} \sim 1$ μV, which now approaches the expected magnitude of the signal. Under this condition, the experiment is still possible, however utmost care must be exercised. We emphasize that as detailed in Supplementary Note 2, we had intentionally designed the device architecture to minimize V_{S1}. Within the practical limitations described in Supplementary Note 2, the architecture of Device E (Supplementary Figure 1) offers the best compromise of all considerations, allowing V_{S1} to be reduced to ~ 1 μV.

We propose two experimental checks that are needed to identify the presence of artefacts in a thermovoltage signal. First, when changing the sign of V_{FG} at zero heater power ($\Delta T = 0$), the voltage measured between the voltmeter terminals will change by $2V_{S1}$ if there is a significant gate leakage contribution. This comes with an advantage that the heater voltage is also zero, ruling out the possibility of any leakage from the heater. In the absence of any leakage, therefore, the nanovoltmeter reading should not change with field-effect gate voltage in steady state.

The second check can be done by switching the position of the ground terminal, e.g. from grounding the cold side to hot side, or vice versa. In the absence of gate leakage, the thermovoltage should be completely symmetrical; changing the position of the ground terminal should not change the voltage profile that is developed by Seebeck effect. On the other hand, the value and polarity of V_{S1} do generally change when switching the position of the ground terminal. This is because $V_{S1} \propto R_{S1}$, and therefore the position of the leakage pathways with respect to the ground terminal will very much influence V_{S1}: the further away the leak is from the ground terminal, the larger is V_{S1}.

To illustrate the argument, we consider an exaggerated case where there is only one defect in the dielectric, and it is exactly on the cold-side temperature sensor, as depicted in Supplementary Figure 53. In this case, $R_{S1} = R_{tot}$, while $R_{S2} = 0$. On panel (a), the ground terminal is located on the hot-side thermometer, i.e. on the opposite end of the device from the leakage pathway. In this case, leakage current will flow through the defect R_L, then through the entire polymer (R_{S1}) before reaching the ground terminal. Consequently, V_{S1} is also maximized at $V_{S1} = R_{S1} \times I_L$.

Panel (b) illustrates the other extremum, where the leak is directly to the the cold-side temperature sensor, which is grounded. In this case, the leakage does not pass through any part of the polymer channel, i.e. $R_{S1} = 0$ and thus $I_L = V_{FG}/R_L$. Although this condition maxmizes the leakage current I_L, by Equation 28 we can also see that the leakage contribution to the measured thermal voltage V_{S1} is zero in this case, because $R_{S1} = 0$.

In panels (c) and (d), we generalize our argument for an arbitrary position of ground terminal x, where $x = 0$ corresponds to the ground being on the hot-side temperature sensor and $x = 1$ on the cold-side. It is evident that depending on the position of the ground terminal, the magnitude of the V_{S1} artifact to the measured signal will be different; in our simple model it varies linearly.

To appreciate better how V_{S1} will manifest itself in our thermovoltage signal, in Supplementary Figure 54 we show expected nanovoltmeter readings for the cases of grounding the hot and cold sides. We assume a p-type material with a true Seebeck coefficient of 20 μV K^{-1}. The total resistance of the channel is

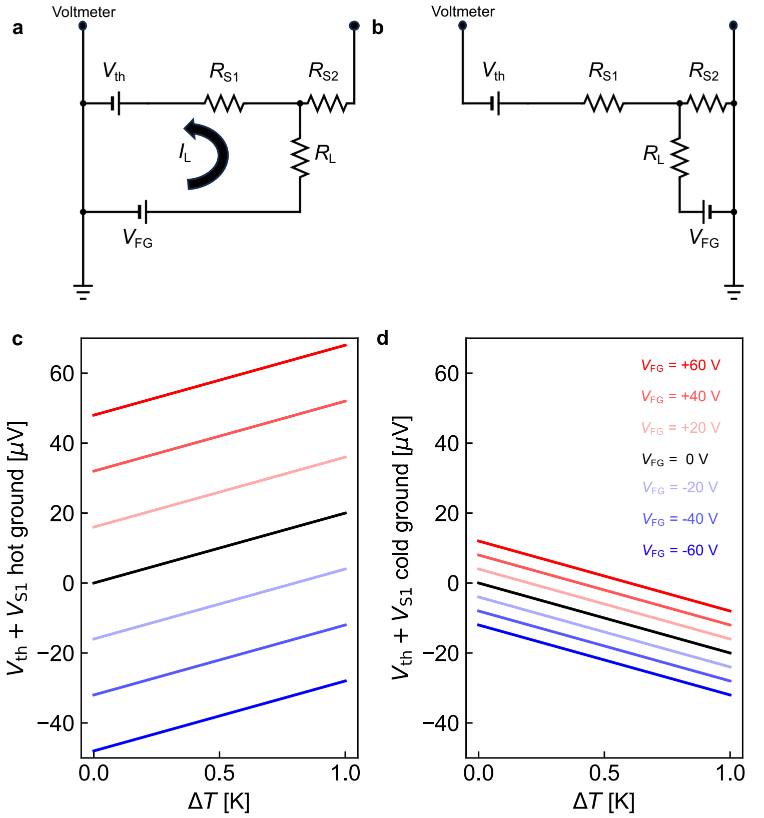

Supplementary Figure 54: **Calculated contributions of the thermovoltage error due to gate leakage V_{S1} on the overall measured signal.** Equivalent circuit model for (a) a hot-grounded measurement and (b) a cold-grounded measurement for the case where the leakage pathway position $x = 4/5$. Calculated (c) hot-grounded and (d) cold-grounded signals with the error contributions at various field-effect gate voltages V_{FG}. Notice that the error associated with positive V_{FG} shifts the signals upwards to more positive values irrespective of the ground polarity, and the opposite is true for negative V_{FG}.

$R_{\text{tot}} = R_{\text{S1}} + R_{\text{S2}} = 10^6\ \Omega$, i.e. $1\ \text{M}\Omega$; the chosen Seebeck coefficient and device resistance match that of our IDT-BT samples in Regime III. We have incorporated a single defect associated with $R_{\text{L}} = 10^{12}\ \Omega$ at $x = 4/5$, i.e. the defect is located 0.8 of the total channel length away from the left (hot) side. The equivalent circuit representation of this diagram is shown in Supplementary Figure 54(a) and (b) for the hot and cold grounded cases respectively.

Panels (c) and (d) summarize the signals that would be measured for the cases of hot and cold grounding respectively, at various field-effect gate voltages V_{FG}. As the material is p-type, under a temperature difference ΔT holes are expected to thermally drift from the hot to the cold end of the channel, resulting in a carrier population imbalance with hole accumulation at the cold end. As the temperature difference ΔT is increased from zero, the nanovoltmeter measures an increasingly positive (negative) signal for the case of hot (cold) grounding, reflecting the build-up of holes (electrons) on the high terminal of the nanovoltmeter. Since the material is assumed to have a Seebeck coefficient of $+20\ \mu\text{V K}^{-1}$, at $\Delta T = 1$ K we expect the high terminal of the nanovoltmeter to be more positive by 20 μV for hot-side-grounding, and more negative by 20 μV for cold-side-grounding — these 20 μV potential differences arise purely from Seebeck effect and are seen for all values of V_{FG}.

We now discuss the changes seen with applied field-effect gate voltage V_{FG}, concentrating first on the case of hot grounding. For positive V_{FG}, we see upwards shifts of the signal measured towards more positive values. Notice that these shifts are independent of ΔT. These shifts correspond to the *thermovoltage error due to gate leakage* $V_{\text{S1}} = R_{\text{S1}} \times I_{\text{L}}$. The polarity of V_{S1} is the same as that of V_{FG}; physically, this can be understood as some of the potential from V_{FG} leaking to the cold-side of the device (for the hot-grounded case). Therefore, for positive V_{FG}, the cold-side of the device is at a more positive potential solely due to the contribution of gate leakage (V_{S1}), which is independent of any Seebeck effect. Conversely, the application of negative V_{FG} leads to some negative potential leaking to the cold-side of the device. V_{S1} now has the opposite polarity to the thermovoltage V_{th}, therefore it causes the measured signal to be more negative. The final feature to note from the hot-grounded signal is the linear increase of V_{S1} with V_{FG}, following Supplementary Equation 28).

We now consider the cold-grounded case shown in Supplementary Figure 54(d). To start, we consider the $V_{\text{FG}} = 0$ V signal, where for $\Delta T = 1$ K the voltmeter measures the hot-side of the device to be 20 μV biased with respect to the cold-side ground. It should be emphasized that this cold-ground signal completely mirrors the hot-ground counterpart discussed before, where the cold-side was measured to be +20 μV biased with respect to the hot-ground. We refer to this as the cold-ground signal being perfectly *symmetrical* to the hot-ground signal.

The application of positive (negative) V_{FG} shifts the hot-side to more positive (negative) potentials, therefore causing shifts in the same direction as for the hot-grounded measurements. It is also evident that the shifts between consecutive gate-voltages, corresponding to V_{S1}, are smaller compared to the hot-grounded counterpart. This follows from the smaller R_{S1}, as illustrated in Supplementary Figure 54(b).

To conclude this Supplementary Note, we summarize the important signatures of the presence of gate leakage artifacts in a thermovoltage measurement:

- The offset of the measured voltage V_{th} at $\Delta T = 0$ shifts towards more positive (negative) values when applying positive (negative) V_{FG}.
- The shifts, corresponding to V_{S1}, are rigid i.e. their magnitudes remain constant for any of ΔT. A gate leakage artifact is not expected to manifest as a slope change in the thermovoltage signal.
- The signals obtained when grounding the hot and cold sides are not in general symmetrical.

10.2.2 Thermovoltage error due to heater leakage

Analogous to the gate leakage, any leakage from the on-chip heater could also result in measurement artifacts. There are two major pathways for heater leakage in these double-gated measurements. The first one, which is also seen for measurements on glass, i.e. without field-effect gating, is leakage as a consequence of incomplete patterning of the polymer film such that the heater is electrically connected to the patterned Hall bar. This contribution can and has been minimized by ensuring that the resistance between the heater and the polymer Hall bar is very high, i.e. beyond the measurement capabilities of a Keithley 2612B Source Measure Unit.

The second leakage pathway is through dielectric defects, similarly to the gate leakage. If defects are present immediately below both the heater and the polymer Hall bar, a leakage current could flow from the heater, via the conducting silicon substrate, into the polymer and subsequently the ground terminal. In an experiment, the two pathways are indistinguishable. We note, however, that the second pathway is likely to be more dominant for measurements on Si/SiO_2 susbtrates, such as the present double-gated Seebeck experiments.

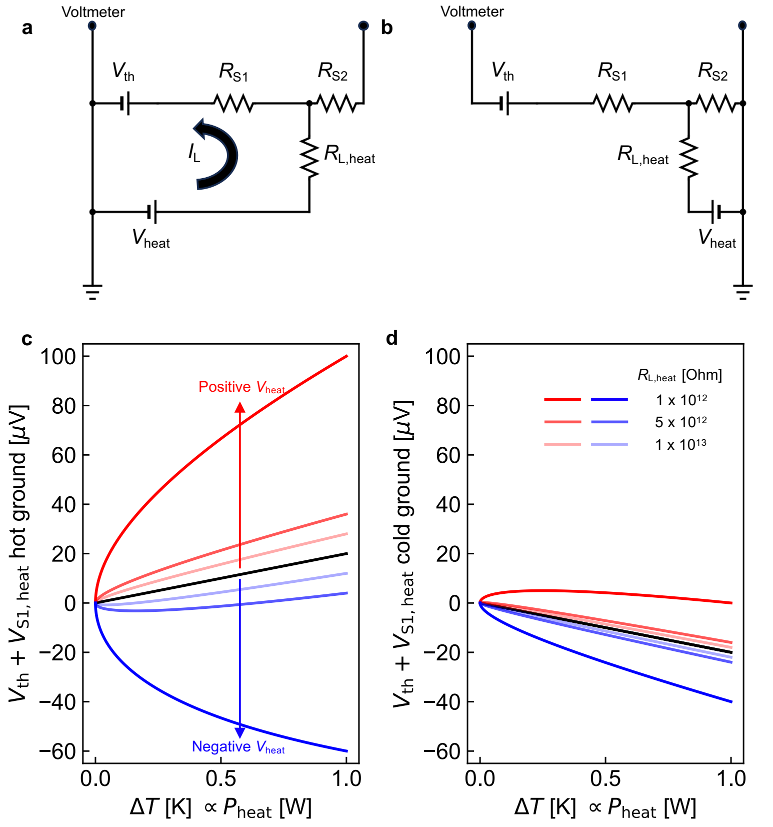

Supplementary Figure 55: **Calculated contributions of the thermovoltage error due to heater leakage $V_{S1,heat}$ on the overall measured signal.** Equivalent circuit model for (a) a hot-grounded measurement and (b) a cold-grounded measurement for the case where the leakage pathway position $x = 4/5$. Calculated (c) hot-grounded and (d) cold-grounded signals with the error contributions at various leakage pathway resistances $R_{L,heat}$. Notice that the error associated with positive heater voltages V_{heat} shifts the signals upwards to more positive values irrespective of the ground polarity, and the opposite is true for negative V_{heat}.

The equivalent circuit diagram for the heater leakage mechanism is shown in Supplementary Figures 55(a) and (b). The polymer film is defined identically to the one for gate leakage analysis (Supplementary Figure 52), with a total resistance $R_{\rm tot}$ comprising $R_{\rm S1}$, the resistance of the polymer between the leak and the left (hot) side of the device, and $R_{\rm S2}$, the resistance of the polymer between the leak and the right (cold) side of the device. We assume again a p-type channel, with the induced thermovoltage represented by $V_{\rm th}$.

The primary distinction of the present model is the leakage considerations. Although represented by a very similar circuit diagram, the leakage current is now driven by a heater voltage $V_{\rm heat}$, which travels through some leakage pathways (either patterning or dielectric defects), associated with a resistance $R_{\rm L,heat}$, into the polymer channel and subsequently, the ground terminal. Assuming a large voltmeter resistance, as in the previous case, it can be shown that:

$$V_{\rm S1,heat} = \left(\frac{R_{\rm S1}}{R_{\rm L,heat} + R_{\rm S1}}\right) V_{\rm heat} \simeq \left(\frac{R_{\rm S1}}{R_{\rm L,heat}}\right) V_{\rm heat}. \tag{29}$$

To better visualize the effects of heater leakage, we calculate the expected nanovoltmeter reading for hot- and cold-grounded measurements in Supplementary Figure 55(c) and (d) respectively. We first recall the basic operation principles of an on-chip heater; this heater is a thin metal strip, that can be Joule heated by application of some heater voltage V_{heat} to ultimately generate a temperature difference ΔT. We assume that the heater strip has a typical resistance $R_{\rm heat}$ of 10 kΩ, and the heater voltage $V_{\rm heat}$ is varied up to 100 V, giving rise to a maximum heater power of $P_{\rm heat} = V_{\rm heat}^2/R_{\rm heat} = 1$ W. At 1 W of heater power, we assume that there is a $\Delta T = 1$ K between the hot and cold sides of the device. We emphasize that all these parameters are well representative of our double-gated measurements. It is the application of this high heater voltage that could potentially lead to *thermovoltage error due to heater leakage* $V_{\rm S1,heat}$.

The channel is modelled using the same parameters as before being a p-type material with a true Seebeck coefficient of 20 μV K^{-1}, and a total resistance $R_{\rm tot} = R_{\rm S1} + R_{\rm S2} = 1$ MΩ. As before, we assume the same leakage pathway position as in Supplementary Figure 54, i.e. at $x = 0.8$, with a resistance of $R_{\rm L,heat}$. In both panels (c) and (d), we show three different values of $R_{\rm L,heat}$, i.e. 1×10^{12} Ω, 5×10^{12} Ω, and 10^{13} Ω, the last value being the same as the $R_{\rm L}$ assumed in the case of gate leakage (Supplementary Figure 54).

We focus on the hot grounded case (panel (c)) to explain the principal differences with gate leakage. First, unlike the error due to gate leakage $V_{\rm S1}$ that is independent of ΔT, the heater leakage error $V_{\rm S1,heat}$ varies with ΔT. To be specific, the temperature difference ΔT varies proportionally to heater power $P_{\rm heat}$, which varies quadratically with heater voltage $V_{\rm heat}$. It follows that $V_{\rm S1,heat} \propto V_{\rm heat} \propto P_{\rm heat}^{1/2}$ varies with the square root of temperature difference ΔT.

The fact that the error associated with heater leakage varies with the square root of ΔT constitutes its most important difference with the gate leakage errors. The heater leakage errors change the slope of the measured signals, and therefore could be misinterpreted as changes in the Seebeck coefficient; this is unlike the gate leakage errors, which do not chnage the slope (due to their independence of ΔT). Another important consequence of the square root dependence is that $V_{\rm S1,heat} = 0$ for $\Delta T = 0$, i.e. the heater leakage does not change the offset of the signal; this contrasts with the gate leakage errors, where $V_{\rm S1}$ shifts of the offset would allow unambiguous identification of leakage artifacts.

A powerful method of checking for heater leakage artifacts is the ground polarity switching routine. From Supplementary Figure 55, it is evident that the heater leakage shifts the signals in the same way as gate leakage — towards more positive bias for positive heater voltages, be it for cold or hot grounding, and vice versa towards more negative for negative heater voltages. This implies that a heater leakage would increase the slope of the signal when grounded on the hot side, and conversely it would decrease the cold-grounded coefficient. Similarly, inverting the sign of the heater voltage has no effect on the temperature gradient and thus the true thermal voltage signal, but will invert the sign of the leakage artifact, $V_{\rm S1,heat}$. Experimentally, checking for such asymmetries in the signal are a very useful way of detecting potential heater and/or gate leakage, which we have implemented in our measurements (Supplementary Figure 56 in the following section).

10.3 Experimental results

We present measurements on PBTTT and IDT-BT to demonstrate the effect of non-equilibrium transport on the Seebeck coefficient. Measurements on IDT-BT were attempted on both the peak between Regimes I and II and the second rise of Regime III, which are the regimes in which the field-effect transfer-curves show the most pronounced ambipolarity. We found that the significantly higher conductivities in Regime III (10 S cm^{-1}) enabled us to achieve better signal-to-noise ratios in the Seebeck coefficient measurements than those measured on the first peak (2 S cm^{-1}). We therefore concentrated on thermopower measurements in Regime III here, where a significantly ambipolar field-effect gate transfer curve indicates the presence of a Coulomb gap. As a reference measurement we also investigated PBTTT at a high doping level where its field-effect transfer-curve is a unipolar p-type response with negligible non-linearity, suggesting the absence

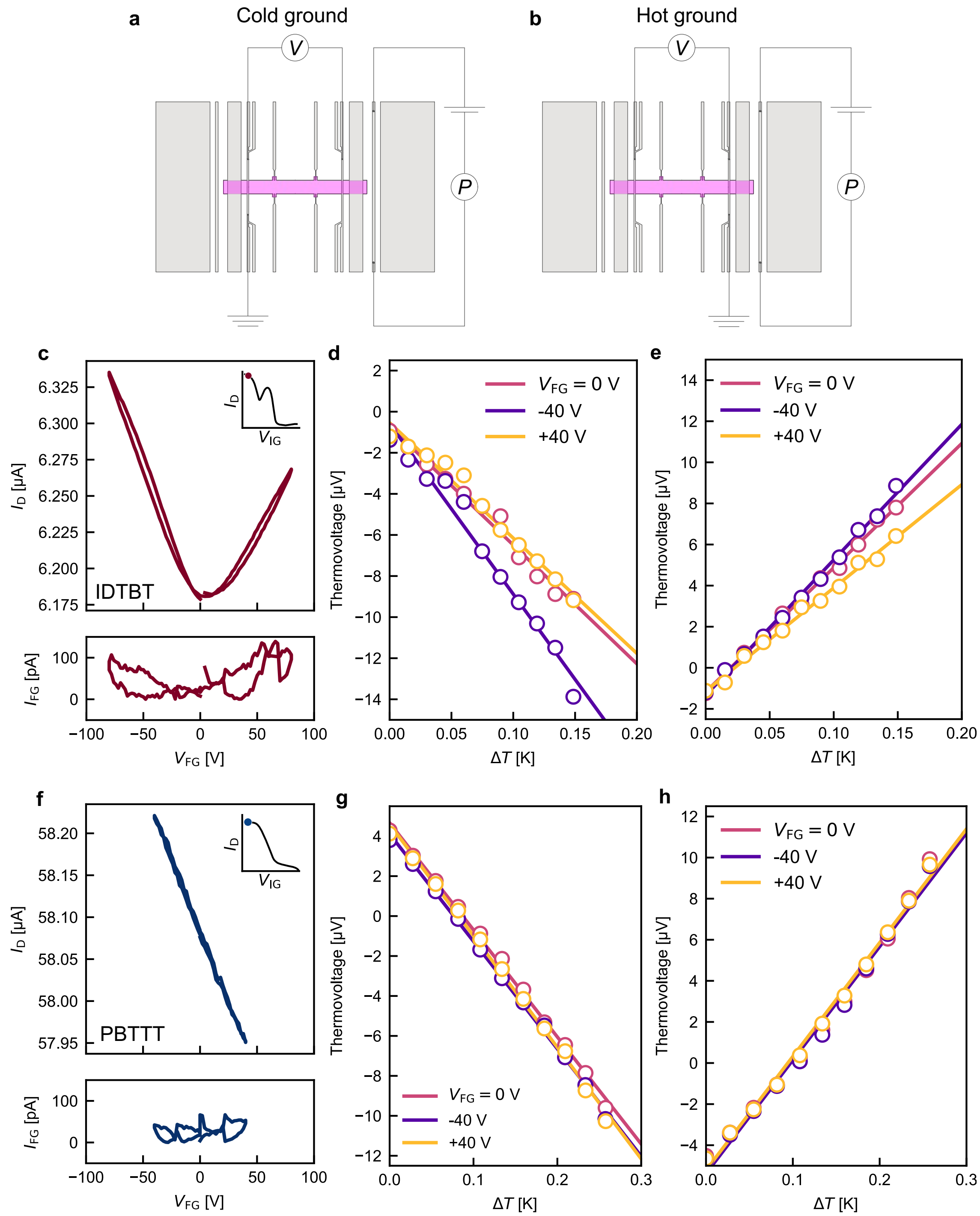

Supplementary Figure 56: **Double gated Seebeck measurements of IDT-BT and PBTTT.** Schematic of the experimental setup, where the thermovoltage is measured while the (a) cold or (b) hot side of the device is electrically grounded. (c) Field-effect gate transfer curve of IDT-BT at the measurement temperature of 190 K. Field effect modulated thermovoltages for experiments where (d) cold side and (e) hot side is grounded. (f) Field-effect gate transfer curve of PBTTT at 190 K, and its field-effect gated thermovoltages with (g) cold and (h) hot ground. Note the negligible effect of field effect modulation in the Seebeck coefficient of the doped PBTTT.

of any energy gap around its Fermi level E_F. The measurement temperature was chosen to be 190 K as a compromise between the following considerations:

- The measurement temperature needs to be high enough for the polymers to show a relatively large Seebeck coefficient. Recall that the Seebeck coefficients of the highly doped IDT-BT in Regime III and the highly doped PBTTT both decrease linearly with temperature obeying the metal-like Mott formula dependence, rendering measurements at very low temperatures impractical due to their vanishingly small coefficients.

- The measurement temperature needs to be low enough for the non-equilibrium transport signatures to be fully developed, i.e. to be below the ionic glass transition temperatures. At high temperatures, the field-effect transfer-curve of the IDT-BT is fully linear, signifying that there is negligible non-equilibrium component to its transport.

The field-effect gated Seebeck measurements of the doped IDT-BT and PBTTT films were performed using on-chip microfabricated devices on Si/SiO_2, where the doping level is adjusted with BMP TFSI ion gel. The PBTTT measurements were performed on Device C, while for IDT-BT we used Device E (see Supplementary Figure 1) in order to allow enough device conductance for the use of the high-resolution Keithley K2182A Nanovoltmeter (input impedance: $\sim$ 10 GΩ, resolution: 1 nV). The overall procedure of the thermovoltage measurements remains similar to our only ion gel gated only devices, discussed in Supplementary Note 1.2. Having adjusted the ion gel doping-state of the device, we apply a constant voltage to the field-effect gate in order to fine-tune the exact position of the Fermi level E_F in the density-of-states $g(E)$. We wait for at least five minutes after the application of a new field-effect gate potential to ensure the gate was fully charged. The thermovoltage is then characterized by sweeping the heater power linearly, allowing one minute of waiting time after the application of a new heater power for thermal stabilization.

10.3.1 PBTTT

We first discuss the Seebeck data for PBTTT, shown in Supplementary Figures 56(g) and (h), in light of the discussion in Supplementary Notes 10.2.1 and 10.2.2. We notice that even in the absence of temperature difference ΔT, a potential difference of +4.1 to +4.7 μV (cold ground) and -5.2 to -5.4 μV (hot ground) was measured. As $\Delta T = 0$, this voltage could not be caused by Seebeck effect, and likewise it could not have a heater leakage origin as there was no voltage applied on the heater i.e. $V_{\text{heat}} = 0$. The fact that the voltage was measured even when $V_{\text{FG}} = 0$, and that this voltage appeared essentially unchanged upon modulation of the field-effect gate bias to $V_{\text{FG}} = \pm 40$ V, provide strong evidence that there was no meaningful gate-leakage in this PBTTT device; this is consistent with the small gate-leakage current measured in the device shown in Supplementary Figure 56(f). We therefore attribute this $\Delta T = 0$ voltage as being associated with the instrument offset of the nanovoltmeter.[58, 9] The effect of the offset is excluded by taking the net thermoelectric signal to be the difference between the measured voltages in the presence and in the absence of ΔT, i.e. by taking the slope of the signal.

As ΔT was increased, we measured increasingly more negative (positive) potentials on high terminal of the nanovoltmeter in a cold (hot) grounded measurement. This is indicative of a p-type thermoelectric response, where the majority carriers, i.e. holes, drift from the hot to the cold side of the channel. Importantly, the change of the measured voltage with ΔT is almost entirely linear, which contrasts with the $\Delta T^{1/2}$ variation expected in the case of significant heater leakage (Supplementary Figure 55). We notice that for any ΔT, the signals for the different field-effect gate voltages overlap very well. Therefore, solely on basis of the variation of the hot or cold grounded signals (individually) with $\Delta T \propto V_{\text{heat}}$ and/or V_{FG}, there was no indication of the signals being compromised by gate and/or heater leakage.

We now consider the symmetry of the cold and hot grounded signals, as a further check for any potential leakage. We saw very symmetrical signals for both ground configurations for all the studied field-effect gate voltages. For instance, for $V_{\text{FG}} = 0$ we observed a Seebeck coefficient of 53.6 μV K^{-1} on a cold-grounded measurement, compared to 55.1 μV K^{-1} the hot-grounded. Similarly, for $V_{\text{FG}} = -40$ V, we see highly consistent coefficients of 53.8 μV K^{-1} for cold-ground and 55.2 μV K^{-1} for hot-ground; the values were 55.8 μV K^{-1} and 55.4 μV K^{-1} respectively for $V_{\text{FG}} = -40$ V. For ease of comparison, all these values are summarized in Supplementary Table 6. The very good agreement between the coefficients measured on cold and hot grounded experiments provides important reassurance that leakage is not a concern in these measurements.

Having established confidence in the measurements, we are now in position to interpret the signal trends. As evident in Supplementary Figure 56(g) and (h), even with the application of either +40 V or -40 V field-effect gate potentials, we saw less than 0.5% changes in the Seebeck coefficient. The fact that the coefficient is almost completely unaffected by field-effect gating is indicative of the absence of a Coulomb gap at 190 K for this highly doped state of PBTTT, which is consistent with the absence of non-linearity in the transfer

Supplementary Table 6: Seebeck coefficient values extracted from Supplementary Figure 56.

Polymer	V_{FG} [V]	Cold-Ground		Hot-Ground	
		S [μV/K]	Offset [μV]	S [μV/K]	Offset [μV]
IDT-BT	0	57.8	-0.71	60.5	-1.18
	-40	83.0	-0.59	66.0	-1.35
	+40	56.1	-0.54	50.3	-1.14
PBTTT	0	53.6	4.67	55.1	-5.24
	-40	53.8	4.11	55.2	-5.38
	+40	55.8	4.60	55.4	-5.22

curve (Supplementary Figure 56(f)). In the absence of a gap around the Fermi level E_F, we do not expect the slope of density-of-states $g(E)$, which is proportional to the Seebeck coefficient as discussed above, to change appreciably over the small modulation range of E_F possible by field-effect gating.

However, even in the absence of a Coulomb gap, theoretically we still expect that the Seebeck coefficient should remain field-effect modulated purely as a consequence of the change in carrier density. The Seebeck coefficient can be understood as the entropy carried per charge carrier. For relatively narrow-band transport in polymers the framework of Heikes formula has often been used to quantify the expected dependence of the Seebeck coefficient on mobile carrier concentration n:

$$S = \frac{k_B}{e} \ln\left(\frac{N-n}{n}\right) + \frac{k_B}{e} \ln(2), \tag{30}$$

where the first term relates the Seebeck coefficient to the change of the entropy of mixing associated with adding a carrier to the density of thermally accessible transport states, and the second term accounts for the entropy change associated with the two-fold spin degeneracy. k_B is the Boltzmann's constant, e is the magnitude of the elementary charge, and N is the number of the thermally accessible sites. In a typical organic field-effect transistor, $n << N$, such that the entropy of mixing dominates the Seebeck coefficient. From Heikes formula, for every ten-fold increase in n, we expect a decrease in the Seebeck coefficient by $(k_B/e)\ln 10 = 198$ μV K^{-1}. In our previous field-effect gated Seebeck measurements on conjugated polymer FETs, we have observed that Heikes formula describes the n-dependence of the Seebeck coefficient reasonably well in IDT-BT [48] and for hole-transport in DPP-BTz,[59] although for other DPP-polymers [59] we have observed significantly higher $\mathrm{d}S/\mathrm{d}\log n$, presumably reflecting their higher energetic disorders.

We can use Heikes formula to estimate the magnitude of the dependence of the Seebeck coefficient on carrier concentration. For this it is important consider that our present samples are highly doped. The upper limit of the changes in the carrier density due to field-effect modulation $\Delta n \sim 10^{19}$ cm^{-2} is roughly only 1% of the doped carrier density of $\sim 10^{21}$ cm^{-2}, as discussed in the main article. Supposing that $\mathrm{d}S/\mathrm{d}\log n$ from Heikes formula holds true in this carrier density regime, we expect a change of less than 2 μV K^{-1} associated with the field-effect modulation. With the 0.25 K temperature difference attainable in our present devices, the signal size that we could expect is on the order of 0.5 μV. Experimentally, we typically see voltage noise on the order of hundreds of nV in our experiments, limiting our ability to detect this small Heikes response.

10.3.2 IDT-BT

For the IDT-BT measurements, we chose doping states where a significant non-linearity and ambipolarity is observed on the transfer-curves, from which a large contribution of the Coulomb gap would be expected. We first discuss the measurements in Regime III, where the measurement is made easier by the high conductivity of $\sim$ 10 S cm^{-1}. The field-effect gate transfer curve of this doping state is shown in Supplementary Figure 56(c) along with the leakage current. The leakage current in this IDT-BT device is slightly larger than that of the PBTTT device, which is likely a consequence of the larger device architecture used for IDT-BT (Device E) compared to PBTTT (Device C), as shown in Supplementary Figure 1. The corresponding cold- and hot-grounded thermovoltage signals are shown in Supplementary Figures 56(d) and (e) respectively.

We note that there is again a voltage offset at $\Delta T = 0$ but with smaller magnitude than seen in PBTTT, ranging between -0.54 to -0.71 μV K^{-1} for cold-ground and -1.14 to -1.35 μV K^{-1} for hot-ground. Again this offset is quite consistent irrespective of the applied V_{FG}. Consequently, we conclude that gate leakage is not likely to dominate either the cold or hot grounded measurements, and that the $\Delta T = 0$ voltages are also the

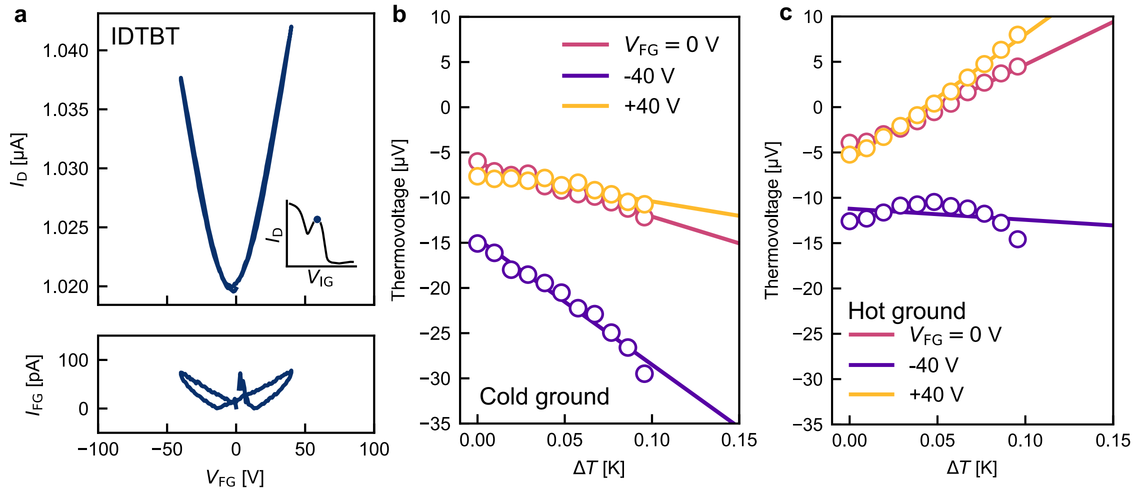

Supplementary Figure 57: **Double gated Seebeck measurements of a more resistive IDT-BT state.** (a) Field-effect gate transfer curve of IDT-BT on the conductivity peak. Thermovoltage measurements taken with (b) cold side and (c) hot side grounded. Notice that the hot- and cold-grounded signals are highly anti-symmetric.

manifestation of nanovoltmeter offset as in PBTTT. This is consistent with the low leakage current of ~ 100 pA seen in the field-effect gate transfer curve (Supplementary Figure 56(c)).

Under finite temperature differences ΔT, we note that both the cold-grounded and hot-grounded signals are still linearly proportional to ΔT. We did observe a larger voltage noise in the IDT-BT signal, as evident from the deviation of some datapoints from the expected linear response. We attribute this to Johnson or thermal noise of the resistive polymer channel. The larger voltage noise is likely to originate from IDT-BT's channel resistance ($\sigma \sim 10$ S cm^{-1}) being higher than PBTTT ($\sigma \sim 1000$ S cm^{-1}), recalling that the magnitude of Johnson noise is proportional to the square root of the resistance. Although a larger channel resistance could also lead to a larger thermovoltage error due to heater leakage ($V_{\mathrm{S1,heat}} \propto R_{\mathrm{S1}}$ as argued in Supplementary Equation 29), this is unlikely since the voltage fluctuation is not proportional $\Delta T^{1/2}$ as would be expected in the case of heater leakage (Supplementary Figure 55), but instead appears to be randomly distributed around a straight line.

We finally discuss the symmetry of the hot- and cold-grounded signals of IDT-BT. At $V_{\mathrm{FG}} = 0$, there is a good agreement between the Seebeck coefficient measured when grounding the cold-side (57.8 μV K^{-1}) and the hot-side (60.5 μV K^{-1}). We acknowledge that the symmetry is less ideal, compared to PBTTT, for measurements with non-zero field-effect gate potentials. For $V_{\mathrm{FG}} = +40$ V, we see a reduction of the coefficient of 3% on the cold-grounded measurement, which is significantly smaller than the nearly 17% reduction seen on the hot-grounded counterpart. Similarly, we see a larger increase of the cold-grounded coefficient of $\sim 44\%$ for $V_{\mathrm{FG}} = -40$ V, compared to the hot-grounded increase of 9%. We attribute this less ideal symmetry to the higher channel resistance of IDT-BT. In Supplementary Equations 28 and 29, the resistance of the channel R_{S1} is around two orders of magnitude higher for IDT-BT, such that even for the same dielectric performance and the same leakage current I_{L} we expect to see a more signficant effect of the leakage, as a larger thermovoltage error, on IDT-BT.

We emphasize, however, that we have observed the Seebeck coefficient to consistently be increased under negative field-effect gate voltages, and decreased under positive field-effect gate voltages irrespective of the grounding configuration. These trends could not have been solely by any form of leakage. For instance, if the signals were completely dominated by the application of positive field-effect gate or heater voltage, we would expect the coefficient to be increased on the cold-grounded measurement, and decreased on the hot-grounded measurement, as argued in Supplementary Figure 54 and 55. In the case of severe leakage effects, the symmetry would not just be less ideal; in such a case, there would not be any symmetry in the cold- and hot-grounded signals at all.

To appreciate this better, we now discuss the double-gated Seebeck measurement on a lower conductivity state, which in this case is the conductivity peak between Regimes I and II. On this state, we still see a significant ambipolarity of the transfer curve, although the channel resistance is higher by a factor of six compared to the second rise (compare Supplementary Figures 56(c) and 57(a)). We note that measurements on both states were performed on the same device. While we saw comparably low leakage currents on both states, we would expect the thermovoltage errors due to gate and heater leakage to be consequently higher by a factor of six in this more resistive state.

Supplementary Table 7: Seebeck coefficient values extracted from Supplementary Figure 57.

Polymer	V_{FG} [V]	Cold-Ground		Hot-Ground	
		S [μV/K]	Offset [μV]	S [μV/K]	Offset [μV]
IDT-BT	0	59.2	-6.19	94.2	-4.73
	-40	139	-14.6	-12.3	-11.2
	+40	31.9	-7.23	139	-5.93

As expected, we observe multiple signatures of leakage effects influencing the thermovoltage measurements. First, we notice that the offset for $V_{FG} = -40$ V was shifted to very negative values of -14.6 and -11.2 μV K^{-1} for the cold- and hot-grounded measurements respectively. This, as discussed in Supplementary Note 10.2.1, is an indication of thermovoltage error due to gate leakage. We did not see a similar signature for the case of positive V_{FG}; the reason for this is unclear, although we note that this might be related to the difference in the capacitance of the silicon oxide dielectric for positive and negative gate voltages.

Further leakage signatures are more evident when comparing the signal symmetry. The Seebeck coefficients at various V_{FG} and ground configurations for this device are summarized in Supplementary Table 7. Even at zero field-effect gate voltage, we saw a sizeable difference measurements using hot-ground (94.2 μV K^{-1}) and cold-ground (59.2 μV K^{-1}). Very importantly, for $V_{FG} = +40$ V, the measured coefficient became larger for the hot-grounded signal (139 μV K^{-1}), and smaller for the cold-grounded signal (31.9 μV K^{-1}). The fact that the size of the signal changes differently for hot- and cold-grounding strongly suggests that these changes are related to the field-effect gate voltage leakage (Supplementary Figure 54), as opposed to intrinsic thermoelectric effects of the channel material (Supplementary Figure 50, left panels). Consistently, for $V_{FG} = -40$ V, the asymmetry manifests as a larger cold-grounded signal, and a smaller hot-grounded counterpart. Comparing Supplementary Figures 57(b) and (c), there is a lack of symmetry between the hot- and cold-grounded signals, from which we conclude that the measured voltages in this case as being dominated by leakage errors. In contrast, the more symmetrical signals in Supplementary Figures 56(d) and (e) show that for the more conducting state in Regime III the signal is dominated by intrinsic response of the channel material. We opt for the term 'dominated' here to acknowledge that gate leakage is most probably still present in the Regime III measurement (as the measurement was performed on the same device as the Regime I data in Supplementary Figure 56), however its contribution is not significant enough to mask the intrinsic Seebeck coefficient changes.

These intrinsic changes in the Seebeck coefficient are indicative of the presence of a Coulomb gap centred at the Fermi level. We recall our arguments based on the Boltzmann transport framework (Supplementary Note 10.1, Supplementary Figures 50 and 51) that the Seebeck coefficient in Regime I and III should become more positive as the Fermi level shifts towards more negative energies, and on the other extreme the coefficient should become increasingly negative at positive-enough energies. Experimentally, applying negative field-effect gate voltages is equivalent to shifting the Fermi level to more negative energies in the density-of-states, and the opposite is true for positive field-effect gate voltages. For positive field-effect gate voltage we observed a reduction in the p-type Seebeck response, as opposed to the coefficient becoming negative as argued in Supplementary Figures 50 and 51. This is best understood by taking into account that the total Seebeck coefficient that is measured S_{total} as being a conductance-weighted average of the coefficients of the bulk S_{bulk} and the field-effect modulated channel locally on the dielectric/polymer interface $S_{\text{field-effect}}$:

$$S_{\text{total}} = \frac{S_{\text{bulk}} \times G_{\text{bulk}} + S_{\text{field-effect}} \times G_{\text{field-effect}}}{G_{\text{bulk}} + G_{\text{field-effect}}}, \tag{31}$$

where G_{bulk} and $G_{\text{field-effect}}$ denote the conductance values of the bulk and the field-effect modulated channel respectively. The measured signals in Supplementary Figure 56 and 57 represent S_{total}, therefore its reduction at positive field-effect gate voltages can be understood as a consequence of $S_{\text{field-effect}}$ turning negative, or at least being smaller than S_{bulk}. We conclude that the trends seen in Supplementary Figure 56 are qualitatively fully consistent with the expectations from the Boltzmann transport framework.

Although in our experiments so far there is no way to access directly the thermopower of the field-effect modulated carriers $S_{\text{field-effect}}$, Supplementary Equation 31 can be used to estimate this value. We assume for simplicity that the conductivities of the bulk and field-effect modulated channel are the same, i.e. $\sigma_{\text{bulk}} = \sigma_{\text{field-effect}}$. In effect, this assumption signifies that there is no field-effect modulation of the channel conductance, which is not unreasonable (see state #14 of Figure 2 in the main text). The second assumption is that out of the total polymer thickness of 10 nm, only the carriers in the first nm experiences field-effect

Supplementary Table 8: $S_{\text{field-effect}}$ extracted from Supplementary Equation 31 and Supplementary Table 6.

Polymer	V_{FG} [V]	Cold-Ground $S_{\text{field-effect}}$ [μV/K]	Hot-Ground $S_{\text{field-effect}}$ [μV/K]
IDT-BT	-40	310	116
	+40	40.8	-41.5

modulation. From the two assumptions the conductance-weighting is then reduced a thickness-weighting problem, with $G_{\text{bulk}} = 9\ G_{\text{field-effect}}$ solely due to the ratio of their thicknesses. The inferred values of the Seebeck coefficient of only the field-effect modulated carriers $S_{\text{field-effect}}$ are summarized in Supplementary Table 8.

References

[1] W. Zhang, J. Smith, S.E. Watkins, R. Gysel, M. McGehee, A. Salleo, J. Kirkpatrick, S. Ashraf, T. Antopoulos, M. Heeney, and I. McCulloch. Indacenodithiophene Semiconducting Polymers for High-Performance, Air-Stable Transistors. *J. Am. Chem. Soc.*, 132:11437–11439, 2010.

[2] M. Gruber, S.-H. Jung, S. Schott, D. Venkateshvaran, A.J. Kronemeijer, J.W. Andreasen, C.R. McNeill, W.W.H. Wong, M. Shahid, M. Heeney, J.-K. Lee, and H. Sirringhaus. Enabling high-mobility, ambipolar charge-transport in a DPP-benzotriazole copolymer by side-chain engineering. *Chem. Sci.*, 6(12):6949–6960, 2015.

[3] I. McCulloch, M. Heeney, C. Bailey, K. Genevicius, I. MacDonald, M. Shkunov, D. Sparrowe, S. Tierney, R. Wagner, W. Zhang, M.L. Chabinyc, R.J. Kline, M.D. McGehee, and M.F. Toney. Liquid-crystalline semiconducting polymers with high charge-carrier mobility. *Nat. Mater.*, 5(4):328–333, 2006.

[4] Y. Huang, D.H.L. Tjhe, I.E. Jacobs, X. Jiao, Q. He, M. Statz, X. Ren, X. Huang, I. McCulloch, M. Heeney, C. McNeill, and H. Sirringhaus. Design of experiment optimization of aligned polymer thermoelectrics doped by ion-exchange. *Appl. Phys. Lett.*, 119(11):111903, 2021.

[5] I.E. Jacobs, Y. Lin, Y. Huang, X. Ren, D. Simatos, C. Chen, D.H.L. Tjhe, M. Statz, L. Lai, P.A. Finn, W.G. Neal, G.D'Avino, V. Lemaur, S. Fratini, D. Beljonne, J. Strzalka, C.B. Nielsen, S. Barlow, S.R. Marder, I. McCulloch, and H. Sirringhaus. High-Efficiency Ion-Exchange Doping of Conducting Polymers. *Adv. Mater.*, 34(22):2102988, 2021.

[6] I.E. Jacobs, G. D'Avino, V. Lemaur, Y. Lin, Y. Huang, C. Chen, T.F. Harrelson, W. Wood, L.J. Spalek, T. Mustafa, C.A. O'Keefe, X. Ren, D. Simatos, D.H.L. Tjhe, M. Statz, J.W. Strzalka, J.-K. Lee, I. McCulloch, S. Fratini, D. Beljonne, and H. Sirringhaus. Structural and Dynamic Disorder, Not Ionic Trapping, Controls Charge Transport in Highly Doped Conducting Polymers. *J. Am. Chem. Soc.*, 144:3005–3019, 2022.

[7] J.-F. Chang, M.C. Gwinner, M. Caironi, T. Sakanoue, and H. Sirringhaus. Conjugated-Polymer-Based Lateral Heterostructures Defined by High-Resolution Photolithography. *Adv. Funct. Mater.*, 20(17):2825–2832, 2010.

[8] K. H. Lee, M.S. Kang, S. Zhang, Y. Gu, T.P. Lodge, and C.D. Frisbie. "Cut and Stick" Rubbery Ion Gels as High Capacitance Gate Dielectrics. *Adv. Mater.*, 24(32):4457–4462, 2012.

[9] Keithley K2182A Nanovoltmeter Data Sheet. Available online at `https://download.tek.com/datasheet/2182A-15912.pdf`.

[10] Keithley K2600B System SourceMeter Datasheet. Available online at `https://download.tek.com/datasheet/1KW-60906-0_Series_2600BDatasheet_112718.pdf`.

[11] Keithley K6430 Sub-femtoamp Remote SourceMeter SMU Instrument Datasheet. Available online at `https://download.tek.com/datasheet/6430.pdf`.

[12] User's Guide: Agilent 4155B Semiconductor Parameter Analyzer and Agilent 4156B Precision Semiconductor Parameter Analyzer. Available online at `https://www.keysight.com/us/en/assets/9018-07927/user-manuals/9018-07927.pdf`.

[13] S. Watanabe, M. Ohno, Y. Yamashita, T. Terashige, H. Okamoto, and J. Takeya. Validity of the Mott formula and the origin of thermopower in π-conjugated semicrystalline polymers. *Phys. Rev. B*, 100(24), 2019.

[14] K.G. Cho, D.Z. Adrahtas, K.H. Lee, and C.D. Frisbie. Sub-Band Filling and Hole Transport in Polythiophene-Based Electrolyte-Gated Transistors: Effect of Side-Chain Length and Density. *Adv. Funct. Mater.*, 2023.

[15] Alan B Kaiser. Systematic Conductivity Behavior in Conducting Polymers: Effects of Heterogeneous Disorder. *Adv. Mater.*, 13:927–941, 2001.

[16] S. Winkler, P. Amsalem, J. Frisch, M. Oehzelt, G. Heimel, and N. Koch. Probing the energy levels in hole-doped molecular semiconductors. *Mater. Horizons*, 2(4):427–433, 2015.

[17] R. Ghosh and F.C. Spano. Excitons and Polarons in Organic Materials. *Acc. Chem. Res.*, 53(10):2201–2211, 2020.

[18] R. Ghosh, A.R. Chew, J. Onorato, V. Pakhnyuk, C.K. Luscombe, A. Salleo, and F.C. Spano. Spectral Signatures and Spatial Coherence of Bound and Unbound Polarons in P3HT Films: Theory Versus Experiment. *J. Phys. Chem. C*, 122:18048–18060, 2018.

[19] H. Tanaka, M. Hirate, S. Watanabe, and S.-I. Kuroda. Microscopic signature of metallic state in semicrystalline conjugated polymers doped with fluoroalkylsilane molecules. *Adv. Mater.*, 26(15):2376–2383, 2014.

[20] H. Tanaka, S. Nishio, H. Ito, and S.-I. Kuroda. Microscopic signature of insulator-to-metal transition in highly doped semicrystalline conducting polymers in ionic-liquid-gated transistors. *Appl. Phys. Lett.*, 107(24):243302, 2015.

[21] X. Zhang, H. Bronstein, A.J. Kronemeijer, J. Smith, Y. Kim, R.J. Kline, L.J. Richter, T.D. Anthopoulos, H. Sirringhaus, K. Song, M. Heeney, W. Zhang, I. McCulloch, and D.M. DeLongchamp. Molecular origin of high field-effect mobility in an indacenodithiophene–benzothiadiazole copolymer. *Nat. Commun.*, 4(1):2238, 2013.

[22] C. Cendra, L. Balhorn, W. Zhang, K. O'Hara, K. Bruening, C.J. Tassone, H.G. Steinrück, M. Liang, M.F. Toney, I. McCulloch, M.L. Chabinyc, A. Salleo, and C.J. Takacs. Unraveling the Unconventional Order of a High-Mobility Indacenodithiophene-Benzothiadiazole Copolymer. *ACS Macro Lett.*, 10(10):1306–1314, 2021.

[23] J. Rivnay, S.C.B. Mannsfeld, C.E. Miller, A. Salleo, and M.F. Toney. Quantitative determination of organic semiconductor microstructure from the molecular to device scale. *Chem. Rev.*, 112(10):5488–5519, 2012.

[24] W. Liu, L. Müller, S. Ma, S. Barlow, S.R. Marder, W. Kowalsky, A. Köhn, and R. Lovrincic. Origin of the π–π Spacing Change upon Doping of Semiconducting Polymers. *J. Phys. Chem. C*, 122(49):27983–27990, 2018.

[25] C.G. Bischak, L.Q. Flagg, K. Yan, T. Rehman, D.W. Davies, R.J. Quezada, J.W. Onorato, C.K. Luscombe, Y. Diao, C.-Z. Li, and D.S. Ginger. A reversible structural phase transition by electrochemically-driven ion injection into a conjugated polymer. *J. Am. Chem. Soc.*, 142(16):7434–7442, 2020.

[26] A.C. Mayer, M.F. Toney, S.R. Scully, J. Rivnay, C.J. Brabec, M. Scharber, M. Koppe, M. Heeney, I. McCulloch, and M.D. McGehee. Bimolecular crystals of fullerenes in conjugated polymers and the implications of molecular mixing for solar cells. *Adv. Funct. Mater.*, 19(8):1173–1179, 2009.

[27] R. Dovesi, R. Orlando, A. Erba, C.M. Zicovich-Wilson, B. Civalleri, S. Casassa, L. Maschio, M. Ferrabone, M De La Pierre, P. D'Arco, Y. Noël, M. Causà, M. Rérat, and B. Kirtman. CRYSTAL14: A program for the *ab initio* investigation of crystalline solids. *Int. J. Quantum Chem.*, 114(19):1287–1317, 2014.

[28] J.P. Perdew, M. Ernzerhof, and K. Burke. Rationale for mixing exact exchange with density functional approximations. *J. Chem. Phys.*, 105(22):9982–9985, 1996.

[29] H.J. Monkhorst and J.D. Pack. Special points for Brillouin-zone integrations. *Phys. Rev. B*, 13:5188–5192, 1976.

[30] F. Neese. The ORCA program system. *Wiley Interdiscip. Rev. Comput. Mol. Sci.*, 2(1):73–78, 2012.

[31] G. Brocks, J. van den Brink, and A.F. Morpurgo. Electronic Correlations in Oligo-acene and -Thiopene Organic Molecular Crystals. *Phys. Rev. Lett.*, 93:146405, 2004.

[32] A. Vaknin, Z. Ovadyahu, and M. Pollak. Nonequilibrium field effect and memory in the electron glass. *Phys. Rev. B*, 65(13), 2002.

[33] T. Grenet, J. Delahaye, M. Sabra, and F. Gay. Anomalous electric-field effect and glassy behaviour in granular aluminium: electron glass? *Phys. Status Solidi C*, 5(3):680–683, 2008.

[34] J. Delahaye and T. Grenet. Electron glass signatures up to room temperature in disordered insulators. *J. Phys. Condens. Matter*, 34(13):135603, 2022.

[35] H.H. Choi, K. Cho, C.D. Frisbie, H. Sirringhaus, and V. Podzorov. Critical assessment of charge mobility extraction in FETs. *Nat. Mater.*, 17, 2018.

[36] A.L. Efros and B.I. Skhlovskii. Coulomb gap and low temperature conductivity of disordered systems. *J. Phys. C*, 8:49–51, 1975.

[37] H. Sirringhaus, P.J. Brown, R.H. Friend, M.M. Nielsen, K. Bechgaard, B.M.W. Langeveld-Voss, A.J.H. Spiering, R.A.J. Janssen, E.W. Meijer, P. Herwig, and D.M. de Leeuw. Two-dimensional charge transport in self-organized, high-mobility conjugated polymers. *Nature*, 401:685–688, 1999.

[38] K. Kang, S. Watanabe, K. Broch, A. Sepe, A. Brown, I. Nasrallah, M. Nikolka, Z. Fei, M. Heeney, D. Matsumoto, K. Marumoto, H. Tanaka, S. Kuroda, and H. Sirringhaus. 2D coherent charge transport in highly ordered conducting polymers doped by solid state diffusion. *Nat. Mater.*, 15(8):896–902, 2016.

[39] N.F. Mott. Metal-Insulator Transition. *Rev. Mod. Phys.*, 40(4):677–683, 1968.

[40] C.P. Slichter. *Principles of magnetic resonance*, volume 1. Springer Science & Business Media, 2013.

[41] W.J. Baker, T.L. Keevers, J.M. Lupton, D.R. McCamey, and C. Boehme. Slow hopping and spin dephasing of coulombically bound polaron pairs in an organic semiconductor at room temperature. *Phys. Rev. Lett.*, 108(26):267601, 2012.

[42] S. Schott, U. Chopra, V. Lemaur, A. Melnyk, Y. Olivier, R. Di Pietro, I. Romanov, R.L. Carey, X. Jiao, C. Jellett, M. Little, A. Marks, C.R. McNeill, I. McCulloch, E.R. McNellis, D. Andrienko, D. Beljonne, J. Sinova, and H. Sirringhaus. Polaron spin dynamics in high-mobility polymeric semiconductors. *Nat. Phys.*, 15(8):814–822, 2019.

[43] R.A. Evarestov. *Quantum Chemistry of Solids.* Springer Berlin Heidelberg, 2007.

[44] A.-K. Tornberg. The Ewald sums for singly, doubly and triply periodic electrostatic systems. *Adv. Comput. Math.*, 42(1):227–248, 2016.

[45] S. Ciuchi and S. Fratini. Hopping dynamics of interacting polarons. *Phys. Rev. B*, 79:035113, 2009.

[46] Y. Pramudya, H. Terletska, S. Pankov, E. Manousakis, and V. Dobrosavljević. Nearly frozen Coulomb liquids. *Phys. Rev. B*, 84:125120, 2011.

[47] D. Di Sante, S. Fratini, Vladimir Dobrosavljević, and S. Ciuchi. Disorder-Driven Metal-Insulator Transitions in Deformable Lattices. *Phys. Rev. Lett.*, 118:036602, 2017.

[48] D. Venkateshvaran, M. Nikolka, A. Sadhanala, V. Lemaur, M. Zelazny, M. Kepa, M. Hurhangee, A.J. Kronemeijer, V. Pecunia, I. Nasrallah, I. Romanov, K. Broch, I. McCulloch, D. Emin, Y. Olivier, J. Cornil, D. Beljonne, and H. Sirringhaus. Approaching disorder-free transport in high-mobility conjugated polymers. *Nature*, 515(7527):384–388, 2014.

[49] S. Fratini, D. Mayou, and S. Ciuchi. The Transient Localization Scenario for Charge Transport in Crystalline Organic Materials. *Adv. Funct. Mater.*, 26(14):2292–2315, 2016.

[50] S. Fratini and S. Ciuchi. Dynamical localization corrections to band transport. *Phys. Rev. Research*, 2:013001, 2020.

[51] K.E. Ziemelis, A.T. Hussain, D.D.C. Bradley, R.H. Friend, J. Rühe, and G. Wegner. Optical spectroscopy of field-induced charge in poly (3-hexyl thienylene) metal-insulator-semiconductor structures: Evidence for polarons. *Phys. Rev. Lett.*, 66(17):2231, 1991.

[52] R. Uchida, H. Yada, M. Makino, Y. Matsui, K. Miwa, T. Uemura, J. Takeya, and H. Okamoto. Charge modulation infrared spectroscopy of rubrene single-crystal field-effect transistors. *Appl. Phys. Lett.*, 102(9), 2013.

[53] M. C. Abrams, G. C. Toon, and R. A. Schindler. Practical example of the correction of Fourier-transform spectra for detector nonlinearity. *Appl. Opt.*, 33(27):6307–6314, 1994.

[54] J. Krupka, J. Breeze, A. Centeno, N. Alford, T. Claussen, and L. Jensen. Measurements of permittivity, dielectric loss tangent, and resistivity of float-zone silicon at microwave frequencies. *IEEE Trans. Microw. Theory Tech.*, 54(11):3995–4001, 2006.

[55] S.S. Li and W.R. Thurber. The dopant density and temperature dependence of electron mobility and resistivity in n-type silicon. *Solid State Electron.*, 20(7):609–616, 1977.

[56] H. Fritzsche. A general expression for the thermoelectric power. *Solid State Commun.*, 9:1813–1815, 1971.

[57] S.D. Kang and G.J. Snyder. Charge-transport model for conducting polymers. *Nat. Mater.*, 16(2):252–257, 2016.

[58] R. Fletcher, V.M. Pudalov, A.D.B. Radcliffe, and C. Possanzini. Critical behaviour of thermopower and conductivity at the metal-insulator transition in high-mobility Si-MOSFETs. *Semicond. Sci. Technol.*, 16:386–393, 2001.

[59] K. Broch, D. Venkateshvaran, V. Lemaur, Y. Olivier, D. Beljonne, M. Zelazny, I. Nasrallah, D.J. Harkin, M. Statz, R.D. Pietro, A.J. Kronemeijer, and H. Sirringhaus. Measurements of Ambipolar Seebeck Coefficients in High-Mobility Diketopyrrolopyrrole Donor-Acceptor Copolymers. *Adv. Electron. Mater.*, 3(11):1700225, 2017.
